# Synthesis and Anticancer Activity of A-Ring-Modified Derivatives of Dihydrobetulin

**DOI:** 10.3390/ijms24129863

**Published:** 2023-06-07

**Authors:** Irina Tolmacheva, Yulia Beloglazova, Mikhail Nazarov, Olga Gagarskikh, Victoria Grishko

**Affiliations:** Perm Federal Scientific Centre, Institute of Technical Chemistry UB RAS, Academician Korolev St. 3, 614013 Perm, Russia; tolmair@gmail.com (I.T.); beloglazova.y@itcras.ru (Y.B.); mihailnazarov705@gmail.com (M.N.); gagarsckih.olga@yandex.ru (O.G.)

**Keywords:** cytotoxicity, drug resistance, P-glycoprotein, apoptosis, intrinsic pathway, ROS, triterpenoids, dihydrobetulin

## Abstract

Multidrug resistance (MDR) is a common phenomenon in clinical oncology, whereby cancer cells become resistant to chemotherapeutic drugs**.** A common MDR mechanism is the overexpression of ATP-binding cassette efflux transporters in cancer cells, with P-glycoprotein (P-gp) being one of them. New 3,4-seco-lupane triterpenoids, and the products of their intramolecular cyclization with the removed 4,4-gem-dimethyl group, were synthesized by the selective transformations of the A-ring of dihydrobetulin. Among the semi-synthetic derivatives, the MT-assay-enabled methyl ketone **31** (**MK**), exhibiting the highest cytotoxicity (0.7–16.6 µM) against nine human cancer cell lines, including P-gp overexpressing subclone HBL-100/Dox, is identified. In silico, **MK** has been classified as a potential P-gp-inhibitor; however, the Rhodamine 123 efflux test, and the combined use of P-gp-inhibitor verapamil with **MK** in vitro, showed the latter to be neither an inhibitor nor a substrate of P-gp. As the studies have shown, the cytotoxic effect of **MK** against HBL-100/Dox cells is, arguably, induced through the activation of the ROS-mediated mitochondrial pathway, as evidenced by the positive Annexin V-FITC staining of apoptotic cells, the cell cycle arrest in the G0/G1 phase, mitochondrial dysfunction, cytochrome *c* release, and the activation of caspase-9 and -3.

## 1. Introduction

Over centuries, people have been employing bioactive natural compounds for medicinal purposes, e.g., as part of medicinal plant extracts. Therefore, it is not surprising that, with the development and upgrade of chemical methods (extraction, chromatography, and physicochemical techniques for identifying individual compounds), and within such sections of chemistry as phytochemistry, the chemistry of natural compounds and medicinal chemistry, a great deal of natural compounds and their semisynthetic derivatives began to be actively employed as medicinal products. Thus, over the period 1981–2019, about 25% of approved anticancer drugs are either small molecules of natural origin, or derivations from those [1].

Currently, chemotherapy occupies a leading position in clinical oncology amid the available cancer treatments, such as radiation therapy, surgery, immunotherapy, or these methods combined. Simultaneously, the use of chemotherapy in cancer treatment provokes the development of multidrug resistance (MDR), when tumors become tolerant to multiple anticancer drugs. Multiple MDR mechanisms limit the pharmacological efficacy of cancer treatment and are also responsible for the majority of cancer recurrences and are associated with high rates of cancer mortality [2]. One of the MDR mechanism’s developments is related to the overexpression of a key member of the ATP-binding cassette (ABC) family of transporters, P-glycoprotein (P-gp), a transmembrane drug carrier that promotes the efflux of endogenous xenobiotics through cell membranes [2,3]. P-gp is the best characterized efflux pump whose subcellular expression can mediate MDR in a variety of cancers such as breast cancer, colorectal cancer, ovarian cancer, and lung cancer [3,4]. P-gp inhibitors can reverse drug resistance and, in combination with anticancer drugs, restore the ability of the latter to accumulate in cancer cells, thereby increasing the effectiveness of chemotherapy treatment [5]. The failure of clinical studies of P-gp inhibitors, caused by their severe side effects, only emphasizes the urgent demand for developing new reverse agents that would inhibit the efflux functions of the ABC transporter [6].

Since most anticancer drugs do not necessarily work well against many of the resistance mechanisms expressed in surviving cells, and the use of targeted agents does not always lead to a cure, there has been renewed interest in the search for more cytotoxic anticancer drugs in the natural environment [4]. Concurrently, natural compounds, and their semisynthetic derivatives, represent a significant alternative to synthetic compounds for the development of promising P-gp inhibitors [7,8]. For example, plant-derived pentacyclic lupane-type triterpenoids represent a valuable source of new effective inhibitors of cancer cell proliferation and angiogenesis, and are often able to distinguish cancer and normal cells, selectively interacting with cancer cells [9,10,11,12,13]. Furthermore, Laiolo et al. [14] have provided important evidence that betulin, a lupane triterpenoid widely distributed in nature, may be a safe and worthwhile compound for developing novel agents able to help overcome P-gp-mediated drug efflux. This conclusion is supported by the discovery of P-gp-inhibiting properties in the case of structurally related betulin derivatives. Therefore, betulinic acid has been shown to have anticancer activity against MDR cancer cells overexpressing ABC transporters, including P-gp [15]. 23-Hydroxybetulinic acid has an anticancer effect and re-sensitizes chemotherapy-resistant cancer cells, while its piperidinyl derivatives act as specific P-gp-reversing agents significantly enhancing the toxicity of doxorubicin (Dox), vincristine, and paclitaxel [16,17]. Recently, we have demonstrated the ability of betulin derivatives with a modified A-ring to effectively suppress the expression of the ABC transporter genes (*MDR1*, *MRP*, *MVP*, and *BCRP*) and the transport function of P-gp [18,19]. In subtoxic concentrations, these triterpenoids can induce a 10-fold increase in the sensitivity of Dox-resistant cancer cells to Dox [18]. Taking into account that steroid derivatives, such as triterpenoids, can modulate a variety of effects on P-gp [5], we herein report the synthesis of new dihydrobetulin derivatives, including A-seco-derivatives and “triterpenoid-steroid” hybrids, as well as presenting an evaluation of their cytotoxicity against several cancer cell lines, including the Dox-resistant subclone.

## 2. Results and Discussion

### 2.1. Chemistry

**Scheme 1:**
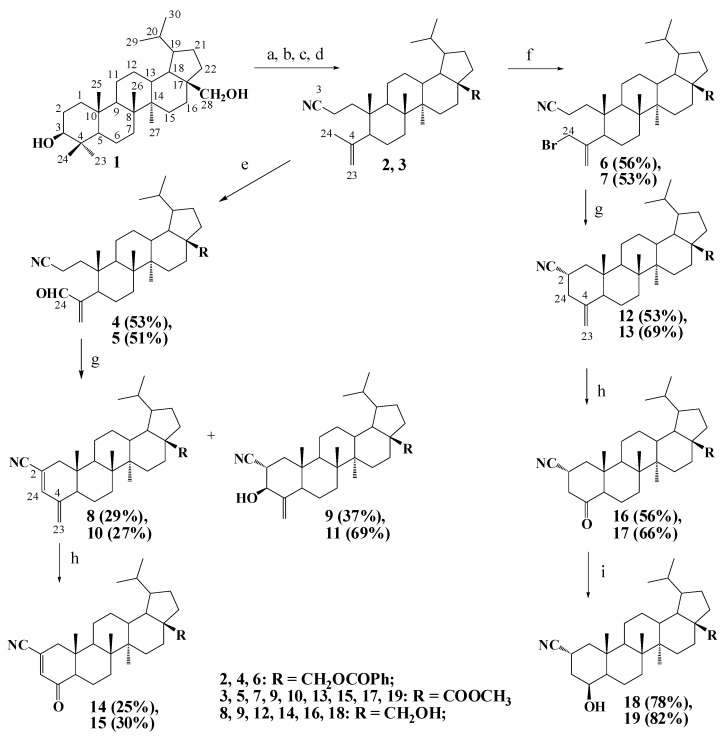
Synthesis of compounds **4**–**19**. Reagents and conditions: (a) PhCOCl, Py, 25 °C; (b) CrO_3_, acetone, H_2_SO_4_; (c) NH_2_OH·HCl, Py, reflux; (d) TsCl, Py, reflux; (e) H_2_SeO_3_, 1,4-dioxane, reflux; (f) NBS, CCl_4_, reflux; (g) *t*-BuOK, *t*-BuOH, reflux; (h) O_3_, CH_2_Cl_2_, −50 °C; and (i) NaBH_4_, MeOH, reflux.

Previously, we had shown the intramolecular nitrile anionic cyclization as being able to be carried out, owing to the methylene protons in α-position, to the nitrile group and an electrophilic center of *CN*-containing A-seco-triterpenoids [20,21,22]. Here, we present the synthesis and chemical modifications of novel A-seco-lupane derivatives. The starting 3,4-seco-lupane 3-nitriles **2** [23] and **3** [24] were obtained from dihydrobetulin **1** (Scheme 1). The oxidation with H_2_SeO_3_, or allyl bromination with *N*-bromosuccinimide (NBS) of nitriles **2** and **3**, led to the corresponding 24-aldehydes **4**, **5** [25] or 24,30-dibromo derivatives **6**, **7** (Scheme 1). The intramolecular cyclization of compounds **4**–**7** proceeded in *t*-BuOH in the presence of *t*-BuOK with the formation of 2-cyano-derivatives **8**–**13**. 4-Oxo derivatives **14**–**17** were obtained from compounds **8**, **10**, **12**, and **13** by ozonolysis (Scheme 1). The reduction in ketonitriles **16**, **17** with sodium borohydride produced 4β-hydroxy derivatives **18**, **19**.

**Scheme 2:**
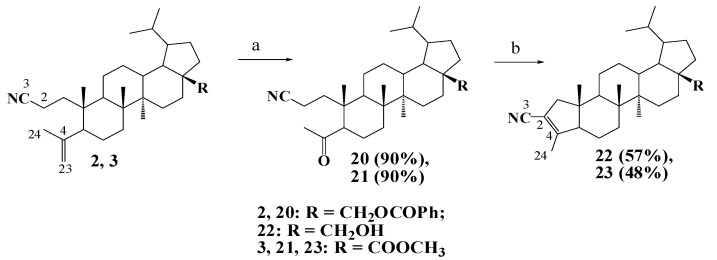
Synthesis of compounds **20**–**23**. Reagents and conditions: (a) O_3_, CH_2_Cl_2_, −50 °C and (b) *t*−BuOK, *t*−BuOH, reflux.

The ozonolysis of the exomethylene fragment in 3,4-seco-lupane triterpenoids **2**, **3** gave rise to methyl ketones **20**, **21** which readily underwent intramolecular oxonitrile cyclization in *t*-BuOH in the presence of *t*-BuOK to form compounds **22**, **23** containing an α,β-unsaturated nitrile fragment in the pentacyclic A-ring (Scheme 2).

**Scheme 3:**
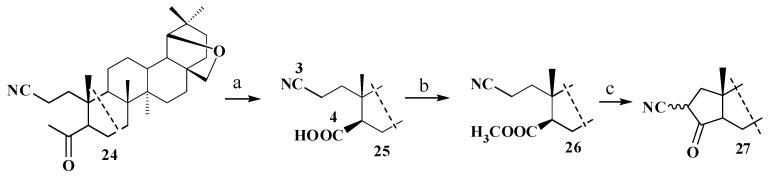
Synthesis of 2-cyano-19β,28-epoxy-4-oxo-3,23,24-trinor-18αH-oleanane **27**. Reagents and conditions: (a) 3 eq. C_5_H_5_NHBr_3_, CH_3_COOH, reflux; (b) CH_2_N_2_, C_2_H_5_OC_2_H_5_, 0–5 °C; and (c) *t*-BuOK, THF, reflux.

Previously, the exhaustive bromination of 2-cyano-3,4-seco-oleanane methyl ketone **24**, followed by elimination of CHBr_3_, was evinced to lead to the formation of 2-cyano-3,4-seco-3,23,24-trinor-18αH-olean-4-oic acid **25**, the methyl ester **26** of which was cyclized to A-pentacyclic ketonitrile **27** [22] (Scheme 3).

**Scheme 4:**
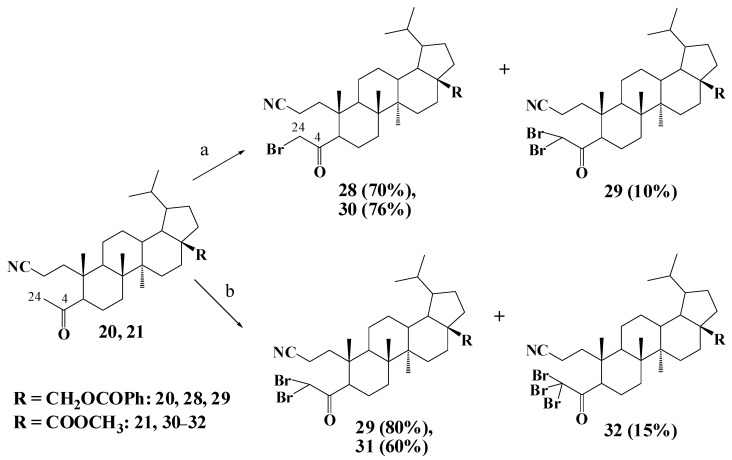
Synthesis of compounds **28**–**32**. Reagents and conditions: (a) 1 eq. C_5_H_5_NHBr_3_, AcOH, 25 °C, 24 h; (b) 3 eq. C_5_H_5_NHBr_3_, AcOH, reflux.

We have studied the reactions of methyl ketones **20** and **21** with pyridinium bromide perbromide (C_5_H_5_NHBr_3_) in acetic acid. Methyl ketone **20**, with an equimolar amount of C_5_H_5_NHBr_3_ at room temperature, gave a mixture of 24-mono- (**28**) and 24,24-dibromo- (**29**) derivatives in 70% and 10% yields, respectively. In turn, methyl ketone **21** formed only 24-monobromo derivative **30** in 76% yield under these conditions (Scheme 3). The reaction of methyl ketone **20** with an excess of C_5_H_5_NHBr_3_ under reflux resulted in the formation of only dibromo derivative **29** (80%), while methyl ketone **21** gave, under analogous conditions, a mixture of di- (**31**, 60%) and tri- (**32**, 15%) bromo-substituted methyl ketones (Scheme 4).

It should be noted that, in contrast with the oleanane analogs, the elimination of CHBr_3_ with the formation of 2-cyano-4-oic acids did not occur during the exhaustive bromination of methyl ketones **20**, **21**, regardless of the molar excess of C_5_H_5_NHBr_3_ and the boiling duration of the reaction mixture.

The structures of the new compounds were confirmed by the 1D NMR (^1^H-NMR, ^13^C-NMR) and MS methods. The determination of the configuration at C(2) and C(3) stereogenic centers in compounds **9** and **11**, as well as at C(2) and C(4) in compounds **18** and **19**, was carried out using the data of 2D NMR spectra (HMBC, HSQC, NOESY, COZY) of compounds **11** and **19**, with the results shown in the Experimental Section and Supplementary Materials.

### 2.2. Biology

The antiproliferative activity of the synthesized lupane derivatives was evaluated by the MTT assay against a panel of cancer cell lines including MCF-7 (breast), HCT116 (colon), RD TE32 (soft tissue sarcoma), MS (melanocytes), A549 (lung), PC-3 (prostate), HEpG2 (liver), HBL-100 (breast, the cells immortalized by SV-40 virus and turned tumorigenic owing to protracted cultivation [26]), and HBL-100/Dox (breast, P-gp overexpressed cell line) [18]. In this study, the human embryonic kidney cell line HEK293 was used as the non-cancerous cell line.

The screening studies (Table S1) had shown most of the cyclic and fragmented derivatives to be devoid of cytotoxicity—compounds **4**–**11**, **13**, **15**–**18**, **20**–**23**, **28**, and **29**—or to exhibit relatively weak or moderate cytotoxicity—compounds **12**, **14**, **19**, and **30** (Table 1). Only compound **31** (**MK**) had a pronounced cytotoxic effect, with the IC_50_ values 0.7–7.1 μM against RD TE32, HBL-100, HBL-100/Dox, HCT116, A549, and MCF-7 cell lines.

The structure–activity relationship (SAR) trends are summarized as follows. The structural variations around the C-28 position of the lupane skeleton of 24-bromo-derivatives had a strong influence. This was, probably, the outcome of steric hindrance, on the part of the bulky 28-benzoyl substituent, by the manifestation of cytotoxic activity by compounds **28**, **29**. The presence of 28-carbomethoxy substituent gave rise to major enhancement of potency, as exemplified by derivatives **30**, **31**. The degree of cytotoxicity was directly related to the amount of bromine atoms in the 3,4-seco-lupane fragment. High cytotoxic values (3.6–13.5 μM) of 24-monobromethyl ketone **30** were observed relative to the HBL-100/Dox, HBL-100, HCT116, and RD TE32 cells, whereas the emergence of two bromine atoms in the structure of methyl ketone **31** conspicuously heightened cytotoxicity against all the cell lines (0.7–16.6 μM). The 3,4-seco-lupane cyclization products were mostly non-cytotoxic. However, derivatives **12**, **14**, and **19** showed moderate activity (16.0–77.8 μM) against almost all the tested cancer cell lines, with the relationship between the structure and cytotoxicity of these compounds not being evident.

The selectivity index (SI) for the most active compounds was calculated as a simple ratio of IC_50_ values between healthy and cancer cells in order to assess the selective toxic effect of the studied derivatives **12**, **14**, **19**, **30**, and **31** against cancer cells and predict their therapeutic potential. As is apparent from Table S2, 24,24-dibromo-substituted methyl ketone **31** (**MK**) displayed the best SI, especially against HBL-100/Dox, HBL-100, and RD TE32 cells (SI = 6.39–16.43).

Currently, plant-derived natural products (including triterpenoids and their derivatives) with selective cytotoxicity against cancer cells are also regarded as a worthwhile source of new therapeutic MDR reversal agents characterized by P-gp inhibitors [7,27,28]. The synthesized **MK** proved to be the most active compound against the P-gp-overexpressing cell line HBL-100/Dox. A detailed analysis of individual molecular properties [29] showed molecular hydrophobicity (MlogP) to be a determining descriptor for identifying P-gp inhibitors. The hydrophobicity, calculated by the Web-tools (MlogP > 2.93 [30]), predicted the P-glycoprotein-binding inhibitory properties as being foreseeable for **MK** (Table 2).

The interactions between the compound and P-gp leading to MDR reversion may take place due to the competition between substrates for P-gp or the non-competitive inhibition of P-gp activity. The prediction of a possibility for the compounds to act as a substrate or an inhibitor of P-gp, as performed using freely accessible Web-tools ADMETlab 2.0 (https://admetmesh.scbdd.com/ (accessed on 1 March 2023)), pkCSM (http://biosig.unimelb.edu.au/ (accessed on 1 March 2023)), and PgpRules (https://pgprules.cmdm.tw/ (accessed on 1 March 2023)), based on a classification algorithm and regression tree [31], enabled **MK** and the reference drug verapamil to be classified as the inhibitors of this protein (Table 2). Concurrently, the well-known P-gp inhibitor and MDR reversal agent verapamil were also classified as P-gp substrates.

**Figure 1:**
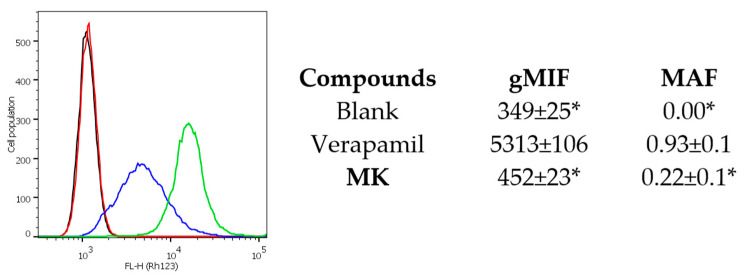
Rh123 efflux from HBL-100/Dox cells. After staining with Rh123 (green line), the cells were incubated for 60 min under the following conditions: blank (grey line), verapamil (30 μM, blue line), and **MK** (2 μM, red line). Asterisks denote significant difference (*p* < 0.05) between verapamil and the untreated/treated cells.

The ability of **MK** to prevent the efflux of typical P-gp substrate Rhodamine 123 (Rh123) was evaluated by the flow cytometric analysis of efflux by means of the dye accumulation technique. The Rh123 test is convenient for characterizing the P-gp inhibitory potential of various agents interacting with the Rh123-binding site of P-gp (so-called “R-site”) [32]. As shown in Figure 1, the efflux of Rh123 from HBL-100/Dox cells was active after 60 min of incubation with the tested compound dosed close to the IC_50_ value. The efflux values appeared to be significantly different from those observed with verapamil (*p* < 0.05). The level of MAF value of **MK** (0.22) was distinctly inferior to those of verapamil (0.93), indicating an extremely low P-gp inhibitory activity of the synthesized compound.

**Figure 2:**
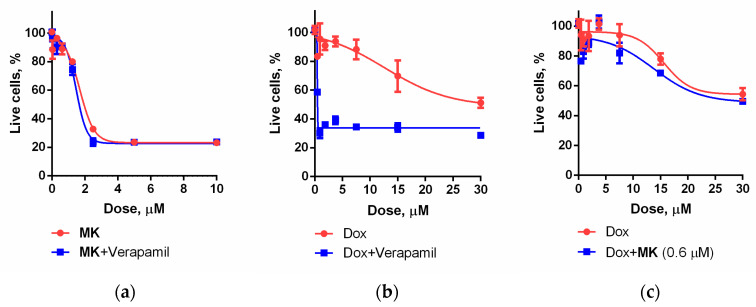
Viability of HBL-100/Dox cells after 72 h exposure to **MK** (**a**), Dox (**b**) taken alone or in the case of blocking the P-gp activity with 30 μM of verapamil, and in the case of Dox in combination with 0.6 µM **MK** (**c**).

**Figure 3:**
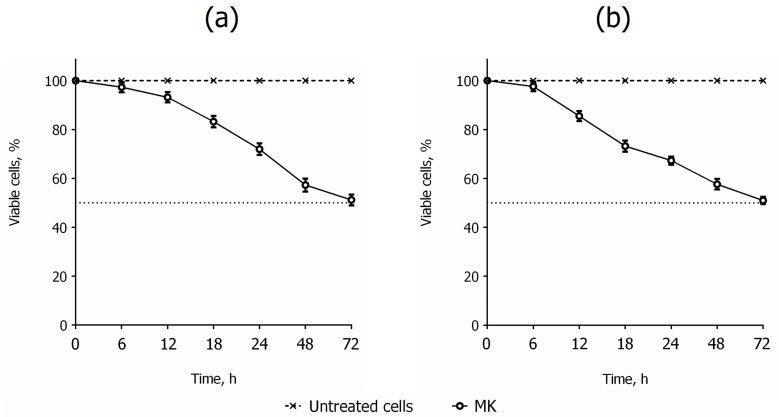
Time-dependent cytotoxicity (0–72 h) of **MK** (IC_50_ values) against (**a**) HBL-100 and (**b**) HBL-100/Dox cells.

To confirm the absence of competitive inhibition, in addition to the efflux test, we performed an experiment aimed at revealing the effects of **MK** on resistant cells in the presence of verapamil. In case of blocking the P-gp activity, the cytotoxic compound, being a substrate of protein, should have had a more pronounced cytotoxic effect. As in the Rh123 efflux test, the effect of **MK** was not dependent on the activity of P-gp in the case when the addition of 30 μM verapamil led to the suppression of P-gp-activity; that is, the number of living cells in the compound, and its combination with 30 μM verapamil, remained unchanged (Figure 2a), whereas the IC_50_ value of Dox against HBL-100/Dox cells had diminished 37-fold (0.8 ± 0.1 μM) with the use of Dox in combination with verapamil (Figure 3b). Thus, despite the encouraging data provided by the prediction services of web-tools (Table 2), both tests in vitro indicated the cytotoxic effect of **MK** against P-gp-overexpressing cells as being in no way related to its interaction with the ABC-transporter P-gp.

While evaluating the effect of the combination of Dox and **MK,** combined at subtoxic concentration (0.6 μM), against resistant cells (Figure 2c), no potentiation in the effectiveness of Dox: 29.6 ± 0.9 µM vs. 30.1 ± 0.5 µM (taken alone vs. in combination, *p* > 0.05) was observed. Thus, any other interaction between the triterpenoid **MK** and the chemotherapeutic drug differing from P-gp inhibition was also excluded.

Then, **MK** was investigated more thoroughly against HBL-100 and HBL-100/Dox cells to prove a common mechanism of cytotoxic action. At first, we analyzed the changes in HBL-100 (Figure 3a) and HBL-100/Dox (Figure 3b) cell proliferation after being exposed to **MK** in a time-course manner (6, 12, 18, 24, 48, 72 h) by MTT assay. Exposure to **MK** at doses corresponding to IC_50_ values induced a time-dependent reduction in cell proliferation in comparison to the untreated cells. As apparent from Figure 3, the cell viability curves began to descend after 12 h incubation. After 18 h treatment, the most significant drop in HBL-100 and HBL-100/Dox viability was observed, with a 50% death of the cells attained after 72 h exposure.

**Figure 4:**
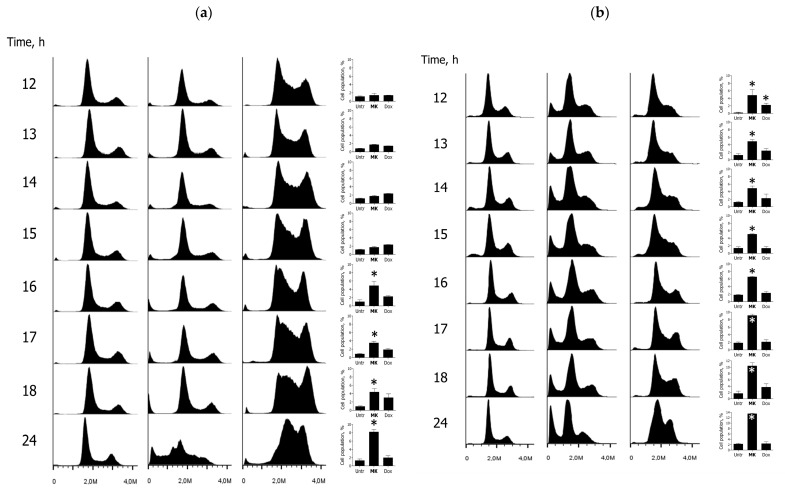
Cell cycle distribution of (**a**) HBL-100 and (**b**) HBL-100/Dox cells treated with **MK** and Dox (at doses corresponding to IC_50_ values) for 12–24 h. Asterisks indicate significant difference (*p* < 0.05) for sub-G0/G1 cell fractions compared with the untreated cells.

Since a continuous **MK** treatment for 12–24 h led to a significant decrease in cell viability, the DNA content of HBL-100 and HBL-100/Dox cells in sub-G0/G1 phase, G0/G1 phase, S phase, and G2/M phase was determined using the flow cytometry technique within the same time interval. As presented in Figure 4a, the HBL-100 cell fractions in sub-G0/G1 phase (indicative of cell death) increased from 1.44 ± 0.44 to 8.22 ± 0.59%, with marked differences (*p* < 0.05) recorded after 16 h exposure to **MK**. After 24 h incubation, the proportion of **MK**-treated cells in the G0/G1 phase increased to 66.48 ± 1.33%, while the proportion of cells in the G2/M phase reduced from 16.66 ± 0.15 to 4.43 ± 0.50% in comparison to the untreated cells. As for the HBL-100/Dox cells (Figure 4b), the sub-G0/G1 cell population increased in a time-dependent manner from 4.84 ± 1.48 to 13.47 ± 2.44% (registered in the 12–24 h interval). This is in accord with a conspicuous reduction in the cells in the S phase from 30.69 ± 0.99 to 8.99 ± 0.42% (from 12 to 24 h). In comparison to the untreated HBL-100/Dox cells (40.34 ± 0.57%), an accumulation in the G0/G1 phase of 52.16 ± 1.94% was observed in **MK**-treated cells after 17 h of incubation. After 24 h, along with the accumulation of the **MK**-treated HBL-100/Dox cells in phase G0/G1 (64.14 ± 0.55%), an increase in the G2/M phase (26.87 ± 1.13%), in comparison to the untreated cells (G0/G1 phase: 51.24 ± 1.17%, G2/M phase: 13.19 ± 1.07%), was observed. Therefore, there was no dominant interference with the cell cycle regulation caused by **MK**; the triterpenoid blocked or slowed down the cell cycle progression through the G0/G1 phase for parental and resistant cells (Tables S3 and S4). Meanwhile, Dox induced the S phase arrest of the HBL-100 and HBL-100/Dox cells, starting at 12 h, and without any significant change in the sub-G0/G1 phase.

**Figure 5:**
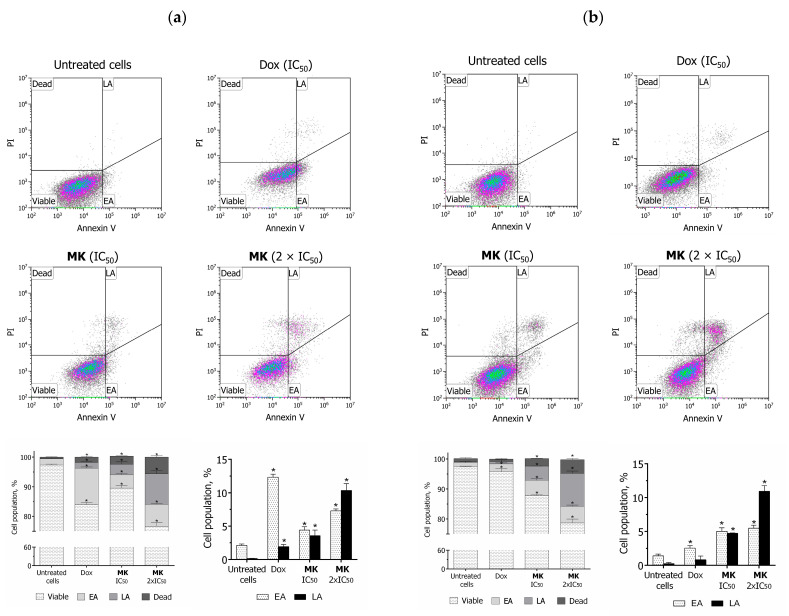
Representative dot plots and summary result histograms of both Annexin V-FITC and PI-stained HBL-100 (**a**) and HBL-100/Dox (**b**) cells. Asterisks denote a significant difference (*p* < 0.05) relative to the untreated control.

The most significant changes in sub-G0/G1 population (*p* < 0.05) for the HBL-100 and HBL-100/Dox cells were recorded after 16 h, following **MK** treatment. With fractional DNA content being a specific marker of cell death most often attributed to the induction of apoptosis in the case of triterpenoids [11,12], we additionally evaluated the dose–response apoptotic **MK** effect in the HBL-100 and HBL-100/Dox cells by Annexin V-FITC detection and propidium iodide (PI) staining using the flow cytometry technique. Annexin V has a strong binding affinity with phosphatidylserine (PS), a membrane phospholipid that moves from the inside of the cell membrane to its outer side during apoptosis, while PI has the ability to bind to DNA and can only penetrate necrotic or late apoptotic cells [33]. Therefore, the proportions of dead (Annexin V−/PI+) to viable (Annexin V−/PI−) cells, as well as early (EA, Annexin V+/PI−) to late (LA, Annexin V+/PI+) apoptotic cells, were measured for quantitative comparison (Table S5). In the experiment, the HBL-100 and HBL-100/Dox cells were treated with different doses of **MK** (IC_50_ and 2 × IC_50_) and Dox (IC_50_) for 16 h, with the subsequent assessment of apoptosis. The flow cytometry plots for all the cells are shown in Figure 5. The quantity of **MK**-treated viable cells decreased in a dose-dependent manner to 89.57 ± 0.93 and 76.73 ± 1.18% of the HBL-100 cells (Figure 5a), and 87.82 ± 0.44 and 78.69 ± 1.29% (HBL-100/Dox cells, Figure 5b) relative to the untreated cells (97.31 ± 0.35 and 97.36 ± 0.25, respectively). The proportion of EA apoptotic HBL-100 cells varied from 4.14 ± 0.17 to 7.29 ± 0.28%, depending on the dose of **MK**. This is in accord with the increase in the LA cell population from 3.59 ± 0.82 to 10.37 ± 1.01%. There were no statistically significant differences in the case of the EA HBL-100/Dox cells (5.04 ± 0.51 vs. 5.76 ± 0.38%, *p* > 0.05) found; by contrast, the proportion of LA cells increased from 4.74 ± 0.44 to 10.94 ± 0.81% after **MK** treatment. These results provided evidence that **MK** provokes a notable increase in cellular apoptosis in a dose-dependent manner. In the case of Dox, the population of EA cells increased to 12.35 ± 0.42 (HBL-100 cells) and 2.56 ± 0.36% (HBL-100/Dox cells) in comparison with the untreated cells (2.12 ± 0.20 and 1.41 ± 0.25%, respectively).

The double-fluorescent staining was employed to further validate the underlying mechanisms of apoptosis. The HBL-100 and HBL-100/Dox cells were stained with Hoechst 33342 and TMRE (tetramethylrhodamine ethyl ester perchlorate) after being treated with 1.0 and 1.8 µM **MK** for 16 h, respectively. Hoechst 33342 is a blue fluorescent dye that binds itself to DNA and it can be used to observe nuclear condensation by fluorescence microscopy. In healthy, untreated cells, nuclei appear round and evenly stained. In apoptotic cells, nuclei are generally fragmented and stained more intensely because of the condensation of DNA [34]. TMRE is a cationic red-orange fluorescent dye used for measuring mitochondrial membrane potential (MMP). The depolarized or inactive mitochondria decrease MMP and fail to sequester TMRE, so that apoptotic cells exhibit a weak fluorescence, while the accumulation of TMRE indicate the liveliness of cells [35].

**Figure 6:**
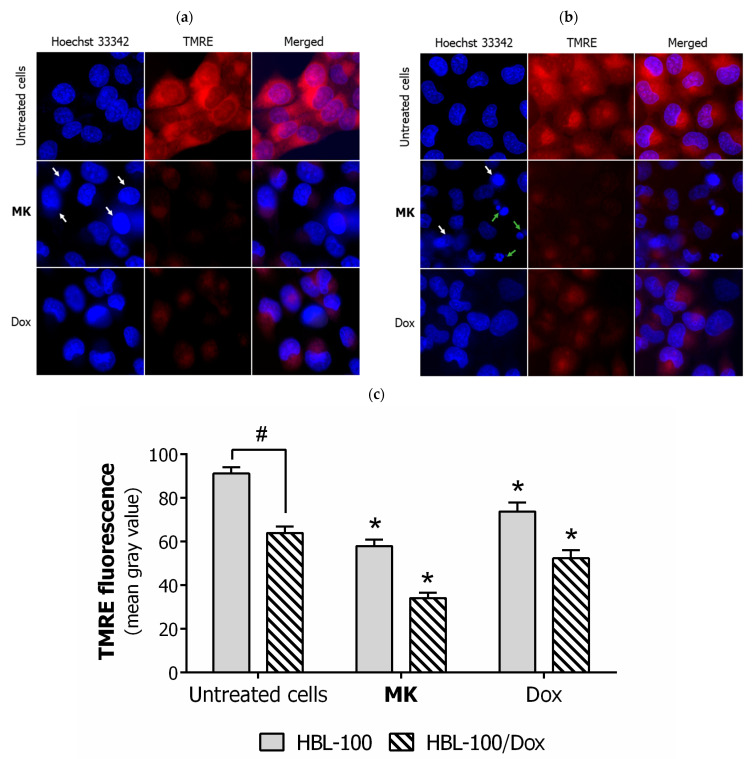
Double-fluorescence of **MK** and Dox-treated HBL-100 (**a**) and HBL-100/Dox (**b**) cells. The cells were stained with Hoechst 33342 and TMRE. Summary graph of TMRE fluorescence intensity (**c**): * significant difference (*p* < 0.05) relative to the untreated control of corresponding cells, and # significant difference (*p* < 0.05) between the untreated groups. Arrows denote typical changes in the nuclear morphology: white—karyopycnosis, green—karyorrhexis.

Our observation showed the untreated HBL-100 and HBL-100/Dox cells to demonstrate weak blue Hoechst 33342 fluorescence whereas, in the groups treated with **MK** and Dox, the apoptotic cells exhibited typical nuclear changes, including bright staining and condensed or fragmented nuclei (Figure 6a,b). Moreover, the number of cells with nuclear morphology changes increased after **MK** treatment to 21.2% (HBL-100 cells) and 33.1% (HBL-100/Dox cells) in comparison with the untreated controls (HBL-100: 3.1%; HBL-100/Dox: 4.1%), while the number of Dox-treated cells was 25.2% (HBL-100 cells) and 9.9% (HBL-100/Dox cells). A significant (*p* < 0.05) reduction in MMP, as indicated by low TMRE fluorescence intensity in the **MK**-treated HBL-100 and HBL-100/Dox cells, when compared with the untreated control (Figure 6c,d), was observed.

The flow cytometry and fluorescent staining data, analyzed comparatively, indicated a more noticeable triterpenoid treatment effect 16 h later, when observed against HBL-100/Dox cells and compared with HBL-100 cells. Thus, an increased nuclear density (or karyopycnosis) was visualized in parental cells (Figure 6a), while the subsequent stage of nuclear fragmentation (or karyorrhexis) was recorded in resistant cells (Figure 6b). Furthermore, in comparison with the untreated cells, a significant increase in the fractional DNA content was recorded much earlier in HBL-100/Dox cells in comparison to the HBL-100 cells treated with **MK** for 12 or 16 h, respectively (Figure 4). This observation is in concordance with the Annexin V-FITC/PI cellular staining after 16 h of **MK** treatment, when the apoptosis-associated externalization of PS and the loss of plasma membrane integrity were more pronounced in the HBL-100/Dox cells in comparison to the HBL-100 cells (Figure 5). Thus, the cells overexpressing P-gp appeared to be significantly more susceptible to the **MK**’s toxic effect.

Mitochondrial metabolic plasticity is known to contribute to the drug resistance of cancer cells [36]. One of the mechanisms of MDR is an increased antioxidant system that protects cells from the increased oxidative stress caused by chemotherapy, which might be attained by reducing mitochondrial biogenesis. For example, sensitive and Dox-resistant osteosarcoma cells have a different mitochondrial profile, in particular, demonstrating a reduced mitochondrial membrane mass, potential, and activity in comparison to Dox-sensitive cells [37]. As apparent from Figure 6c, the HBL-100 and HBL-100/Dox cells also had different basal levels of mitochondrial activity based on TMRE staining, demonstrating a reduction in MMP in native Dox-resistant cells. After **MK** treatment, a noticeable decrease in MMP in the both cell lines, which indicated the participation of mitochondria in **MK**-mediated apoptosis (Figure 6), was observed.

Mitochondrial membrane permeability is a crucial step in the apoptotic pathway cascades. In the mitochondrial apoptotic pathway, the destruction of the outer mitochondrial membrane leads to the release of cytochrome *c* from mitochondria into cytosol. Initiator caspase-9 is activated earlier than effector caspase-3 by binding to a complex containing Apaf-1 and cytochrome *c*. When caspase-9 proteolytically cleaves downstream effector caspase-3, the cleaved caspase-3 transfers to the nucleus and initiates the degradation phase of apoptosis, including DNA fragmentation, by involving the DNA repair enzymes, such as PARP and ICAD [38]. Betulinic acid, and its numerous semi-synthetic derivatives, most often induce apoptosis in cancer cells through the mitochondrial signaling cascade, which involves the expression of caspase-9 and -3 [11].

**Figure 7:**
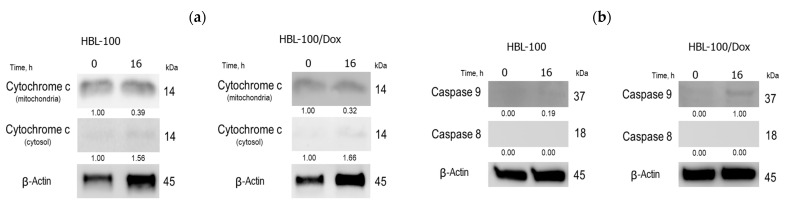
Western blot analysis of the expression of cytochrome *c* (**a**) and the activation of caspase-9 and -8 (**b**) in HBL-100 and HBL-100/Dox cells after **MK** treatment for 16 h. β-Actin was used as loading control.

Indeed, after 16 h of incubation, **MK** provoked the release of cytochrome *c* from mitochondria to cytosol (Figure 7a), as well as the sequential activation of initiator caspase-9 (Figure 7b) in the HBL-100 and HBL-100/Dox cells. This process was significantly more pronounced in resistant cells in comparison with parental cells. Meanwhile, the activation of initiator caspase-8 was not observed in both cell lines, suggesting that this caspase does not participate in **MK**-mediated apoptosis. Thus, **MK** induced the mitochondrial caspase-9-dependent pathway.

We additionally assessed whether the caspase-9 activation leads to the downstream activation of caspase-3, using the flow cytometry technique. We also observed the caspase-3 activation in the HBL-100/Dox cells to be doubly pronounced, in comparison to the HBL-100 cells, after 24 h of **MK** treatment, with the effect being dose-dependent (Figure S5). This was in accordance with a marked increase in fragmented DNA in the HBL-100/Dox cells in same time interval (peak sub-G0/G1, Figure 4).

**Figure 8:**
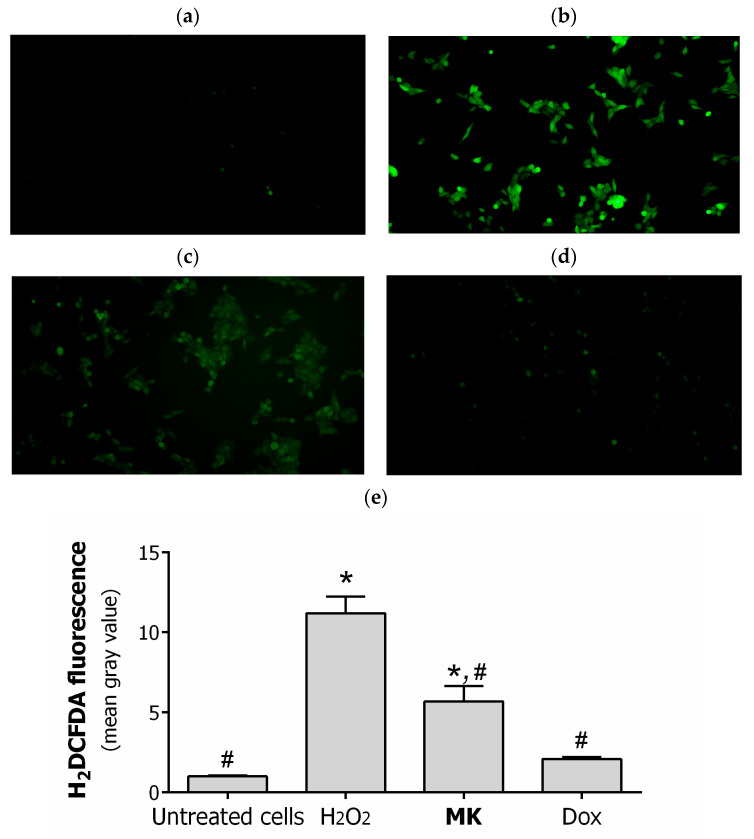
Fluorescent images of ROS in HBL-100/Dox cells. ROS accumulation measured by H_2_DCFDA staining after 4 h of incubation in different conditions: (**a**) untreated cells as negative control, (**b**) H_2_O_2_-treated cells as positive control, (**c**) **MK**-treated cells, and (**d**) Dox-treated cells. Summary graph of H_2_DCFDA fluorescence intensity (**e**): * significant difference (*p* < 0.05) relative to the untreated cells (negative control), and # significant difference (*p* < 0.05) relative to H_2_O_2_-treated cells (positive control).

One of the promising strategies of anticancer drug development is the design and development of mitochondria-targeted compounds [36,39]. Among apoptosis-inducing pentacyclic triterpenoids, the lupane triterpenoids with cation moiety can accumulate in the mitochondrial matrix against their concentration gradient, due to the negative potential of the mitochondrial inner membrane, thereby promoting cytochrome *c* release into the cytoplasm and initiating the mitochondrial pathway of apoptosis by activating the caspase cascade [40,41]. At the same time, hydrophobic betulin and betulinic acid, the parent compounds of **MK**, directly affect the bioenergetics and membrane behavior of mitochondria isolated from the liver of Wistar rats [42]. **MK** may act in a similar way, but we could not exclude the modulation of apoptotic cascade due to oxidative stress by production of an abnormally high level of intracellular reactive oxygen species (ROS), which is also characteristic of triterpenoids [43,44]. Therefore, the ROS status was evaluated in the HBL-100/Dox cells after 4 h of exposure to **MK**, when a decrease in cell viability was not yet observed (Figure 3b), because apoptotic signs were only registered starting at 12 h (Figure 4b). As determined by the H_2_DCFDA fluorescent staining (Figure 8), treatment with **MK** (1.8 μM) significantly heightened the ROS generation by the HBL-100/Dox cells, in contrast to the drug-untreated (negative control) and Dox-treated HBL-100/Dox cells.

The results obtained are in good agreement with the supposition that ROS generation by triterpenoids leads to changes in the inner mitochondrial membrane, causing mitochondrial permeability and damaging its integrity. Consequently, the loss of MMP and the release of proapoptotic proteins into the cytosol, followed by the activation of the caspase cascade [43,44], is present.

## 3. Materials and Methods

### 3.1. Chemistry

All the commercially available reagents were purchased from Merck (Darmstadt, Germany), Sigma–Aldrich Pty Ltd. (an affiliate of Merck KGaA, Darmstadt, Germany, or Acros Organics (Geel, Belgium), and used without further purification. Column chromatography was carried out on a Macherey-Nagel 60 Silica (Düren, Germany) (0.063–0.2 mm). The Sorbfil plates (IMID, Krasnodar, Russia) were employed for thin-layer chromatography (TLC). Melting points were determined on an OptiMelt MPA100 device (Stanford Research Systems, Sunnyvale, CA, USA) at heating rate 1 °C/min. IR spectra (ν, cm^−1^) were recorded on a Bruker IFS 66/S FT-IR spectrometer (Bruker Optik GmbH, Ettlingen, Germany) using a thin film obtained by evaporation from the solution of the substance in CHCl_3_. The 1D spectra (^1^H NMR and ^13^C NMR) and 2D spectra (^1^H-^1^H COSY, ^1^H-^1^H NOESY, ^1^H-^13^C HSQC, ^1^H-^13^C HMBC) were recorded on a Bruker AVANCE II spectrometer (Bruker BioSpin GmbH, Rheinstetten, Germany) at 400 MHz (^1^H NMR) and 100 MHz (^13^C NMR) in CDCl_3_ with hexamethyldisilane as an internal standard. Chemical shifts (δ) and coupling constants (*J*) were expressed in parts per million (ppm) and Hertz (Hz), respectively. Optical rotation was measured on a Perkine Elmer 341 polarimeter (Perkin Elmer, Waltham, MA, USA) using the sodium D line (589 nm) as a light source for CHCl_3_ solutions. Mass spectra (MS) were determined on an Agilent 6890N/5975B chromatograph (Agilent Technologies, Wilmington, NC, USA) equipped with an HP-5 ms UI capillary column (4 m × 0.25 mm, 0.25 µm; 70 eV electron impact). Ozonation reaction was carried out with use of a laboratory ozonizer OGVK-01 (CJSC MELP, St. Petersburg, Russia). Compounds **2**, **3** were prepared according to standard procedures [23,24].

#### 3.1.1. General Procedure for Synthesis of Compounds **4**, **5**

An amount of 5.0 Mmol of H_2_SeO_3_ was added to a solution of 2 mmol **2** (or **3**) in 1,4-dioxane (50 mL). The reaction mixture was refluxed for 5–30 min. The reaction was monitored by TLC. The reaction mixture was diluted with H_2_O (50 mL), and then extracted with ethylacetate (50 mL × 2). The organic layer was separated and dried over anhydrous MgSO_4_. The solvent was evaporated. The residue was purified by column chromatography with SiO_2_. The elution with a mixture of petroleum ether-ethylacetate-chloroform (10:1:1) yielded compounds **4**, **5** as white solids.

28-Benzoyloxy-2-cyano-3,4-seco-3-norlup-4(23)-en-24-al (**4**)

Yield: 53%. M.p. 176.5 °C. ${\left[\alpha \right]}_{D}^{21}$ − 13.7 (*c* 0.5, CHCl_3_). IR (CHCl_3_) cm^−1^: 2954, 2929, 2869, 2245, 1717, 1690, 1602, 1584, 1457, 1387, 1273, 1118, 757, 713 cm^–1^. ^1^H NMR (400 MHz, CDCl_3_) δ (ppm): 0.78 (s, 3H), 0.80 (d, *J* = 6.8 Hz, 3H), 0.86 (d, *J* = 6.8 Hz, 3H), 1.02 (s, 3H), 1.15 (s, 3H), 2.21 (ddd, *J* = 16.6, 6.3, 10.6 Hz, 1H), 2.60–2.68 (m, 2H), 4.04 (d, *J* = 10.6 Hz, 1H), 4.54 (d, *J* = 10.6 Hz, 1H), 6.15 (s, 1H), 6.32 (s, 1H), 7.43 (t, *J* = 7.8 Hz, 2H), 7.54 (t, *J* = 7.4 Hz, 1H), 8.03 (d, *J* = 7.1 Hz, 2H), 9.47 (s, 1H). ^13^C NMR (100 MHz, CDCl_3_) δ (ppm): 195.06, 166.96, 152.18, 137.75, 132.84, 130.58, 129.55 (2C), 128.37 (2C), 120.40, 63.25, 48.16, 46.90, 44.66, 43.50, 40.71, 39.75, 38.99, 38.51, 37.28, 35.35, 34.85, 32.71, 29.99, 29.50, 27.07, 26.40, 24.97, 22.89, 21.71, 21.68, 18.52, 16.15, 14.91, 14.54, 11.39. MS (ESI): m/z (M+) calcd for (C_37_H_51_NO_3_), 557.39 found: 557.3 [M]^+^ (100).

2-Cyano-3,4-seco-3-norlup-4(23)-en-24-al-28-oic acid methyl ester (**5**)

Yield: 51%. M.p. 97.3 °C. ${\left[\alpha \right]}_{D}^{21}$ − 34.7 (*c* 0.6, CHCl_3_) (lit.: Mp 97–99 °C. ${\left[\alpha \right]}_{D}^{21}$−35.0) [25]. IR (CHCl_3_) cm^−1^: 2954, 2929, 2869, 2245, 1717, 1690, 1457, 1273, 1118, 757, 713 cm^–1^. ^1^H NMR (400 MHz, CDCl_3_) δ (ppm): 0.75 (d, *J* = 6.8 Hz, 3H), 0.76 (s, 3H), 0.85 (d, *J* = 6.8 Hz, 3H), 0.98 (s, 6H), 2.15–2.30 (m, 4H), 2.59–2.68 (m, 2H), 3.64 (s, 3H), 6.14 (s, 1H), 6.30 (s, 1H), 9.47 (s, 1H). ^13^C NMR (100 MHz, CDCl_3_) δ (ppm): 195.02, 176.76, 152.19, 137.63, 120.37, 56.97, 51.13, 48.85, 44.19, 43.08, 40.42, 39.97, 38.99, 38.53, 38.04, 37.26, 35.39, 32.84, 31.95, 29.73, 29.63, 26.44, 24.98, 22.92, 22.78, 21.78, 18.53, 16.02, 14.67, 14.44, 11.34. MS (ESI): m/z (M+) calcd for (C_31_H_47_NO_3_), 481.36 found: 481.3 [M]^+^ (100).

#### 3.1.2. General Procedure for Synthesis of Compounds **6**, **7**

An amount of 6 Mmol of NBS was added to a solution of 3 mmol compound **2** (or **3**) in 50 mL of anhydrous CCl_4_. The mixture was boiled under stirring for 2–4 h. The reaction was monitored by TLC. The solvent was distilled under vacuum, and compounds **6**, **7** were purified by column chromatography with SiO_2_ using the mixture of petroleum ether-ethylacetate-chloroform (20:1:1) as eluent.

28-Benzoyloxy-24-bromo-2-cyano-3,4-seco-3-norlup-4(23)-ene (**6**)

Yield: 56%. M.p. 87.9 °C. ${\left[\alpha \right]}_{D}^{21}$ − 11.1 (*c* 0.5, CHCl_3_). IR (CHCl_3_) cm^−1^: 2955, 2928, 2869, 2246, 1716, 1602, 1584, 1453, 1387, 1273, 1118, 758, 712 cm^–1^. ^1^H NMR (400 MHz, CDCl_3_) δ (ppm): 0.80 (d, *J* = 6.6 Hz, 3H), 0.87 (d, *J* = 6.6 Hz, 3H), 0.88 (s, 3H), 1.01 (s, 3H), 1.14 (s, 3H), 2.10 (dd, *J* = 3.0, 12.7 Hz, 1H), 2.25 (dd, *J* = 6.6, 10.9 Hz, 1H), 2.32 (dd, *J* = 4.7, 11.0 Hz, 1H), 3.84 (d, *J* = 10.3 Hz, 1H), 4.00 (d, *J* = 10.3 Hz, 1H), 4.05 (d, *J* = 11.1 Hz, 1H), 4.52 (d, *J* = 11.1 Hz, 1H), 5.03 (s, 1H), 5.39 (s, 1H), 7.43 (t, *J* = 7.8 Hz, 2H), 7.54 (t, *J* = 7.4 Hz, 1H), 8.03 (d, *J* = 7.1 Hz, 2H). ^13^C NMR (100 MHz, CDCl_3_) δ (ppm): 166.91, 146.82, 132.82, 130.56, 129.53 (2C), 128.35 (2C), 119.72, 119.16, 63.20, 48.15, 46.89, 46.63, 44.64, 43.43, 40.67, 40.18, 39.46, 38.86, 37.25, 34.83 (2C), 32.80, 29.98, 29.50, 27.06, 26.47, 26.08, 22.87, 21.69, 21.59, 19.26, 16.10, 14.90, 14.58, 11.68. MS (ESI): m/z (M+) calcd for (C_37_H_52_BrNO_2_), 623.32 found: 541.3 [M −HBr]^+^ (100).

24-Bromo-2-cyano-3,4-seco-3-norlup-4(23)-en-28-oic acid methyl ester (**7**)

Yield: 53%. M.p. 72.7°C. ${\left[\alpha \right]}_{D}^{21}$ − 10.2 (*c* 0.6, CHCl_3_). IR (CHCl_3_) cm^−1^: 2953, 2869, 2246, 1725, 1457, 1378, 1160, 758 cm^–1^. ^1^H NMR (400 MHz, CDCl_3_) δ (ppm): 0.75 (d, *J* = 6.8 Hz, 3H), 0.85 (s, 3H), 0.85 (d, *J* = 6.8 Hz, 3H), 0.96 (s, 3 H), 0.97 (s, 3H), 2.09 (dd, *J* = 2.9, 12.6 Hz, 1H), 2.19–2.33 (m, 5H), 3.64 (s, 3H), 3.84 (d, *J* = 10.4 Hz, 1H), 3.99 (d, *J* = 10.4 Hz, 1H), 5.02 (s, 1 H), 5.38 (s, 1H). ^13^C NMR (100 MHz, CDCl_3_) δ (ppm): 176.74, 146.87, 119.74, 119.13, 56.98, 51.16, 48.87, 46.69, 44.19, 43.04, 40.42, 40.40, 39.49, 38.84, 38.04, 37.26, 34.88, 32.94, 31.97, 29.77, 29.66, 26.55, 26.11, 22.93, 22.78, 21.71, 19.31, 16.01, 14.69, 14.51, 11.66. MS (ESI): m/z (M+) calcd for (C_31_H_48_BrNO_2_), 545.39 found: 545.3 [M]^+^ (100).

#### 3.1.3. General Procedure for Synthesis of Compounds **8**–**13**, **22**, **23**

An amount of 3.0 Mmol of *t*-BuOK was added to a solution of 1 mmol **4** (or **5**, or **6**, or **7**, or **20**, or **21**) in *t*-BuOH (50 mL). The reaction mixture was refluxed for 15–30 min. The course of the reaction was monitored by TLC. The solvent was evaporated under vacuum. The residue was purified by column chromatography with either the mixture of petroleum ether-ethylacetate (10:1) (for compounds **22**, **23**), with that of petroleum ether-ethylacetate-chloroform (10:1:1) (for compounds **8**, **9**, **12**), or with that of petroleum ether-ethylacetate-chloroform (20:1:1) (for compounds **10**, **11**, **13**), as eluent.

2-Cyano-28-hydroxy-3,4-seco-3-nor-2,24-cyclolup-2(24),4(23)-diene (**8**)

Yield: 29%. M.p. 196.1 °C. ${\left[\alpha \right]}_{D}^{21}$ + 130.8 (*c* 0.7, CHCl_3_). IR (CHCl_3_) cm^−1^: 3490, 2954, 2868, 2213, 1626, 1599, 1459, 1386, 1216, 1034, 1021, 911, 757 cm^–1^. ^1^H NMR (400 MHz, CDCl_3_) δ (ppm): 0.65 (s, 3H), 0.77 (d, *J* = 6.8 Hz, 3H), 0.85 (d, *J* = 6.8 Hz, 3H), 0.97 (s, 3H), 1.05 (s, 3H), 1.98 (d, *J* = 17.5 Hz, 1H), 2.27 (d, *J* = 17.5 Hz, 1H), 3.32 (d, *J* = 10.9 Hz, 1H), 3.76 (d, *J* = 10.9 Hz, 1H), 5.16 (s, 1H), 5.17 (s, 1H), 6.82 (d, *J* = 2.5 Hz, 1H). ^13^C NMR (100 MHz, CDCl_3_) δ (ppm): 143.81, 143.44, 120.06, 117.63, 109.56, 60.60, 48.03, 47.95, 47.15, 45.96, 44.62, 43.64, 43.01, 40.58, 36.84, 36.75, 34.04, 31.74, 29.52, 29.28, 26.88, 26.73, 22.92, 21.78, 21.57, 20.10, 15.43, 14.87, 14.50, 13.62. MS (ESI): m/z (M+) calcd for (C_30_H_45_NO), 435.35 found: 433.3 [M –H_2_]^+^ (100).

2-Cyano-24β,28-dihydroxy-3,4-seco-3-nor-2,24-cyclolup-4(23)-ene (**9**)

Yield: 37%. M.p. 170.1 °C. ${\left[\alpha \right]}_{D}^{21}$ + 2.4 (*c* 0.5, CHCl_3_). IR (CHCl_3_) cm^−1^: 3435, 2955, 2928, 2727, 2243, 1460, 1377, 1271, 1026, 759 cm^–1^. ^1^H NMR (400 MHz, CDCl_3_) δ (ppm): 0.65 (s, 3H), 0.77 (d, *J* = 6.9 Hz, 3H), 0.85 (d, *J* = 6.9 Hz, 3H), 0.97 (s, 3H), 1.05 (s, 3H), 2.12 (dd, *J* = 4.4, 13.4 Hz, 1H), 2.58 (ddd, *J* = 13.1, 4.3, 10.6 Hz, 1H), 3.31 (d, *J* = 10.9 Hz, 1H), 3.75 (d, *J* = 10.9 Hz, 1H), 4.12 (br d, *J* = 10.7 Hz, 1H), 4.79 (s, 1H), 5.21 (s, 1H). ^13^C NMR (100 MHz, CDCl_3_) δ (ppm): 148.10, 121.52, 105.97, 73.20, 60.63, 50.02, 48.04, 47.92, 47.34, 44.63, 43.14, 42.40, 41.12, 38.57, 36.78, 35.50, 34.03, 32.36, 29.50, 29.32, 26.84, 26.73, 22.92, 21.76, 21.74, 21.05, 15.84, 14.86, 14.47, 13.66. MS (ESI): m/z (M+) calcd for (C_30_H_47_NO_2_), 453.36 found: 453.3 [M]^+^ (100).

2-Cyano-3,4-seco-3-nor-2,24-cyclolup-2(24),4(23)-dien-28-oic acid methyl ester (**10**)

Yield: 27%. M.p. 76.6 °C. ${\left[\alpha \right]}_{D}^{21}$ + 16.4 (*c* 0.5, CHCl_3_). IR (CHCl_3_) cm^−1^: 2954, 2869, 2211, 1726, 1626, 1599, 1456, 1385, 1162, 758 cm^–1^. ^1^H NMR (400 MHz, CDCl_3_) δ (ppm): 0.64 (s, 3H), 0.75 (d, *J* = 6.8 Hz, 3H), 0.86 (d, *J* = 6.8 Hz, 3H), 0.92 (s, 3H), 0.95 (s, 3H), 1.98 (d, *J* = 17.5 Hz, 1H), 2.28 (d, *J* = 17.5 Hz, 1H), 3.64 (s, 3H), 5.14 (s, 1H), 5.16 (s, 1H), 6.81 (d, *J* = 2.9 Hz, 1H). ^13^C NMR (100 MHz, CDCl_3_) δ (ppm): 176.77, 143.84, 143.40, 120.04, 117.52, 109.60, 57.02, 51.14, 48.87, 47.39, 46.02, 44.22, 43.66, 42.66, 40.30, 38.06, 37.29, 36.79, 32.01, 31.83, 29.76, 29.59, 26.76, 22.94, 21.79, 21.65, 20.09, 15.40, 14.67, 14.45, 13.60. MS (ESI): m/z (M+) calcd for (C_31_H_45_NO_2_), 463.35 found: 463.3 [M]^+^ (100).

*2-Cyano-24β-hydroxy-3,4-seco-3-nor-2,24-cyclolup-4(23)-en-28-oic acid methyl ester (**11**)*

Yield: 69%. M.p. 133.9 °C. ${\left[\alpha \right]}_{D}^{21}$ − 7.6 (*c* 0.6, CHCl_3_). IR (CHCl_3_) cm^−1^: 3461, 2953, 2869, 2242, 1725, 1454, 1385, 1377, 1216, 1163, 758 cm^–1^. ^1^H NMR (400 MHz, CDCl_3_) δ (ppm): 0.64 (s, 3H), 0.75 (d, *J* = 6.8 Hz, 3H), 0.85 (d, *J* = 6.8 Hz, 3H), 0.93 (s, 3H), 0.96 (s, 3H), 2.13 (dd, *J* = 4.5, 13.4 Hz, 1H), 2.58 (ddd, *J* = 13.1, 4.3, 10.7 Hz, 1H), 3.46 (s, 3H), 4.12 (br d, *J* = 10.8 Hz, 1H), 4.78 (s, 1 H), 5.20 (s, 1H). ^13^C NMR (100 MHz, CDCl_3_) δ (ppm): 176.81, 148.14, 121.55, 105.90, 73.17, 56.97, 51.17, 50.07, 48.89, 47.58, 44.23, 42.79, 42.40, 40.84, 38.61, 38.03, 37.30, 35.49, 32.44, 32.07, 29.73, 29.51, 26.75, 22.94, 22.79, 21.82, 21.05, 15.83, 14.83, 14.40, 13.63. MS (ESI): m/z (M+) calcd for (C_31_H_47_NO_3_), 481.36 found: 481.3 [M]^+^ (100).

2-Cyano-28-hydroxy-3,4-seco-3-nor-2,24-cyclolup-4(23)-ene (**12**)

Yield: 53%. M.p. 185.1 °C. ${\left[\alpha \right]}_{D}^{21}$ + 20.9 (*c* 0.5, CHCl_3_). IR (CHCl_3_) cm^−1^: 3468, 2953, 2868, 2239, 1696, 1452, 1386, 1271, 1017, 758, 713 cm^–1^. ^1^H NMR (400 MHz, CDCl_3_) δ (ppm): 0.64 (s, 3H), 0.77 (d, *J* = 6.8 Hz, 3H), 0.84 (d, *J* = 6.8 Hz, 3H), 0.97 (s, 3H), 1.05 (s, 3H), 2.11 (dd, *J* = 3.8, 13.0 Hz, 1H), 2.23 (br t, *J* = 12.8 Hz, 1H), 2.61 (ddd, *J* = 2.0, 4.5, 12.9 Hz, 1H), 2.69 (tt, *J* = 12.8, 4.4 Hz, 1H), 3.30 (d, *J* = 10.8 Hz, 1H), 3.75 (d, *J* = 10.8 Hz, 1H), 4.60 (s, 1H), 4.82 (s, 1H). ^13^C NMR (100 MHz, CDCl_3_) δ (ppm): 145.81, 122.45, 108.87, 60.60, 51.26, 48.03, 47.93, 47.28, 44.62, 43.21, 43.15, 41.15, 39.00, 38.72, 36.73, 34.03, 32.22, 29.48, 29.32, 26.79, 26.76, 25.69, 22.91, 21.75, 21.47, 20.87, 15.90, 14.85, 14.45, 13.54. MS (ESI): m/z (M+) calcd for (C_30_H_47_NO), 437.37 found: 437.2 [M]^+^ (100).

2-Cyano-3,4-seco-3-nor-2,24-cyclolup-4(23)-en-28-oic acid methyl ester (**13**)

Yield: 69%. M.p. 177.7 °C. ${\left[\alpha \right]}_{D}^{21}$ + 9.9 (*c* 0.5, CHCl_3_). IR (CHCl_3_) cm^−1^: 2952, 2869, 2238, 1727, 1454, 1365, 1161, 757 cm^–1^. ^1^H NMR (400 MHz, CDCl_3_) δ (ppm): 0.64 (s, 3H), 0.75 (d, *J* = 6.9 Hz, 3H), 0.86 (d, *J* = 6.9 Hz, 3H), 0.93 (s, 3H), 0.96 (s, 3H), 2.11 (dd, *J* = 4.2, 13.1 Hz, 1H), 2.20–2.29 (m, 5H), 2.60 (ddd, *J* = 1.9, 4.7, 12.9 Hz, 1H), 2.68 (tt, *J* = 12.8, 4.4 Hz, 1H), 3.63 (s, 3H), 4.59 (s, 1H), 4.81 (s, 1H). ^13^C NMR (100 MHz, CDCl_3_) δ (ppm): 176.68, 145.89, 122.31, 108.68, 57.02, 51.44, 50.98, 49.01, 47.66, 44.33, 43.35, 42.88, 40.98, 39.11, 38.79, 38.12, 37.30, 32.40, 32.13, 29.71, 29.52, 26.82, 25.74, 22.85, 22.80, 21.61, 20.89, 15.89, 14.62, 14.41, 13.49. MS (ESI): m/z (M+) calcd for (C_31_H_47_NO_2_), 465.36 found: 465.3 [M]^+^ (100).

2-Cyano-28-hydroxy-3,4-seco-3,23-dinor-2,4-cyclolup-2(4)-ene (**22**)

Yield: 57%. M.p. 184.2 °C. ${\left[\alpha \right]}_{D}^{21}$ + 14.9 (*c* 0.5, CHCl_3_). IR (CHCl_3_) cm^−1^: 3389, 2955, 2868, 2211, 1454, 1385, 1024, 757 cm^–1^. ^1^H NMR (400 MHz, CDCl_3_) δ (ppm): 0.77 (d, *J* = 6.8 Hz, 3H), 0.77 (s, 3H), 0.83 (d, *J* = 6.8 Hz, 3H), 0.98 (s, 3H), 1.03 (s, 3H), 1.92 (dd, *J* = 3.2, 13.0 Hz, 1H), 1.92 (s, 3H), 2.17 (br d, *J* = 13.3 Hz, 1H), 3.30 (d, *J* = 10.7 Hz, 1H), 3.74 (d, *J* = 10.7 Hz, 1H). ^13^C NMR (100 MHz, CDCl_3_) δ (ppm): 164.03, 117.83, 107.02, 60.61, 59.83, 48.77, 48.42, 48.11, 47.91, 46.68, 44.62, 43.25, 41.68, 36.91, 34.17, 34.09, 29.51, 29.41, 27.11, 26.46, 23.55, 22.91, 21.74, 18.72, 17.21, 15.20, 14.84, 14.75, 14.74. MS (ESI): m/z (M+) calcd for (C_29_H_45_NO), 423.35 found: 423.3 [M]^+^ (100).

2-Cyano-3,4-seco-3,23-dinor-2,4-cyclolup-2(4)-en-28-oic acid methyl ester (**23**)

Yield: 48%. M.p. 168.5 °C. ${\left[\alpha \right]}_{D}^{21}$ + 21.3 (*c* 0.5, CHCl_3_). IR (CHCl_3_) cm^−1^: 2954, 2869, 2214, 1727, 1454, 1386, 1164, 757 cm^–1^. ^1^H NMR (400 MHz, CDCl_3_) δ (ppm): 0.74 (d, *J* = 6.9 Hz, 3H), 0.75 (s, 3H), 0.85 (d, *J* = 6.9 Hz, 3H), 0.91 (s, 3H), 0.97 (s, 3H), 1.89 (dd, *J* = 3.2, 13.0 Hz, 1H), 1.92 (s, 3H), 2.07 (br d, *J* = 11.0 Hz, 1H), 2.16–2.26 (m, 4H), 3.64 (s, 3H). ^13^C NMR (100 MHz, CDCl_3_) δ (ppm): 176.74, 164.02, 117.84, 107.03, 59.89, 56.95, 51.14, 49.01, 48.83, 48.44, 46.93, 44.30, 42.94, 41.41, 38.24, 37.38, 34.24, 32.20, 29.76 (2C), 26.48, 23.65, 22.93, 22.79, 18.73, 17.23, 15.17, 14.72, 14.68, 14.65. MS (ESI): m/z (M+) calcd for (C_30_H_45_NO_2_), 451.35 found: 451.3 [M]^+^ (100).

#### 3.1.4. General Procedure for Synthesis of Compounds **14**–**17**, **20**, **21**

Ozone was passed through a solution of 1 mmol compound **2** (or **3**, or **8**, or **10**, or **12**, or **13**) in dicloromethane (50 mL) for 2 h at minus 50 °C. The reaction was monitored by TLC. The solvent was distilled in vacuo. The residue was purified by column chromatography with the mixture of petroleum ether-ethylacetate-chloroform (10:1:1) as eluent.

2-Cyano-28-hydroxy-4-oxo-3,4-seco-3,23-dinor-2,24-cyclolup-2(24)-ene (**14**)

Yield: 25%. M.p. 121.0 °C. ${\left[\alpha \right]}_{D}^{21}$ − 19.8 (*c* 0.6, CHCl_3_). IR (CHCl_3_) cm^−1^: 3494, 2955, 2869, 2224, 1719, 1691, 1459, 1386, 1368, 1277, 1026, 757, 713 cm^–1^. ^1^H NMR (400 MHz, CDCl_3_) δ (ppm): 0.77 (d, *J* = 6.8 Hz, 3H), 0.85 (d, *J* = 6.8 Hz, 3H), 0.85 (s, 3H), 0.97 (s, 3H), 1.03 (s, 3H), 2.29 (br d, *J* = 18.6 Hz, 1H ), 2.54 (d, *J* = 18.6 Hz, 1H), 3.31 (d, *J* = 10.9 Hz, 1H), 3.74 (d, *J* = 10.9 Hz, 1H), 6.48 (d, *J* = 3.0 Hz, 1H). ^13^C NMR (100 MHz, CDCl_3_) δ (ppm): 198.27, 137.96, 127.49, 117.35, 60.55, 56.17, 47.98, 47.91, 47.46, 44.60, 44.16, 43.11, 40.84, 40.57, 36.77, 34.01, 31.08, 29.55, 29.23, 26.81, 26.45, 22.90, 21.76, 21.11, 16.79, 15.46, 14.86, 14.45, 13.96. MS (ESI): m/z (M+) calcd for (C_29_H_43_NO_2_), 437.33 found: 419.2 [M –H_2_O]^+^ (100).

2-Cyano-4-oxo-3,4-seco-3,23-dinor-2,24-cyclolup-2(24)-en-28-oic acid methyl ester (**15**)

Yield: 30%. M.p. 69.7 °C. ${\left[\alpha \right]}_{D}^{21}$ − 26.7 (*c* 0.5, CHCl_3_). IR (CHCl_3_) cm^−1^: 2953, 2869, 2224, 1724, 1691, 1454, 1368, 1145, 757, 585 cm^–1^. ^1^H NMR (400 MHz, CDCl_3_) δ (ppm): 0.75 (d, *J* = 6.8 Hz, 3H), 0.86 (d, *J* = 6.8 Hz, 3H), 0.85 (s, 3H), 0.92 (s, 3H), 0.95 (s, 3H), 2.30 (d, *J* = 18.3 Hz, 1H), 2.54 (d, *J* = 18.3 Hz, 1H), 3.63 (s, 3 H), 6.48 (d, *J* = 3.0 Hz, 1H). ^13^C NMR (100 MHz, CDCl_3_) δ (ppm): 198.34, 176.68, 137.97, 127.56, 117.38, 56.99, 56.26, 51.21, 48.87, 47.74, 44.22 (2C), 42.78, 40.92, 40.33, 37.98, 37.26, 31.97, 31.19, 29.82, 29.55, 26.51, 22.96, 22.80, 21.24, 16.83, 15.48, 14.69, 14.42, 13.97. MS (ESI): m/z (M+) calcd for (C_30_H_43_NO_3_), 465.32 found: 465.3 [M]^+^ (100).

2-Cyano-28-hydroxy-4-oxo-3,4-seco-3,23-dinor-2,24-cyclolupane (**16**)

Yield: 56%. M.p. 166.6 °C. ${\left[\alpha \right]}_{D}^{21}$ − 4.9 (*c* 0.6, CHCl_3_). IR (CHCl_3_) cm^−1^: 3511, 2955, 2869, 2241, 1717, 1459, 1377, 1269, 1034, 757 cm^–1^. ^1^H NMR (400 MHz, CDCl_3_) δ (ppm): 0.73 (s, 3H), 0.78 (d, *J* = 6.8 Hz, 3H), 0.85 (d, *J* = 6.8 Hz, 3H), 0.97 (s, 3H), 1.05 (s, 3H), 2.19 (dd, *J* = 2.7, 11.8 Hz, 1H), 2.29 (dd, *J* = 3.9, 13.4 Hz, 1H), 2.54 (t, *J* = 13.3 Hz, 1H), 2.64 (dd, *J* = 5.1, 13.9 Hz, 1H), 3.03 (tt, *J* = 12.7, 5.1 Hz, 1H), 3.31 (d, *J* = 10.9 Hz, 1H), 3.73 (d, *J* = 10.9 Hz, 1H). ^13^C NMR (100 MHz, CDCl_3_) δ (ppm): 206.92, 120.81, 60.54, 59.03, 47.95, 47.87, 47.25, 44.59, 43.22, 42.28, 42.16, 41.66, 40.87, 36.61, 33.99, 31.04, 29.49, 29.26, 26.73, 26.55, 25.01, 22.89, 21.73, 21.52, 17.03, 15.79, 14.82, 14.38, 14.13. MS (ESI): m/z (M+) calcd for (C_29_H_45_NO_2_), 439.35 found: 439.3 [M]^+^ (100).

2-Cyano-4-oxo-3,4-seco-3,23-dinor-2,24-cyclolup-28-oic acid methyl ester (**17**)

Yield: 66 %. M.p. 220.8 °C. ${\left[\alpha \right]}_{D}^{21}$ − 4.2 (*c* 0.5, CHCl_3_). IR (CHCl_3_) cm^−1^: 2954, 2870, 2239, 1731, 1720, 1704, 1456, 1376, 1159, 756 cm^–1^. ^1^H NMR (400 MHz, CDCl_3_) δ (ppm): 0.73 (s, 3H), 0.76 (d, *J* = 6.9 Hz, 3H), 0.86 (d, *J* = 6.9 Hz, 3H), 0.94 (s, 3H), 0.96 (s, 3H), 2.10 (dd, *J* = 2.6, 11.8 Hz, 1H), 2.22–2.31 (m, 5H), 2.52 (t, *J* = 13.3 Hz, 1H), 2.62 (dd, *J* = 5.2, 13.9 Hz, 1H), 3.02 (tt, *J* = 12.7, 5.1 Hz, 1H), 3.63 (s, 3H). ^13^C NMR (100 MHz, CDCl_3_) δ (ppm): 206.60, 176.57, 120.74, 59.17, 56.96, 51.02, 48.95, 47.65, 44.30, 42.96, 42.33 (2C), 41.71, 40.71, 37.96, 37.25, 32.05, 31.23, 29.74, 29.47, 26.62, 25.02, 22.84, 22.78, 21.69, 17.08, 15.80, 14.61, 14.34, 14.06. MS (ESI): m/z (M+) calcd for (C_30_H_45_NO_3_), 467.34 found: 467.3 [M]^+^ (100).

28-Benzoyloxy-2-cyano-4-oxo-3,4-seco-3,23-dinorlupane (**20**)

Yield: 90%. M.p. 185.8 °C. ${\left[\alpha \right]}_{D}^{21}$ − 0.5 (*c* 0.6, CHCl_3_). IR (CHCl_3_) cm^−1^: 2955, 2869, 2246, 1716, 1602, 1584, 1457, 1273, 1118, 757, 713 cm^–1^. ^1^H NMR (400 MHz, CDCl_3_) δ (ppm): 0.79 (d, *J* = 6.8 Hz, 3H), 0.86 (d, *J* = 6.8 Hz, 3H), 0.97 (s, 3H), 1.00 (s, 3H), 1.16 (s, 3H), 2.12 (s, 3H), 2.24 (dd, *J* = 3.1, 12.7 Hz, 1H), 2.28 (ddd, *J* = 16.6, 6.6, 9.6 Hz, 1H), 2.46 (ddd, *J* = 16.8, 5.9, 9.6 Hz, 1H), 4.04 (d, *J* = 11.1 Hz, 1H), 4.53 (d, *J* = 11.1 Hz, 1H), 7.43 (t, *J* = 7.8 Hz, 2H), 7.54 (t, *J* = 7.4 Hz, 1H), 8.03 (d, *J* = 8.4 Hz, 2H). ^13^C NMR (100 MHz, CDCl_3_) δ (ppm): 211.60, 166.90, 132.83, 130.53, 129.52 (2C), 128.35 (2C), 119.95, 63.16, 56.49, 48.13, 46.87, 44.61, 43.42, 40.65, 40.12, 38.98, 37.21, 34.81, 34.58, 31.83, 30.42, 29.94, 29.46, 27.05, 26.28, 22.85, 21.72, 21.67, 20.95, 19.37, 16.12, 14.88, 14.50, 11.53. MS (ESI): m/z (M+) calcd for (C_36_H_51_NO_3_), 545.39 found: 545.3 [M]^+^ (100).

2-Cyano-4-oxo-3,4-seco-3,23-dinorlupan-28-oic acid methyl ester (**21**)

Yield: 90%. M.p. 177.2 °C. ${\left[\alpha \right]}_{D}^{21}$ − 11.2 (*c* 0.6, CHCl_3_). IR (CHCl_3_) cm^−1^: 2953, 2246, 1725, 1694, 1550, 1458, 1160, 757 cm^–1^. ^1^H NMR (400 MHz, CDCl_3_) δ (ppm): 0.75 (d, *J* = 6.8 Hz, 3H), 0.86 (d, *J* = 6.8 Hz, 3H), 0.95 (s, 3H), 0.96 (s, 3H), 0.99 (s, 3H), 2.11 (s, 3H), 2.21–2.32 (m, 5H), 2.43 (ddd, *J* = 16.5, 6.1, 9.4 Hz, 1H), 3.64 (s, 3H). ^13^C NMR (100 MHz, CDCl_3_) δ (ppm): 211.23, 176.61, 119.79, 56.97, 56.62, 51.02, 48.94, 44.26, 43.09, 40.52, 40.48, 39.06, 38.10, 37.23, 34.75, 32.10, 31.99, 30.35, 29.72, 29.67, 26.37, 22.83, 22.78, 21.83, 21.16, 19.27, 16.01, 14.63, 14.43, 11.51. MS (ESI): m/z (M+) calcd for (C_30_H_47_NO_3_), 469.36 found: 469.3 [M]^+^ (100).

#### 3.1.5. General Procedure for Synthesis of Compounds **18**, **19**

An amount of 0.5 Mmol of compound **16** (or **17**) was dissolved in CH_3_OH (30 mL), then NaBH_4_ (4 mmol) was gradually added, whereupon the magnetically stirred reaction mixture was heated to 60 °C for 30 min. The reaction was monitored by TLC. The solvent was evaporated under vacuum. The residue was purified by column chromatography with the mixture of petroleum ether-ethylacetate-chloroform (7:1:1) as eluent.

2-Cyano-4β,28-dihydroxy-3,4-seco-3,23-dinor-2,24-cyclolupane (**18**)

Yield: 78%. M.p. 205.7 °C. ${\left[\alpha \right]}_{D}^{21}$ − 6.6 (*c* 0.6, CHCl_3_). IR (CHCl_3_) cm^−1^: 3454, 2954, 2868, 2241, 1459, 1376, 1028, 758 cm^–1^. ^1^H NMR (400 MHz, CDCl_3_) δ (ppm): 0.76 (d, *J* = 6.8 Hz, 3H), 0.84 (d, *J* = 6.8 Hz, 3H), 0.95 (s, 3H), 0.99 (s, 3H), 1.07 (s, 3H), 2.05–2.15 (m, 2H), 3.05 (tt, *J* = 12.8, 3.6 Hz, 1H), 3.31 (d, *J* = 10.9 Hz, 1H), 3.75 (d, *J* = 10.9 Hz, 1H), 3.90 (dd, *J* = 2.5, 5.2 Hz, 1H). ^13^C NMR (100 MHz, CDCl_3_) δ (ppm): 123.21, 70.94, 60.58, 49.59, 48.54, 48.05, 47.88, 44.59, 43.18, 43.04, 41.52, 36.91, 36.70, 36.65, 34.01, 33.34, 29.46, 29.32, 26.84, 26.55, 23.00, 22.88, 21.72, 20.47, 20.30, 16.24, 16.04, 14.82, 14.57. MS (ESI): m/z (M+) calcd for (C_29_H_47_NO_2_), 441.36 found: 441.3 [M]^+^ (100).

2-Cyano-4β-hydroxy-3,4-seco-3,23-dinor-2,24-cyclolup-28-oic acid methyl ester (**19**)

Yield: 82%. M.p. 189.7 °C. ${\left[\alpha \right]}_{D}^{21}$ − 9.3 (*c* 0.6, CHCl_3_). IR (CHCl_3_) cm^−1^: 3508, 2952, 2869, 2241, 1725, 1454, 1368, 1218, 1152, 757 cm^–1^. ^1^H NMR (400 MHz, CDCl_3_) δ (ppm): 0.75 (d, *J* = 6.8 Hz, 3H), 0.85 (d, *J* = 6.8 Hz, 3H), 0.94 (s, 3H), 0.95 (s, 3H), 0.98 (s, 3H), 2.06–2.24 (m, 5H), 3.05 (tt, *J* = 12.8, 3.6 Hz, 1H), 3.64 (s, 3H), 3.89 (dd, *J* = 3.0, 5.5 Hz, 1H). ^13^C NMR (100 MHz, CDCl_3_) δ (ppm): 176.79, 123.24, 70.99, 56.97, 51.13, 49.69, 48.94, 48.82, 44.26, 43.06, 42.87, 41.28, 38.01, 37.31, 36.94, 36.72, 33.44, 32.11, 29.72, 29.54, 26.59, 23.04, 22.93, 22.79, 20.48, 20.41, 16.26, 16.07, 14.65, 14.54. MS (ESI): m/z (M+) calcd for (C_30_H_47_NO_3_), 469.36 found: 469.3 [M]^+^ (100).

#### 3.1.6. General Procedure for Synthesis of Compounds **28**–**30**

An amount of 2 Mmol of C_5_H_5_Br_3_N was added to a solution of 2 mmol of compound **20** (or **21**) in glacial acetic acid (10 mL). The reaction mixture was stirred at room temperature for 24 h. The reaction was monitored by TLC. The reaction mixture was washed with H_2_O (30 mL) and extracted with ethyl acetate (20 mL × 2). The organic layer was separated and washed with NaHCO_3_ and then dried over anhydrous MgSO_4_. The solvent was distilled under vacuum; the products were purified by column chromatography with the mixture of petroleum ether-ethylacetate-chloroform (20:1:1) as eluent. The mixture of compounds **28** (yield 70%) and **29** (yield 10%) was obtained in the reaction with compound **20**. Compound **30** (yield 76%) was obtained in the reaction of compound **21**.

28-Benzoyloxy-24-bromo-2-cyano-4-oxo-3,4-seco-3,23-dinorlupane (**28**)

Yield: 70%. M.p. 91.6 °C. ${\left[\alpha \right]}_{D}^{21}$ − 0.7 (*c* 0.6, CHCl_3_). IR (CHCl_3_) cm^−1^: 2955, 2929, 2869, 2246, 1715, 1602, 1584, 1458, 1387, 1273, 1118, 758, 713 cm^–1^. ^1^H NMR (400 MHz, CDCl_3_) δ (ppm): 0.80 (d, *J* = 6.8 Hz, 3H), 0.86 (d, *J* = 6.8 Hz, 3H), 1.01 (s, 3H), 1.03 (s, 3H), 1.16 (s, 3H), 2.28 (ddd, *J* = 16.5, 6.7, 9.5 Hz, 1H), 2.43 (ddd, *J* = 17.0, 6.0, 9.5 Hz, 1H), 2.67 (dd, *J* = 3.0, 12.5 Hz, 1H), 3.86 (d, *J* = 12.8 Hz, 1H), 3.99 (d, *J* = 12.8 Hz, 1H), 4.03 (d, *J* = 11.1 Hz, 1H), 4.53 (d, *J* = 11.1 Hz, 1H), 7.43 (t, *J* = 7.8 Hz, 2H), 7.55 (t, *J* = 7.4 Hz, 1H), 8.03 (d, *J* = 7.0 Hz, 2H). ^13^C NMR (100 MHz, CDCl_3_) δ (ppm): 203.42, 166.91, 132.86, 130.52, 129.54 (2C), 128.37 (2C), 119.71, 63.12, 53.66, 48.14, 46.89, 44.62, 43.44, 40.65, 40.16, 39.16, 37.22, 35.00, 34.86, 34.82, 32.13, 29.94, 29.50, 27.07, 26.25, 22.87, 22.41, 21.69, 21.02, 19.16, 16.05, 14.90, 14.58, 11.79. MS (ESI): m/z (M+) calcd for (C_36_H_50_BrNO_3_), 623.30 found: 545.4 [M − Br]^+^ (100).

28-Benzoyloxy-2-cyano-24,24-dibromo-4-oxo-3,4-seco-3,23-dinorlupane (**29**)

Yield: 10% (with 1 eq. C_5_H_5_NHBr_3_), 80% (with 3 eq. C_5_H_5_NHBr_3_). M.p. 107.7 °C. ${\left[\alpha \right]}_{D}^{21}$ − 15.6 (*c* 0.6, CHCl_3_). IR (CHCl_3_) cm^−1^: 2956, 2928, 2869, 2247, 1716, 1602, 1584, 1454, 1274, 1119, 758, 713 cm^–1^. ^1^H NMR (400 MHz, CDCl_3_) δ (ppm): 0.80 (d, *J* = 6.7 Hz, 3H), 0.87 (d, *J* = 6.7 Hz, 3H), 1.02 (s, 3H), 1.09 (s, 3H), 1.16 (s, 3H), 2.26 (ddd, *J* = 16.6, 6.6, 10.0 Hz, 1H), 2.42 (ddd, *J* = 16.4, 5.7, 10.0 Hz, 1H), 2.83 (dd, *J* = 3.2, 11.9 Hz, 1H), 4.05 (d, *J* = 11.2 Hz, 1H), 4.54 (d, *J* = 11.2 Hz, 1H), 5.91 (s, 1 H), 7.42 (t, *J* = 7.8 Hz, 2H), 7.53 (t, *J* = 7.4 Hz, 1H), 8.01 (d, *J* = 7.9 Hz, 2H). ^13^C NMR (100 MHz, CDCl_3_) δ (ppm): 196.98, 166.82, 132.76, 130.63, 129.51 (2C), 128.31 (2C), 119.32, 63.13, 51.89, 48.24, 46.94, 44.71, 43.50, 42.92, 40.74, 40.24, 39.20, 37.34, 35.45, 34.85, 32.36, 30.01, 29.51, 27.16, 26.27, 24.44, 22.78, 21.71, 21.26, 19.02, 16.03, 14.86, 14.64, 12.17.

24-Bromo-2-cyano-4-oxo-3,4-seco-3,23-dinorlupan-28-oic acid methyl ester (**30**)

Yield: 76%. M.p. 124.4 °C. ${\left[\alpha \right]}_{D}^{21}$ − 12.2 (*c* 0.6, CHCl_3_). IR (CHCl_3_) cm^−1^: 2953, 2870, 2246, 1723, 1457, 1386, 1161, 757 cm^–1^. ^1^H NMR (400 MHz, CDCl_3_) δ (ppm): 0.75 (d, *J* = 6.9 Hz, 3H), 0.85 (d, *J* = 6.9 Hz, 3H), 0.97 (s, 3H), 0.98 (s, 3H), 1.00 (s, 3H), 2.20–2.31 (m, 5H), 2.42 (ddd, *J* = 16.5, 6.2, 9.6 Hz, 1H), 2.67 (dd, *J* = 2.9, 12.5 Hz, 1H), 3.64 (s, 3H), 3.86 (d, *J* = 12.8 Hz, 1H), 3.98 (d, *J* = 12.8 Hz, 1H). ^13^C NMR (100 MHz, CDCl_3_) δ (ppm): 203.28, 176.57, 119.54, 56.95, 53.76, 51.04, 48.94, 44.25, 43.11, 40.55, 40.47, 39.23, 38.09, 37.22, 35.04, 34.75, 32.35, 31.96, 29.74, 29.67, 26.33, 22.83, 22.78, 22.52, 21.21, 19.08, 15.94, 14.64, 14.48, 11.75. MS (ESI): m/z (M+) calcd for (C_30_H_46_BrNO_3_), 547.27 found: 545.4 [M −H_2_]^+^ (100).

#### 3.1.7. General Procedure for Synthesis of Compounds **29**, **31**, **32**

A solution of 2 mmol of compound **20** (or **21**) and 6 mmol of C_5_H_5_Br_3_N in acetic acid (10 mL) was refluxed for 2 h. The reaction mixture was cooled, washed with H_2_O (30 mL), and extracted with ethyl acetate (20 mL × 2). The organic layer was separated, washed with NaHCO_3_, and dried over anhydrous MgSO_4_. The solvent was distilled under vacuum; the products were purified by column chromatography with the mixture of petroleum ether-ethylacetate-chloroform (20:1:1) as eluent. Compound **29** (yield 80%) was obtained in the reaction with compound **20**. The mixture of compounds **31** (yield 60%) and **32** (yield 15%) was obtained in the reaction of compound **21**.

2-Cyano-24,24-dibromo-4-oxo-3,4-seco-3,23-dinorlupan-28-oic acid methyl ester (**31**).

Yield: 60%. M.p. 147.1 °C. ${\left[\alpha \right]}_{D}^{21}$ − 25.1 (*c* 0.5, CHCl_3_). IR (CHCl_3_) cm^−1^: 2954, 2870, 2247, 1726, 1457, 1384, 1160, 757 cm^–1^. ^1^H NMR (400 MHz, CDCl_3_) δ (ppm): 0.78 (d, *J* = 6.8 Hz, 3H), 0.89 (d, *J* = 6.8 Hz, 3H), 1.00 (s, 3H), 1.02 (s, 3H), 1.10 (s, 3H), 2.23–2.35 (m, 4H), 2.42 (ddd, *J* = 16.4, 5.4, 10.5 Hz, 1H), 2.85 (dd, *J* = 3.3, 11.8 Hz, 1H), 3.67 (s, 3 H), 5.94 (s, 1H). ^13^C NMR (100 MHz, CDCl_3_) δ (ppm): 197.09, 176.64, 119.48, 56.91, 51.76, 51.20, 48.84, 44.15, 44.13, 43.04, 40.38, 40.28, 39.15, 38.00, 37.22, 35.46, 32.42, 31.90, 29.77, 29.65, 26.32, 24.50, 22.91, 22.77, 21.31, 19.21, 15.92, 14.69, 14.54, 12.17. MS (ESI): m/z (M+) calcd for (C_30_H_45_Br_2_NO_3_), 627.17 found: 547.2 [M −Br]^+^ (100).

2-Cyano-4-oxo-24,24,24-tribromo-3,23-dinorlupan-28-oic acid methyl ester (**32**).

Yield: 15%. M.p. 80.3 °C. ${\left[\alpha \right]}_{D}^{21}$ − 34.0 (*c* 0.5, CHCl_3_). IR (CHCl_3_) cm^−1^: 2954, 2930, 2869, 2247, 1726, 1456, 1380, 1160, 758 cm^–1^. ^1^H NMR (400 MHz, CDCl_3_) δ (ppm): 0.79 (d, *J* = 6.8 Hz, 3H), 0.90 (d, *J* = 6.8 Hz, 3H), 1.01 (s, 3H), 1.06 (s, 3H), 1.20 (s, 3H), 2.15–2.38 (m, 6H), 2.49 (ddd, *J* = 16.2, 4.1, 11.8 Hz, 1H), 3.34 (dd, *J* = 2.5, 11.7 Hz, 1H), 3.68 (s, 3H). ^13^C NMR (100 MHz, CDCl_3_) δ (ppm): 191.13, 176.63, 119.36, 56.92, 51.22, 48.84, 47.99, 47.10, 44.15, 43.04, 40.44 (2C), 39.65, 38.06, 37.22, 36.07, 32.52, 31.89, 29.79, 29.67, 27.14, 26.42, 22.91, 22.76, 21.57, 19.33, 16.00, 14.69, 14.59, 12.85. MS (ESI): m/z (M+) calcd for (C_30_H_44_Br_3_NO_3_), 705.09 found: 547.2 [M –Br_3_]^+^ (100).

### 3.2. Biology

#### 3.2.1. Cell Lines and Culture Conditions

The MCF-7 (breast adenocarcinoma), HCT116 (colon carcinoma), RD TE32 (rhabdomyosarcoma), MS (melamona), A549 (non-small cell lung carcinoma), PC-3 (prostate adenocarcinoma), HEpG2 (hepatocellular carcinoma), HEK293 (non-cancerous embryonic kidney cells), HBL-100 (mammary epithelial cells immortalized by SV-40 virus and turned tumorigenic owing to protracted cultivation [26]), and P-gp overexpressed HBL-100/Dox cells were delivered from the N.N. Blokhin National Medical Research Center of Oncology (the Ministry of Health of the Russian Federation, Moscow). The HBL-100/Dox cells were derived from the HBL-100 cells after continuous cell selection in the presence of doxorubicin (Dox; TEVA, Tel-Aviv, Israel) [18]. The cells were maintained in DMEM (MCF-7, HCT116, HEpG2, HEK293) or RPMI-1640 (RD TE32, MS, A549, PC-3, HBL-100, HBL-100/Dox) medium (PanEco, Moscow, Russia) with 10% fetal bovine serum (FBS; Biosera, Cholet, France), 2 mM L-glutamine (PanEco, Moscow, Russia), and 1% Penicillin (50 U/mL)-Streptomycin (50 µg/mL) solution (PanEco, Moscow, Russia). The cells were cultured in a humidified CO_2_ incubator (model 460-CE; Thermo Fisher Scientific, Waltham, MA, USA) at +37 °C and 5% CO_2_.

#### 3.2.2. MTT Cell Viability Assay

To investigate the cytotoxic activity of the compounds under study, the MTT colorimetric assay [45] was performed. Briefly, 1 × 10^4^ cells suspended in 100 μL of growth medium were seeded in 96-well plates (SPL Life Sciences, Pocheon-si, Republic of Korea), and incubated for 24 h at +37 °C and in 5% CO_2_. All the compounds under study, dissolved in DMSO (PanEco, Moscow, Russia) to concentration 1 × 10^−2^ M in advance and further diluted in growth medium, were added to the cells at concentrations ranging from 100 to 0.3125 μM. The DMSO-treated cells (with 1% DMSO, no effect on cell growth) were used as a control, while Dox was used as a reference drug. After 72 h, 20 μL of 5 mg/mL solution of MTT (PanEco, Moscow, Russia), in sterile phosphate-buffered saline (PBS; PanEco, Moscow, Russia), was added to each well and further incubated for 4 h. Then, the medium with the compounds was removed, and the formed formazan crystals were dissolved in 100 μL of DMSO. The optical density of the content of each of the 96-well plates at 544 nm was measured using a microplate reader FLUOstar Optima (BMG Labtech, Ortenberg, Germany). The IC_50_ values were determined from dose-dependent curves, using Prism 6.0 (GraphPad Software, Boston, MA, USA).

The time-dependent viability of the HBL-100 and HBL-100/Dox cells exposed to **MK** was evaluated by MTT assay, as described above. The cells were treated with **MK** (IC_50_ values) for 6, 12, 18, 24, 48, and 72 h. The percentage of the viable cells was calculated according to the following equation: cell viability (%) = 100 × (mean absorbance in the treatment group/mean absorbance in the control group). The results were expressed pictorially using Prism 6.0 (GraphPad Software, Boston, MA, USA).

#### 3.2.3. In Silico P-gp Substrate and P-gp Inhibitor Prediction

Three online services were used to predict whether triterpenoid **MK** can behave as a substrate or an inhibitor of P-gp. ADMETlab 2.0 (https://admetmesh.scbdd.com/ (accessed on 1 March 2023)), an extended version of the widely used ADMETlab, was used to predict the ADMET profiles for chemicals [46]. For the regression endpoints, the concrete predictive values are represented as different symbols: 0–0.1 (−−−), 0.1–0.3 (−−), 0.3–0.5 (−), 0.5–0.7 (+), 0.7–0.9 (++), and 0.9–1.0 (+++).

The approach, called pkCSM, also provides a platform for predicting and optimizing pharmacokinetic and toxicity properties and relies on distance-based graph signatures. The pkCSM is provided online at http://biosig.unimelb.edu.au/pkcsm/ (accessed on 1 March 2023) [47].

PgpRules is a server providing two separate prediction services, one for P-gp substrate and another one for P-gp inhibitor, with classification and regression trees (CART) algorithm [48]. PgpRules is freely accessible at https://pgprules.cmdm.tw/ (accessed on 1 March 2023).

#### 3.2.4. Evaluation of P-gp Functional Activity

The functional activity of P-gp was evaluated using the previously described method [49]. The HBL-100/Dox cells were detached with accutase (HiMedia Laboratories, Maharashtra, India). For efflux assay, the cells were first loaded for 20 min with a cold culture medium containing 5.0 μg/mL of Rhodamine 123 (Rh123; Sigma–Aldrich, St. Louis, MO, USA). After incubation, the cells were washed twice and divided into several fractions (2.5 × 10^5^ cells per point). One fraction was incubated in a pure medium, and the other one was incubated with **MK** (2.0 μM) added. The well-known P-gp inhibitor verapamil (30.0 μM; Alfa Aesar, Ward Hill, MA, USA) served as a reference control. Incubation was carried out in culture medium RPMI-1640 without FBS at 37 °C for 60 min. Cell fluorescence was evaluated on CytoFlex S flow cytometer (Beckman Coulter, Brea, CA, USA). The results were analyzed using FlowJo software (ver. X 10.0.7r2, FlowJo Software, San Jose, CA, USA) and were represented as geometric mean fluorescence intensity (gMFI) and MAF coefficient [50], which were calculated using the following formula: MAF = (Mean (inh.) − Mean (free))/Mean (inh.), where MAF is the MDR activity functional, mean (free) is the mean value of cell fluorescence without inhibitor, and mean (inh.) is the mean value for cell fluorescence with inhibitor.

#### 3.2.5. Combined Action of Compounds

All the combinations of drugs were tested against the HBL-100/Dox cells for 72 h by MTT assay as described above. To run the combined test with verapamil, serial dilutions of the first drug (**MK** or Dox) were prepared directly in a 96-well plate (SPL Life Sciences, Pocheon-si, Republic of Korea), with 30.0 μM of verapamil (Alfa Aesar, Ward Hill, MA, USA) simultaneously added to each well for blocking P-gp activity. To run the combined test with Dox, Dox was present in the range of 30.0–5.0 μM, with 0.6 μM of **MK** simultaneously added into each well of the 96-well plate. The results were expressed pictorially using Prism 6.0 (GraphPad Software, Boston, MA, USA).

#### 3.2.6. Cell Cycle Analysis

To analyze the cell cycle of HBL-100 and HBL-100/Dox cells, cell concentration was adjusted to 1 × 10^5^ cells/well and cultured in 6-well plates for 24 h, then treated with **MK** or Dox (at IC_50_ values) for 12, 13, 14, 15, 16, 17, 18, and 24 h. The cells were trypsinized and rinsed with the growth medium. Then, the cells were harvested by centrifugation at 500× *g* for 3 min, and the supernatant was discarded. The cells were washed with PBS (PanEco, Moscow, Russia) twice and centrifuged under similar conditions. The cells were resuspended in 500 μL of PBS, put into centrifuge tubes and fixed by adding 4.5 mL of 70% ethanol dropwise (on ice). The samples were fixed at 4 °C. Overnight, the fixed cells were centrifugally collected (1000× *g*, 4 °C, 5 min) and washed again with PBS. The cells were finally resuspended in DAPI with 0.1% Triton X-100 in PBS solution (10 μg/mL, Sigma–Aldrich, St. Louis, MO, USA), and stained for 30 min at room temperature in darkness. For each sample, at least 50,000 cells were analyzed using the CytoFlex S flow cytometer (Beckman Coulter, Brea, CA, USA). Cell cycle populations were quantified from DNA histograms with the FlowJo software (ver. X 10.0.7r2, FlowJo Software, San Jose, CA, USA).

#### 3.2.7. Annexin V-FITC/PI Assay

The apoptosis rate of the HBL-100 and HBL-100/Dox cells was detected using a FITC Annexin V Apoptosis Detection Kit I (BD Biosciences, Franklin Lakes, NJ, USA) according to the manufacturer’s instructions. Briefly, after exposing the cells to the tested and control compounds for 16 h, the cells were detached with accutase (HiMedia Laboratories, Maharashtra, India) and washed with cold PBS. Then, the cells were re-suspended in 100 μL of 1 × Binding Buffer at concentration 1 × 10^6^ cells/mL and incubated with 5 μL Annexin V-FITC and 5 μL propidium iodide (PI) for 15 min at room temperature in darkness. After incubation, 400 μL of 1 × Binding Buffer was added to each tube. The samples were analyzed by flow cytometry (CytoFlex S, Beckman Coulter, Brea, CA, USA) within 1 h of staining, and the findings were analyzed using a trial version of the Kaluza software (Beckman Coulter, Brea, CA, USA).

#### 3.2.8. Fluorescent Microscopy

The HBL-100 and HBL-100/Dox cells were seeded onto confocal dishes with glass bottoms (PanEco, Moscow, Russia) at a concentration of 1 × 10^5^ cells/well, and incubated for 24 h at 37 °C under culture conditions. Then, the cells were treated with **MK** and Dox at their IC_50_ values for 16 h (Hoechst 33342 and TMRE staining). For H_2_DCFDA labeling, the HBL-100/Dox cells were pretreated with **MK** (1.8 µM), Dox (30.0 µM), and H_2_O_2_ (25.0 µM, positive control) for 4 h. The DNA marker, termed Hoechst 33342 Ready Flow Reagent, was used as a stock solution (Invitrogen, ThermoFisher Scientific, USA). The mitochondrial marker, LumiTracker Mito TMRE (tetramethylrhodamine, ethyl ester; Lumiprobe, Moscow, Russia), was dissolved in DMSO as 1.0 mM stock solution. The marker of reactive oxygen species, H_2_DCFDA (Lumiprobe, Moscow, Russia), was dissolved in DMSO as 100.0 μM stock solution. After incubation, the cells were incubated with FBS-free culture medium containing the dye mixture of Hoechst 33342 (final concentration 1.0 μM) and LumiTracker Mito TMRE (final concentration 1.0 μM), or H_2_DCFDA dye taken alone (final concentration 1.0 μM), for 30 min at 37 °C in darkness. Fluorescence, at 420 nm (Hoechst 33342), at 510 nm (TMRE), and at 575 nm (H_2_DCFDA), of the test cultures (including controls) was analyzed immediately using a fluorescence microscope CKX 53 (Olympus, Tokyo, Japan). The TMRE fluorescence intensity was quantified using Fiji (ImageJ) [51].

#### 3.2.9. Western Blotting

The HBL-100 and HBL-100/Dox cells were cultured in a 6-well plate with a density of 2 × 10^6^ cells/well. After treatment with IC_50_ concentrations of **MK** for 16 h, all of the detached/dead and viable cells were harvested and lysed in Mitochondria Isolation Kit for Cultured Cells (Thermo Fisher Scientific, Waltham, MA, USA). The cytosol and mitohondrial fractions were separated by centrifugation at 12,000× *g* for 15 min at 4 °C. The supernatant (cytosol fraction) and mitochondrial pellets were then collected, and protein concentration was determined by UV fluorescence at 260 nm using 1–100 μg/mL BSA as standard curve concentrations (UV-2600i spectrophotometer; Shimadzu, Tokyo, Japan).

Then, the same protein amounts (20.0 µg in each lane) were loaded and separated by 4–20% Mini-PROTEAN TGX Stain-Free Protein Gels (Bio-Rad Laboratories, Hercules, CA, USA), followed by transferring onto polyvinylidene difluoride (PVDF) membranes. The membranes were blocked with 1% (*w/v*) casein in Tris-buffered saline containing 0.1% Tween-20 (TBS-T), and were incubated overnight with primary antibodies (1:1000) of Cytochrome *c* (D18C7) Rabbit mAb, Cleaved Caspase-9 (Asp330) (E5Z7N) Rabbit mAb, Cleaved Caspase-8 (Asp374) (18C8) Rabbit mAb, and β-Actin (D6A8) Rabbit mAb (Cell Signaling Technology, Danvers, MA, USA) for cytosol and mitohondrial fractions at 4 °C. The next day, the PVDF membranes were washed thrice in TBS-T and incubated with HRP-conjugated secondary antibodies (1:1000) (Cell Signaling Technology, Danvers, MA, USA) for 1 h at RT. Immunoreactive proteins were detected by Clarity Western ECL Substrate (Bio-Rad Laboratories, Hercules, CA, USA) and then analyzed on the ChemiDoc MP Imaging system (Bio-Rad Laboratories, Hercules, CA, USA). The proteins were quantified by densitometry using Image Lab software (version 6.0; Bio-Rad Laboratories, Hercules, CA, USA). To normalize protein expression data, a blot with β-Actin was used.

#### 3.2.10. Flow Cytometric Analysis of Active Caspase-3

Briefly, the HBL-100 and HBL-100/Dox cells (1 × 10^6^ cells per well) were seeded into a 6-well plate (SPL Life Sciences, Pocheon-si, Republic of Korea). After 24 h, the cells were treated with **MK** (at IC_50_ and 2 × IC_50_ concentrations) for another 24 h period. The cells were detached with accutase (HiMedia Laboratories, Maharashtra, India) and then fixed with 70% ice cold ethanol followed by overnight incubation at 4 °C. The cells were washed twice with PBS, permeabilized using 0.1% Triton X-100 in PBS solution, and subsequently stained with 5 μL of the BD Horizon™ BV605 Rabbit Anti-Active Caspase-3 antibody (BD Biosciences, Franklin Lakes, NJ, USA). The cells were then washed, resuspended in PBS, and analyzed using a CytoFlex S flow cytometer (Beckman Coulter, Brea, CA, USA). Cell fluorescence was evaluated on a CytoFlex S flow cytometer (Beckman Coulter, Brea, CA, USA). The results were analyzed using FlowJo software (ver. X 10.0.7r2, FlowJo Software, San Jose, CA, USA) and represented as geometric mean fluorescence intensity (gMFI).

#### 3.2.11. Statistics

All the experiments were performed in triplicate. The results were presented as mean ± SD values. The differences were considered statistically significant at *p* < 0.05. For statistical analysis and graph design, GraphPad Prism 6.0 was used. The statistical significance was evaluated by the unpaired *t*-test and Mann–Whitney test.

## 4. Conclusions

Overall, new dihydrobetulin derivatives, including A-seco-derivatives and “triterpenoid-steroid” hybrids, were synthesized. Screening studies of cytotoxic activity revealed 24,24-dibromo-substituted methyl ketone **31** (**MK**) as being the most active anticancer agent, the activity of which was also preserved against the P-gp-overexpressing cancer cells. The studies of interaction between **MK** and P-gp have shown that **MK** is not a substrate or an inhibitor of the ABC-transporter P-gp. Regardless of the P-gp status of cancer cells, **MK** causes apoptotic cell death by increasing ROS production, thereby triggering the depolarization of the inner mitochondrial membrane potential, the intrinsic apoptotic pathway with cytochrome *c* release, and the activation of caspases-9 and -3.

## Figures and Tables

**Table 1:** The results of the MTT assay presented as IC_50_ values obtained after 72 h of treatment ^1^.

Cell Lines	IC_50_ Values (Mean ± SD), µM					
	12	14	19	30	31 (MK)	Dox
HEK293	76.5 ± 2.5	77.8 ± 2.9	34.6 ± 1.1	24.2 ± 0.5	11.5 ± 0.6	0.4 ± 0.1
MCF-7	64.2 ± 3.0	56.7 ± 3.7	47.9 ± 0.3	12.0 ± 0.1	7.1 ± 0.7	0.4 ± 0.1
HCT116	51.0 ± 3.7	49.9 ± 0.1	28.2 ± 2.1	13.5 ± 0.3	3.9 ± 0.1	1.3 ± 0.3
RD TE32	98.6 ± 2.3	49.1 ± 1.4	25.2 ± 0.1	7.1 ± 0.9	0.7 ± 0.1	1.3 ± 0.1
MS	>200	69.1 ± 1.0	22.2 ± 2.0	45.9 ± 2.2	16.5 ± 1.5	1.3 ± 0.2
A549	44.4 ± 2.4	63.2 ± 1.4	25.9 ± 1.8	27.7 ± 0.3	7.0 ± 0.2	2.0 ± 0.2
PC-3	55.7 ± 4.9	28.3 ± 2.3	17.2 ± 0.7	34.0 ± 1.3	10.4 ± 1.3	12.6 ± 0.2
HEpG2	>100	>100	31.8 ± 2.7	49.6 ± 0.5	16.6 ± 0.4	1.8 ± 0.3
HBL-100	36.6 ± 1.4	46.9 ± 2.2	16.0 ± 0.9	3.6 ± 0.3	1.0 ± 0.1	0.4 ± 0.1
HBL-100/Dox	63.0 ± 1.9	54.0 ± 1.8	21.1 ± 2.2	4.5 ± 0.2	1.8 ± 0.1	29.6 ± 0.9

^1^ The complete cytotoxic activity data for derivatives **4**–**23**, **28**–**31** are given in Supplementary Materials (Table S1).

**Table 2:** Comparative in silico prediction of P-gp substrates and inhibitors.

Compounds	Web-Tools	Substrate	Inhibitor	MlogP
**MK**	ADMETlab 2.0 ^1^	−−−	+++	5.98
	pkCSM	−	+	8.07
	PgpRules	−	+	8.07
**Verapamil**	ADMETlab 2.0 ^1^	+++	+++	3.33
	pkCSM	+	+	5.09
	PgpRules	+	+	3.13

^1^ The predictive values for ADMETlab 2.0: 0–0.1 (−−−), 0.3–0.5 (−), 0.5–0.7 (+) and 0.9–1.0 (+++).

## Data Availability

Not applicable.

## Supplementary Materials

The following supporting information can be downloaded at: https://www.mdpi.com/article/10.3390/ijms24129863/s1.
