# Supplementary material for: Synthesis and Anticancer Activity of A-Ring-Modified Derivatives of Dihydrobetulin
Source: Int J Mol Sci. 2023 Jun 7;24(12):9863. doi: 10.3390/ijms24129863 (PMC10298530; doi:10.3390/ijms24129863)
Supplement: Supplementary file 1 [file ijms-24-09863-s001.zip › ijms-2437401-supplementary.pdf]

Supplementary Materials

Synthesis and anticancer activity of A-Ring Modified Derivatives of Dihydrobetulin

Irina Tolmacheva, Yulia Beloglazova, Mikhail Nazarov, Olga Gagarskikh, Victoria Grishko*

erm Federal Scientific Centre, Institute of Technical Chemistry UB RAS, Academician Korolev St. 3, 614013 Perm, Russia

* Correspondence: grishvic@gmail.com

Determination of the relative configuration of C(2), C(24) and C(4) atoms of compounds **9**, **11**, **18** and **19** was carried out using the data of 2D NMR spectra (HMBC, HSQC, NOESY, COZY) of compounds **11** and **19** (Fig. 1, 2). Cross peaks in the HMBC spectra of compound **11** C(1)/H(2), C(1)/H_3(25), C(2)/H(24), C(3)/H(1), C(3)/H(2), C(3)/H(24), C(4)/H(5), C(4)/H(23), C(4)/H(24), C(24)/H_2(23) were the most informative. The NOESY correlation between the H(2) and H_3(25) protons, as well as between the H(5) and H(24) protons showed that the product is the 2α-cyano-24β-hydroxy derivative **11** (Figure S1).

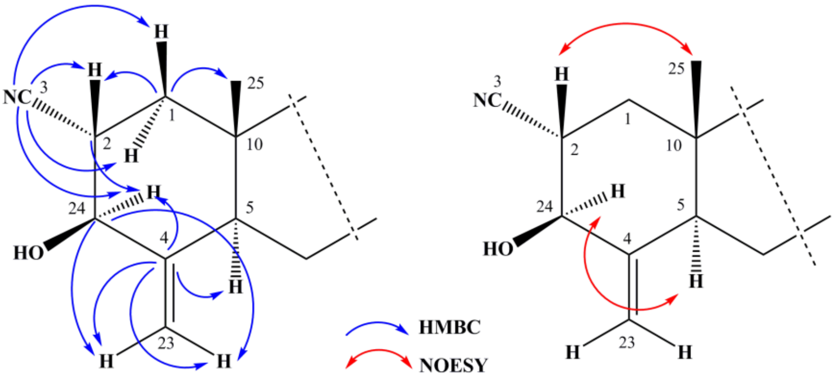

Figure S1. Key HMBC and NOESY correlations for compound **11**.

NOESY correlations of compound **19** H(2)/H_3(25) and H(4)/H(5) showed that the product is 2α-cyano-4β-hydroxy derivative **19** (Figure S2).

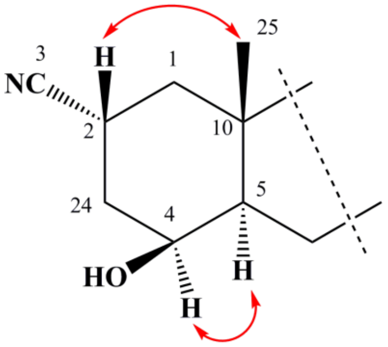

Figure S2. Key NOESY correlations for compound **19**.

1. ^{1}H, ^{13}C NMR spectra.

^{1}H NMR spectra of 28-Benzoyloxy-2-cyano-3,4-seco-3-norlup-4(23)-en-24-al (4).

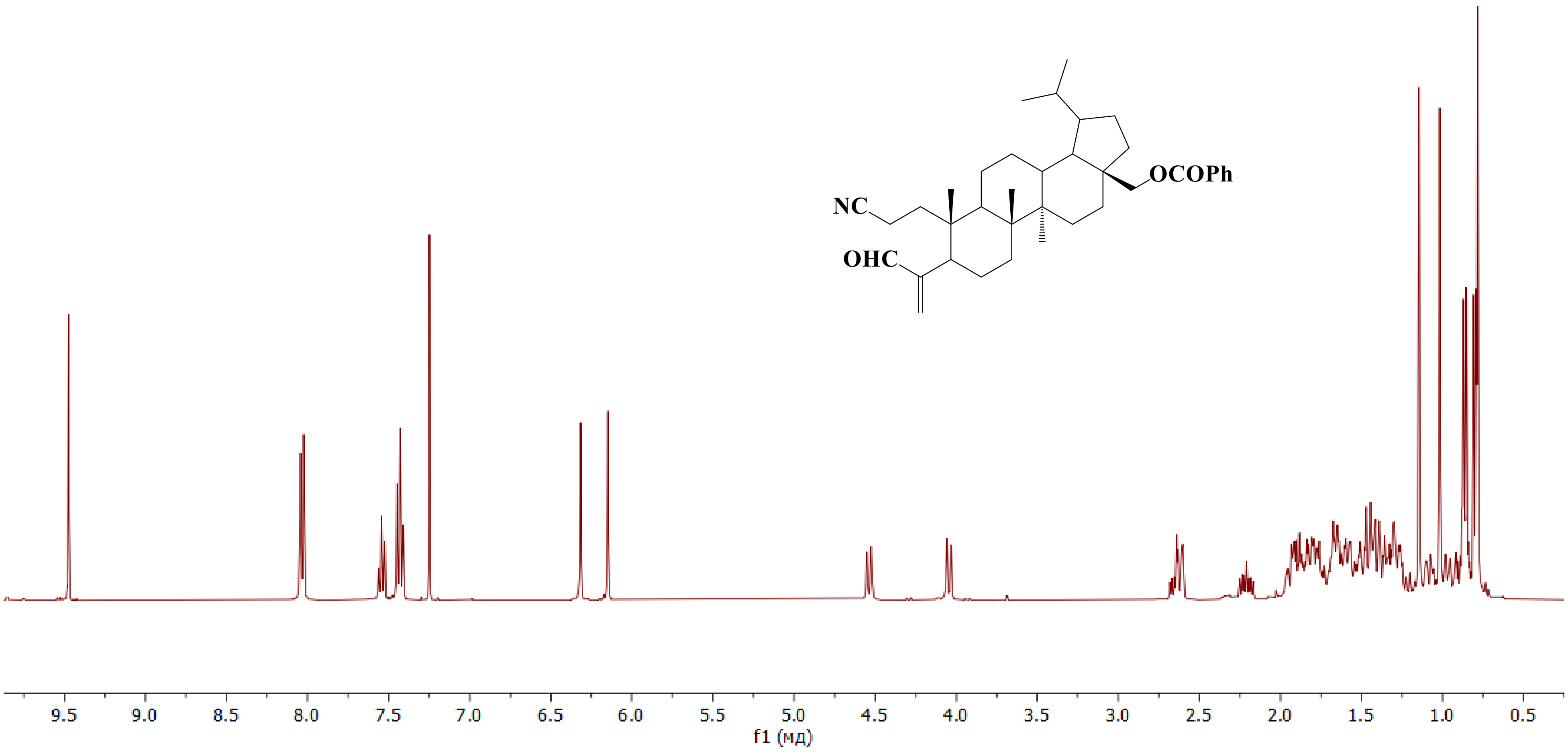

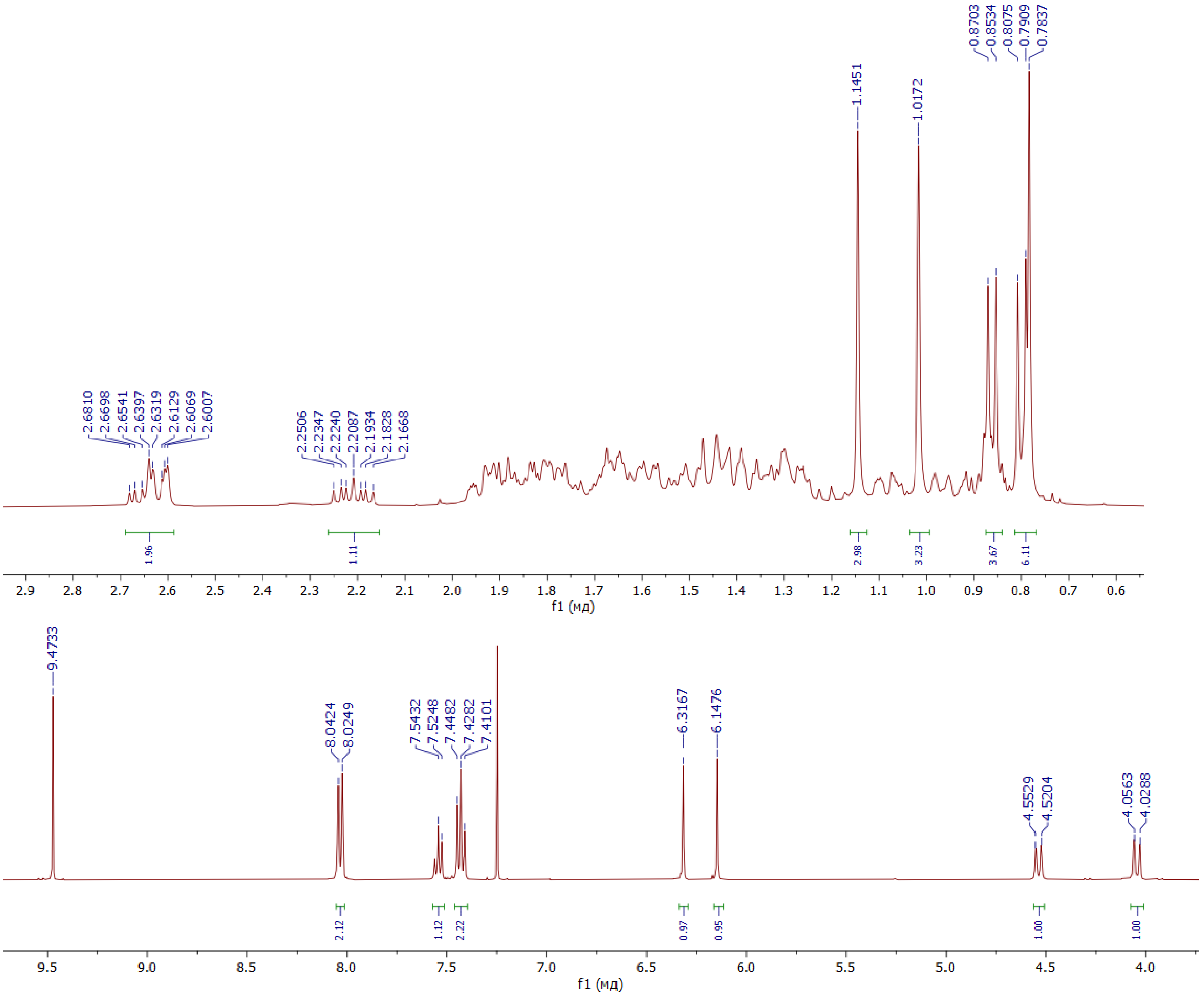
2.6810
2.6698
2.6541
2.6397
2.6319
2.6129
2.6069
2.6007
2.2506
2.2347
2.2240
2.2087
2.1934
2.1828
2.1668
1.1451
1.0172
0.8703
0.8534
0.8075
0.7909
0.7837
1.96
1.11
2.98
3.23
3.67
6.11
f1 (мд)
9.4733
8.0424
8.0249
7.5432
7.5248
7.4482
7.4282
7.4101
6.3167
6.1476
4.5529
4.5204
4.0563
4.0288
2.12
1.12
2.22
0.97
0.95
1.00
1.00
f1 (мд)

^{13}C NMR spectra of 28-Benzoyloxy-2-cyano-3,4-seco-3-norlup-4(23)-en-24-al (4).

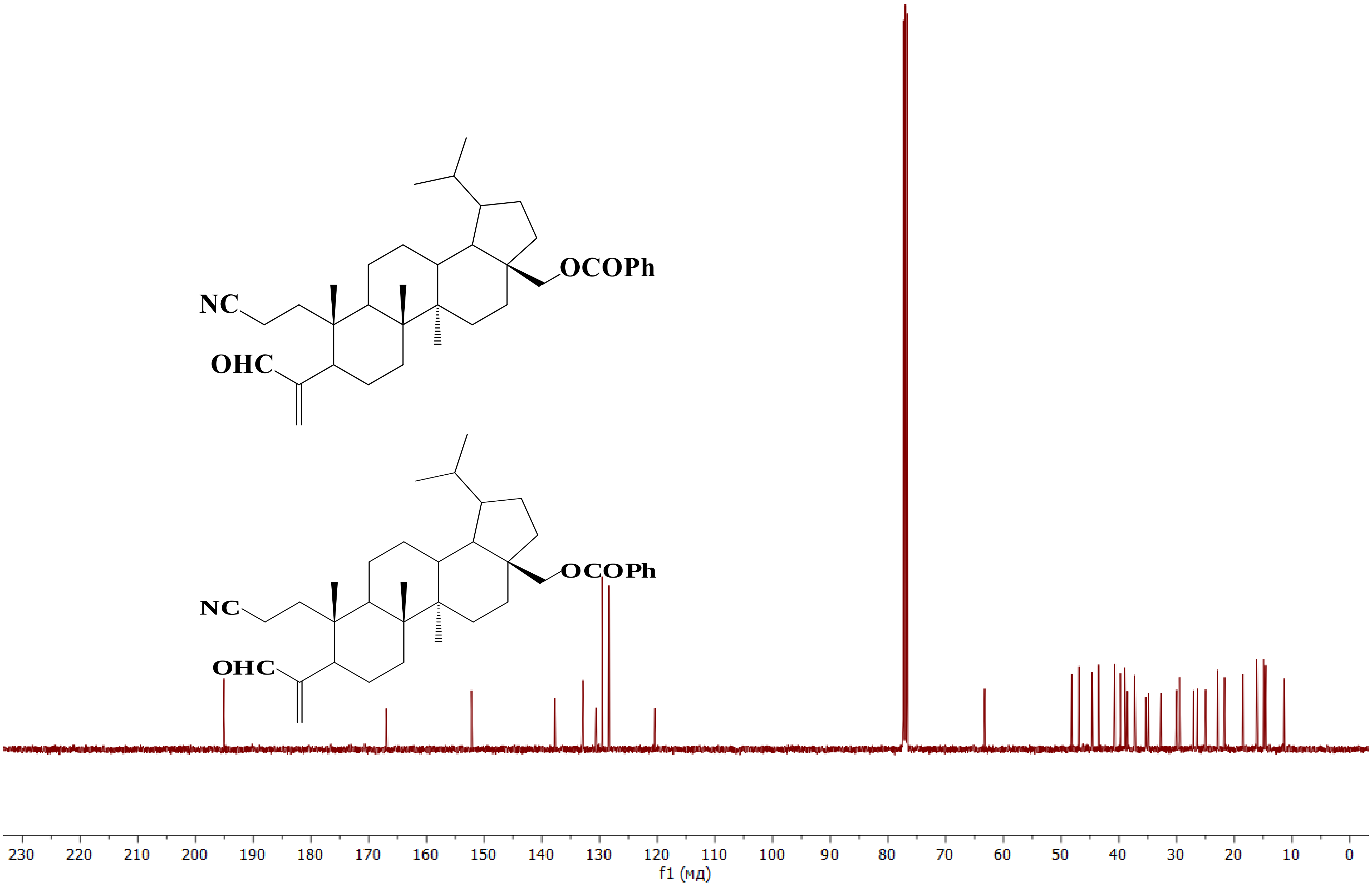

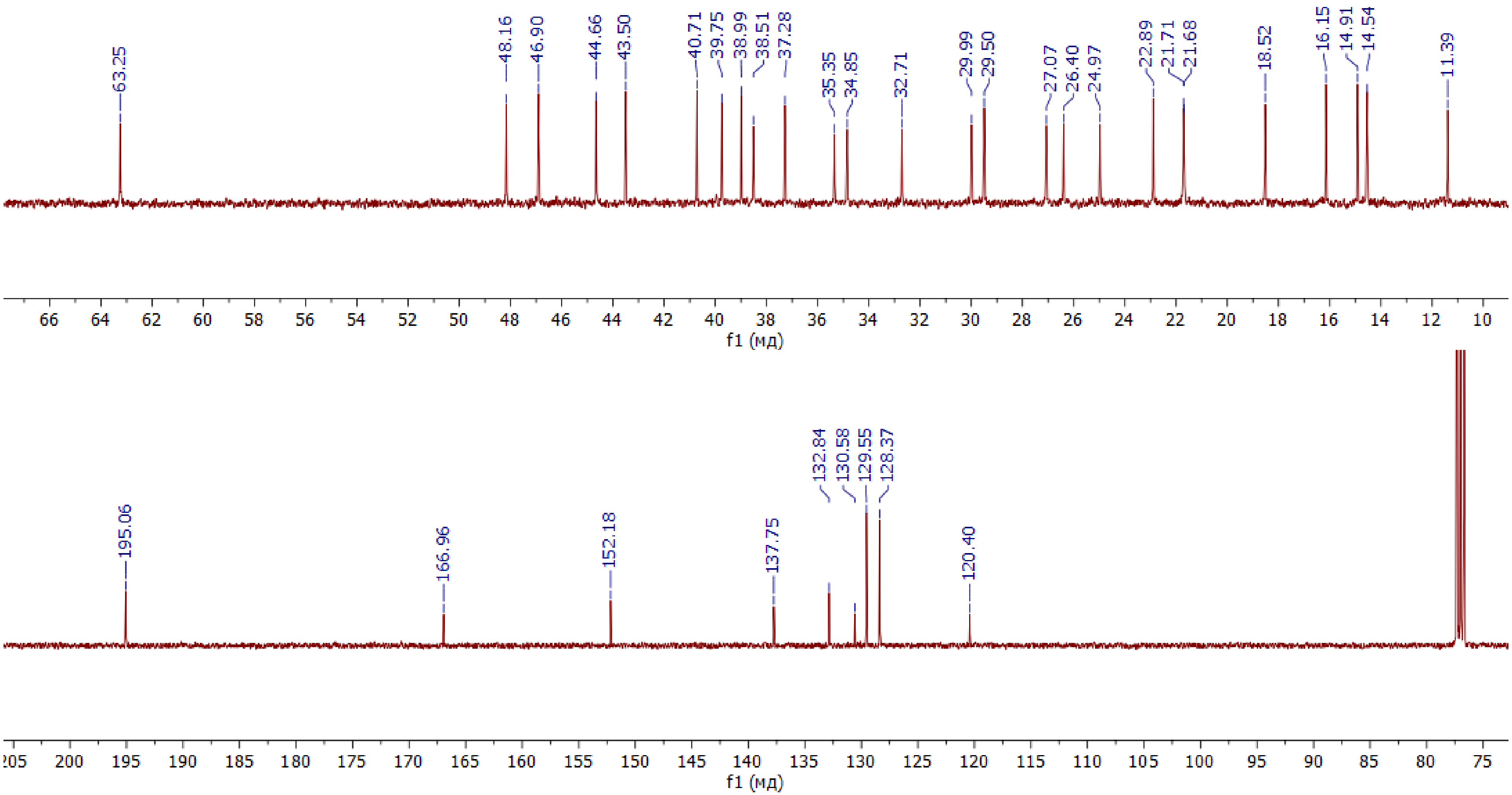

63.25
48.16
46.90
44.66
43.50
40.71
39.75
38.99
38.51
37.28
35.35
34.85
32.71
29.99
29.50
27.07
26.40
24.97
22.89
21.71
21.68
18.52
16.15
14.91
14.54
11.39
f1 (мд)
195.06
166.96
152.18
137.75
132.84
130.58
129.55
128.37
120.40
f1 (мд)

^{1}H NMR spectra of 2-Cyano-3,4-seco-3-norlup-4(23)-en-24-al-28-oic acid methyl ester (5).

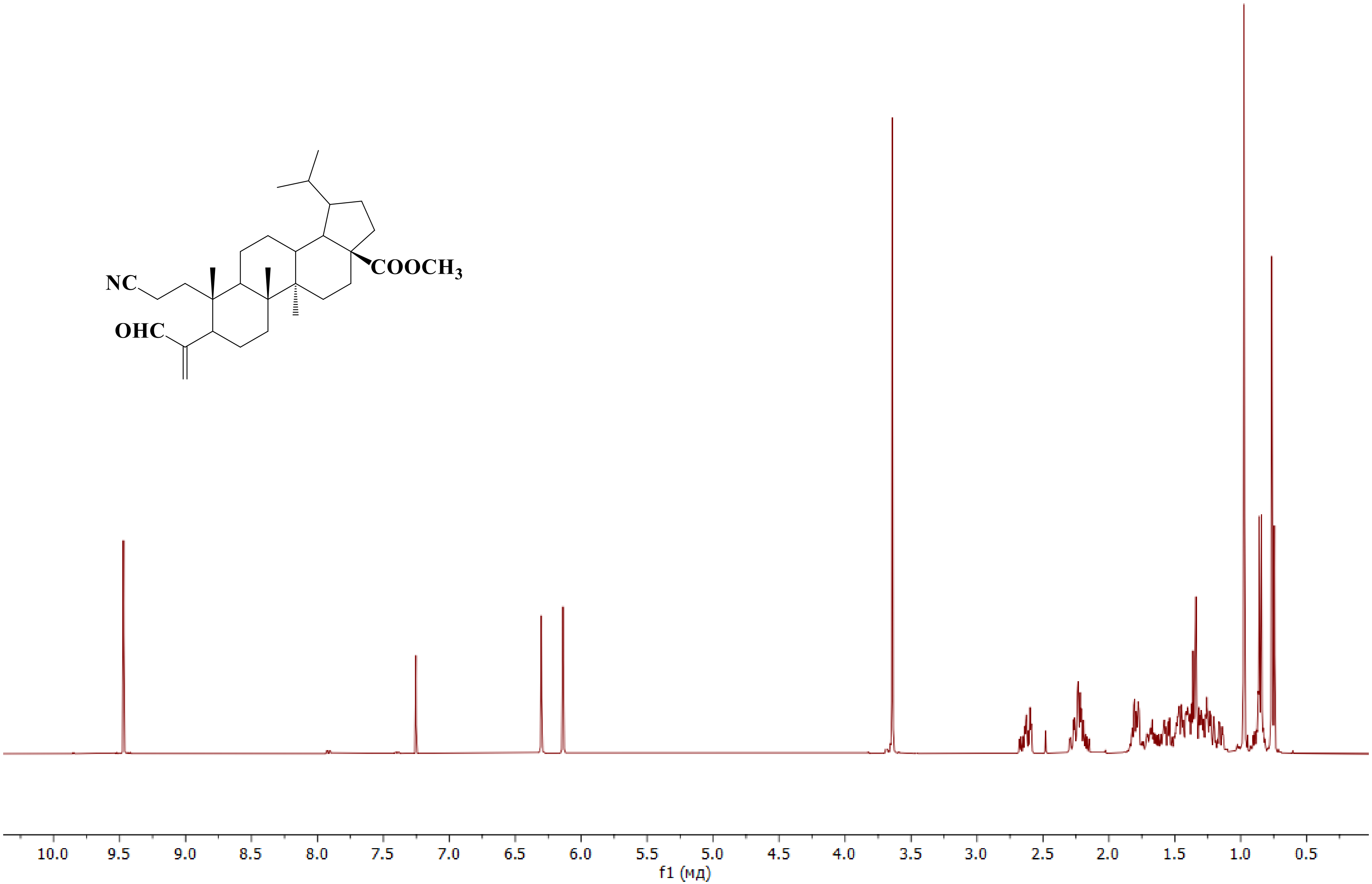

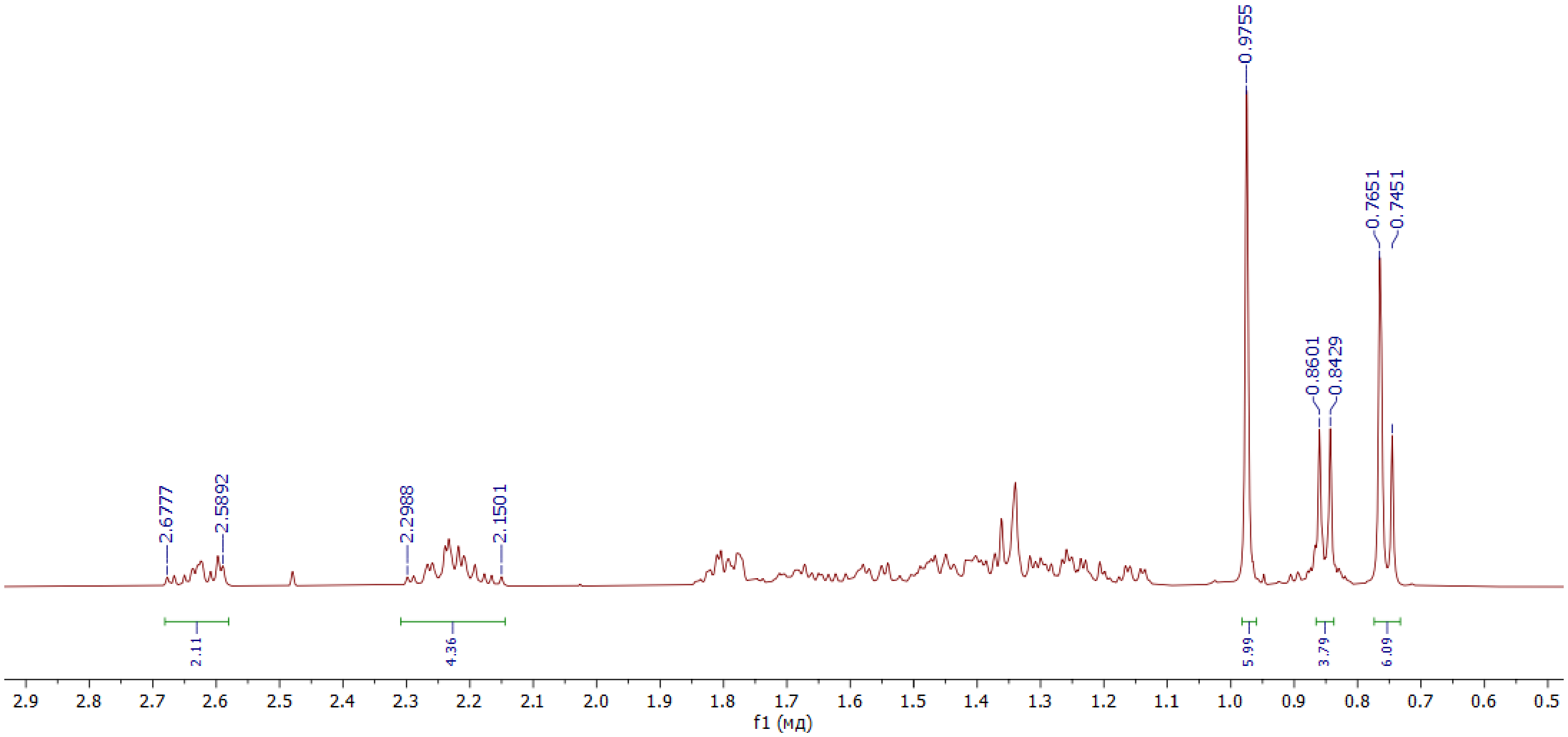
2.6777
2.5892
2.2988
2.1501
0.9755
0.8601
0.8429
0.7651
0.7451
2.11
4.36
5.99
3.79
6.09
2.9 2.8 2.7 2.6 2.5 2.4 2.3 2.2 2.1 2.0 1.9 1.8 1.7 1.6 1.5 1.4 1.3 1.2 1.1 1.0 0.9 0.8 0.7 0.6 0.5
f1 (мд)

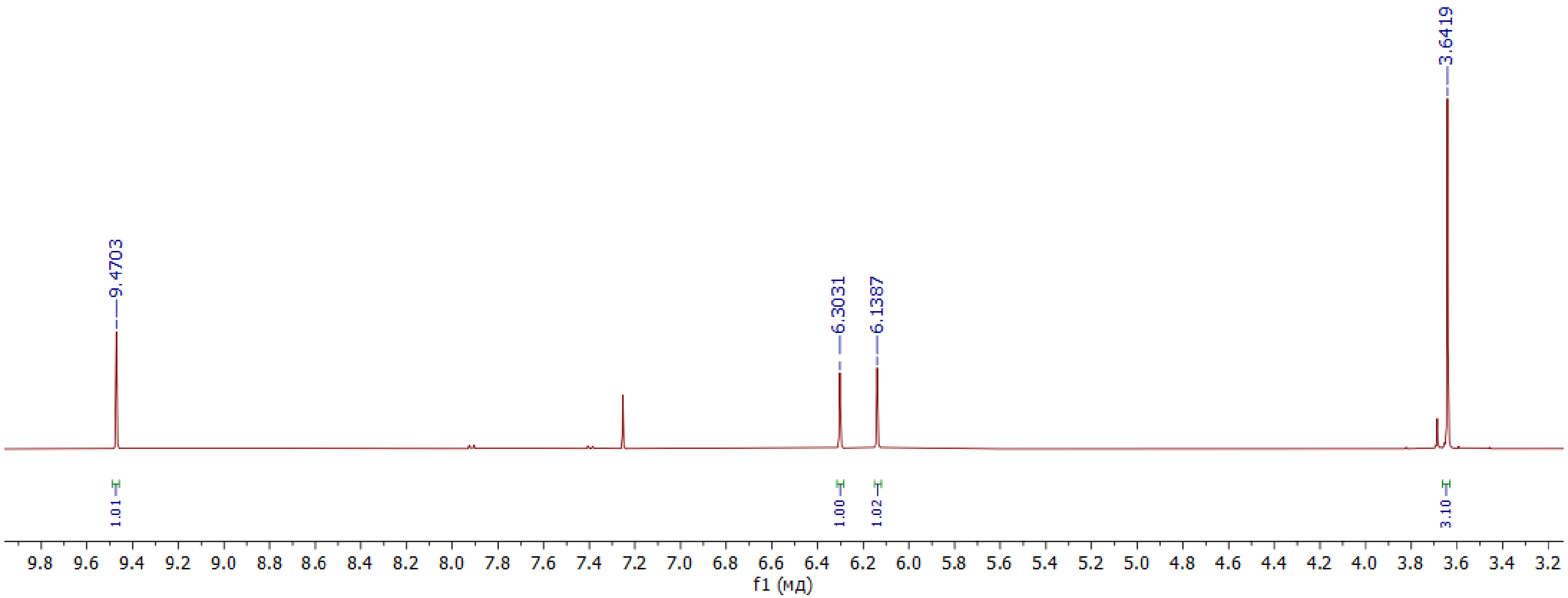
9.4703
6.3031
6.1387
3.6419
1.01
1.00
1.02
3.10
9.8 9.6 9.4 9.2 9.0 8.8 8.6 8.4 8.2 8.0 7.8 7.6 7.4 7.2 7.0 6.8 6.6 6.4 6.2 6.0 5.8 5.6 5.4 5.2 5.0 4.8 4.6 4.4 4.2 4.0 3.8 3.6 3.4 3.2
f1 (мд)

^{13}C NMR spectra of 2-Cyano-3,4-seco-3-norlup-4(23)-en-24-al-28-oic acid methyl ester (5).

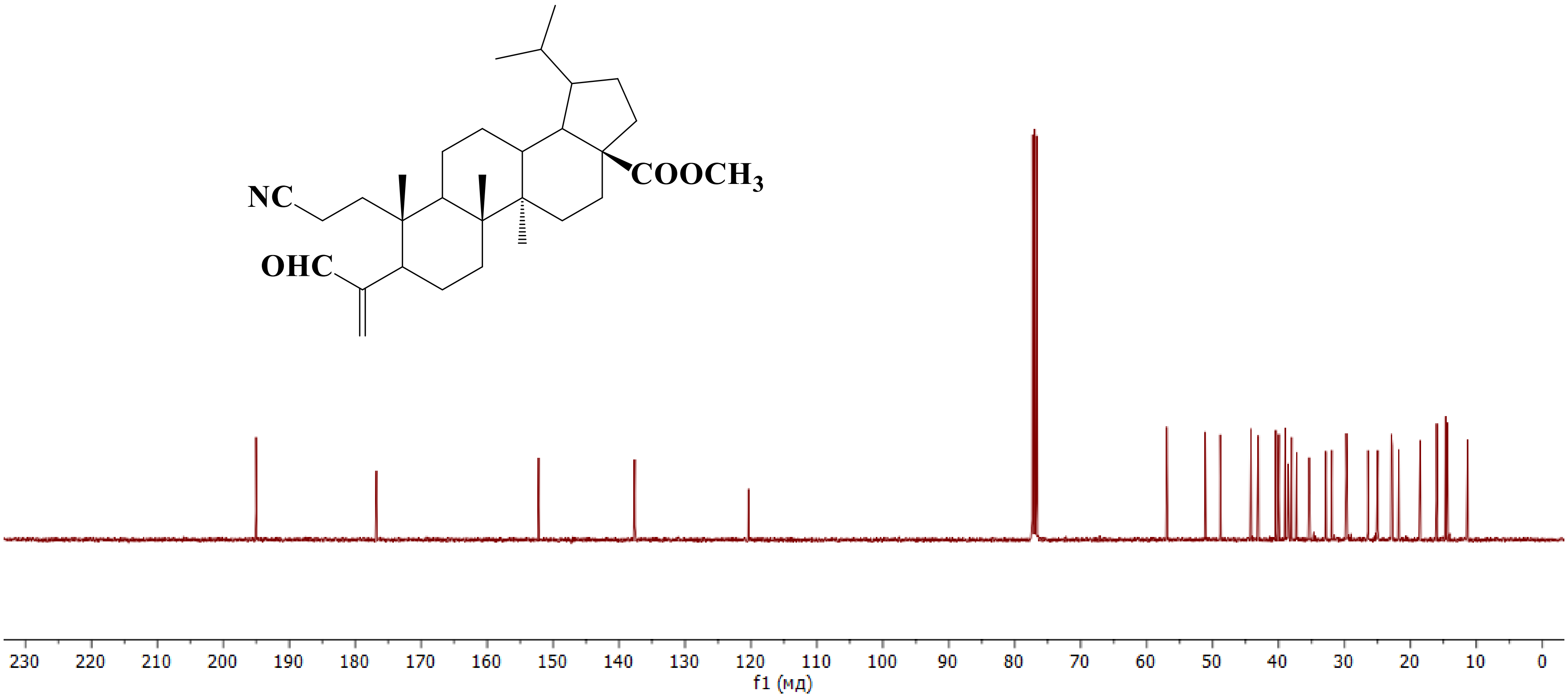

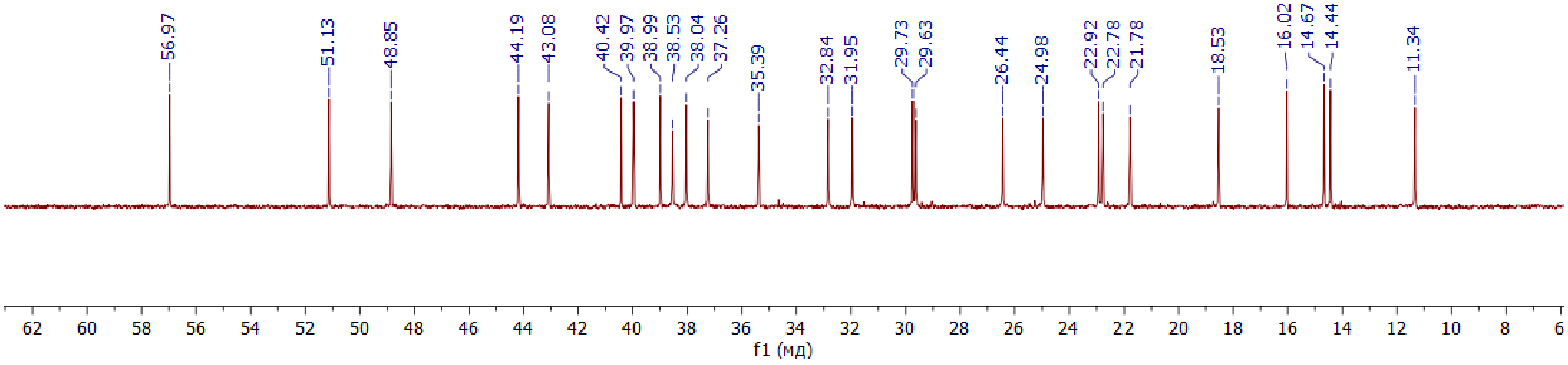
56.97
51.13
48.85
44.19
43.08
40.42
39.97
38.99
38.53
38.04
37.26
35.39
32.84
31.95
29.73
29.63
26.44
24.98
22.92
22.78
21.78
18.53
16.02
14.67
14.44
11.34
f1 (мд)

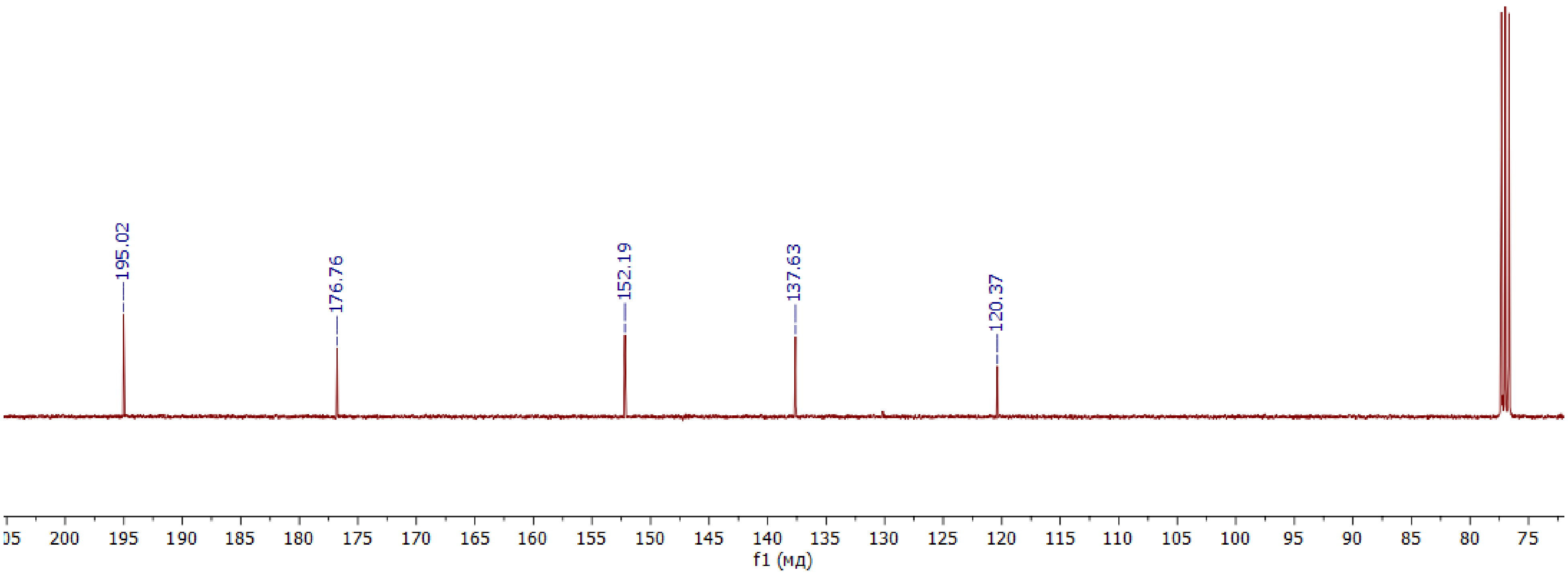
195.02
176.76
152.19
137.63
120.37
f1 (мд)

^{1}H NMR spectra of 28-Benzoxy-24-bromo-2-cyano-3,4-seco-3-norlup-4(23)-ene (6).

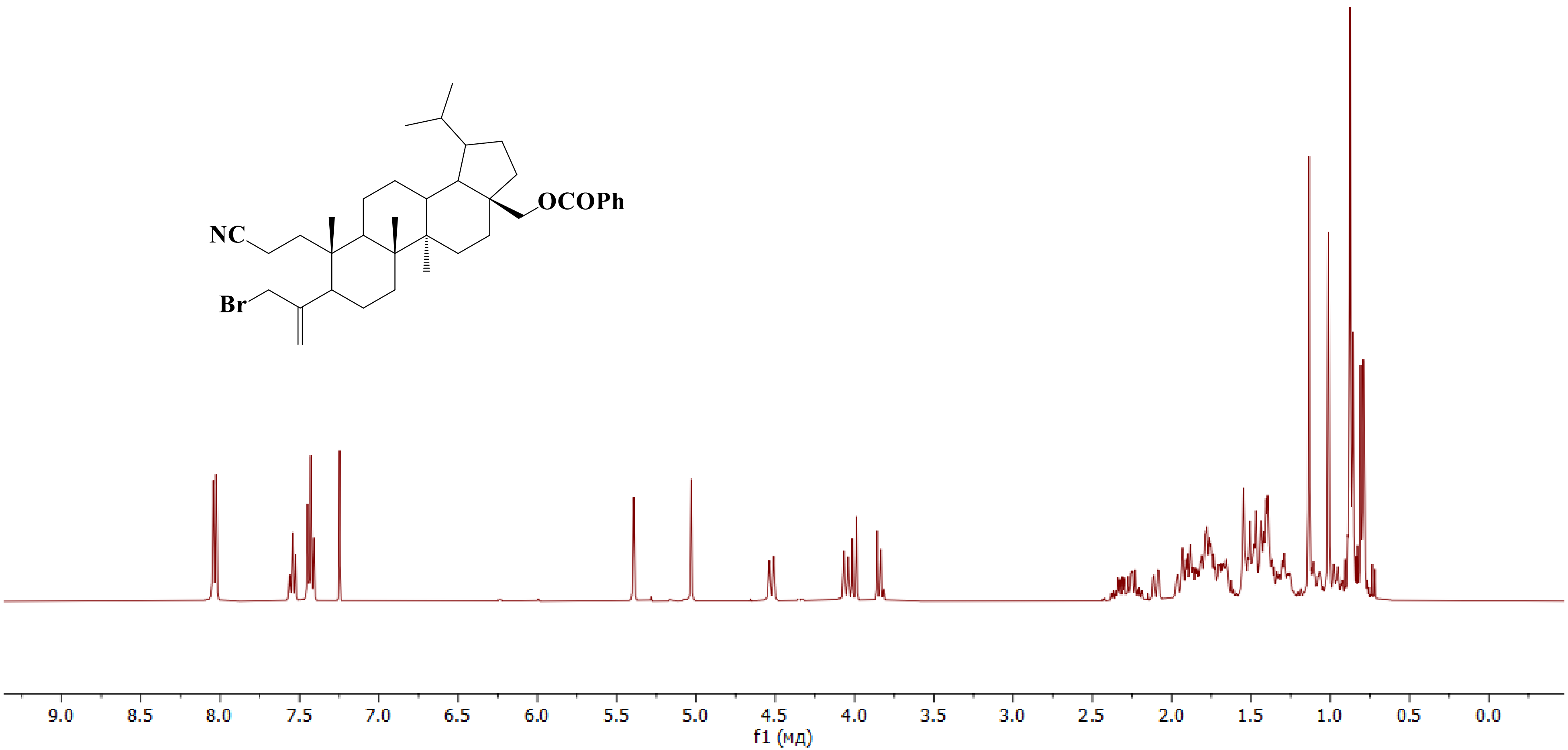

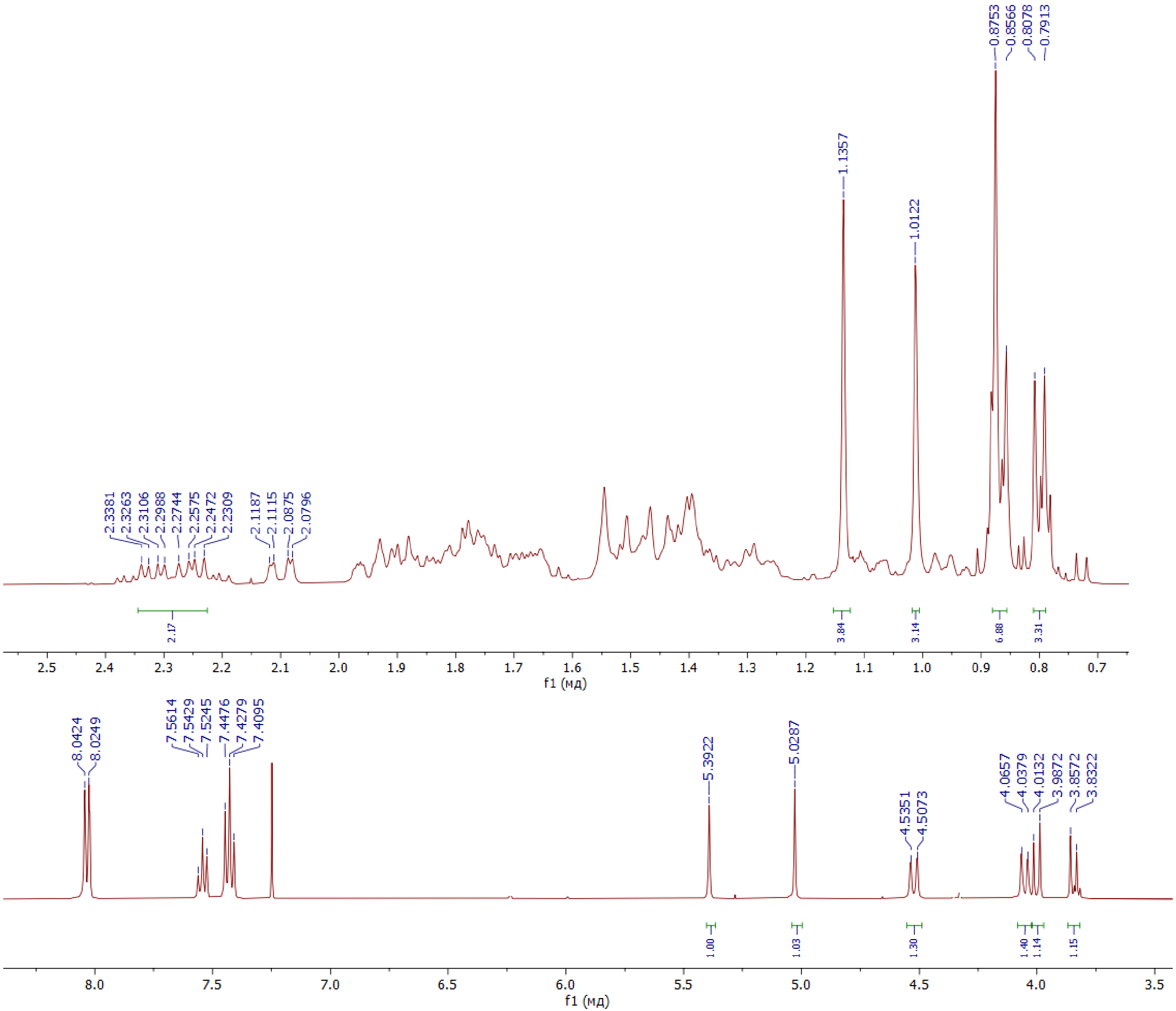
2.3381
2.3263
2.3106
2.2988
2.2744
2.2575
2.2472
2.2309
2.1187
2.1115
2.0875
2.0796
1.1357
1.0122
0.8753
0.8566
0.8078
0.7913
2.17
3.84
3.14
6.88
3.31
f1 (мд)
8.0424
8.0249
7.5614
7.5429
7.5245
7.4476
7.4279
7.4095
5.3922
5.0287
4.5351
4.5073
4.0657
4.0379
4.0132
3.9872
3.8572
3.8322
1.00
1.03
1.30
1.40
1.14
1.15
f1 (мд)

^{13}C NMR spectra of 28-Benzoxy-24-bromo-2-cyano-3,4-seco-3-norlup-4(23)-ene (6).

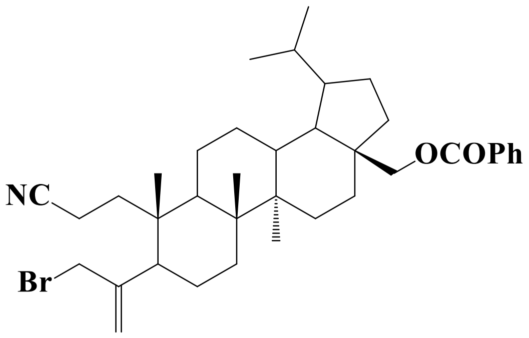

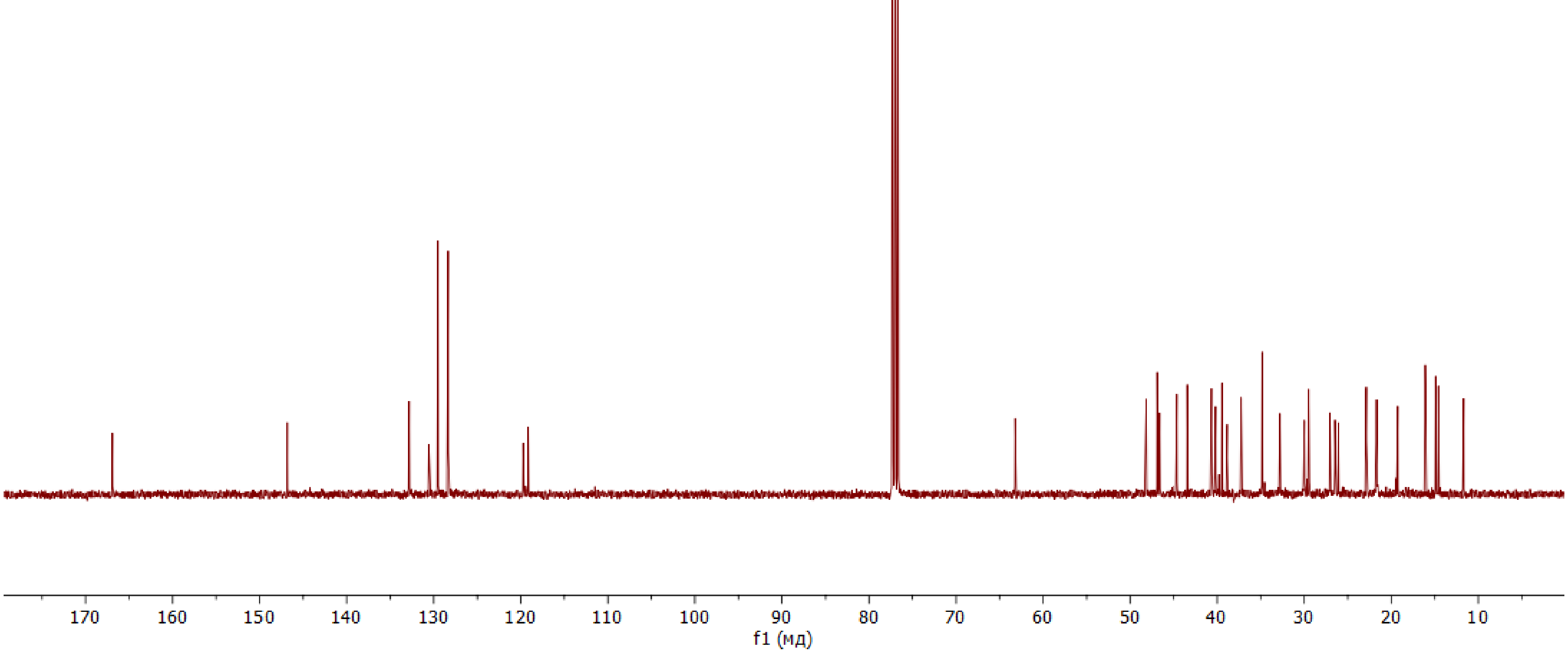

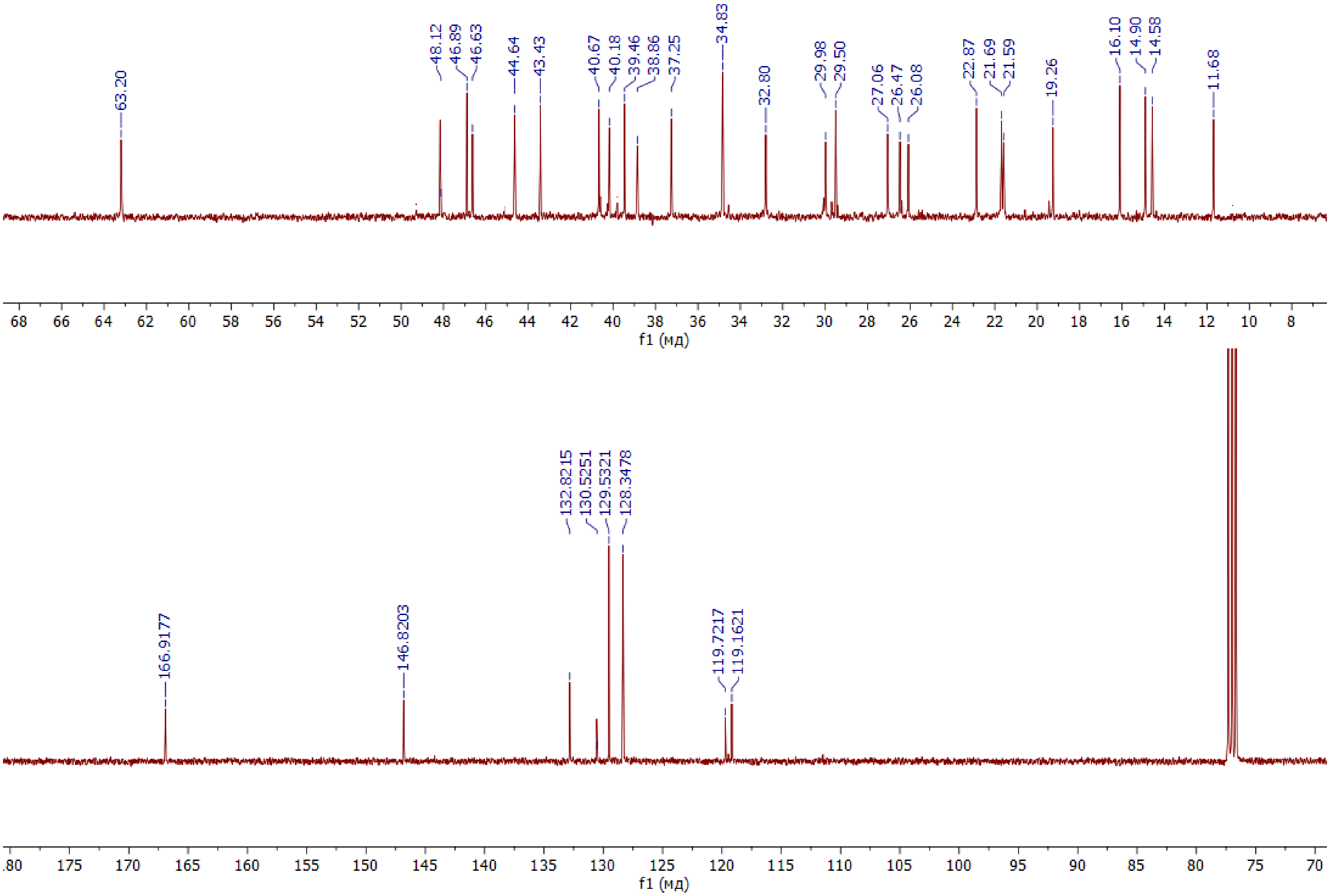
63.20
48.12
46.89
46.63
44.64
43.43
40.67
40.18
39.46
38.86
37.25
34.83
32.80
29.98
29.50
27.06
26.47
26.08
22.87
21.69
21.59
19.26
16.10
14.90
14.58
11.68
f1 (мд)
166.9177
146.8203
132.8215
130.5251
129.5321
128.3478
119.7217
119.1621
f1 (мд)

¹H NMR spectra of 24-Bromo-2-cyano-3-norlup-4(23)-en-28-oic acid methyl ester (7).

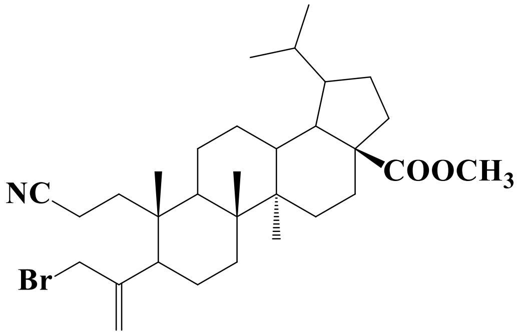

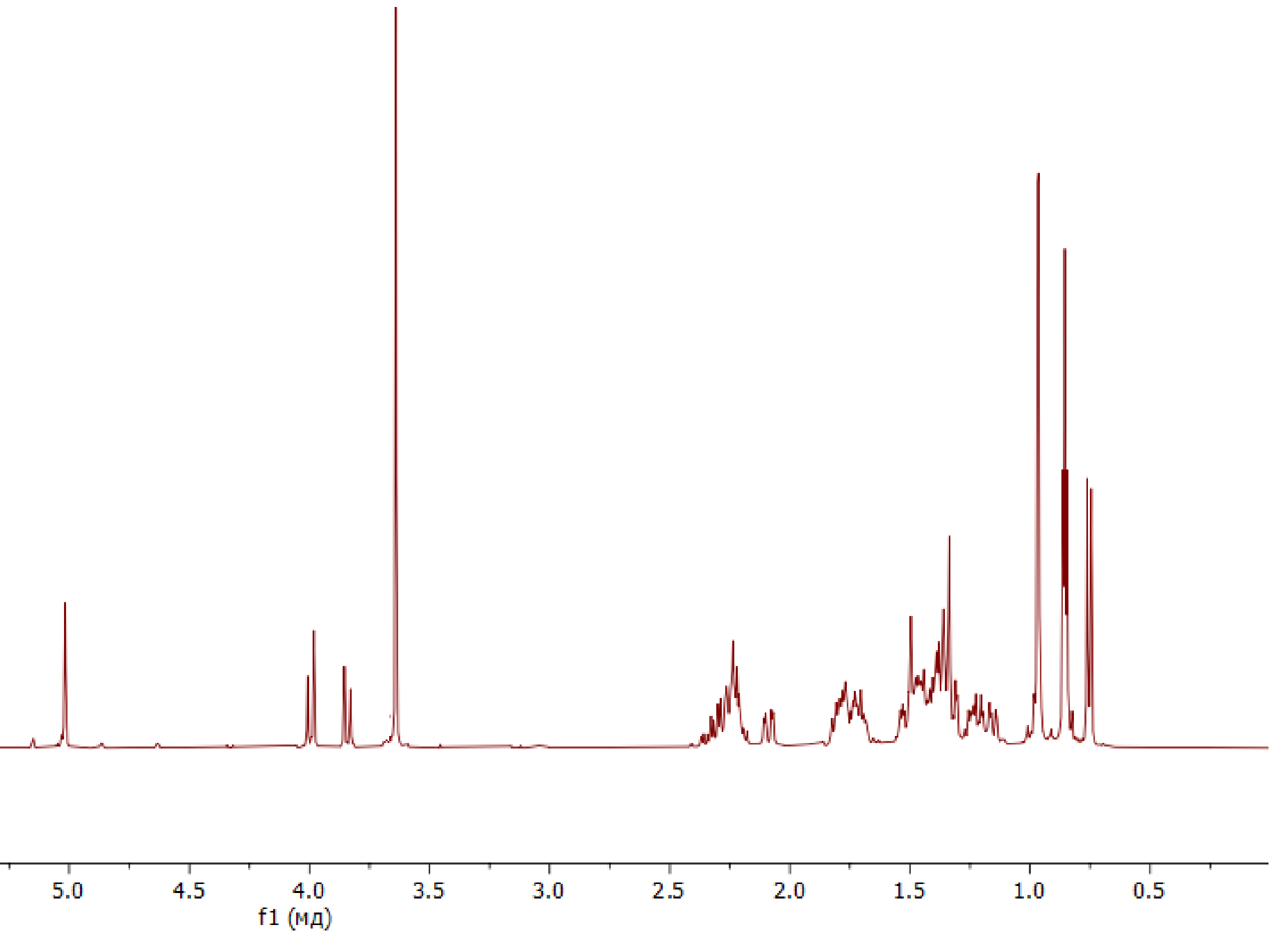

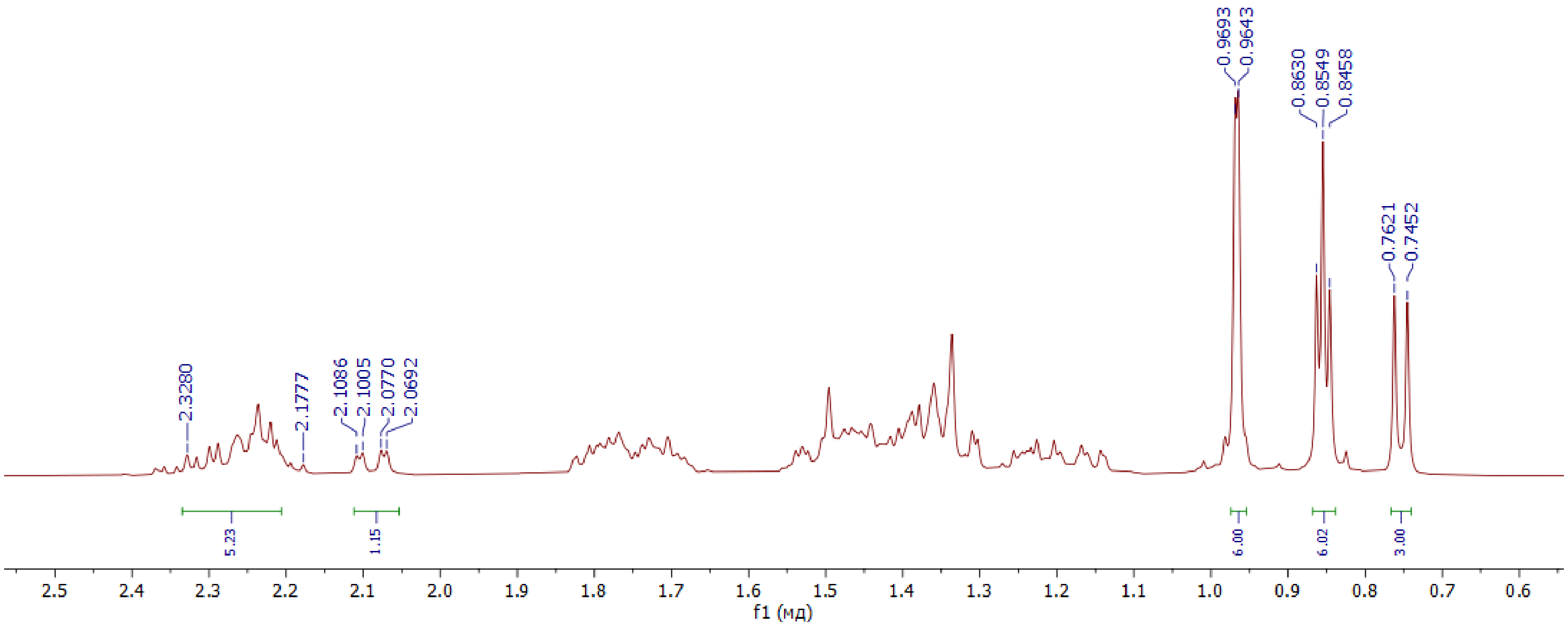

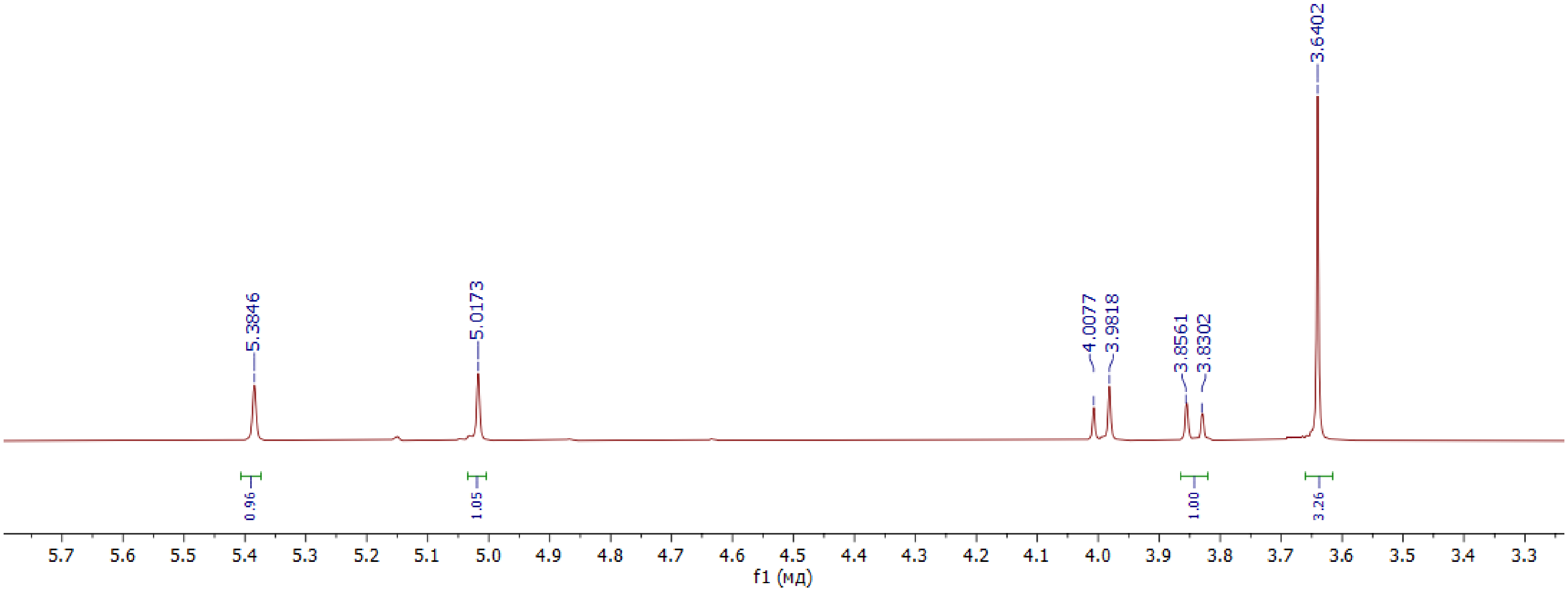

^{13}C NMR spectra of 24-Bromo-2-cyano-3-norlup-4(23)-en-28-oic acid methyl ester (7).

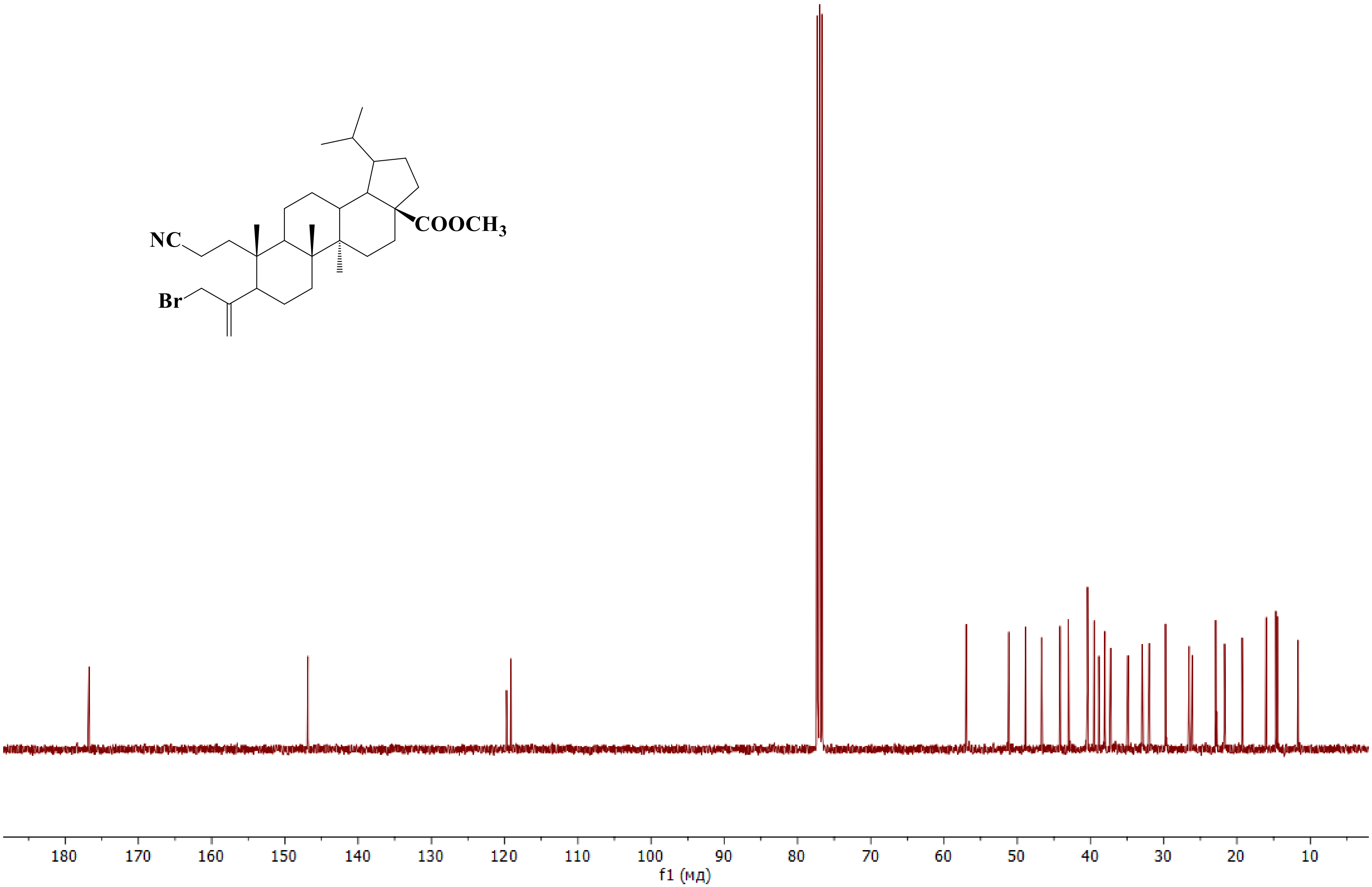
NC
Br
COOCH3
180 170 160 150 140 130 120 110 100 90 80 70 60 50 40 30 20 10
f1 (мд)

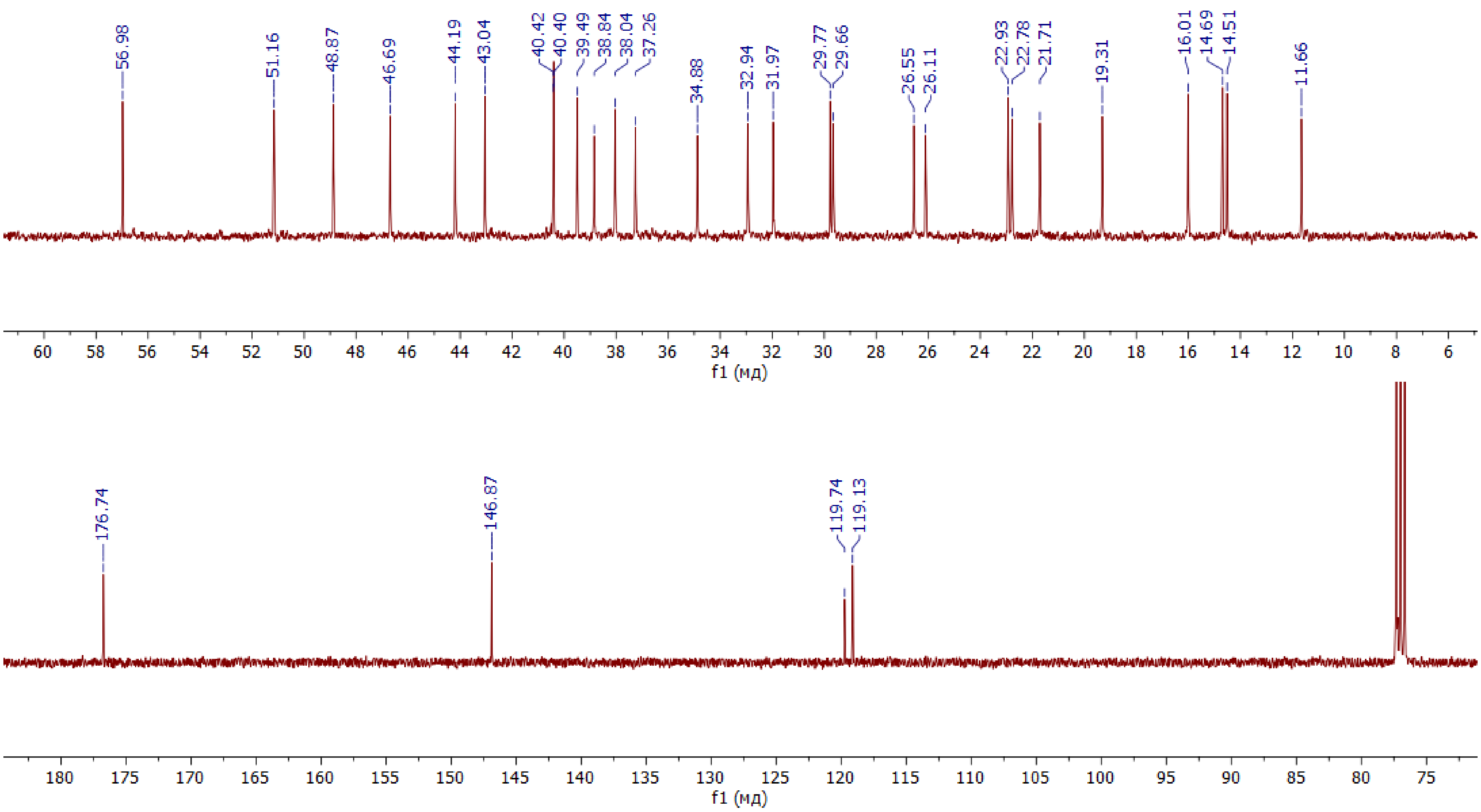
56.98
51.16
48.87
46.69
44.19
43.04
40.42
40.40
39.49
38.84
38.04
37.26
34.88
32.94
31.97
29.77
29.66
26.55
26.11
22.93
22.78
21.71
19.31
16.01
14.69
14.51
11.66
f1 (мд)
176.74
146.87
119.74
119.13
f1 (мд)

^{1}H NMR spectra of 2-Cyano-28-hydroxy-3-norlup-2(24),4(23)-diene (8).

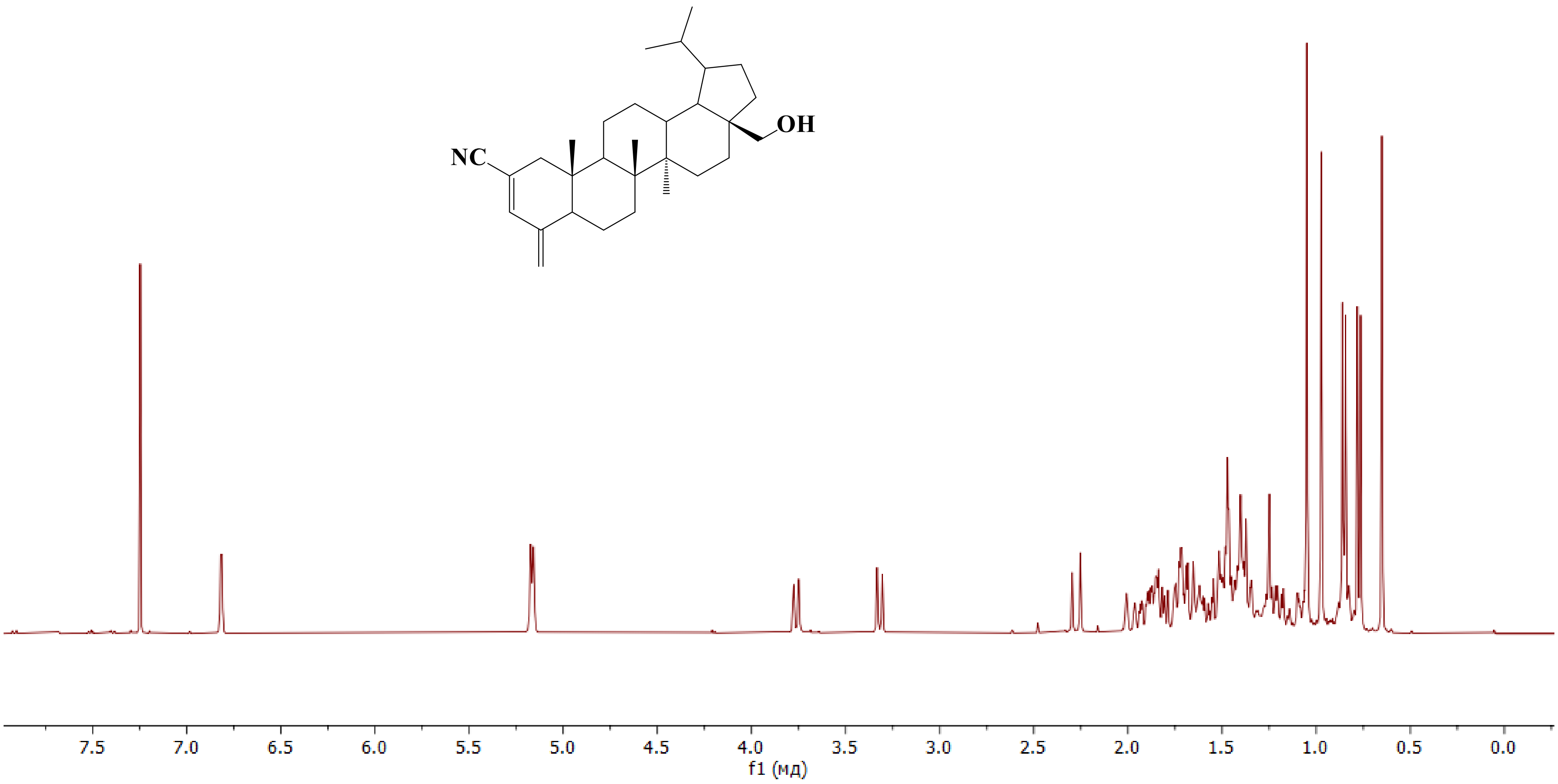

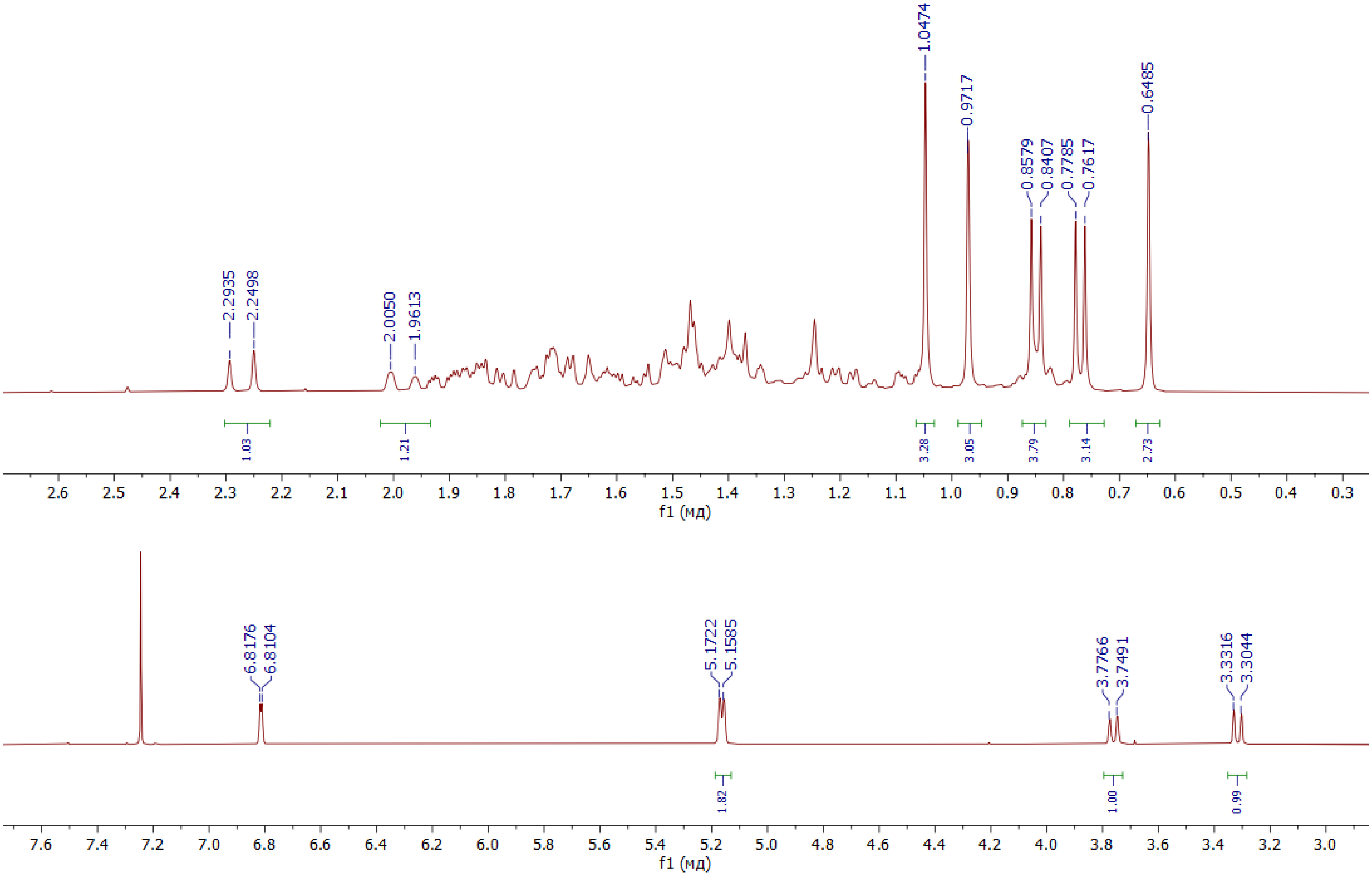

2.2935
2.2498
2.0050
1.9613
1.0474
0.9717
0.8579
0.8407
0.7785
0.7617
0.6485
1.03
1.21
3.28
3.05
3.79
3.14
2.73
2.6 2.5 2.4 2.3 2.2 2.1 2.0 1.9 1.8 1.7 1.6 1.5 1.4 1.3 1.2 1.1 1.0 0.9 0.8 0.7 0.6 0.5 0.4 0.3
f1 (мд)
6.8176
6.8104
5.1722
5.1585
3.7766
3.7491
3.3316
3.3044
1.82
1.00
0.99
7.6 7.4 7.2 7.0 6.8 6.6 6.4 6.2 6.0 5.8 5.6 5.4 5.2 5.0 4.8 4.6 4.4 4.2 4.0 3.8 3.6 3.4 3.2 3.0
f1 (мд)

^{13}C NMR spectra of 2-Cyano-28-hydroxy-3-norlup-2(24),4(23)-diene (8).

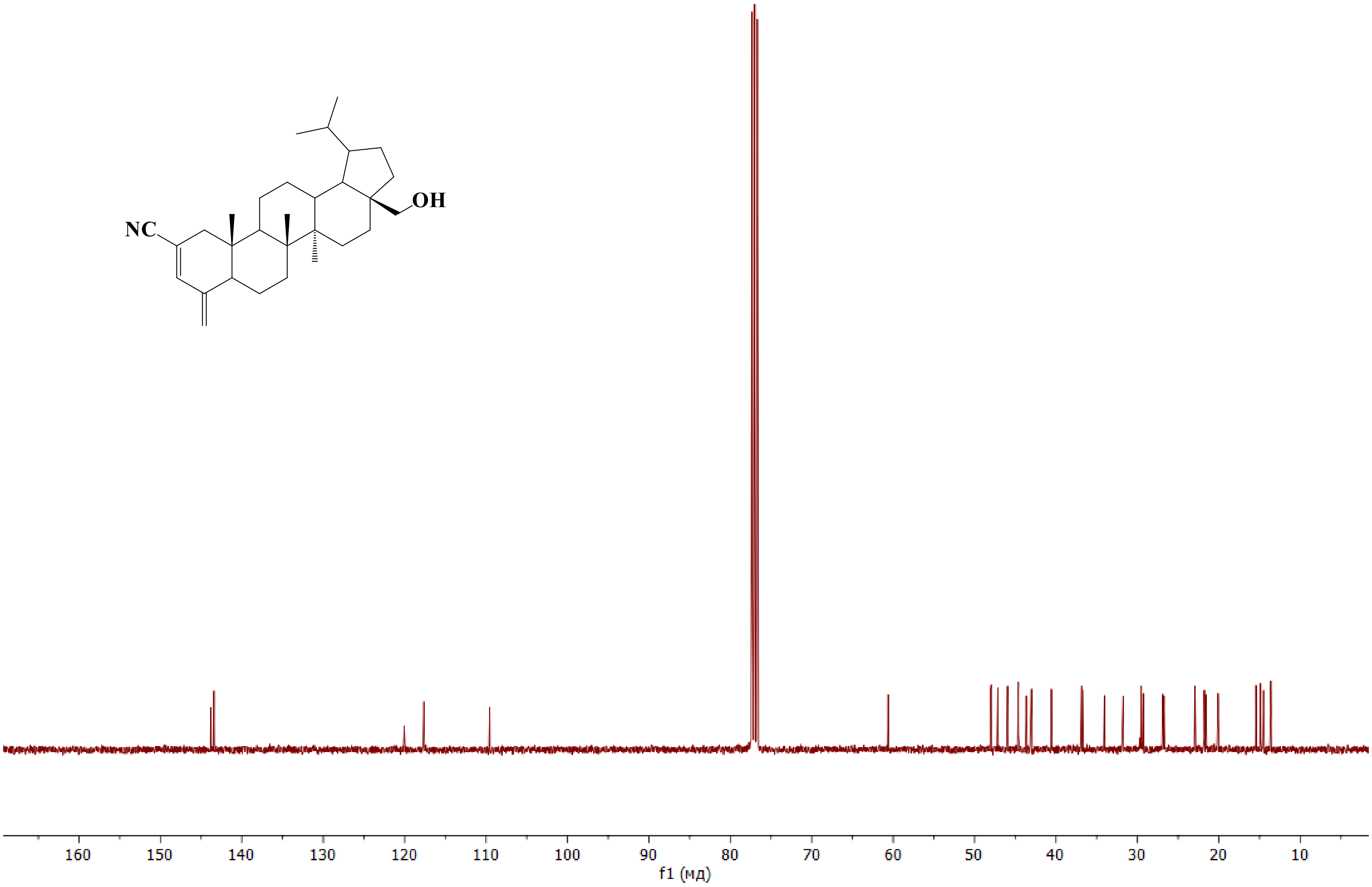

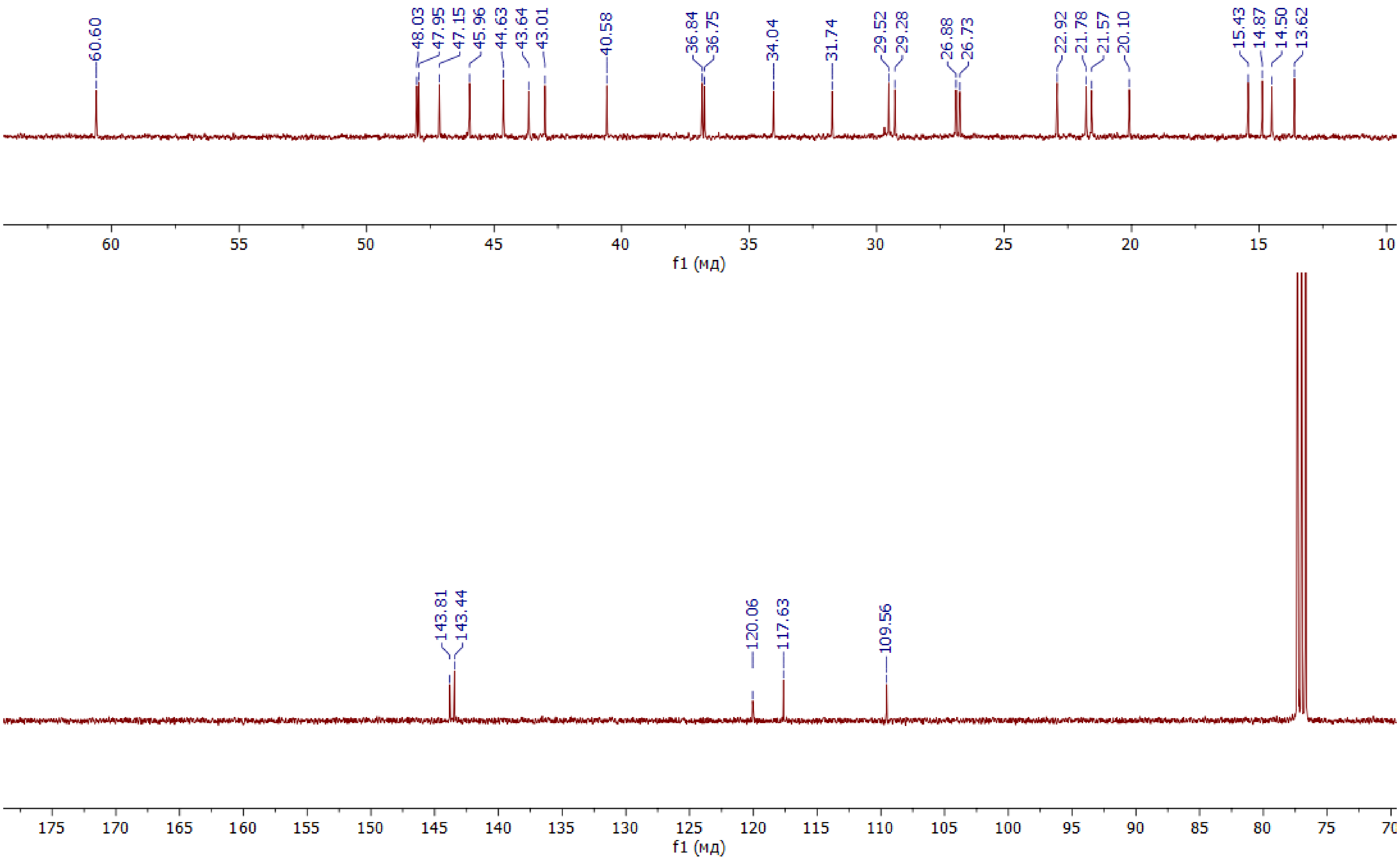
60.60
48.03
47.95
47.15
45.96
44.63
43.64
43.01
40.58
36.84
36.75
34.04
31.74
29.52
29.28
26.88
26.73
22.92
21.78
21.57
20.10
15.43
14.87
14.50
13.62
60 55 50 45 40 35 30 25 20 15 10
f1 (мд)
143.81
143.44
120.06
117.63
109.56
175 170 165 160 155 150 145 140 135 130 125 120 115 110 105 100 95 90 85 80 75 70
f1 (мд)

^{1}H NMR spectra of 2-Cyano-24,28-dihydroxy-3-norlup-4(23)-ene (9).

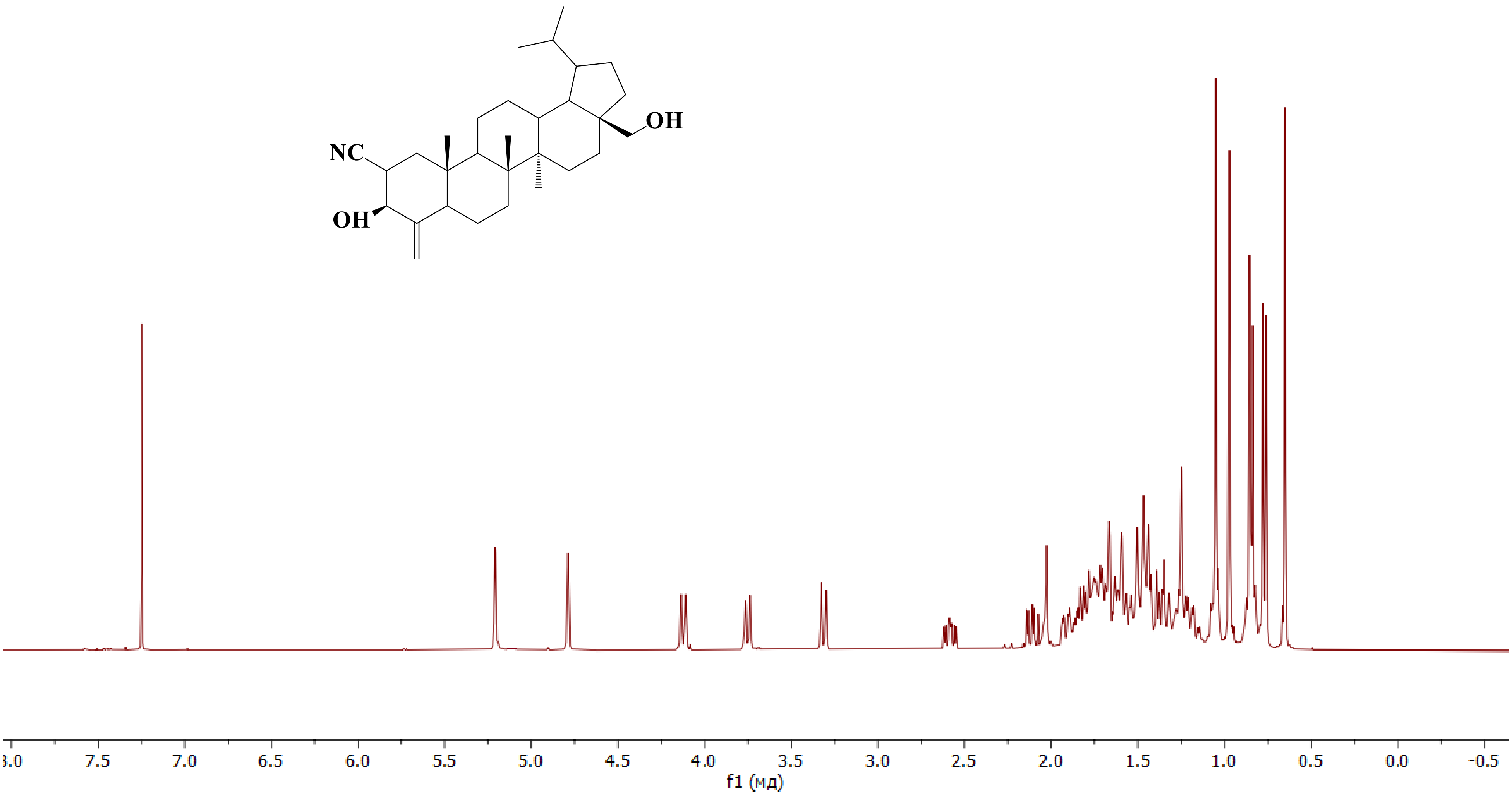

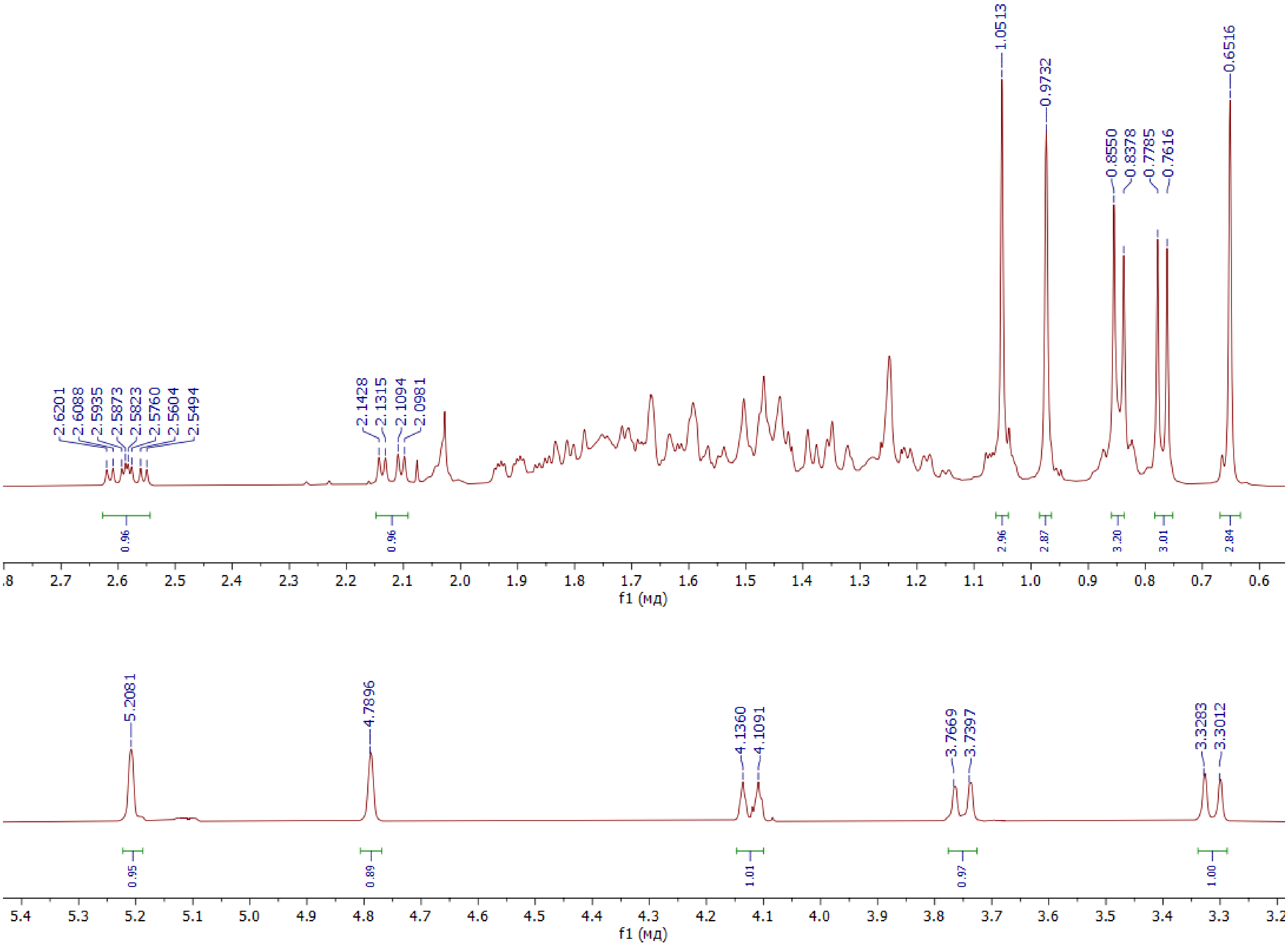
2.6201
2.6088
2.5935
2.5873
2.5823
2.5760
2.5604
2.5494
2.1428
2.1315
2.1094
2.0981
1.0513
0.9732
0.8550
0.8378
0.7785
0.7616
0.6516
0.96
0.96
2.96
2.87
3.20
3.01
2.84
f1 (мд)
5.2081
4.7896
4.1360
4.1091
3.7669
3.7397
3.3283
3.3012
0.95
0.89
1.01
0.97
1.00
f1 (мд)

^{13}C NMR spectra of 2-Cyano-24,28-dihydroxy-3-norlup-4(23)-ene (9).

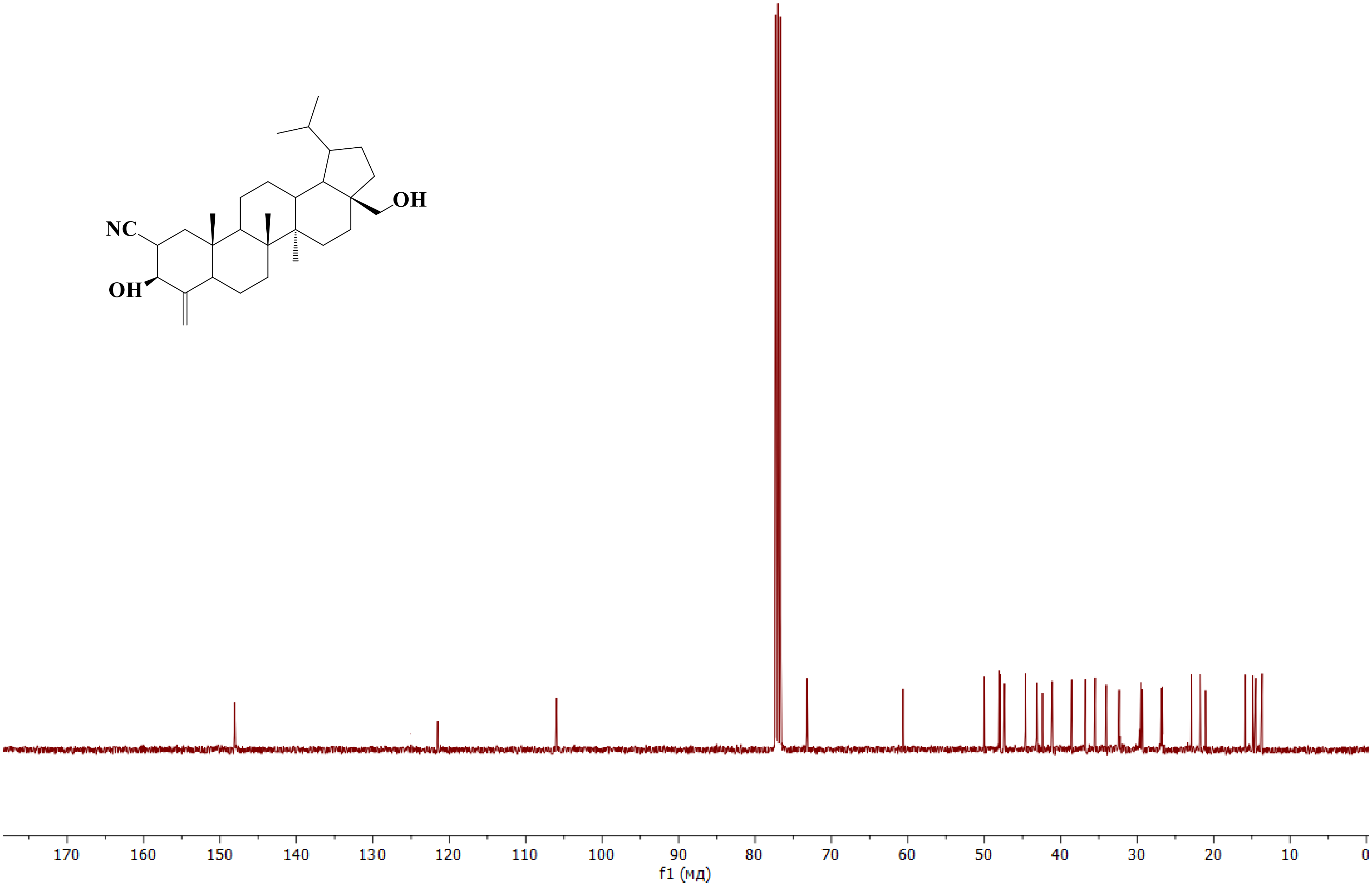

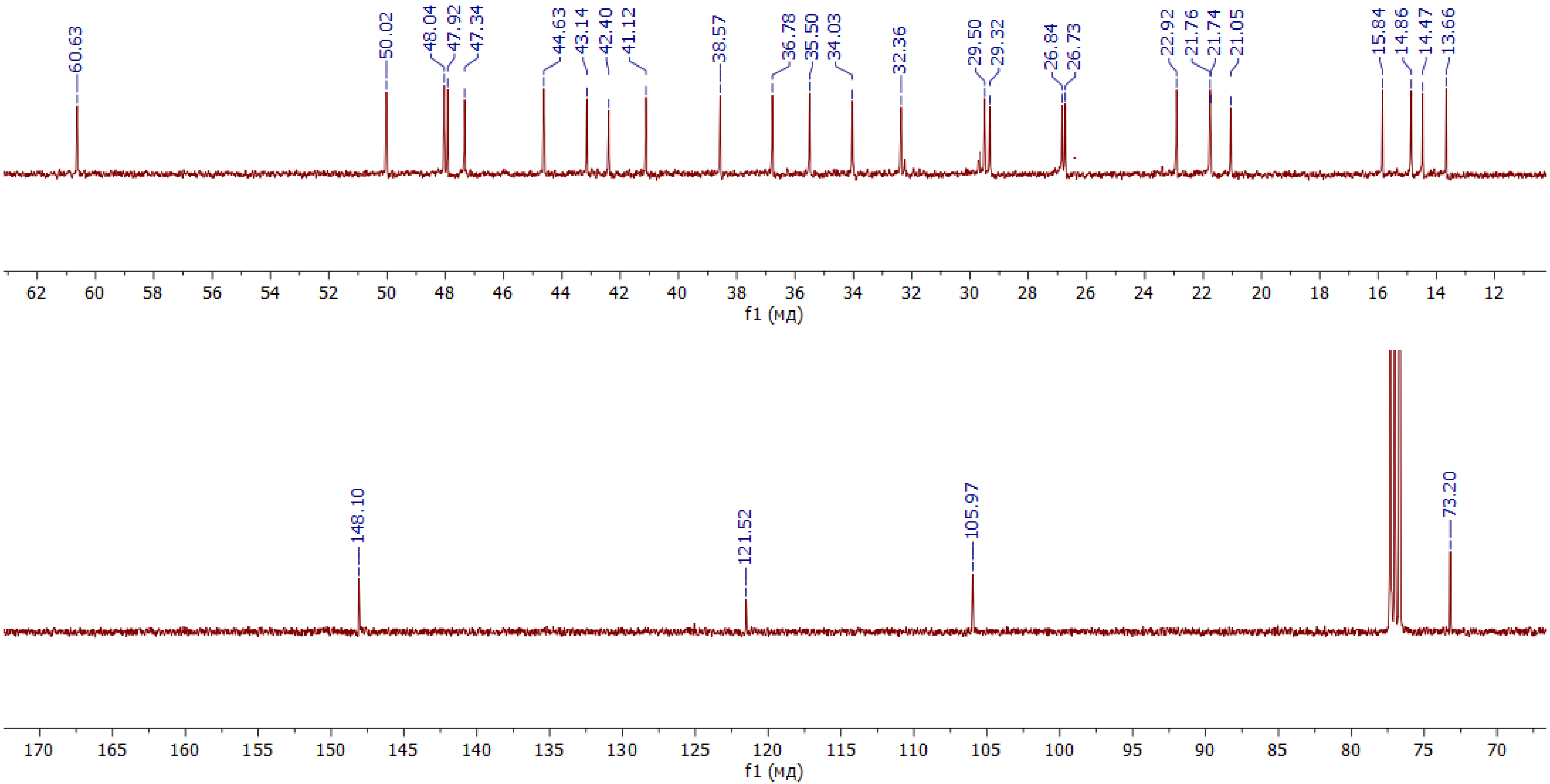

60.63
50.02
48.04
47.92
47.34
44.63
43.14
42.40
41.12
38.57
36.78
35.50
34.03
32.36
29.50
29.32
26.84
26.73
22.92
21.76
21.74
21.05
15.84
14.86
14.47
13.66
f1 (мд)
148.10
121.52
105.97
73.20
f1 (мд)

^{1}H NMR spectra of 2-Cyano-3-norlup-2(24),4(23)-diene-28-oic acid methyl ester (10).

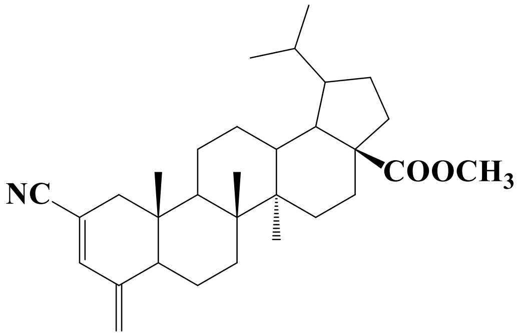

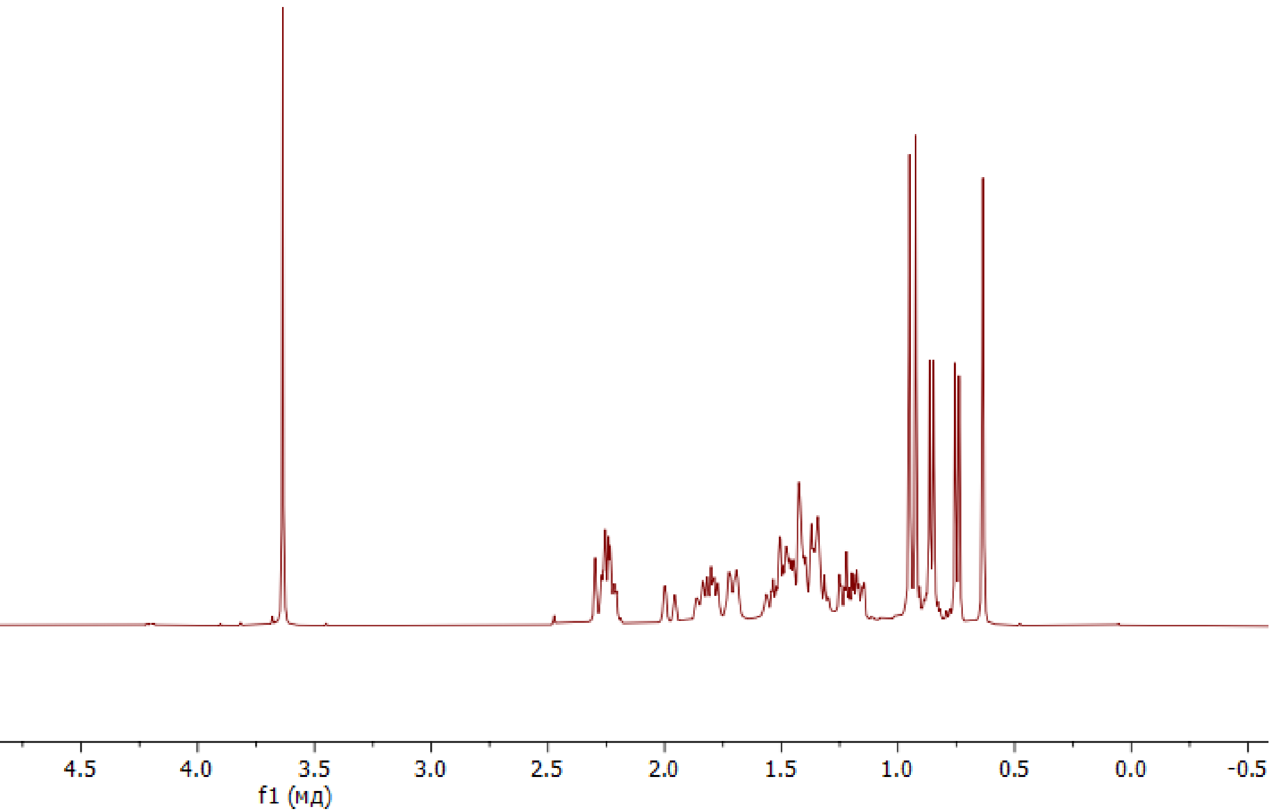

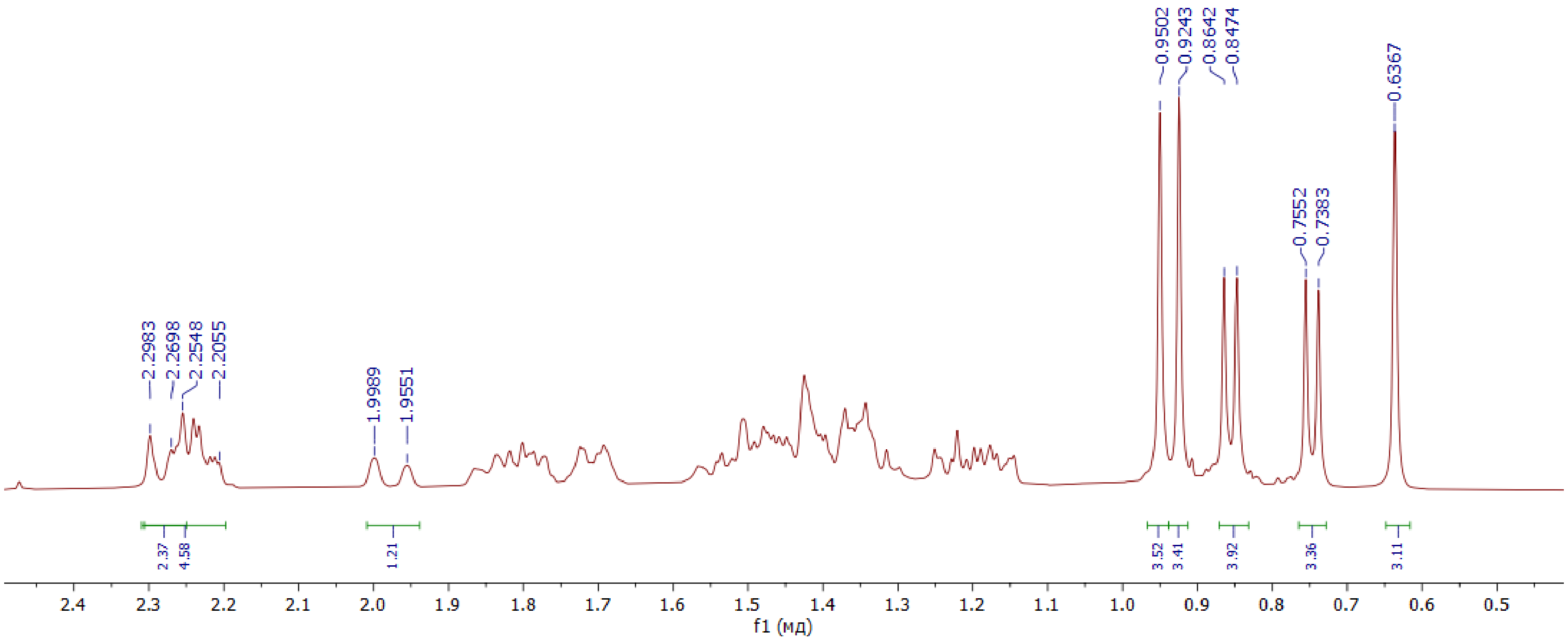
2.2983
2.2698
2.2548
2.2055
1.9989
1.9551
0.9502
0.9243
0.8642
0.8474
0.7552
0.7383
0.6367
2.37
4.58
1.21
3.52
3.41
3.92
3.36
3.11
f1 (мд)
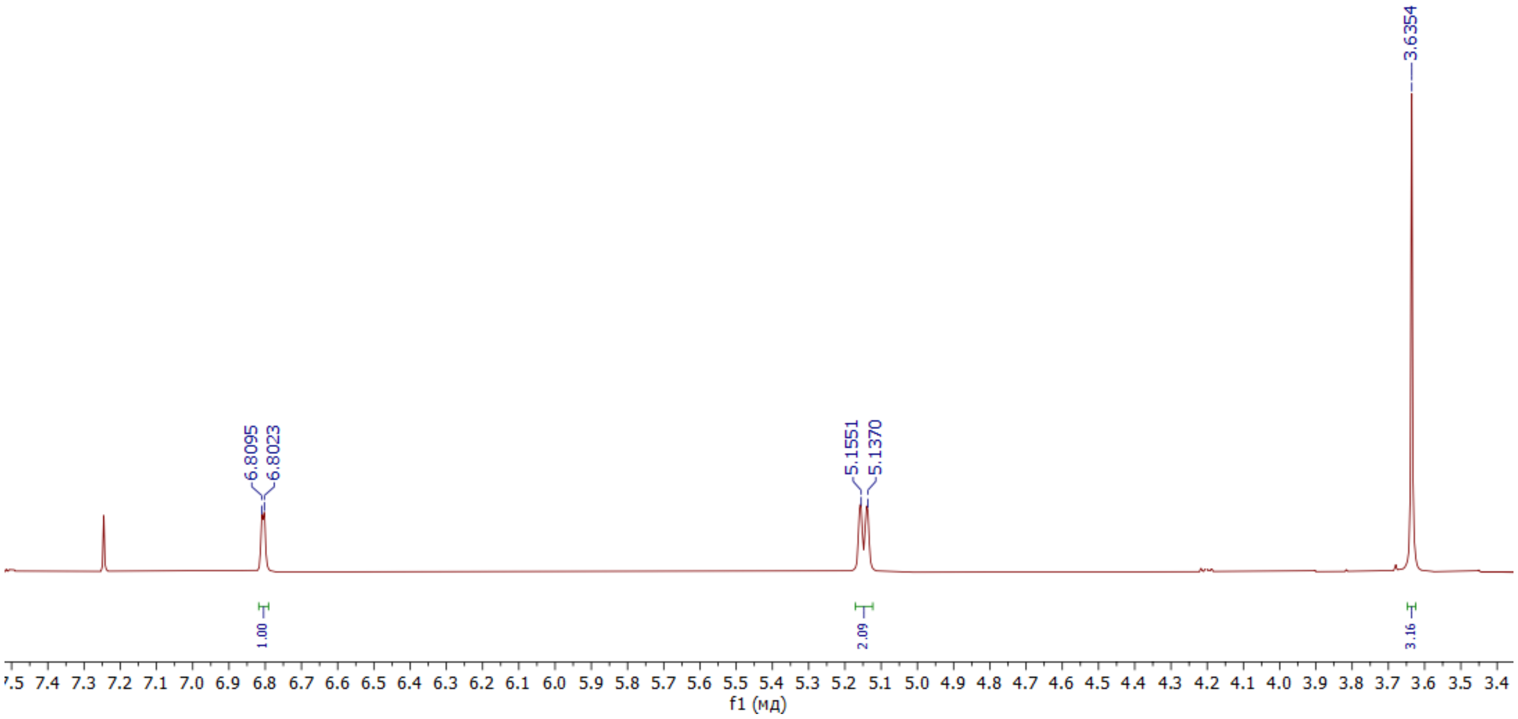
6.8095
6.8023
5.1551
5.1370
3.6354
1.00
2.09
3.16
f1 (мд)

^{13}C NMR spectra of 2-Cyano-3-norlup-2(24),4(23)-diene-28-oic acid methyl ester (10).

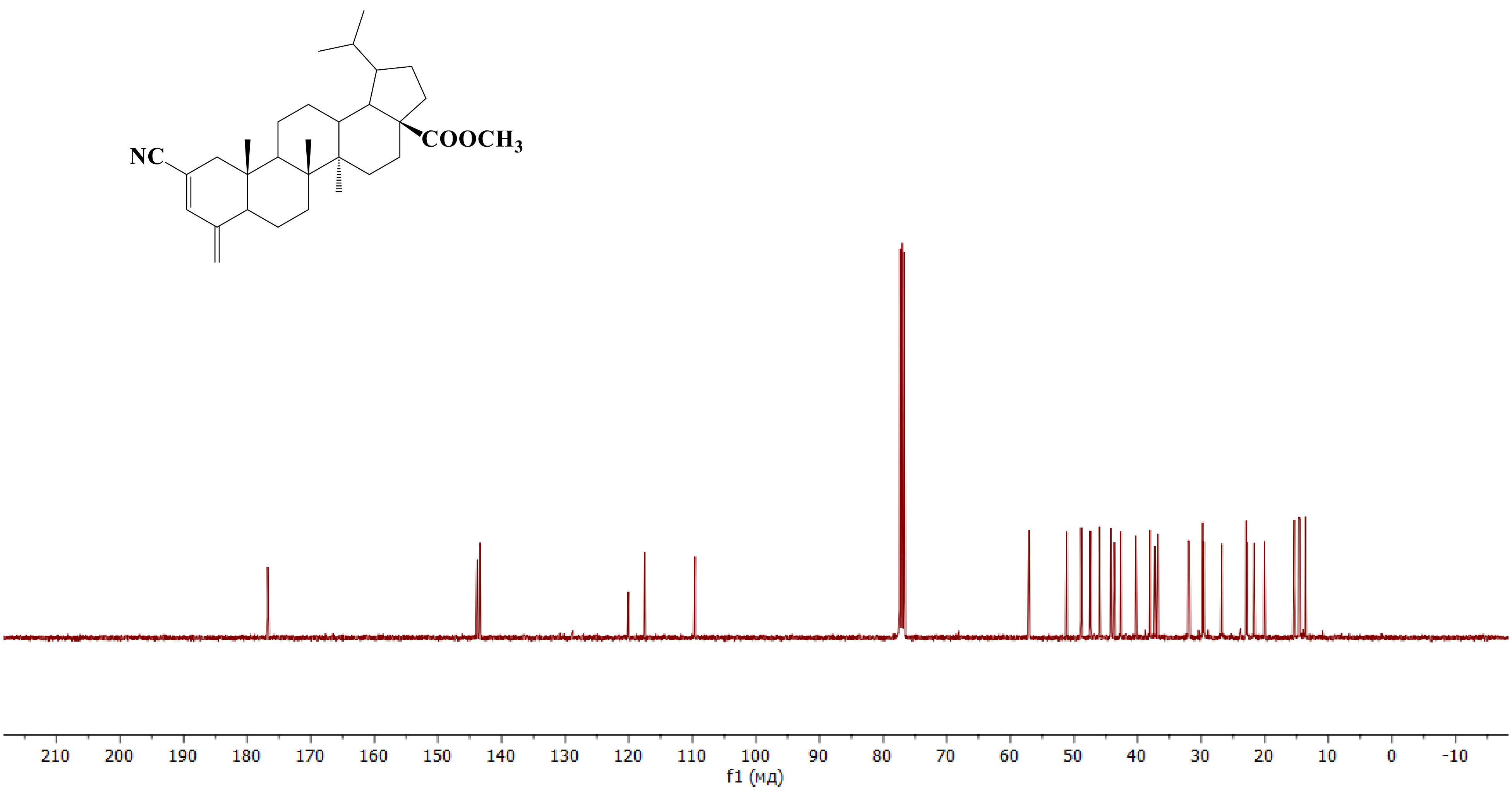

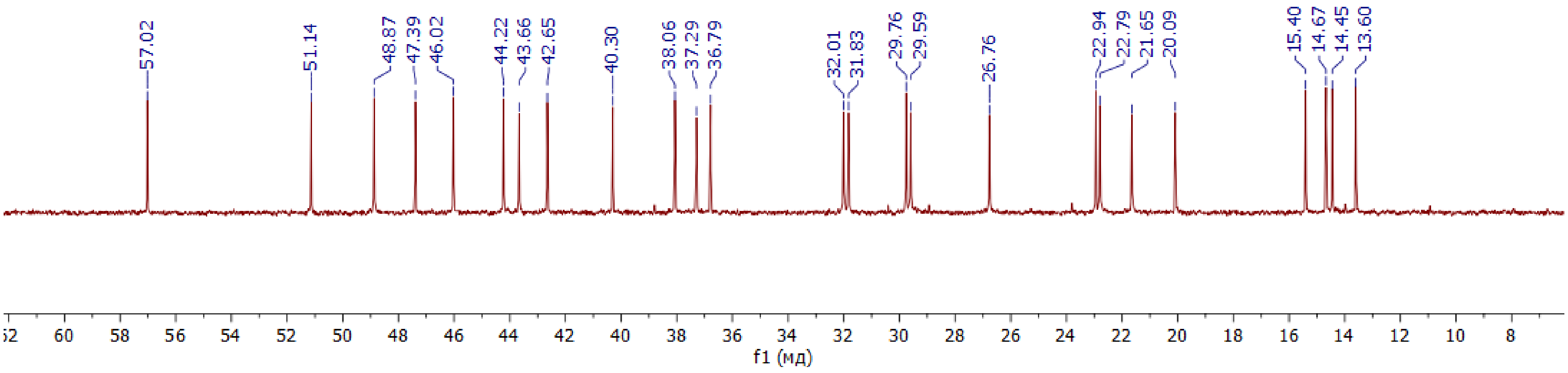
57.02
51.14
48.87
47.39
46.02
44.22
43.66
42.65
40.30
38.06
37.29
36.79
32.01
31.83
29.76
29.59
26.76
22.94
22.79
21.65
20.09
15.40
14.67
14.45
13.60
62 60 58 56 54 52 50 48 46 44 42 40 38 36 34 32 30 28 26 24 22 20 18 16 14 12 10 8
f1 (мд)

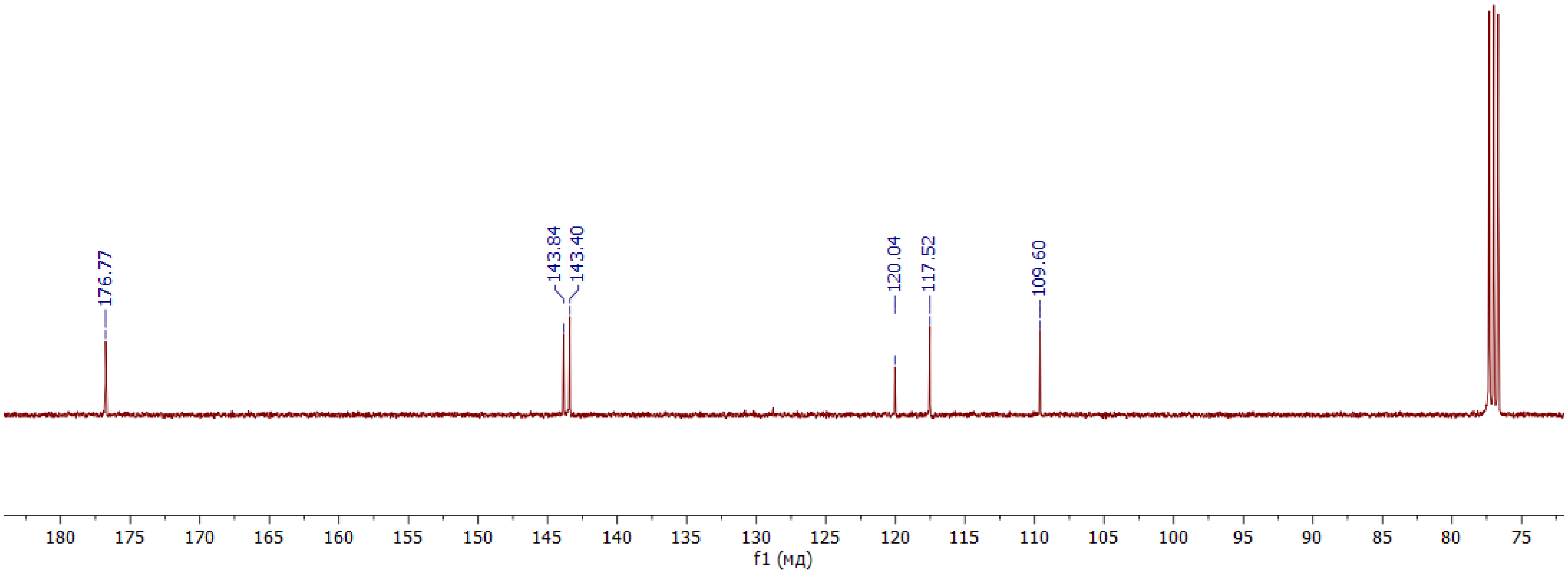
176.77
143.84
143.40
120.04
117.52
109.60
180 175 170 165 160 155 150 145 140 135 130 125 120 115 110 105 100 95 90 85 80 75
f1 (мд)

^{1}H NMR spectra of 2-Cyano-24-hydroxy-3-norlup-4(23)-en-28-oic acid methyl ester (11).

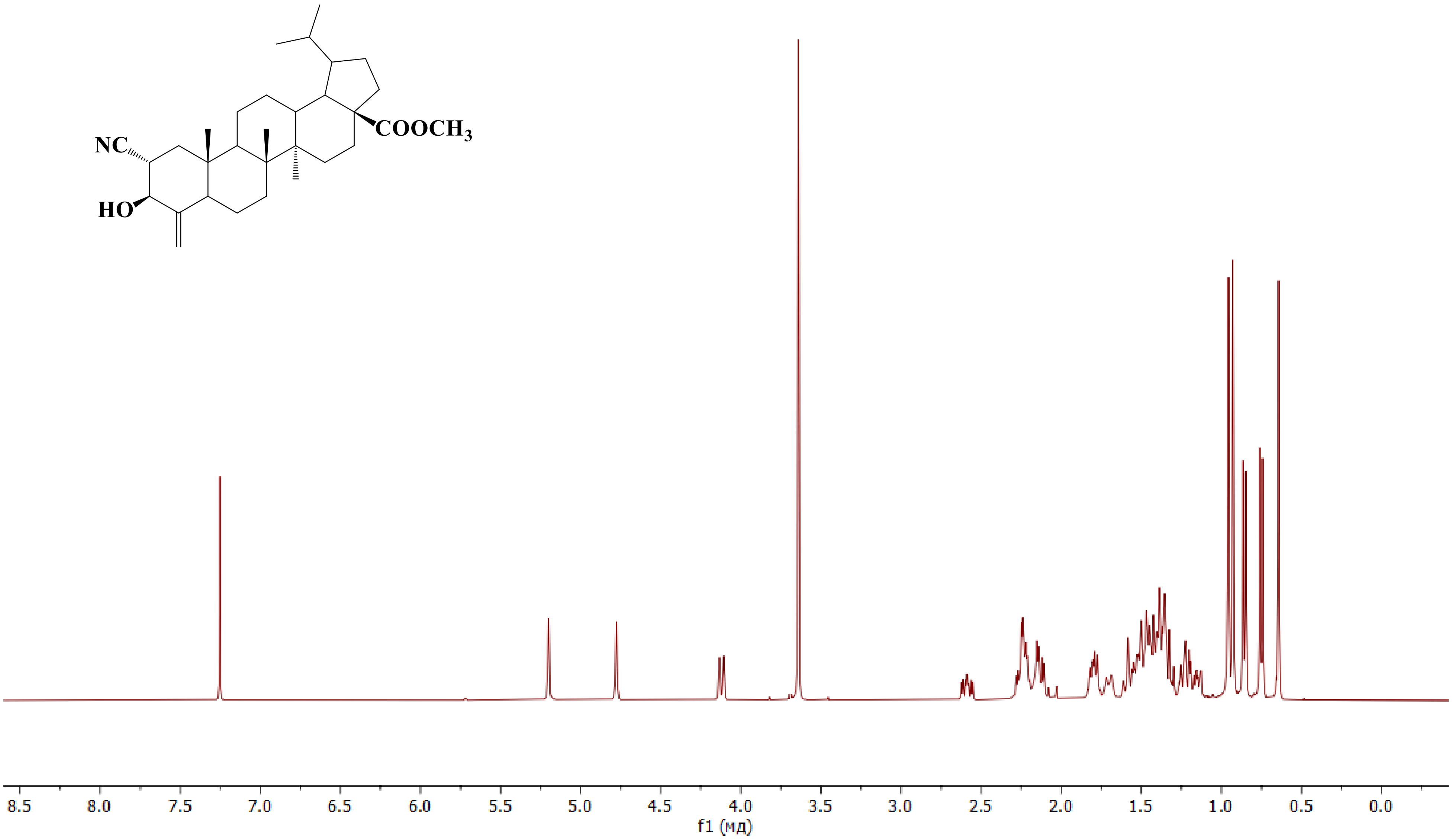

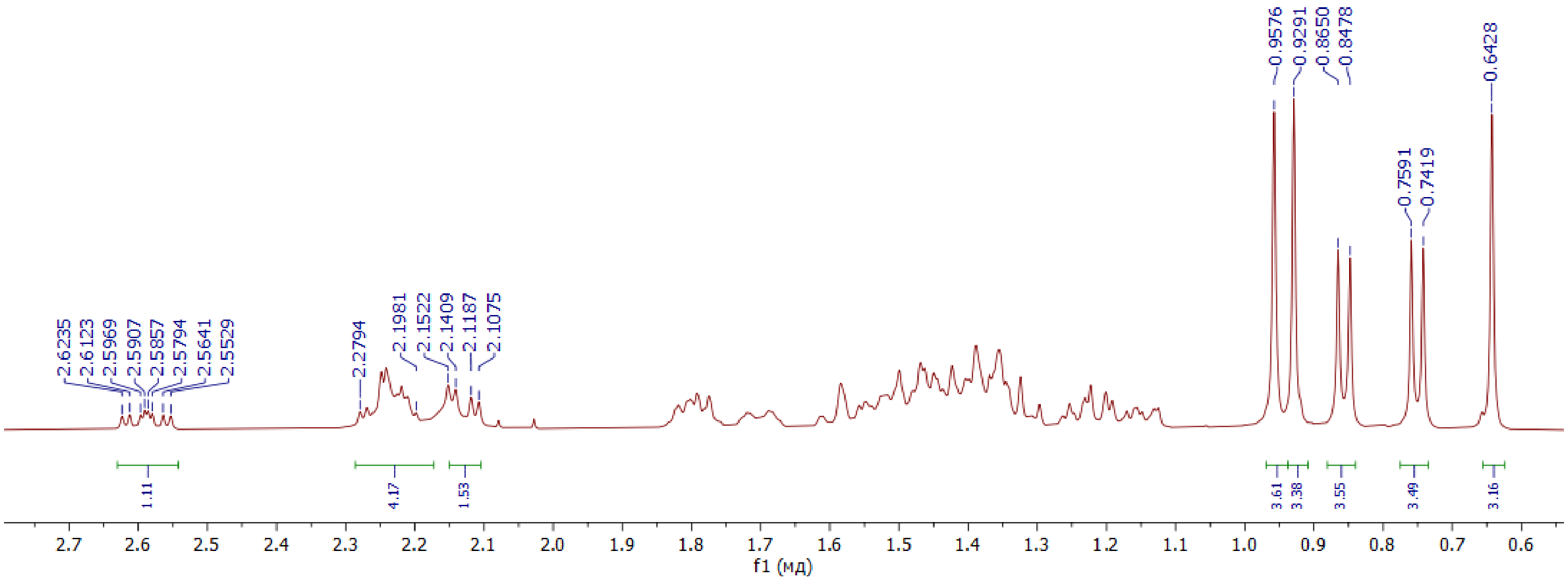
2.6235
2.6123
2.5969
2.5907
2.5857
2.5794
2.5641
2.5529
2.2794
2.1981
2.1522
2.1409
2.1187
2.1075
0.9576
0.9291
0.8650
0.8478
0.7591
0.7419
0.6428
1.11
4.17
1.53
3.61
3.38
3.55
3.49
3.16
2.7 2.6 2.5 2.4 2.3 2.2 2.1 2.0 1.9 1.8 1.7 1.6 1.5 1.4 1.3 1.2 1.1 1.0 0.9 0.8 0.7 0.6
f1 (мд)

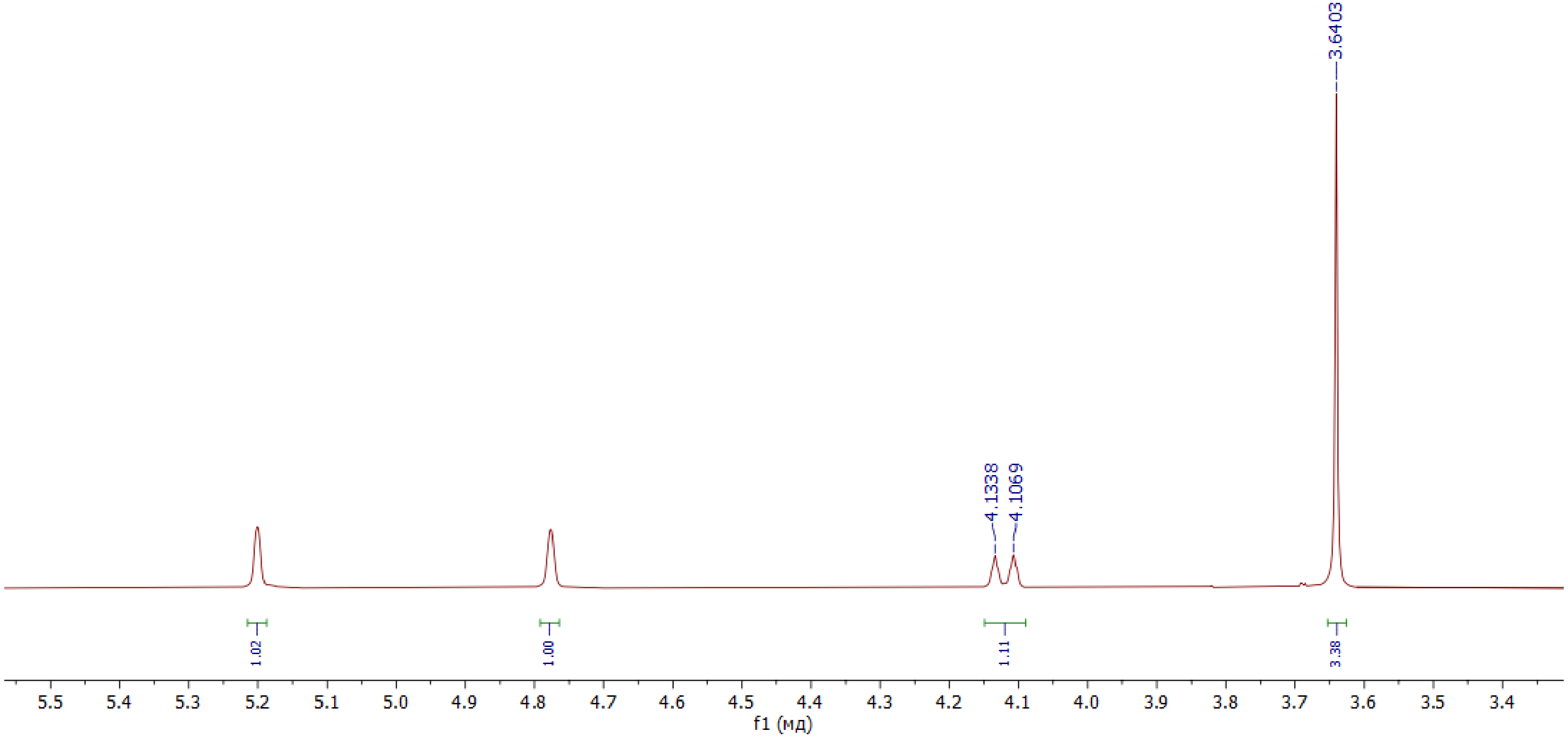
3.6403
4.1338
4.1069
1.02
1.00
1.11
3.38
5.5 5.4 5.3 5.2 5.1 5.0 4.9 4.8 4.7 4.6 4.5 4.4 4.3 4.2 4.1 4.0 3.9 3.8 3.7 3.6 3.5 3.4
f1 (мд)

^{13}C NMR spectra of 2-Cyano-24-hydroxy-3-norlup-4(23)-en-28-oic acid methyl ester (11).

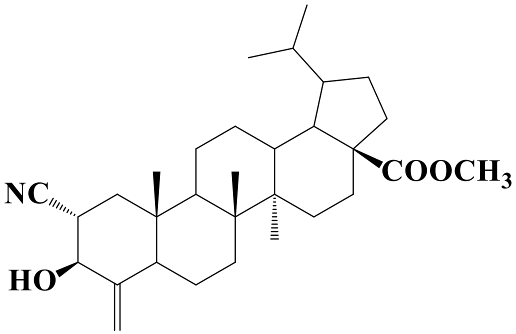

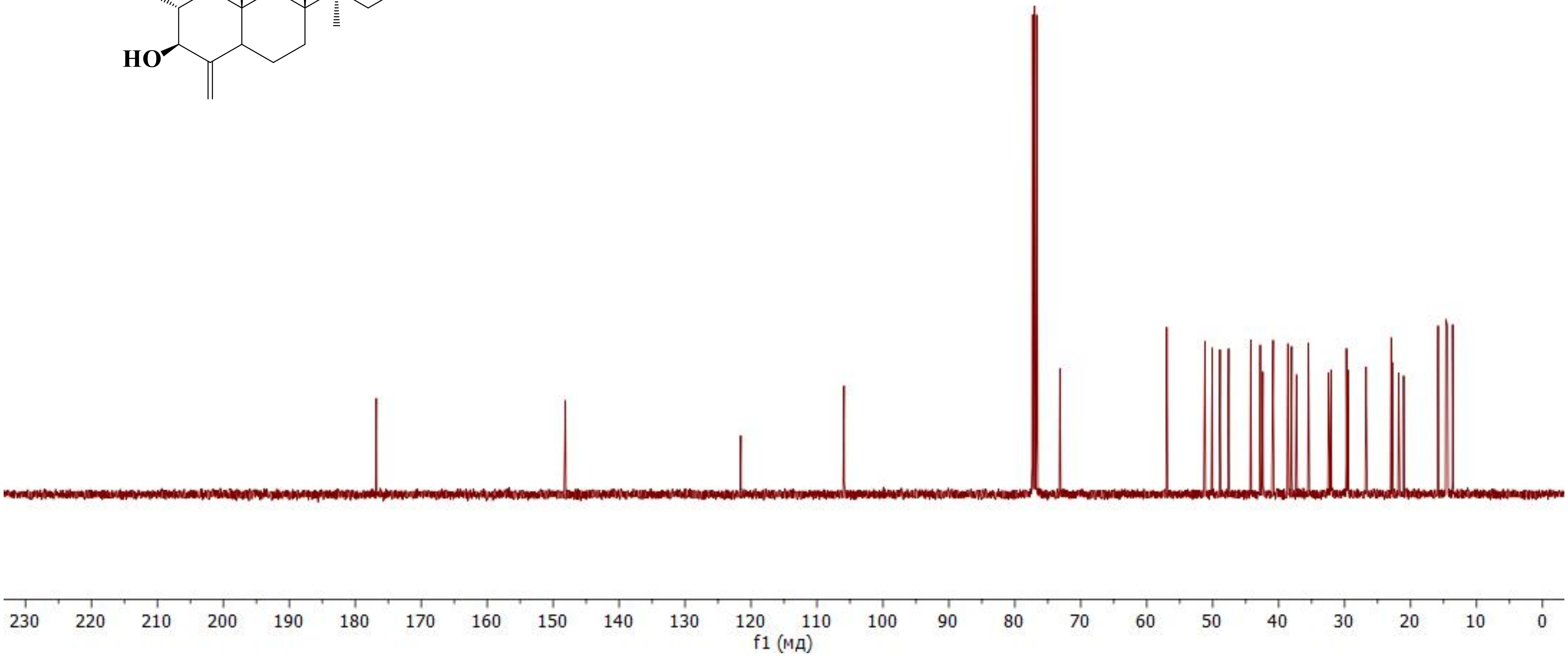

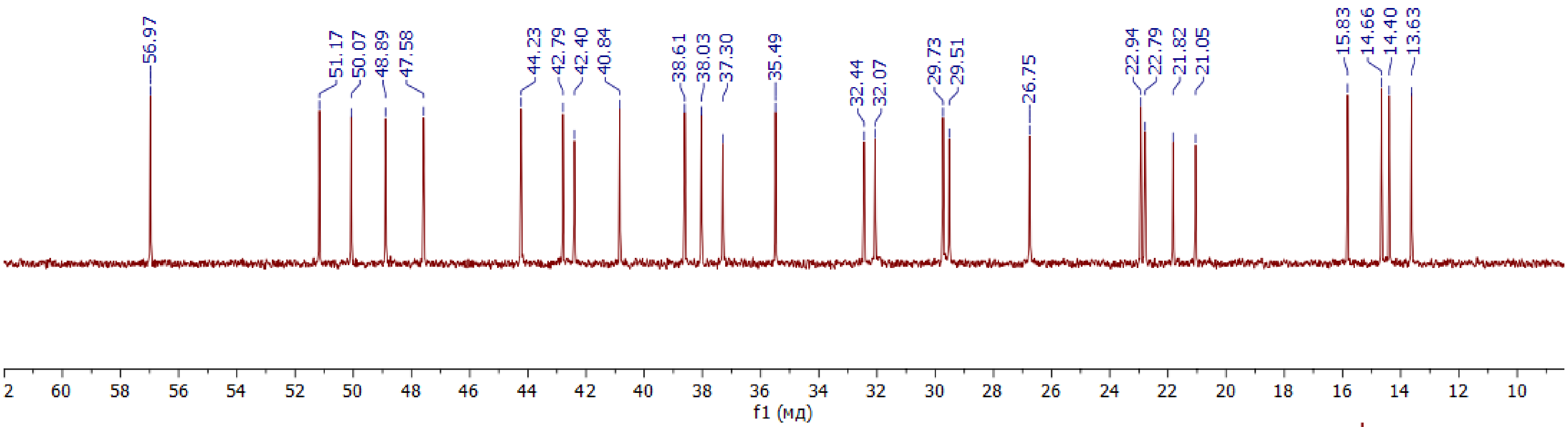

56.97
51.17
50.07
48.89
47.58
44.23
42.79
42.40
40.84
38.61
38.03
37.30
35.49
32.44
32.07
29.73
29.51
26.75
22.94
22.79
21.82
21.05
15.83
14.66
14.40
13.63
2 60 58 56 54 52 50 48 46 44 42 40 38 36 34 32 30 28 26 24 22 20 18 16 14 12 10
f1 (мд)

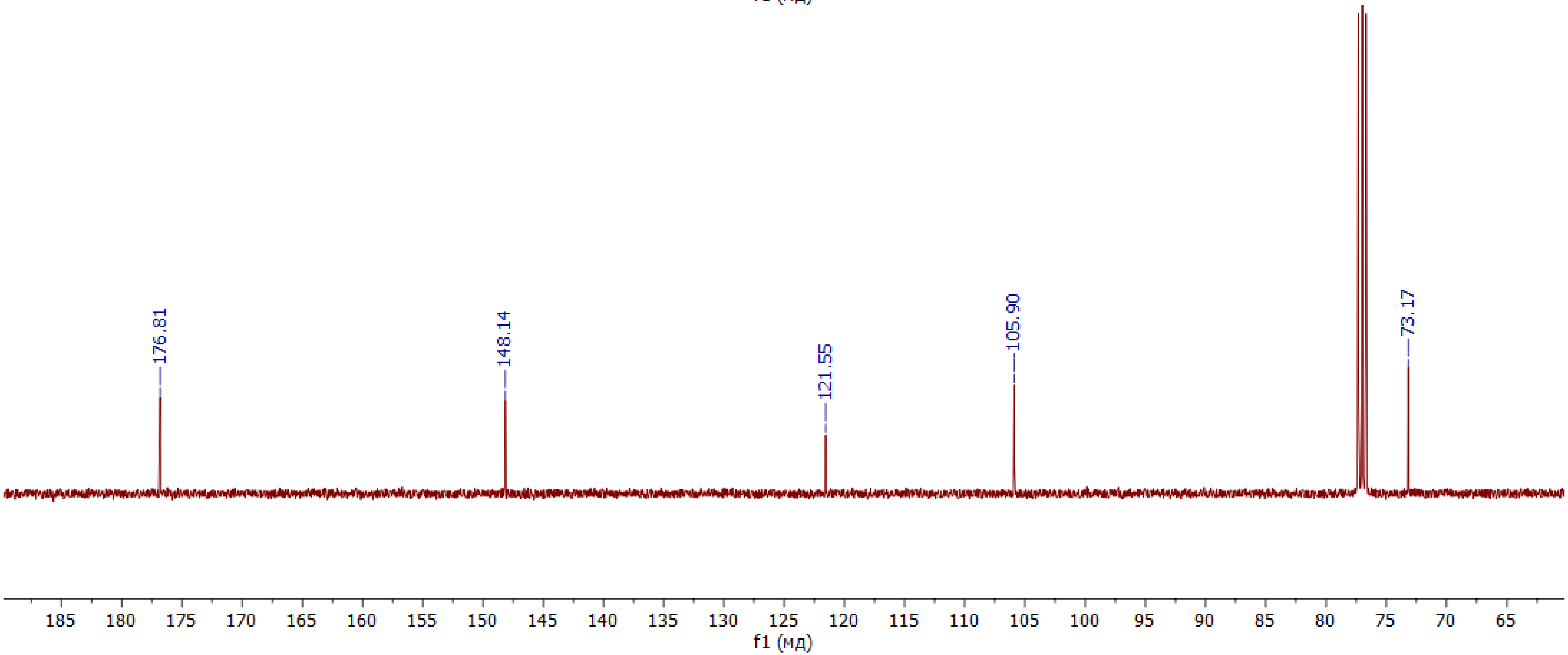

176.81
148.14
121.55
105.90
73.17
185 180 175 170 165 160 155 150 145 140 135 130 125 120 115 110 105 100 95 90 85 80 75 70 65
f1 (мд)

COSY NMR spectra of 2-Cyano-24-hydroxy-3-norlup-4(23)-en-28-oic acid methyl ester (11)

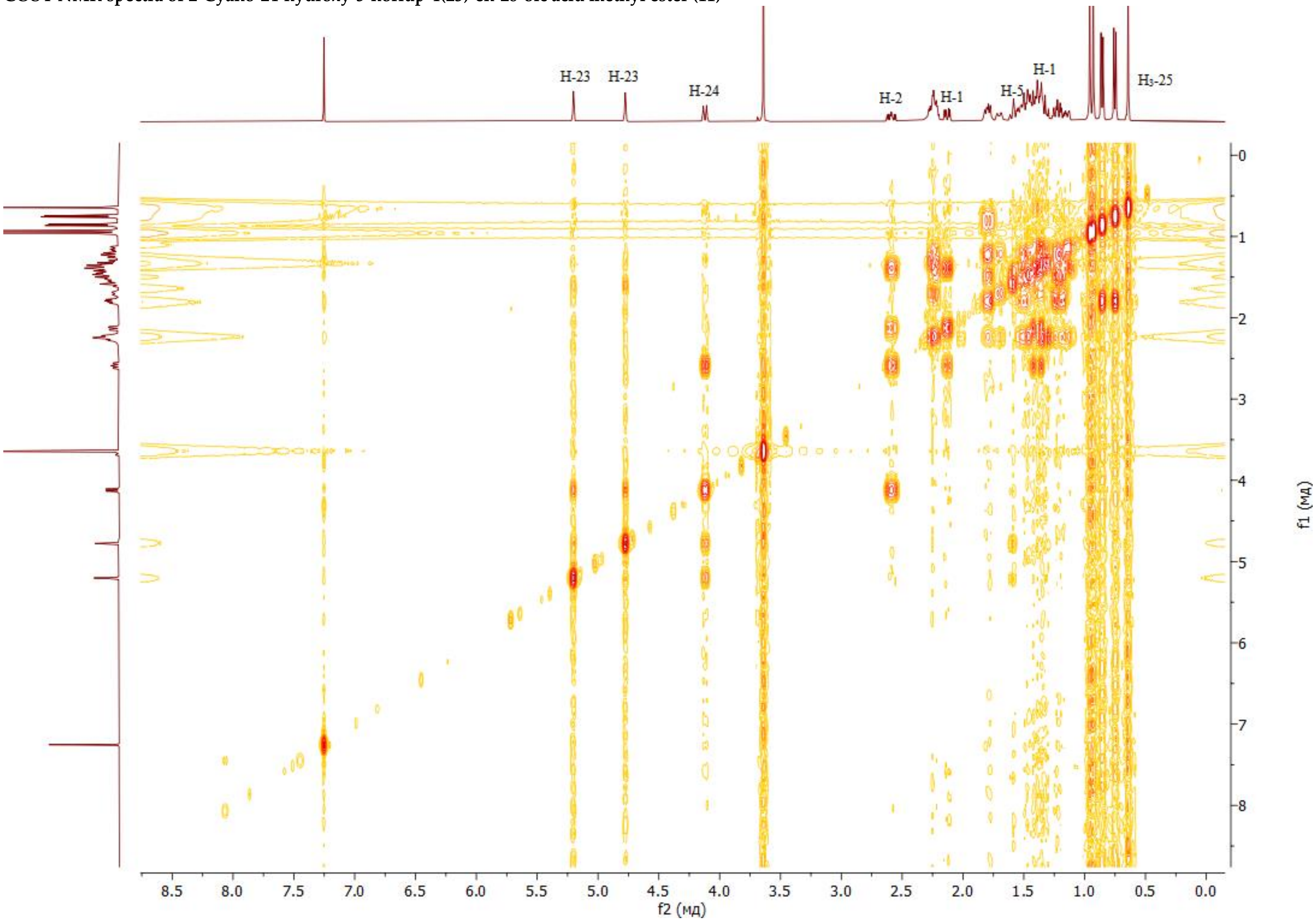

HSQC NMR spectra of 2-Cyano-24-hydroxy-3-norlup-4(23)-en-28-oic acid methyl ester (11)

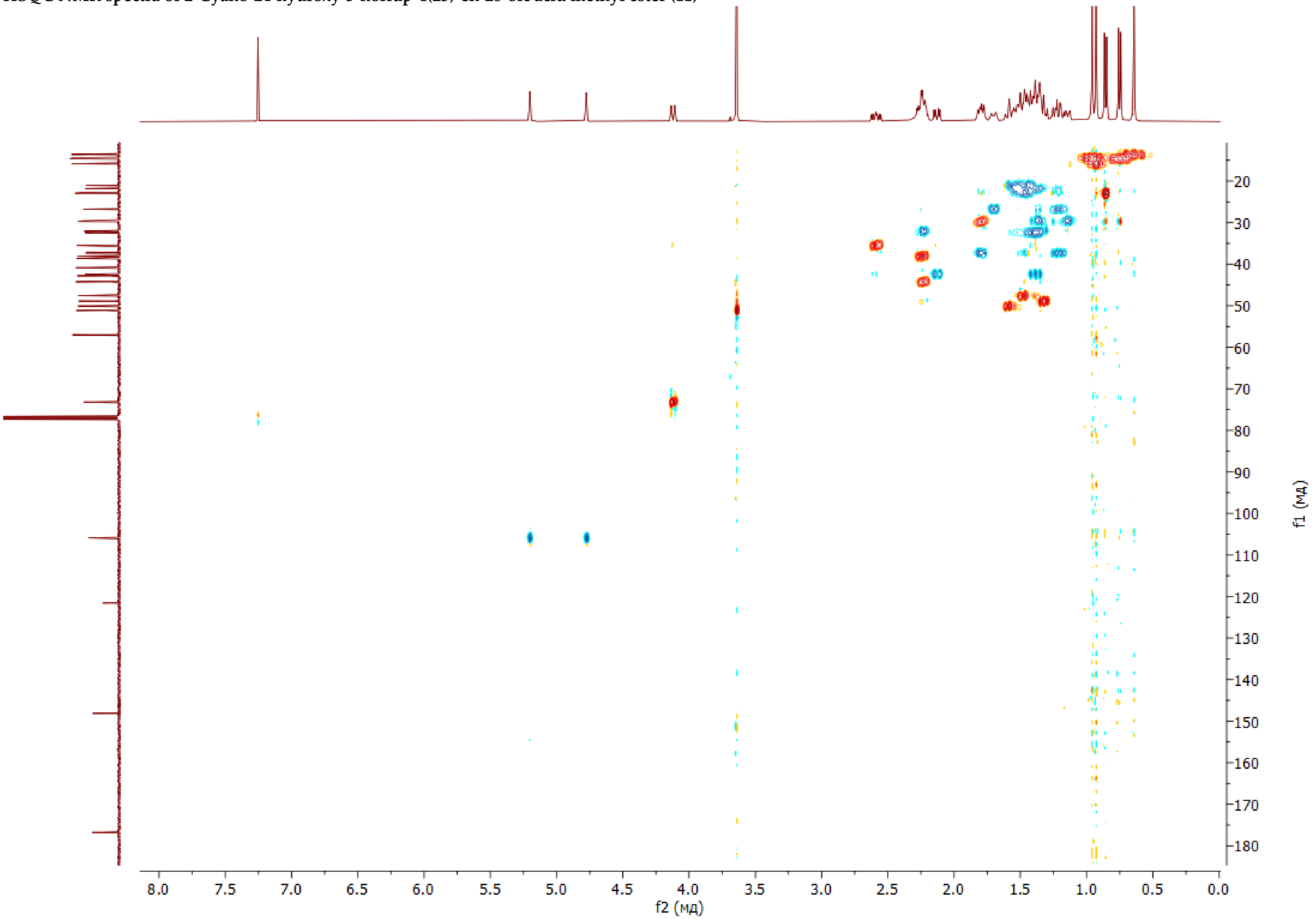

HSQC NMR spectra of 2-Cyano-24-hydroxy-3-norlup-4(23)-en-28-oic acid methyl ester (11)

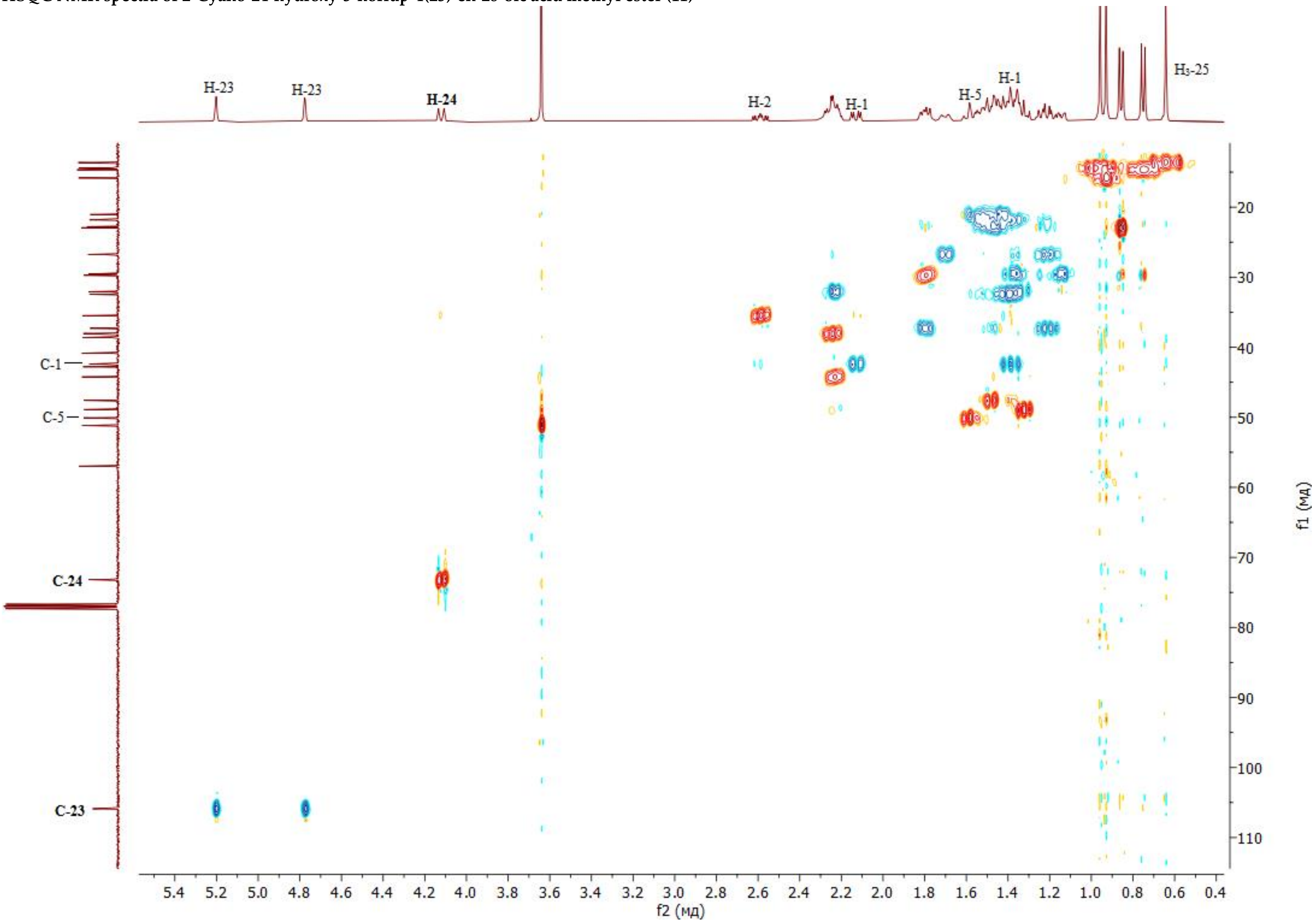

HMBC NMR spectra of 2-Cyano-24-hydroxy-3-norlup-4(23)-en-28-oic acid methyl ester (11)

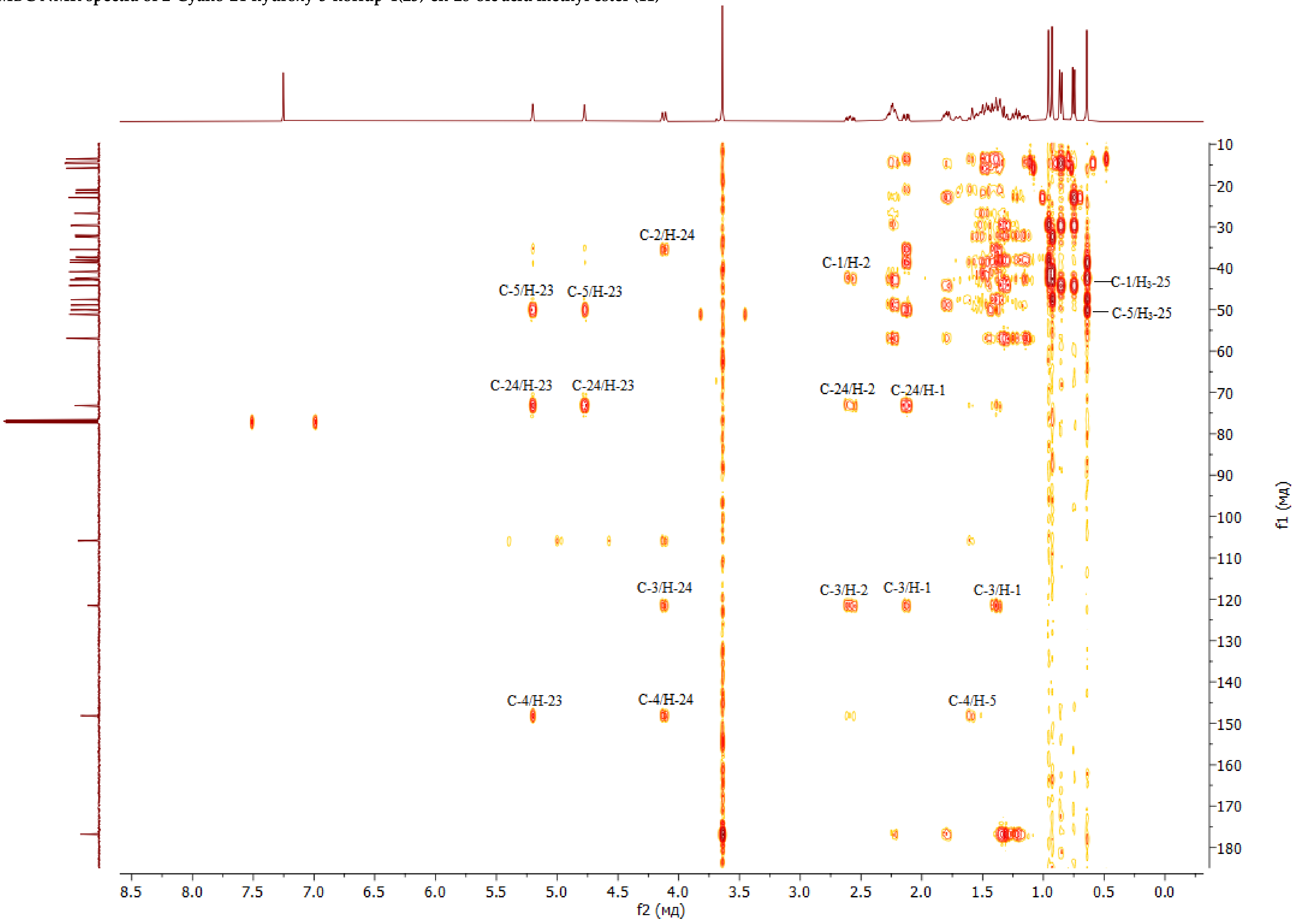

NOESY NMR spectra of 2-Cyano-24-hydroxy-3-norlup-4(23)-en-28-oic acid methyl ester (11)

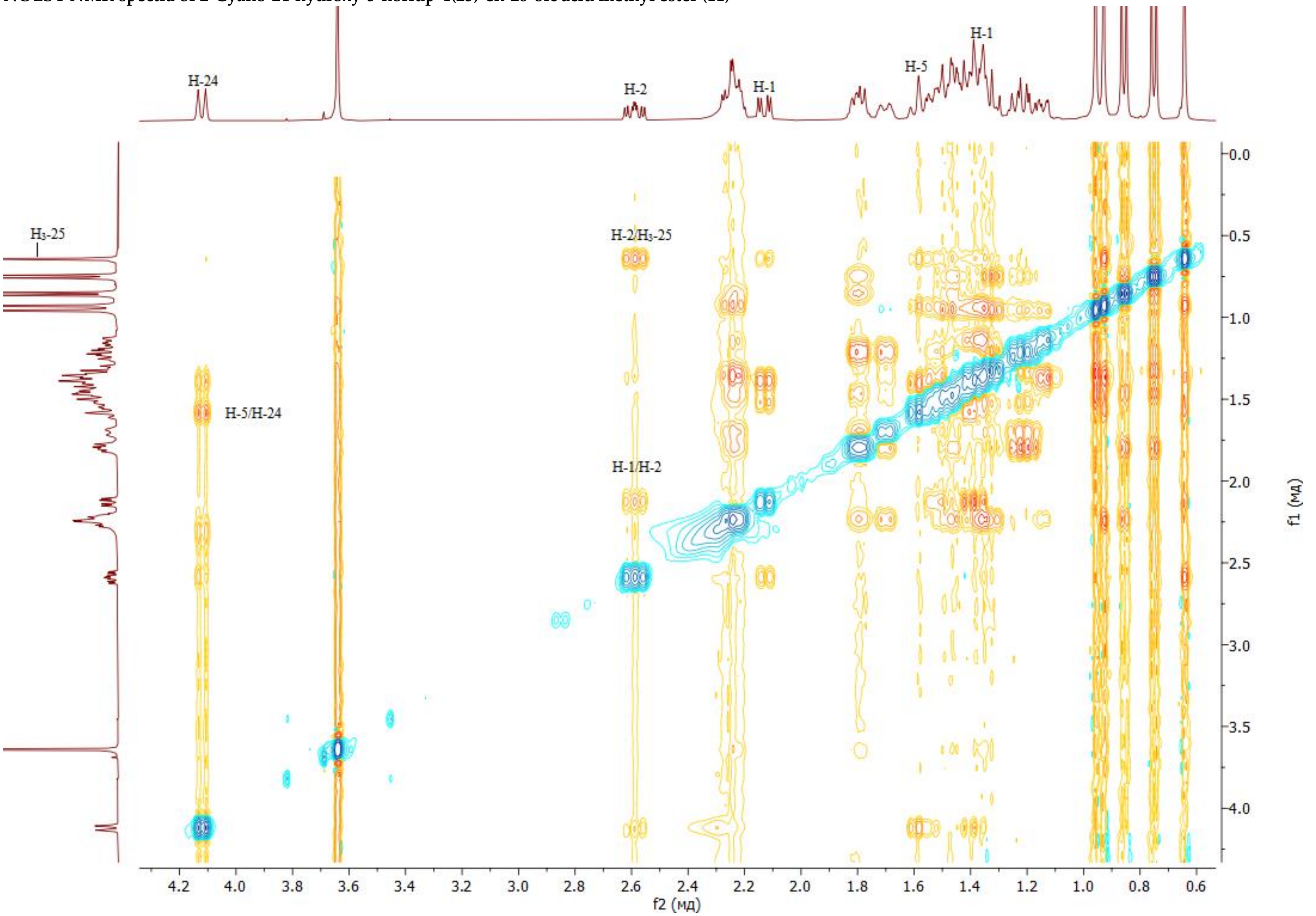

^{1}H NMR spectra of 2-Cyano-28-hydroxy-3-norlup-4(23)-ene (12).

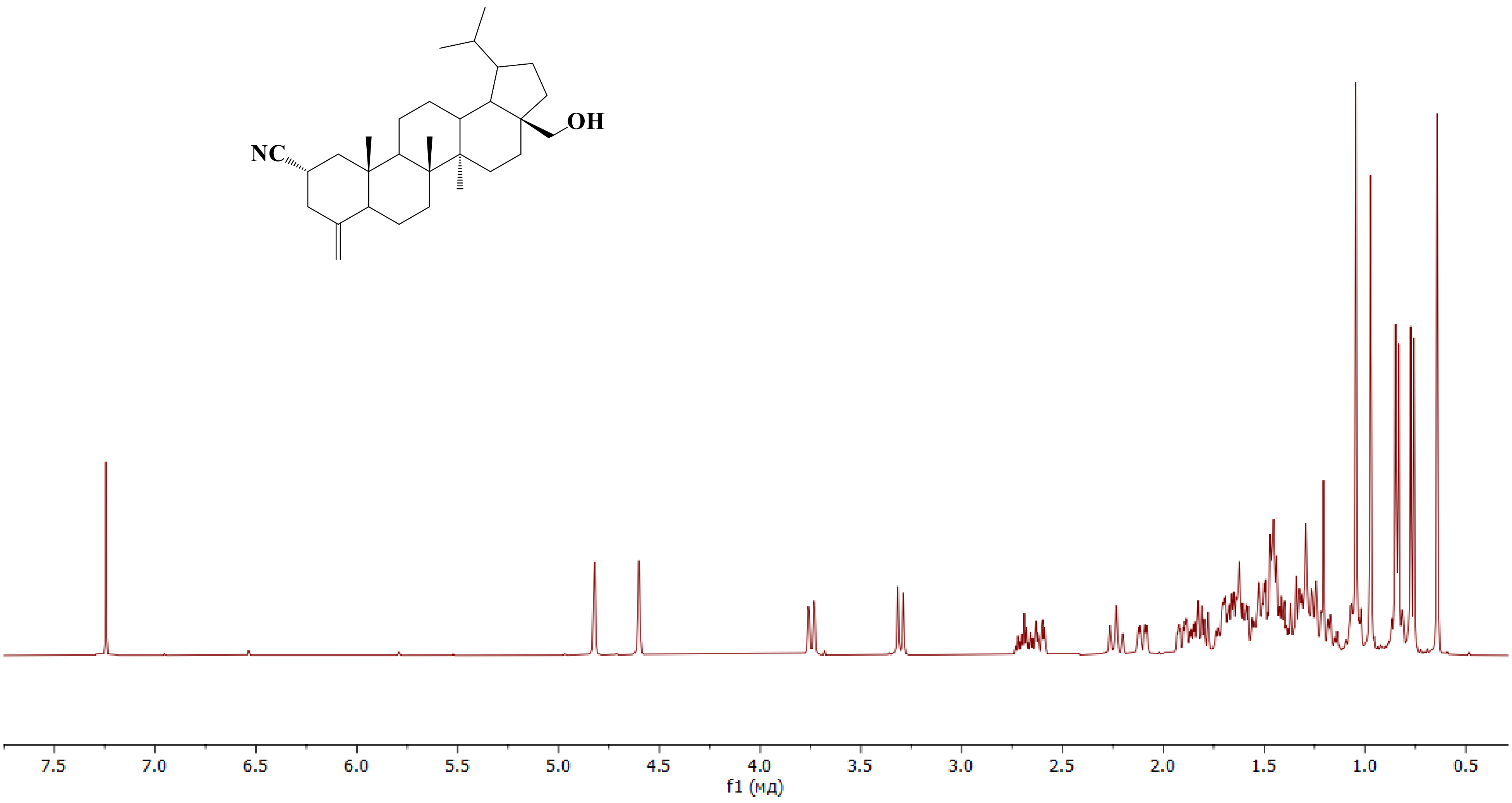

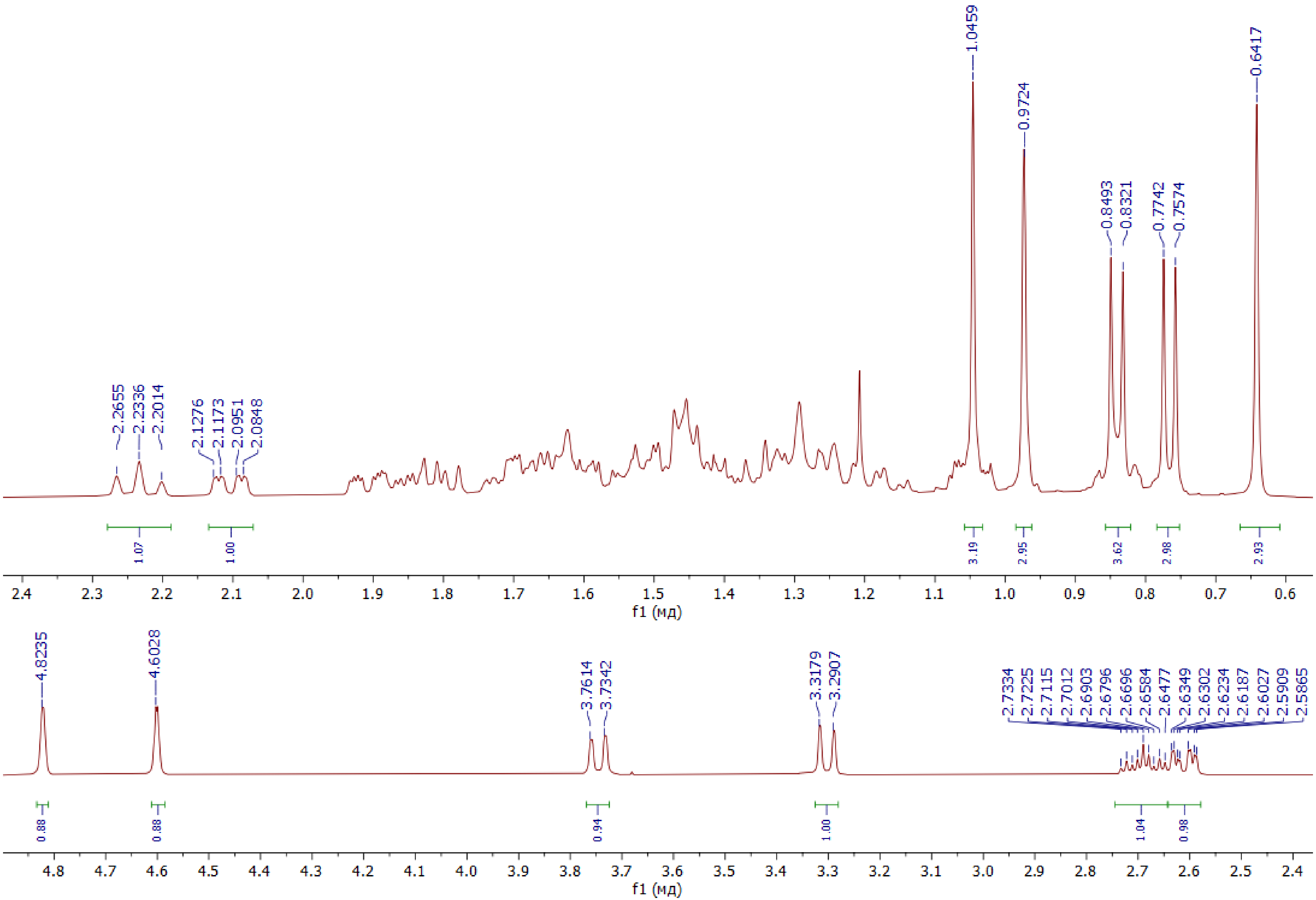
2.2655
2.2336
2.2014
2.1276
2.1173
2.0951
2.0848
1.0459
0.9724
0.8493
0.8321
0.7742
0.7574
0.6417
1.07
1.00
3.19
2.95
3.62
2.98
2.93
2.4 2.3 2.2 2.1 2.0 1.9 1.8 1.7 1.6 1.5 1.4 1.3 1.2 1.1 1.0 0.9 0.8 0.7 0.6
f1 (мд)
4.8235
4.6028
3.7614
3.7342
3.3179
3.2907
2.7334
2.7225
2.7115
2.7012
2.6903
2.6796
2.6696
2.6584
2.6477
2.6349
2.6302
2.6234
2.6187
2.6027
2.5909
2.5865
0.88
0.88
0.94
1.00
1.04
0.98
4.8 4.7 4.6 4.5 4.4 4.3 4.2 4.1 4.0 3.9 3.8 3.7 3.6 3.5 3.4 3.3 3.2 3.1 3.0 2.9 2.8 2.7 2.6 2.5 2.4
f1 (мд)

^{13}C NMR spectra of 2-Cyano-28-hydroxy-3-norlup-4(23)-ene (12).

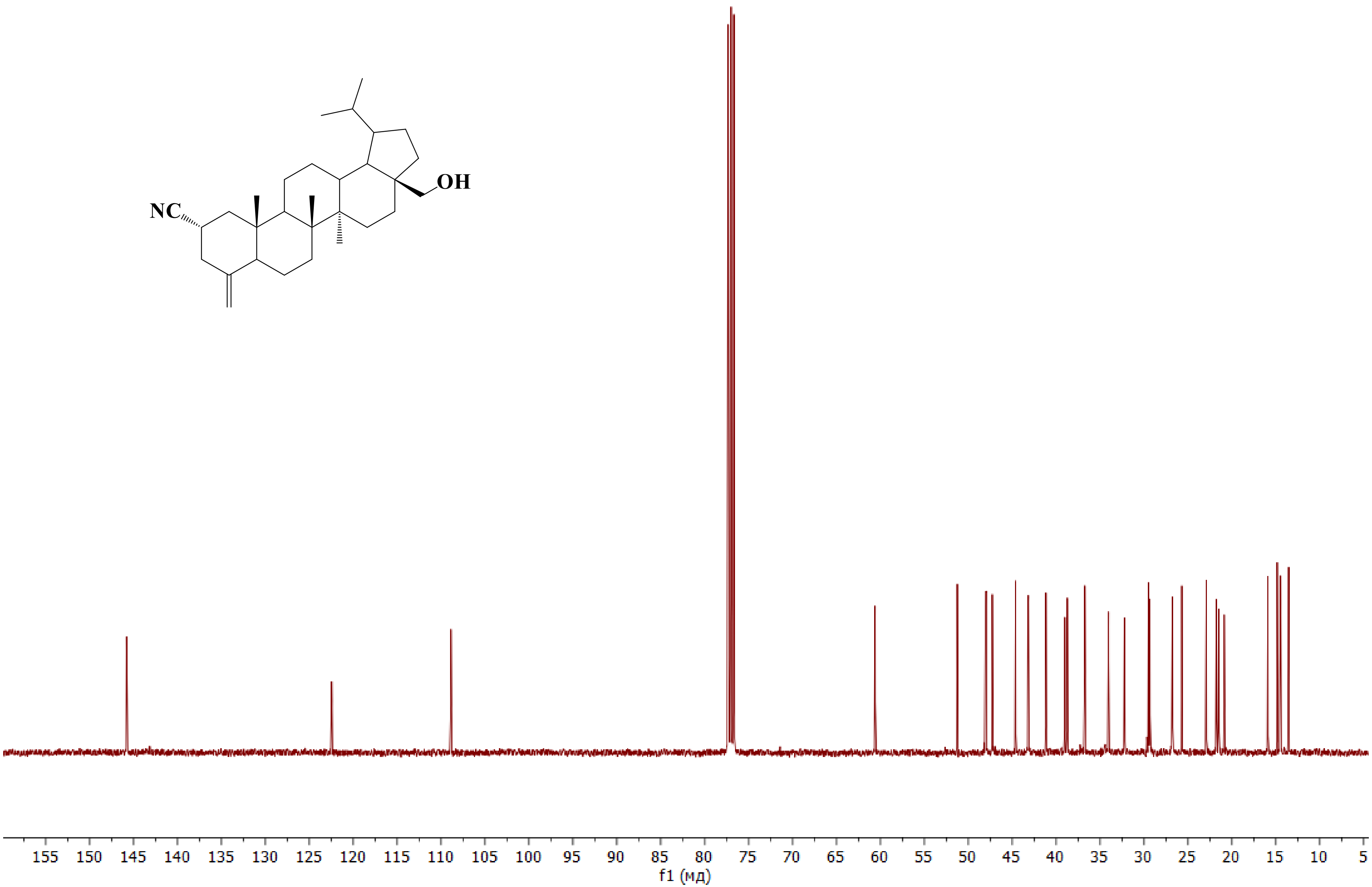

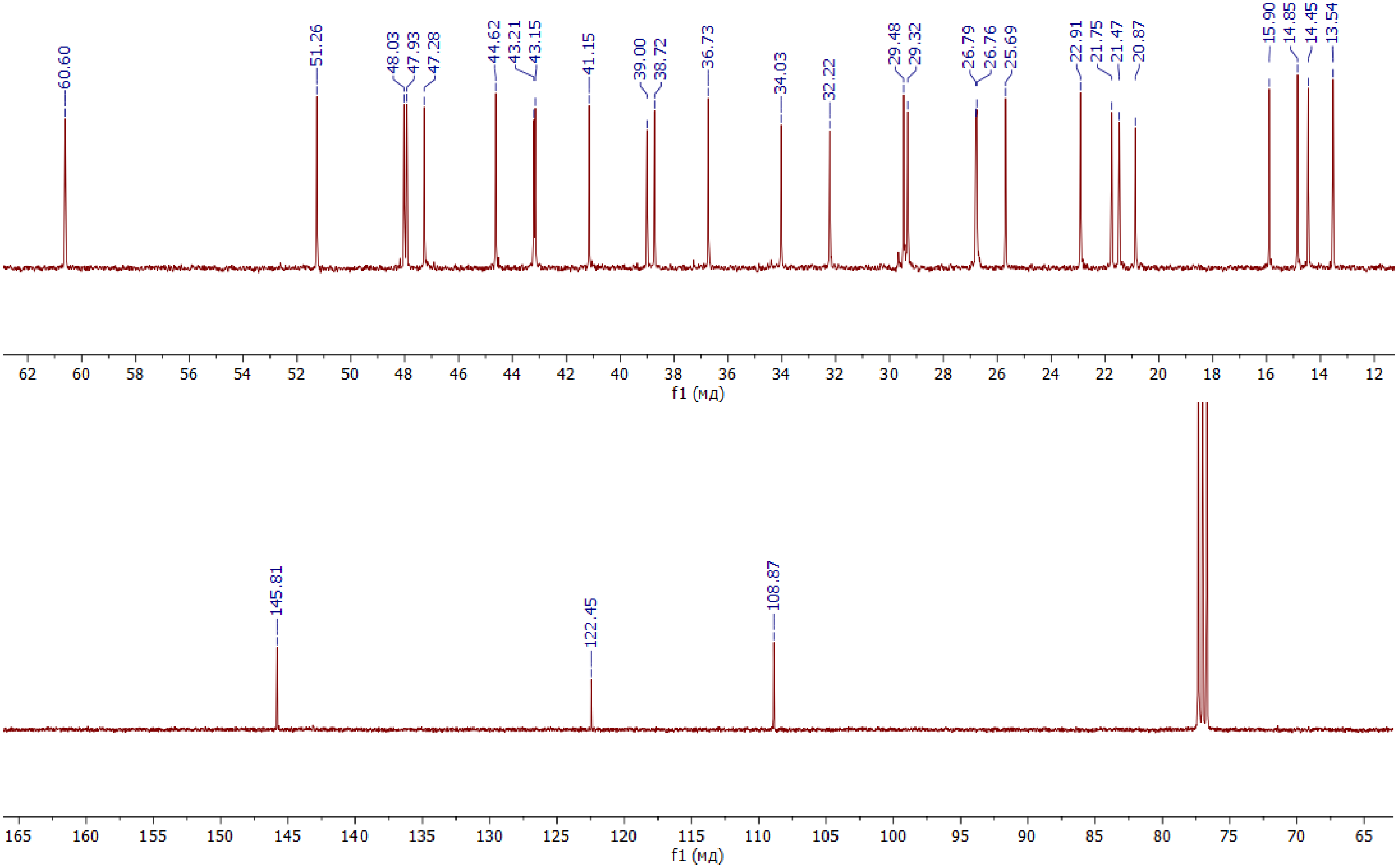
60.60
51.26
48.03
47.93
47.28
44.62
43.21
43.15
41.15
39.00
38.72
36.73
34.03
32.22
29.48
29.32
26.79
26.76
25.69
22.91
21.75
21.47
20.87
15.90
14.85
14.45
13.54
62 60 58 56 54 52 50 48 46 44 42 40 38 36 34 32 30 28 26 24 22 20 18 16 14 12
f1 (мд)
145.81
122.45
108.87
165 160 155 150 145 140 135 130 125 120 115 110 105 100 95 90 85 80 75 70 65
f1 (мд)

^{1}H NMR spectra of 2-Cyano-3-norlup-4(23)-ene-28-oic acid methyl ester (13).

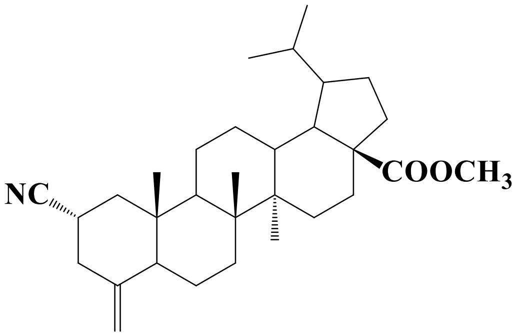

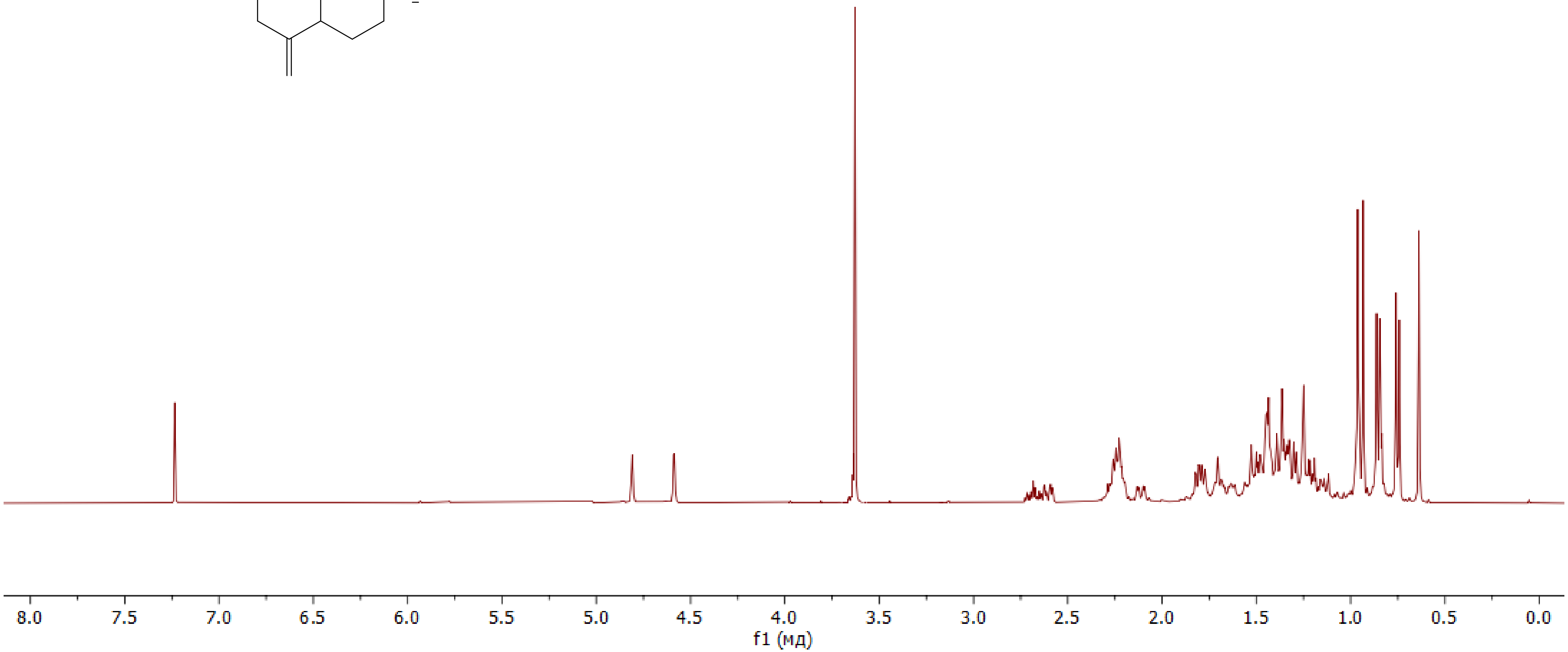

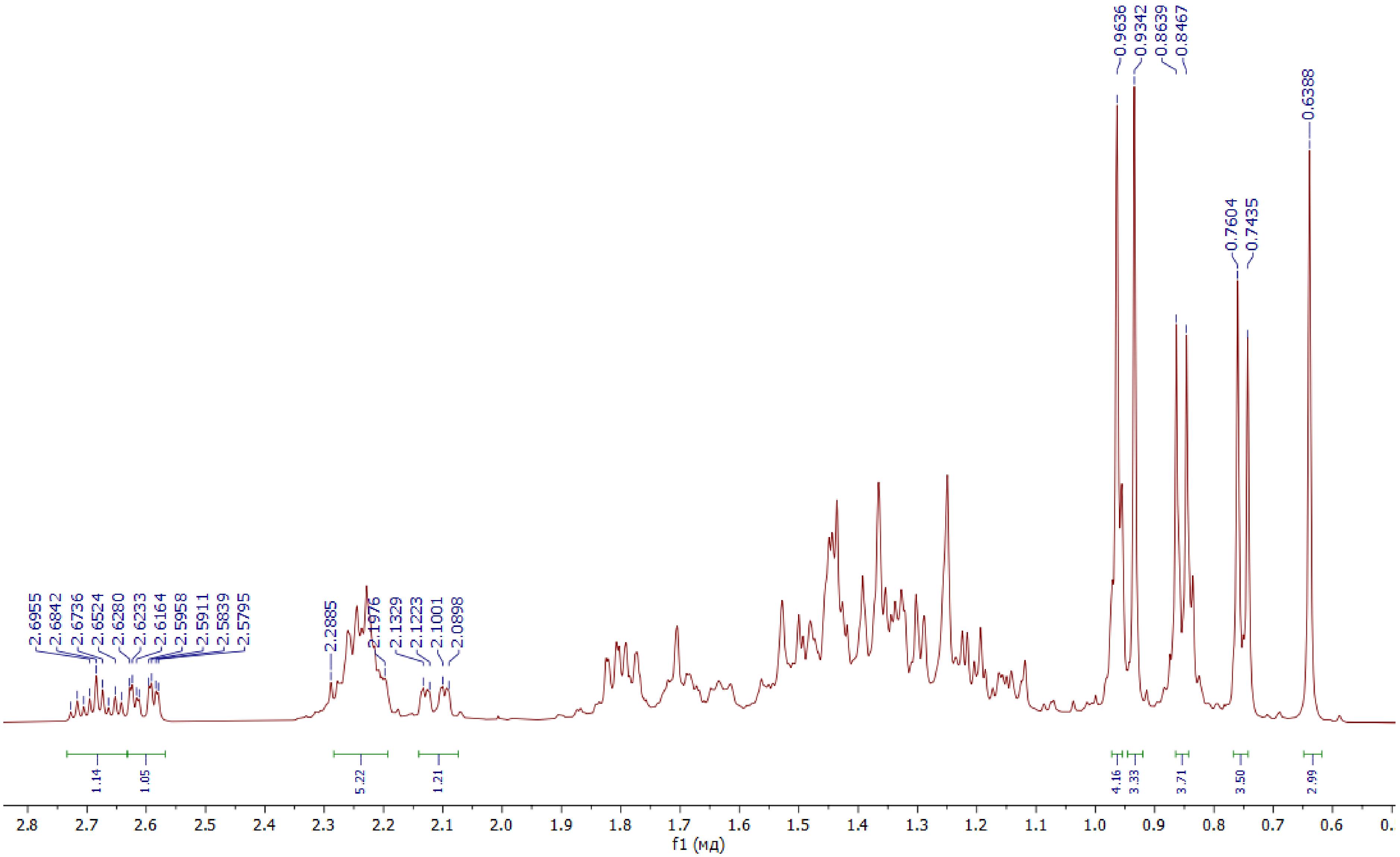

2.6955
2.6842
2.6736
2.6524
2.6280
2.6233
2.6164
2.5958
2.5911
2.5839
2.5795
2.2885
2.1976
2.1329
2.1223
2.1001
2.0898
0.9636
0.9342
0.8639
0.8467
0.7604
0.7435
0.6388
1.14
1.05
5.22
1.21
4.16
3.33
3.71
3.50
2.99
2.8
2.7
2.6
2.5
2.4
2.3
2.2
2.1
2.0
1.9
1.8
1.7
1.6
1.5
1.4
1.3
1.2
1.1
1.0
0.9
0.8
0.7
0.6
0.
f1 (мд)

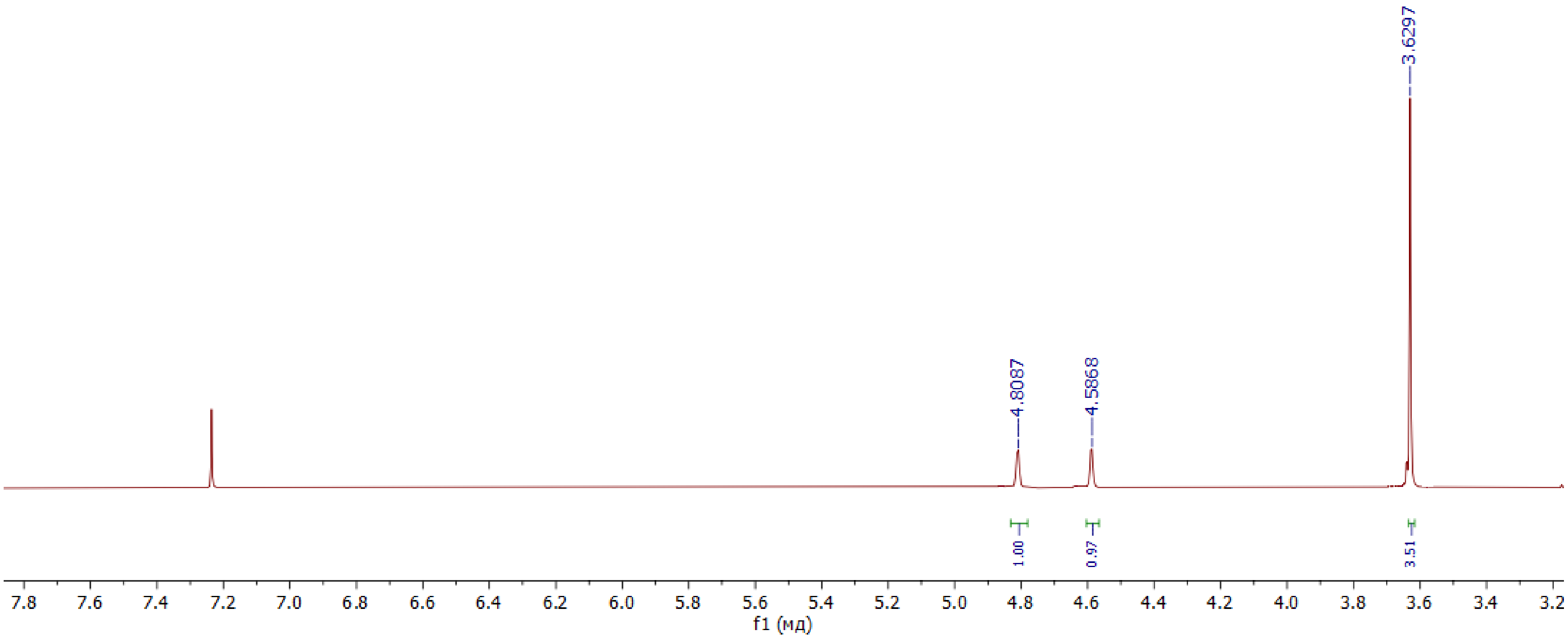
3.6297
4.8087
4.5868
1.00
0.97
3.51
7.8 7.6 7.4 7.2 7.0 6.8 6.6 6.4 6.2 6.0 5.8 5.6 5.4 5.2 5.0 4.8 4.6 4.4 4.2 4.0 3.8 3.6 3.4 3.2
f1 (мд)

^{13}C NMR spectra of 2-Cyano-3-norlup-4(23)-ene-28-oic acid methyl ester (13).

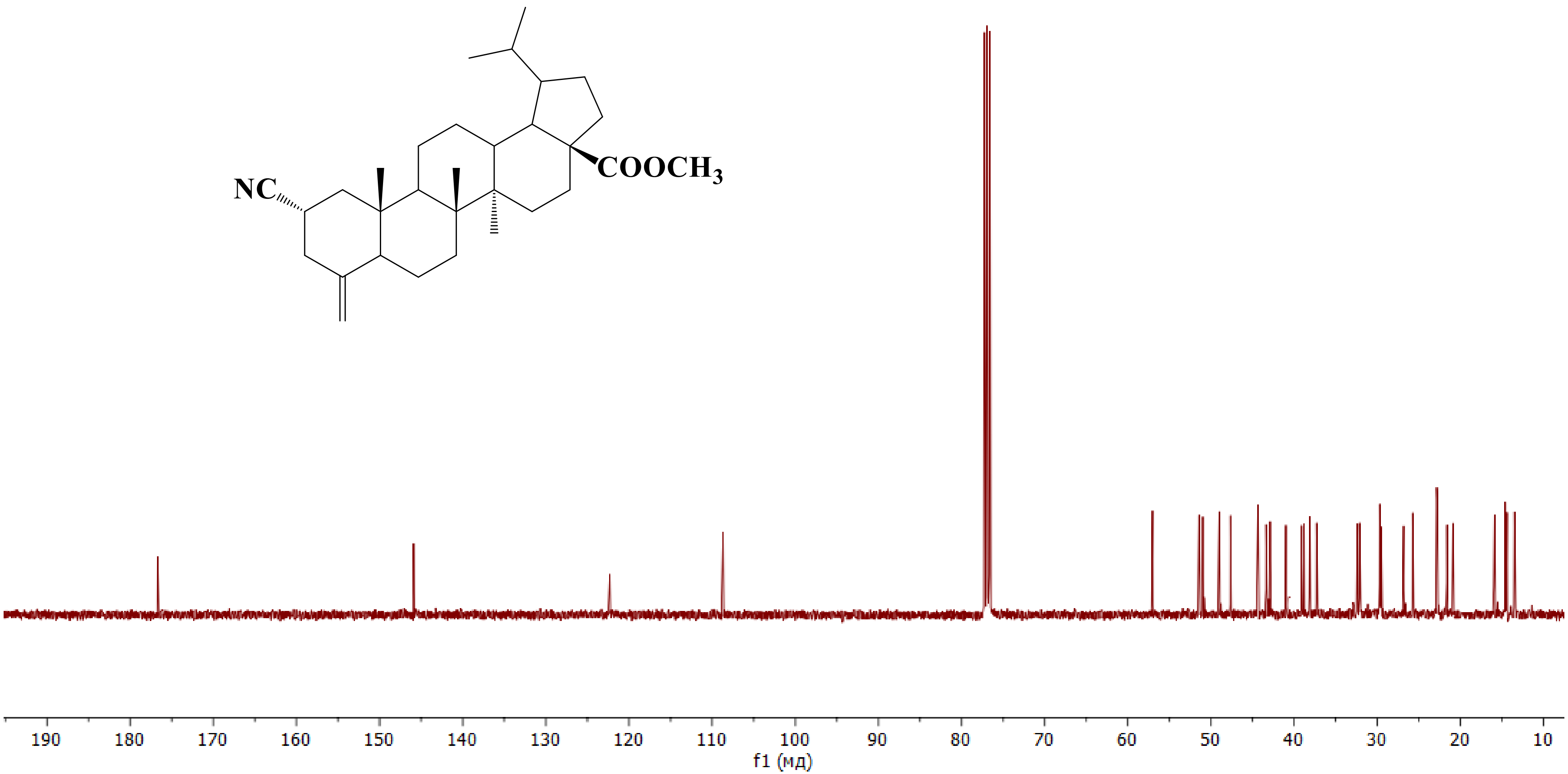

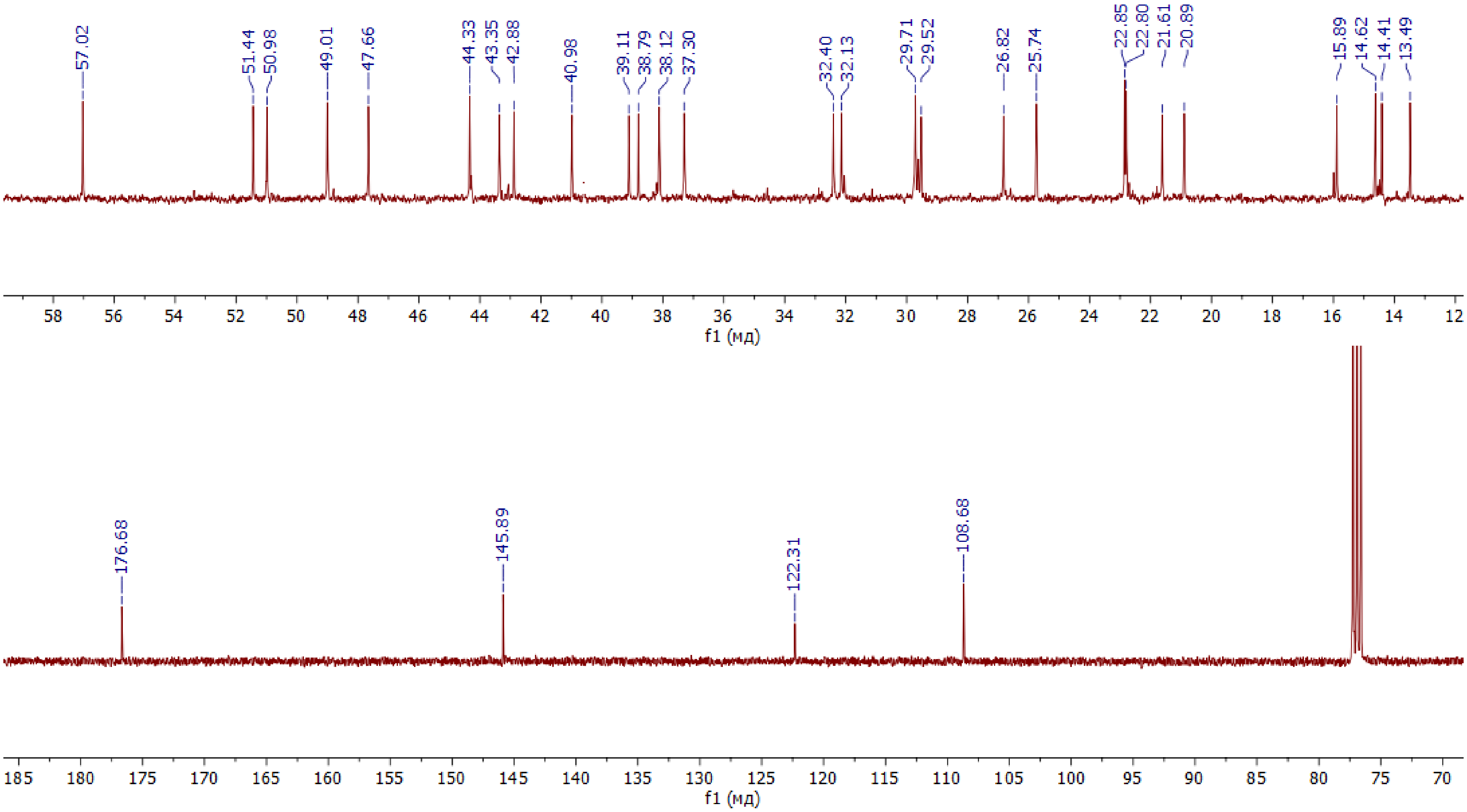
57.02
51.44
50.98
49.01
47.66
44.33
43.35
42.88
40.98
39.11
38.79
38.12
37.30
32.40
32.13
29.71
29.52
26.82
25.74
22.85
22.80
21.61
20.89
15.89
14.62
14.41
13.49
f1 (мд)
176.68
145.89
122.31
108.68
f1 (мд)

^{1}H NMR spectra of 2-Cyano-28-hydroxy-4-oxo-3,23-dinorlup-2(24)-ene (14).

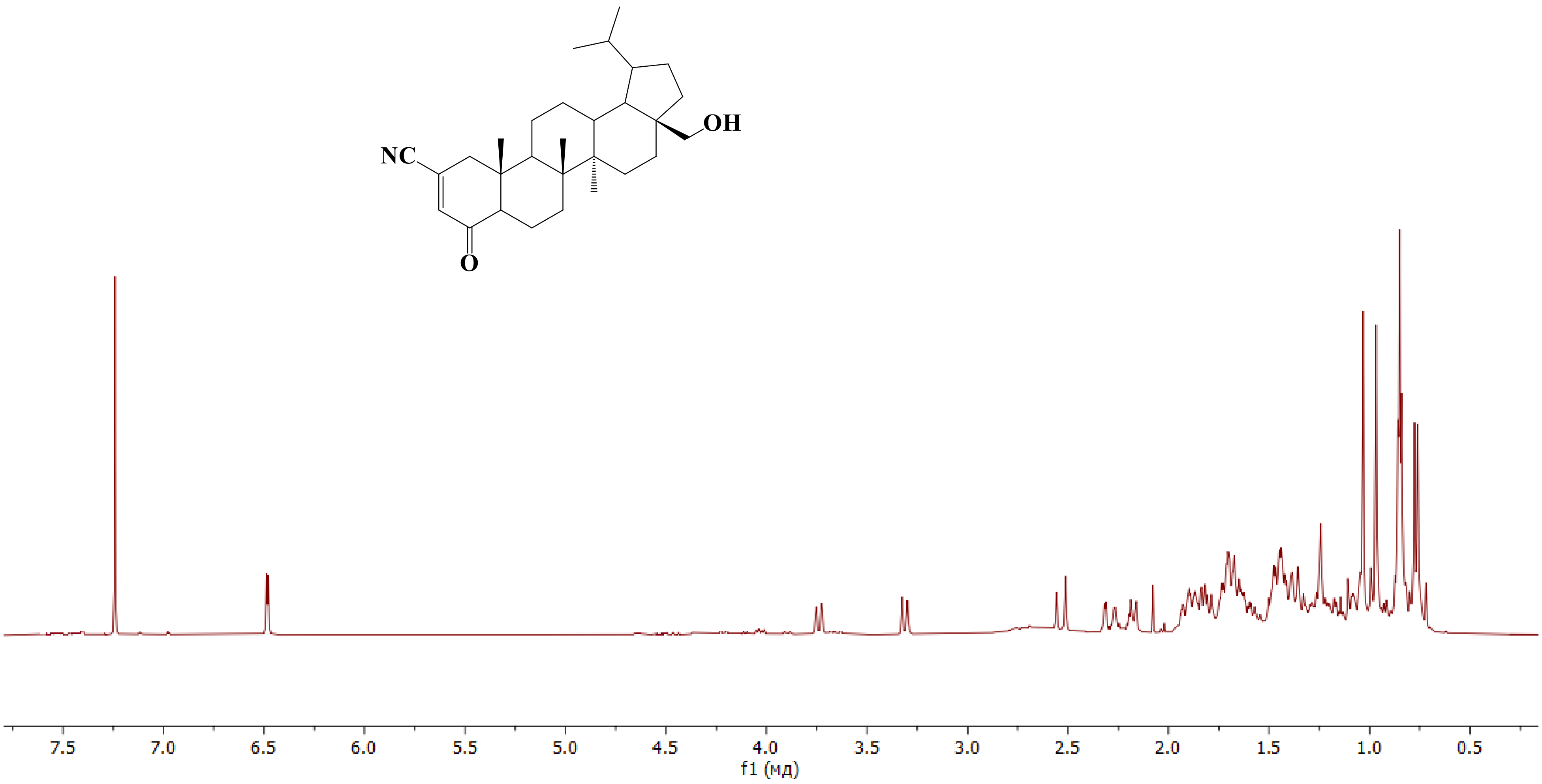

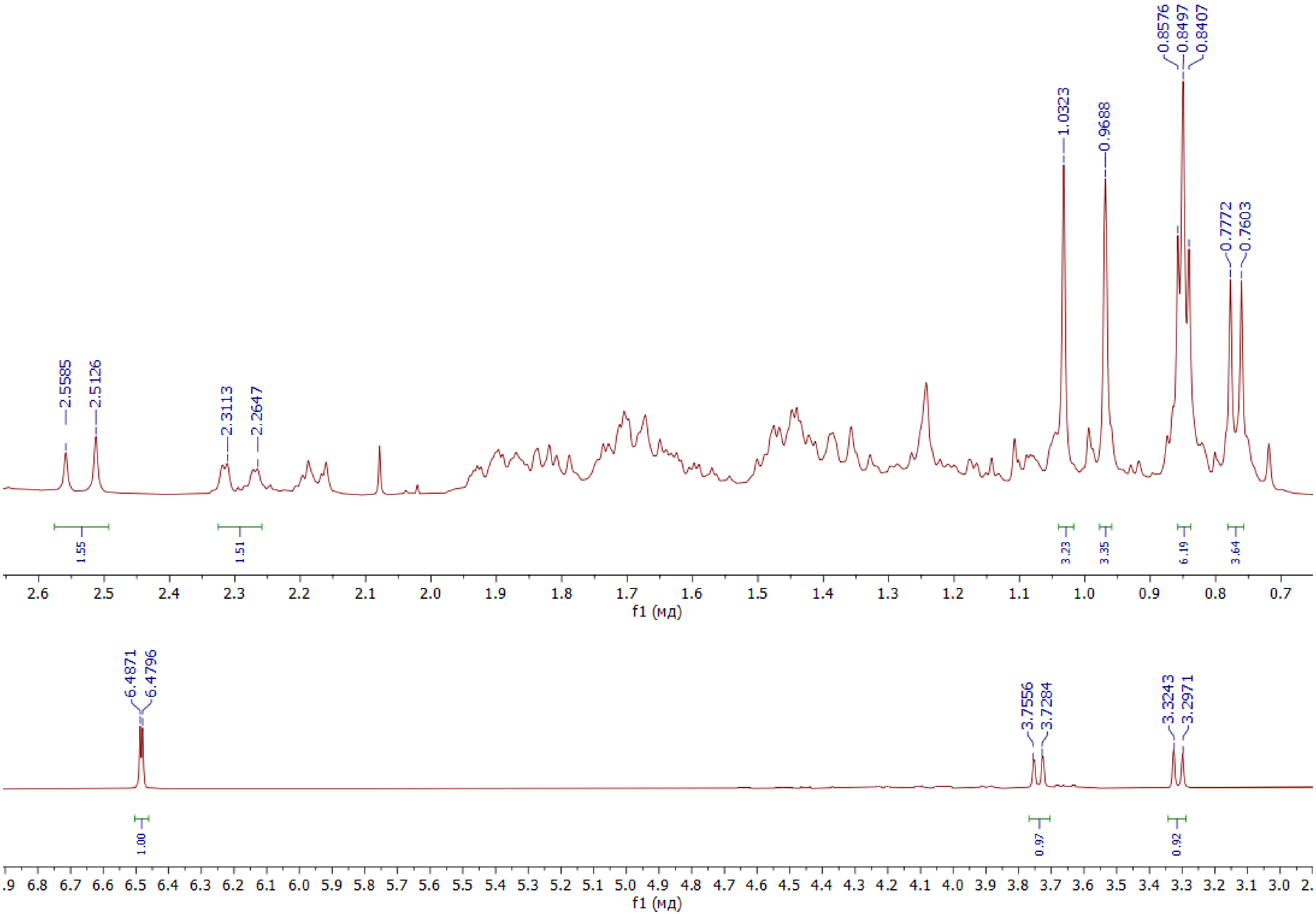
2.5585
2.5126
2.3113
2.2647
1.0323
0.9688
0.8576
0.8497
0.8407
0.7772
0.7603
1.55
1.51
3.23
3.35
6.19
3.64
2.6 2.5 2.4 2.3 2.2 2.1 2.0 1.9 1.8 1.7 1.6 1.5 1.4 1.3 1.2 1.1 1.0 0.9 0.8 0.7
f1 (мд)
6.4871
6.4796
3.7556
3.7284
3.3243
3.2971
1.00
0.97
0.92
.9 6.8 6.7 6.6 6.5 6.4 6.3 6.2 6.1 6.0 5.9 5.8 5.7 5.6 5.5 5.4 5.3 5.2 5.1 5.0 4.9 4.8 4.7 4.6 4.5 4.4 4.3 4.2 4.1 4.0 3.9 3.8 3.7 3.6 3.5 3.4 3.3 3.2 3.1 3.0 2.
f1 (мд)

^{13}C NMR spectra of 2-Cyano-28-hydroxy-4-oxo-3,23-dinorlup-2(24)-ene (14).

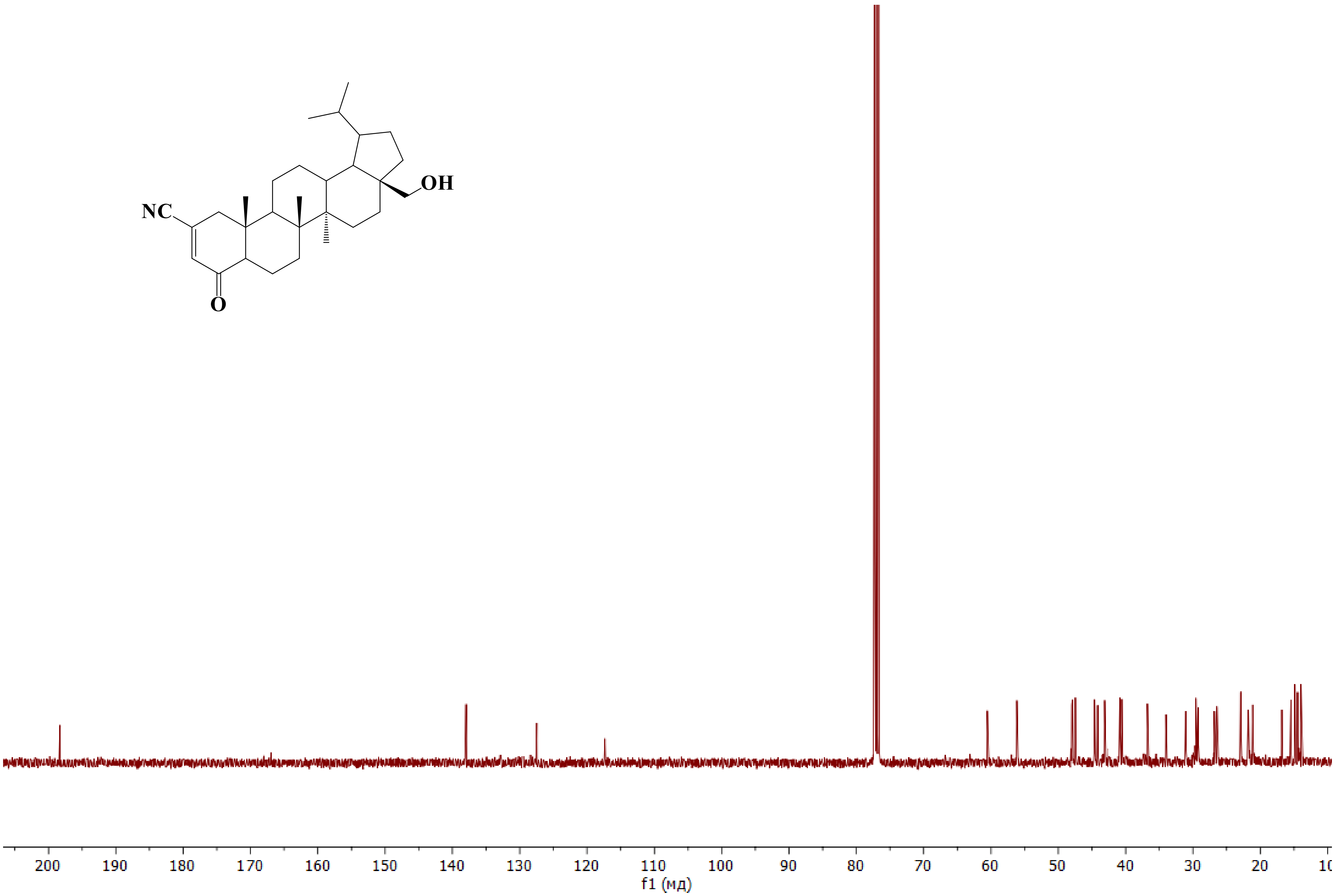

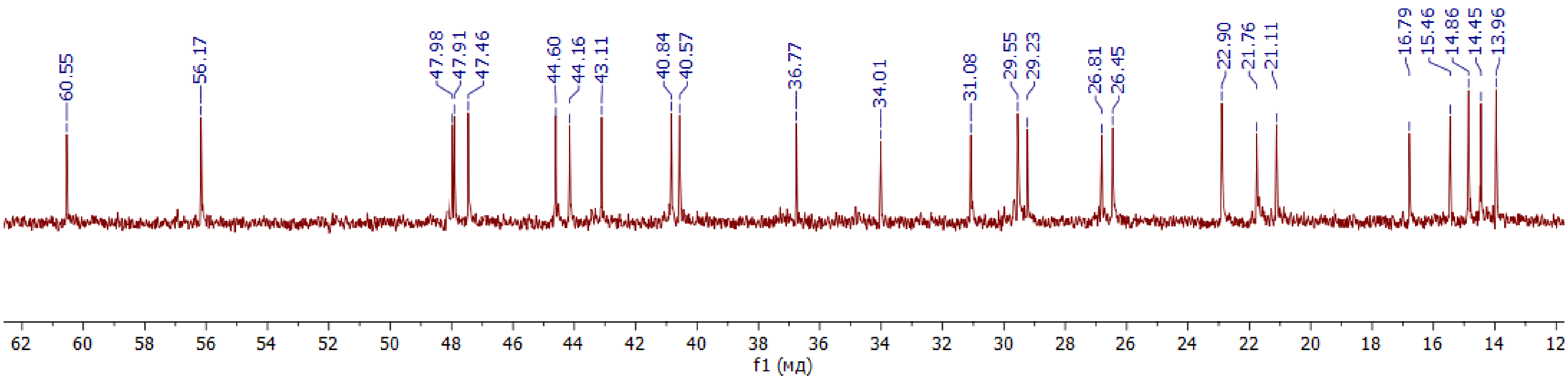
60.55
56.17
47.98
47.91
47.46
44.60
44.16
43.11
40.84
40.57
36.77
34.01
31.08
29.55
29.23
26.81
26.45
22.90
21.76
21.11
16.79
15.46
14.86
14.45
13.96
62 60 58 56 54 52 50 48 46 44 42 40 38 36 34 32 30 28 26 24 22 20 18 16 14 12
f1 (мд)

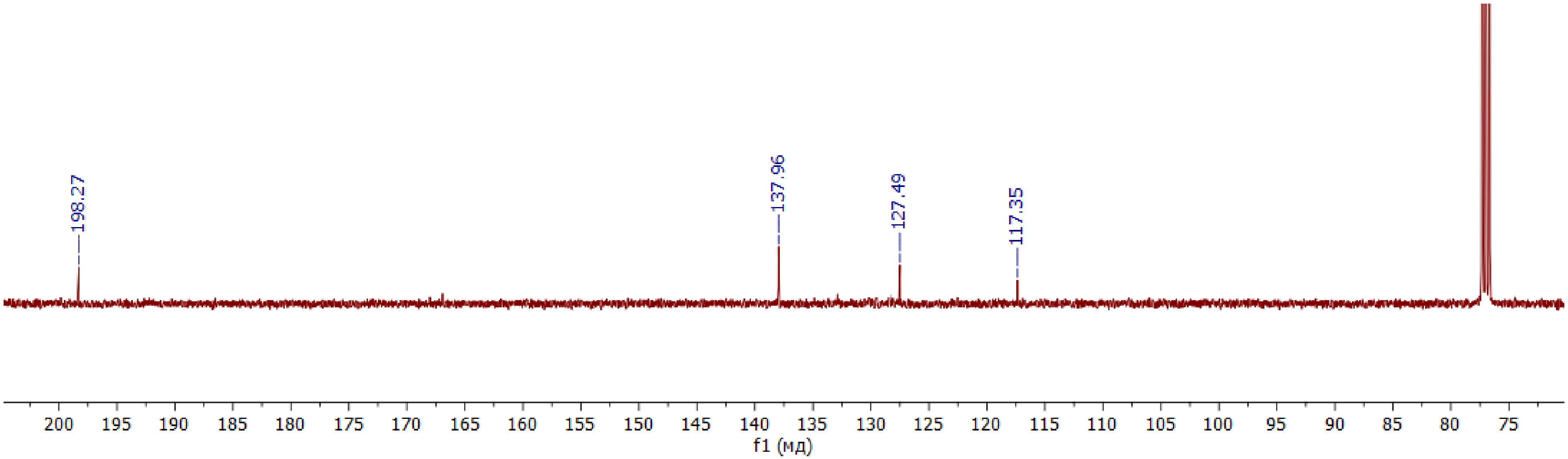
198.27
137.96
127.49
117.35
200 195 190 185 180 175 170 165 160 155 150 145 140 135 130 125 120 115 110 105 100 95 90 85 80 75
f1 (мд)

¹H NMR spectra of 2-Cyano-4-oxo-3,23-dinorlup-2(24)-en-28-oic acid methyl ester (15).

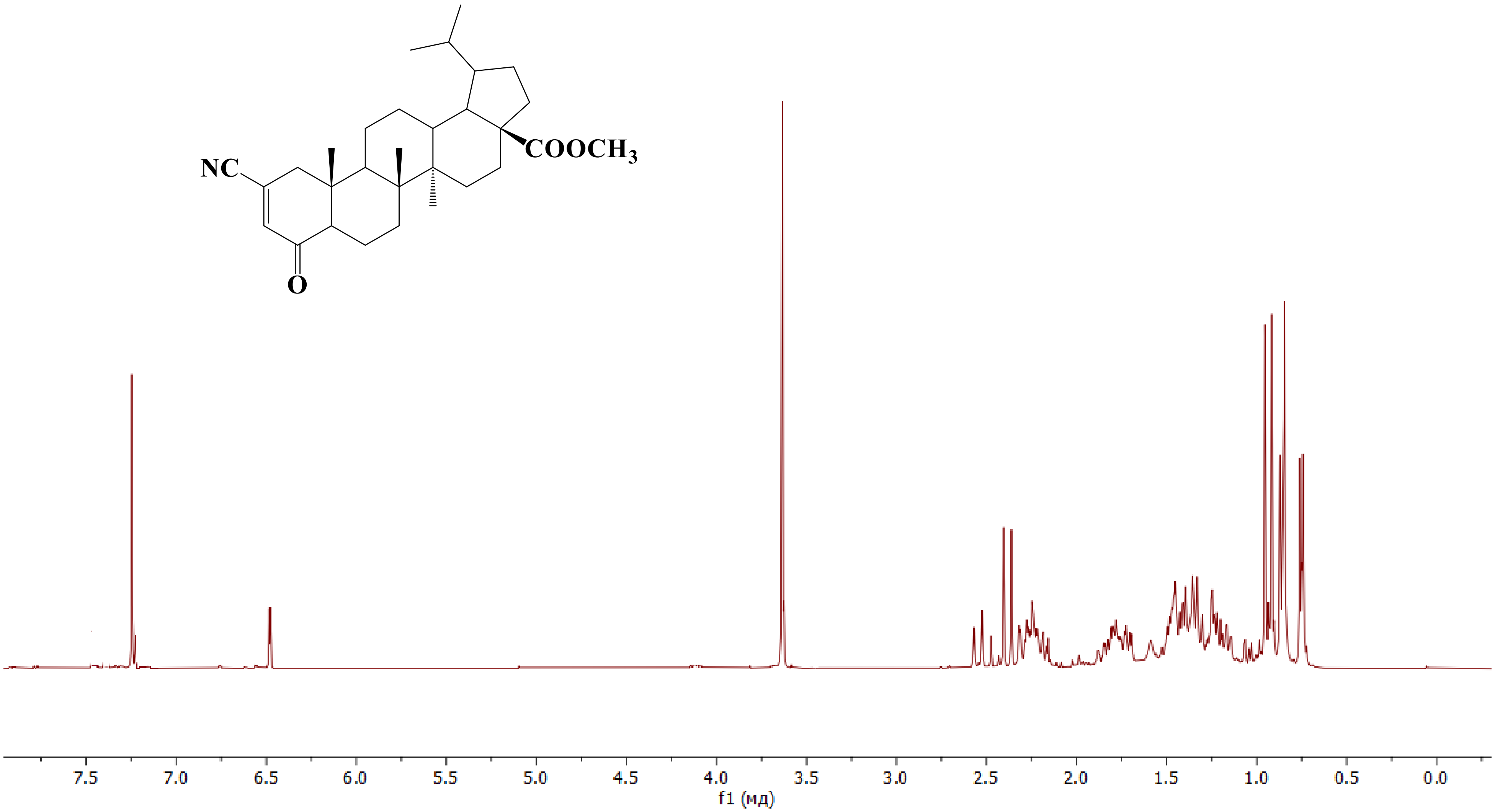

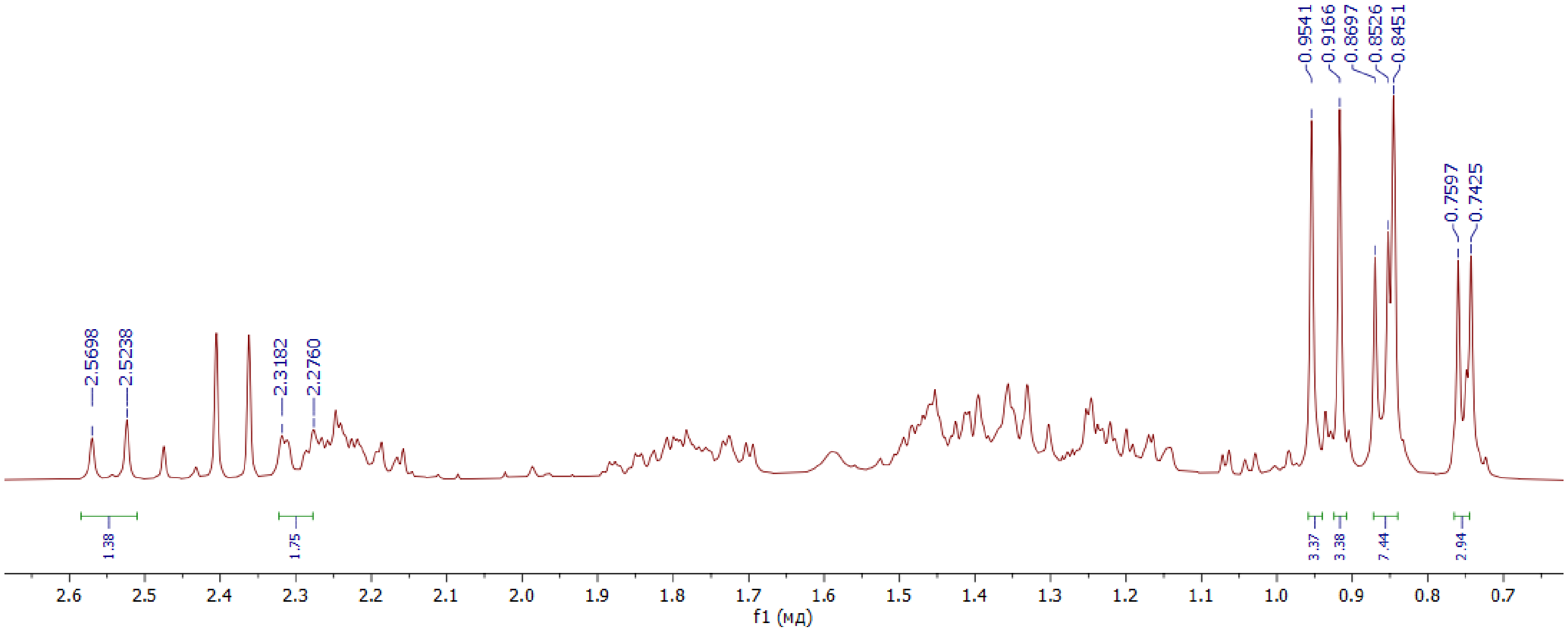

2.5698
2.5238
2.3182
2.2760
0.9541
0.9166
0.8697
0.8526
0.8451
0.7597
0.7425
1.38
1.75
3.37
3.38
7.44
2.94
2.6 2.5 2.4 2.3 2.2 2.1 2.0 1.9 1.8 1.7 1.6 1.5 1.4 1.3 1.2 1.1 1.0 0.9 0.8 0.7
f1 (мд)

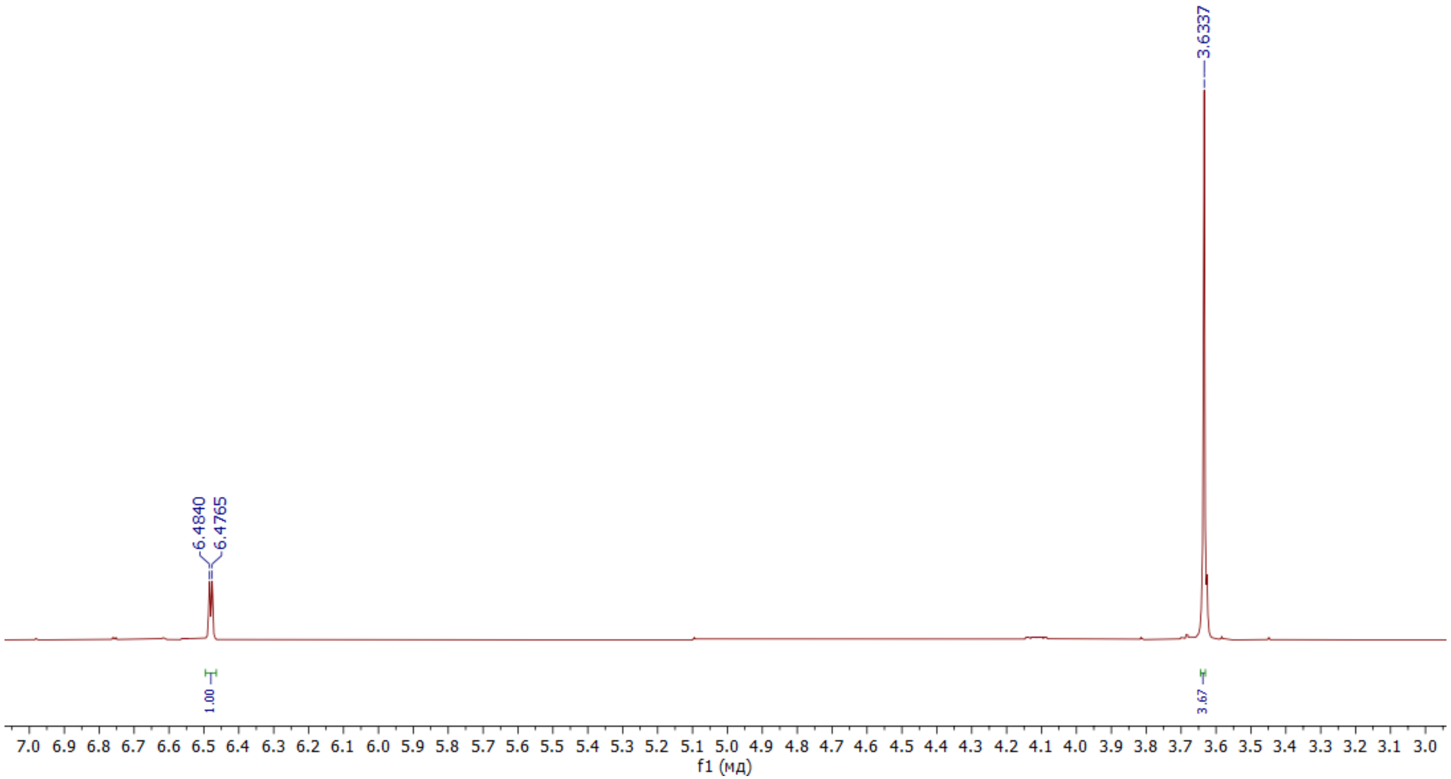

3.6337
6.4840
6.4765
1.00
3.67
7.0 6.9 6.8 6.7 6.6 6.5 6.4 6.3 6.2 6.1 6.0 5.9 5.8 5.7 5.6 5.5 5.4 5.3 5.2 5.1 5.0 4.9 4.8 4.7 4.6 4.5 4.4 4.3 4.2 4.1 4.0 3.9 3.8 3.7 3.6 3.5 3.4 3.3 3.2 3.1 3.0
f1 (мд)

^{13}C NMR spectra of 2-Cyano-4-oxo-3,23-dinorlup-2(24)-en-28-oic acid methyl ester (15).

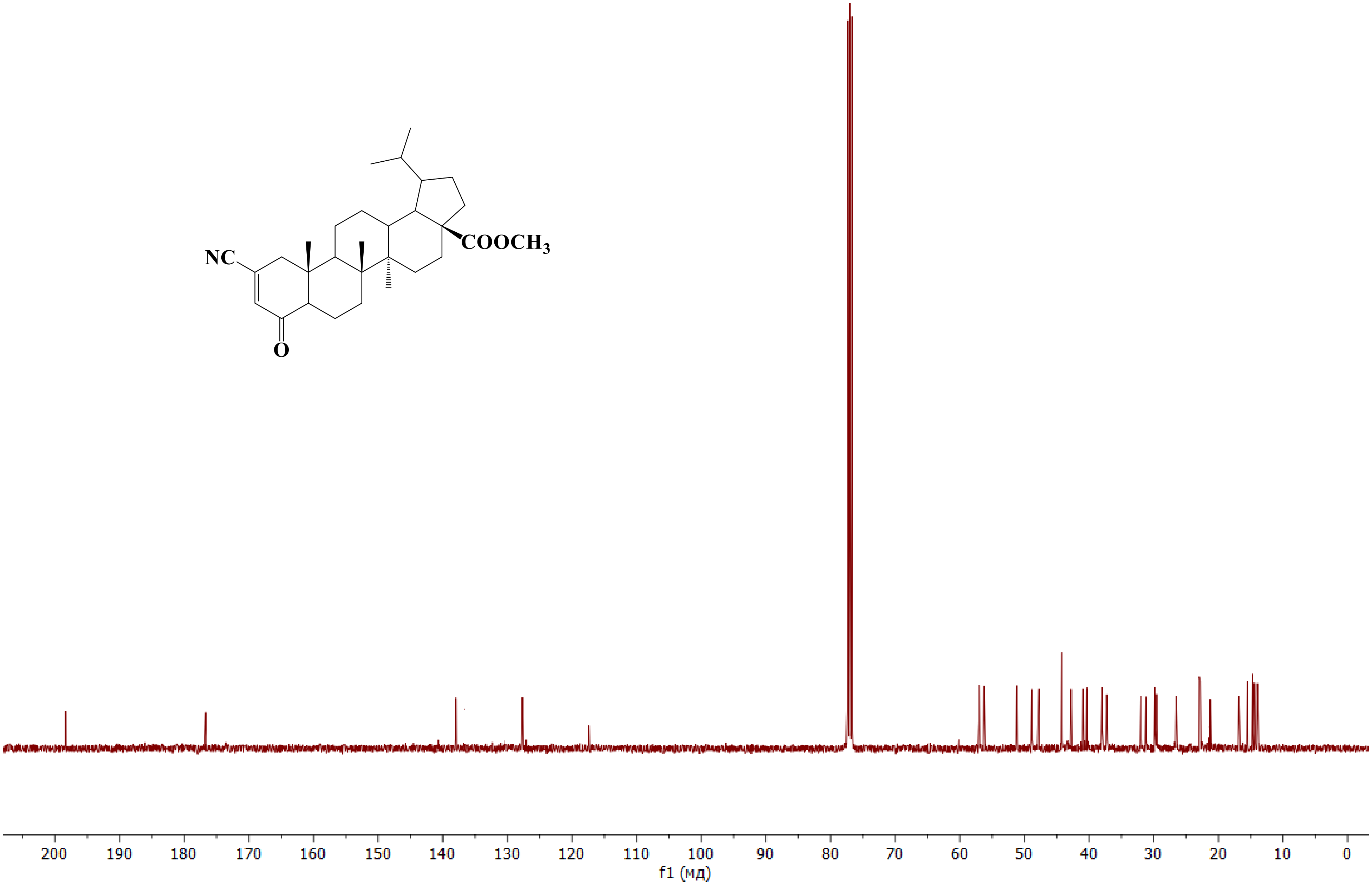

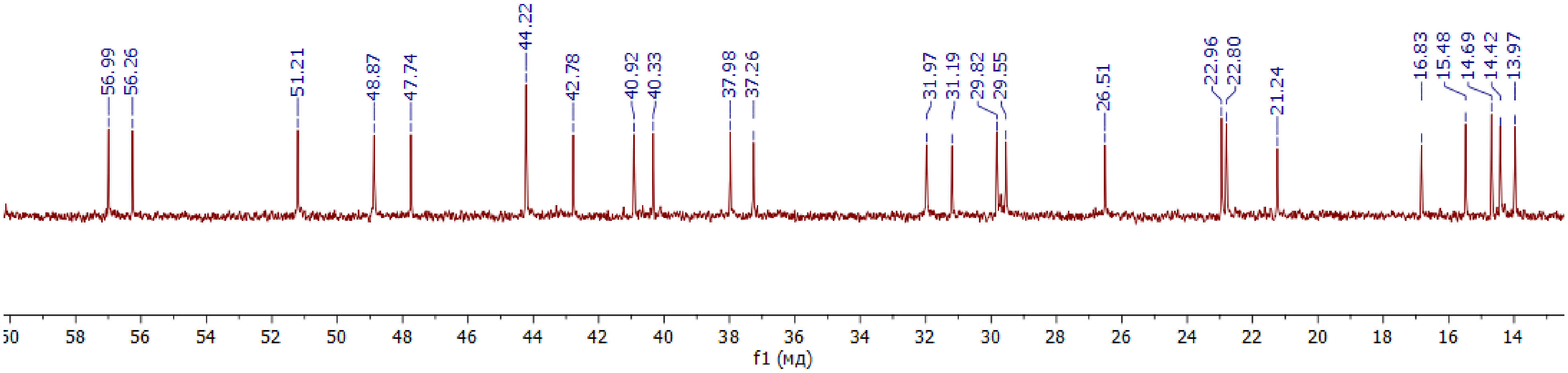
56.99
56.26
51.21
48.87
47.74
44.22
42.78
40.92
40.33
37.98
37.26
31.97
31.19
29.82
29.55
26.51
22.96
22.80
21.24
16.83
15.48
14.69
14.42
13.97
f1 (мд)

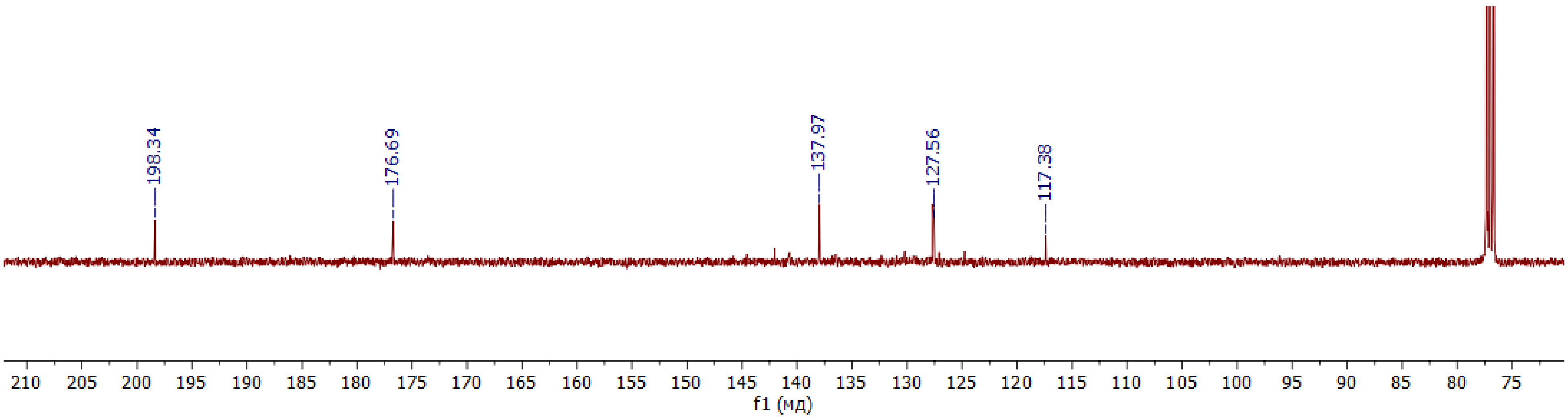
198.34
176.69
137.97
127.56
117.38
f1 (мд)

^{1}H NMR spectra of 2-Cyano-28-hydroxy-4-oxo-3,23-dinorlupan (16).

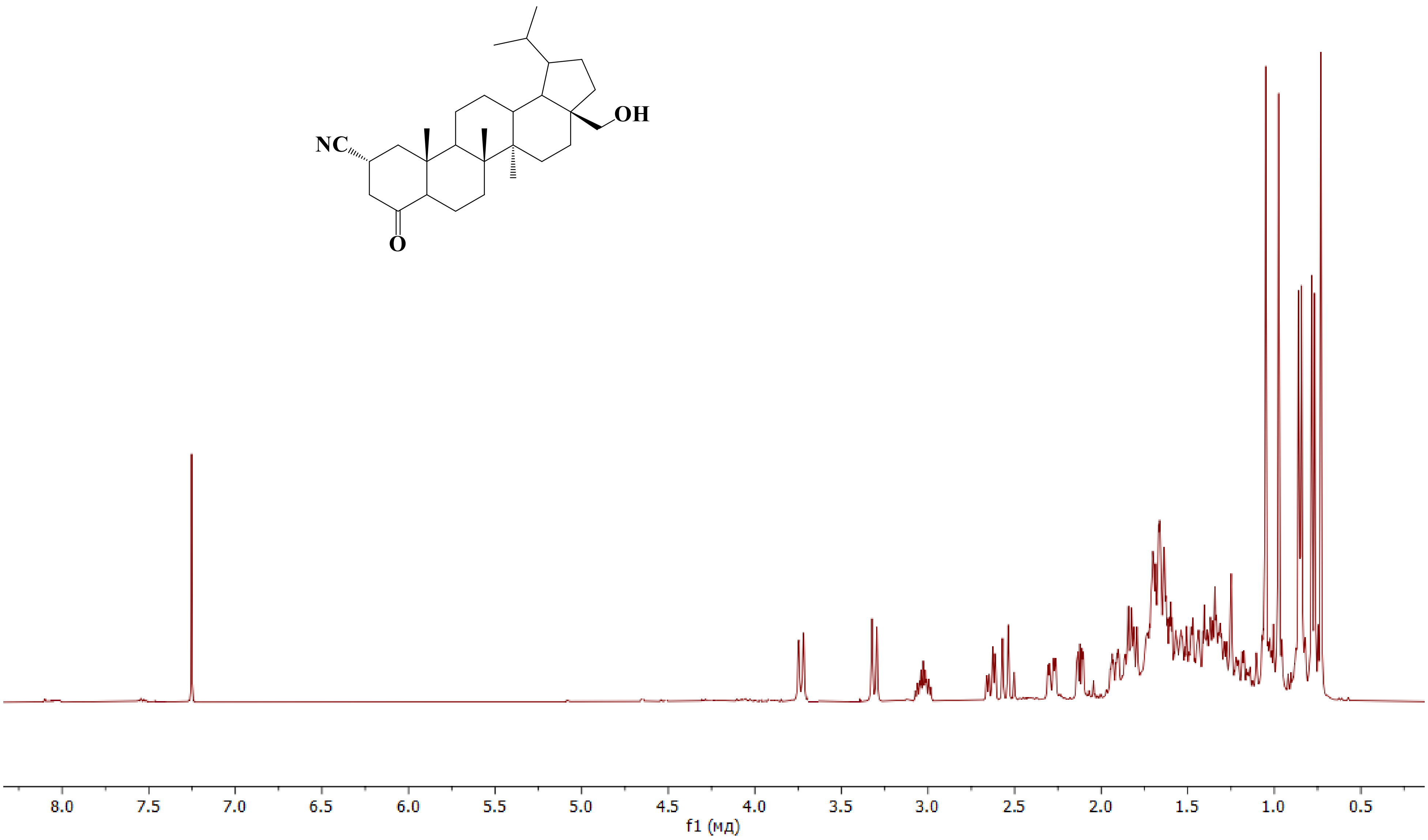

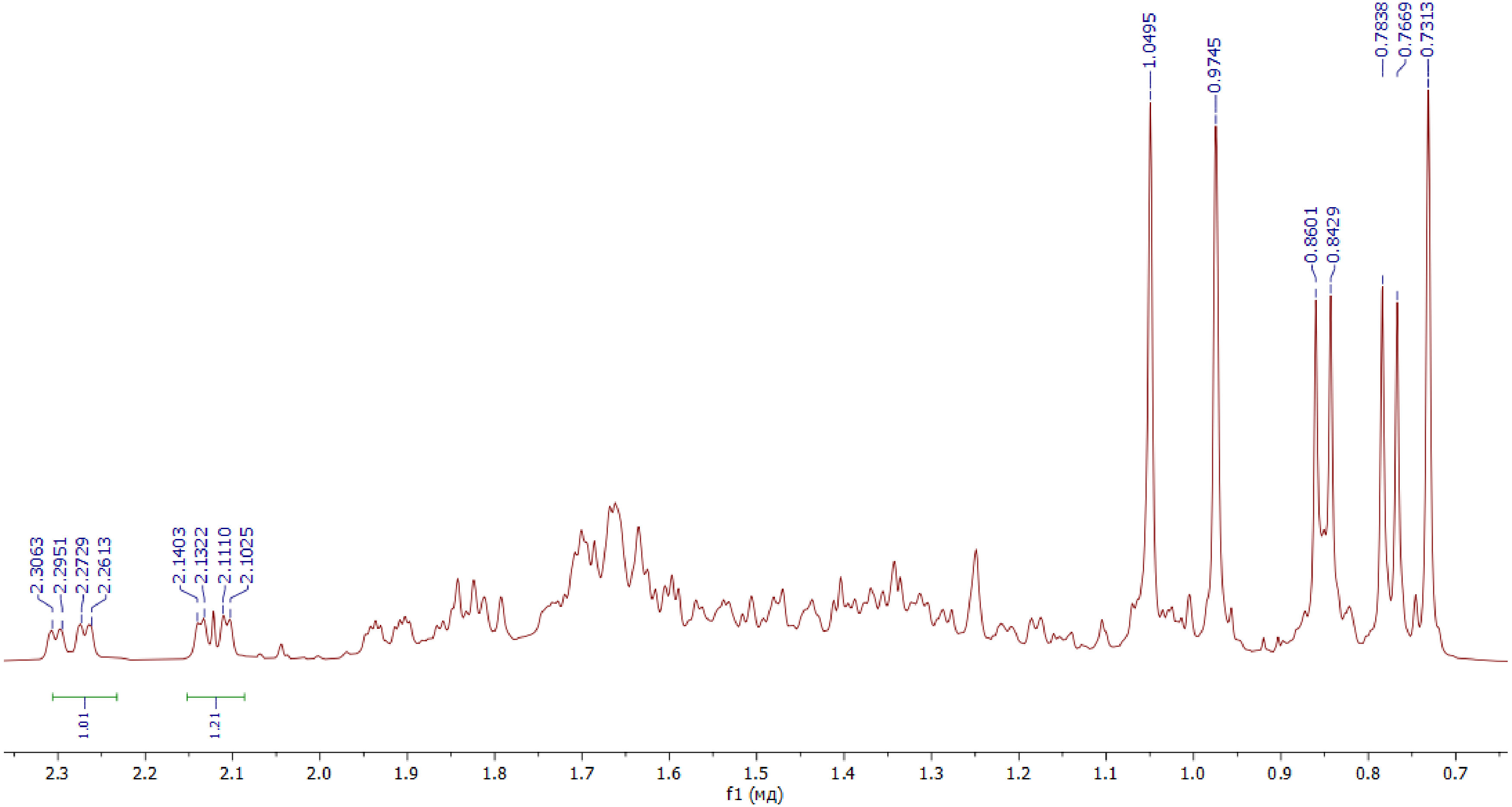
2.3063
2.2951
2.2729
2.2613
2.1403
2.1322
2.1110
2.1025
1.0495
0.9745
0.8601
0.8429
0.7838
0.7669
0.7313
1.01
1.21
2.3 2.2 2.1 2.0 1.9 1.8 1.7 1.6 1.5 1.4 1.3 1.2 1.1 1.0 0.9 0.8 0.7
f1 (мд)

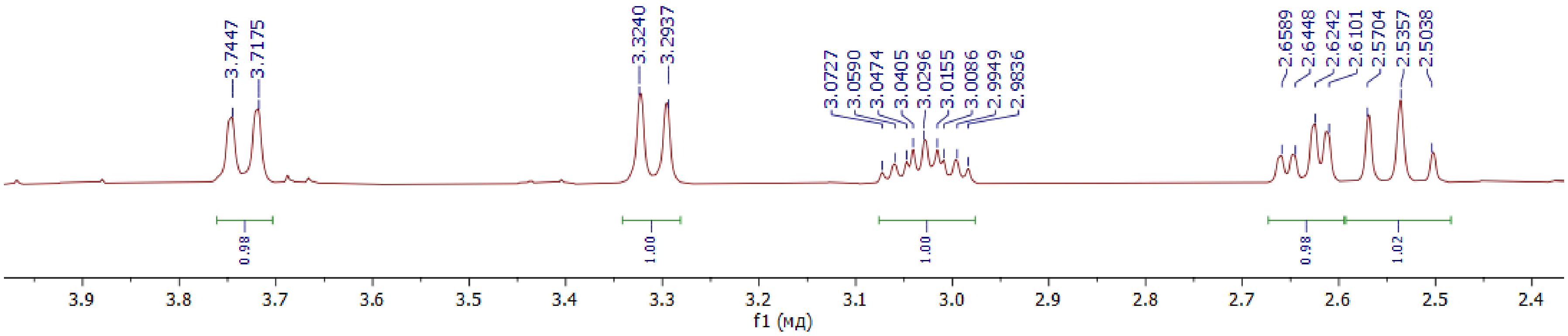
3.7447
3.7175
3.3240
3.2937
3.0727
3.0590
3.0474
3.0405
3.0296
3.0155
3.0086
2.9949
2.9836
2.6589
2.6448
2.6242
2.6101
2.5704
2.5357
2.5038
0.98
1.00
1.00
0.98
1.02
3.9 3.8 3.7 3.6 3.5 3.4 3.3 3.2 3.1 3.0 2.9 2.8 2.7 2.6 2.5 2.4
f1 (мд)

^{13}C NMR spectra of 2-Cyano-28-hydroxy-4-oxo-3,23-dinorlupan (16).

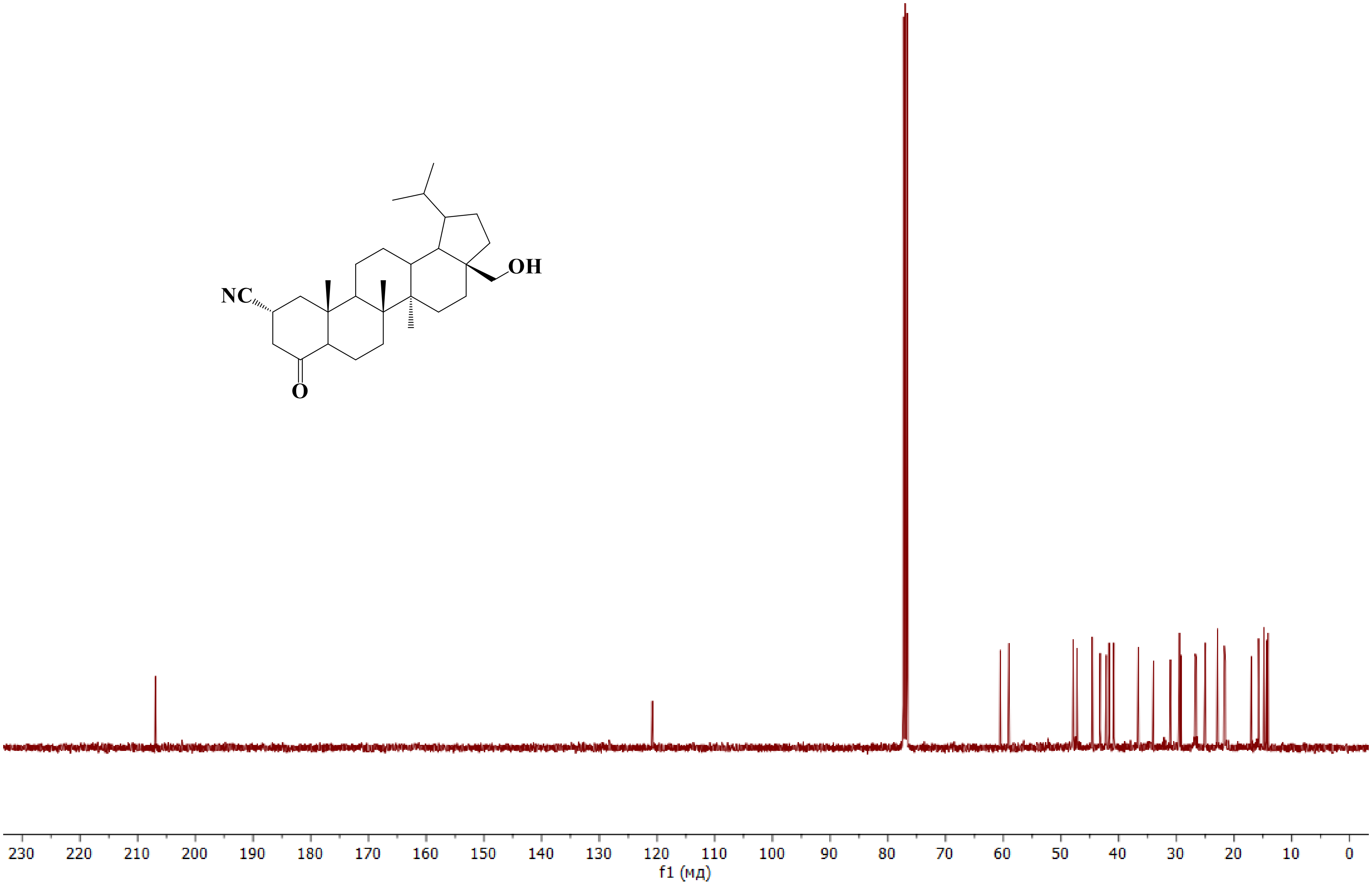

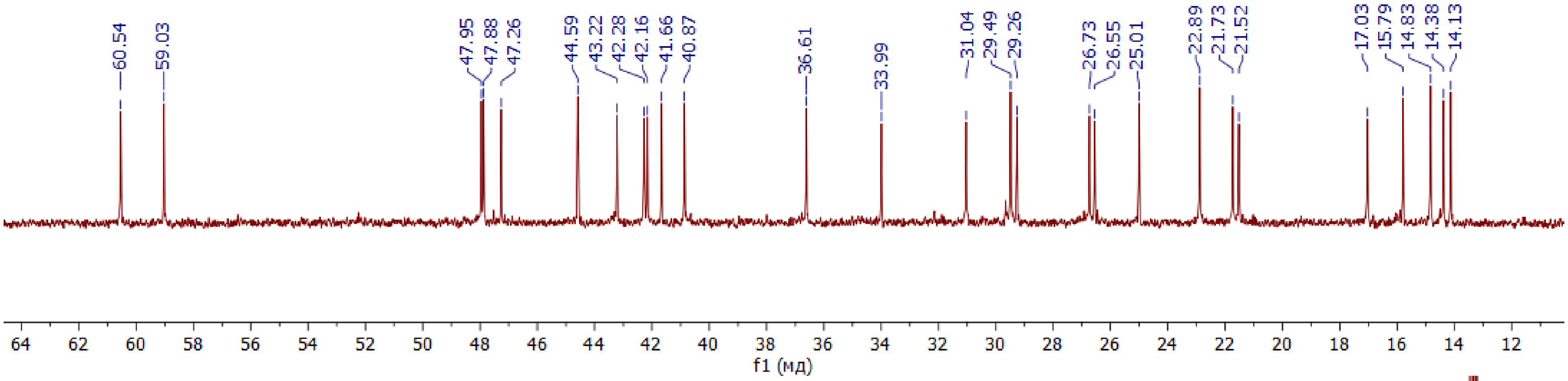

60.54
59.03
47.95
47.88
47.26
44.59
43.22
42.28
42.16
41.66
40.87
36.61
33.99
31.04
29.49
29.26
26.73
26.55
25.01
22.89
21.73
21.52
17.03
15.79
14.83
14.38
14.13
64 62 60 58 56 54 52 50 48 46 44 42 40 38 36 34 32 30 28 26 24 22 20 18 16 14 12
f1 (мд)

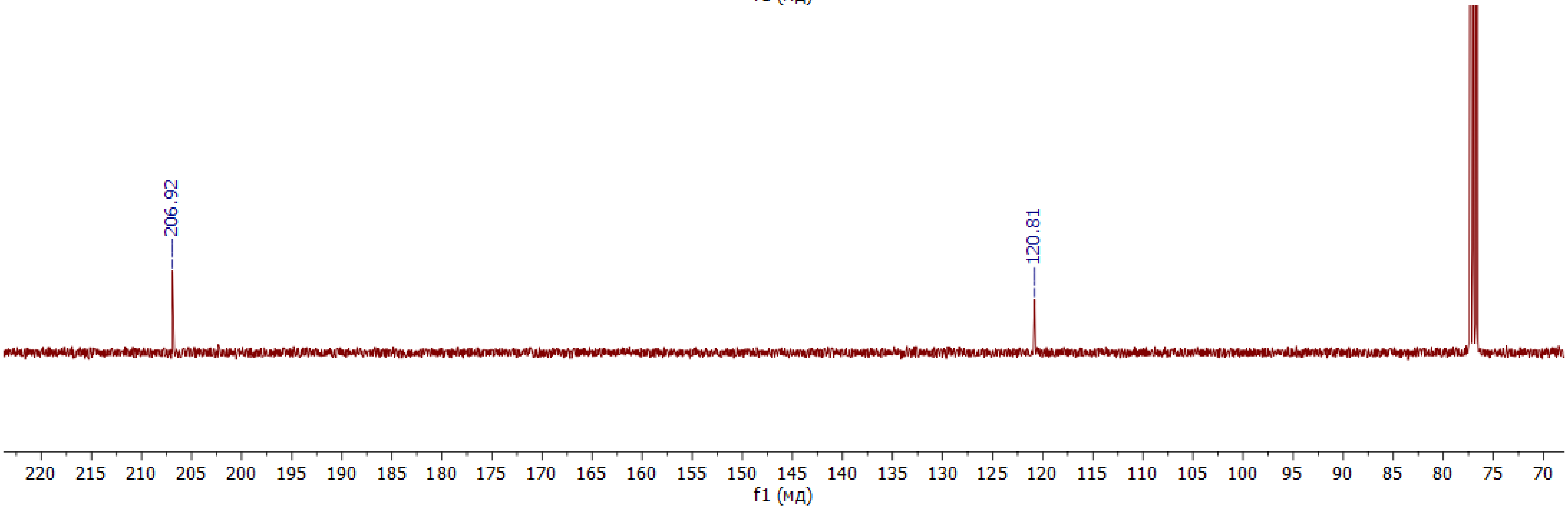

206.92
120.81
220 215 210 205 200 195 190 185 180 175 170 165 160 155 150 145 140 135 130 125 120 115 110 105 100 95 90 85 80 75 70
f1 (мд)

^{1}H NMR spectra of 2-Cyano-4-oxo-3,23-dinorlupan-28-oic acid methyl ester (17).

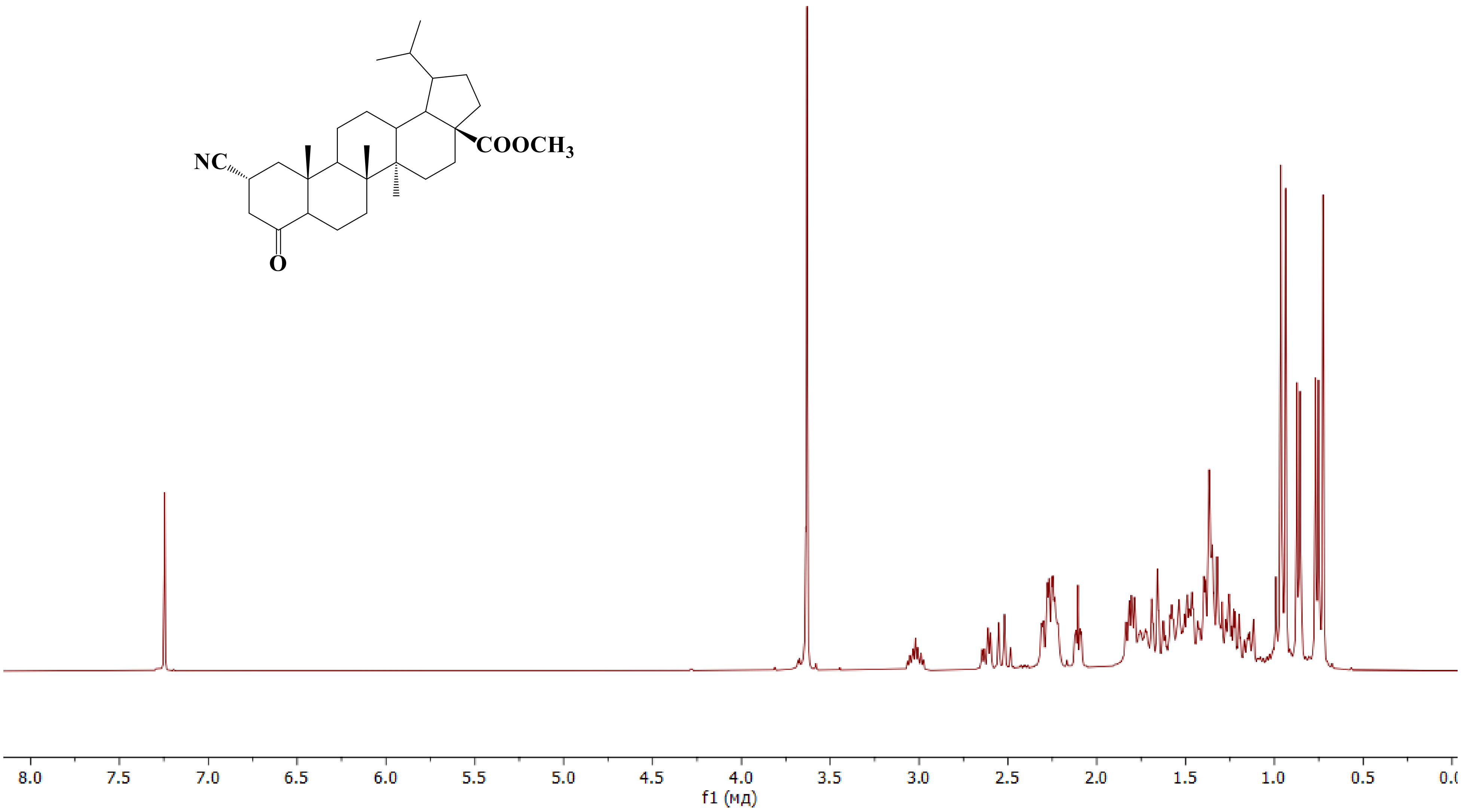

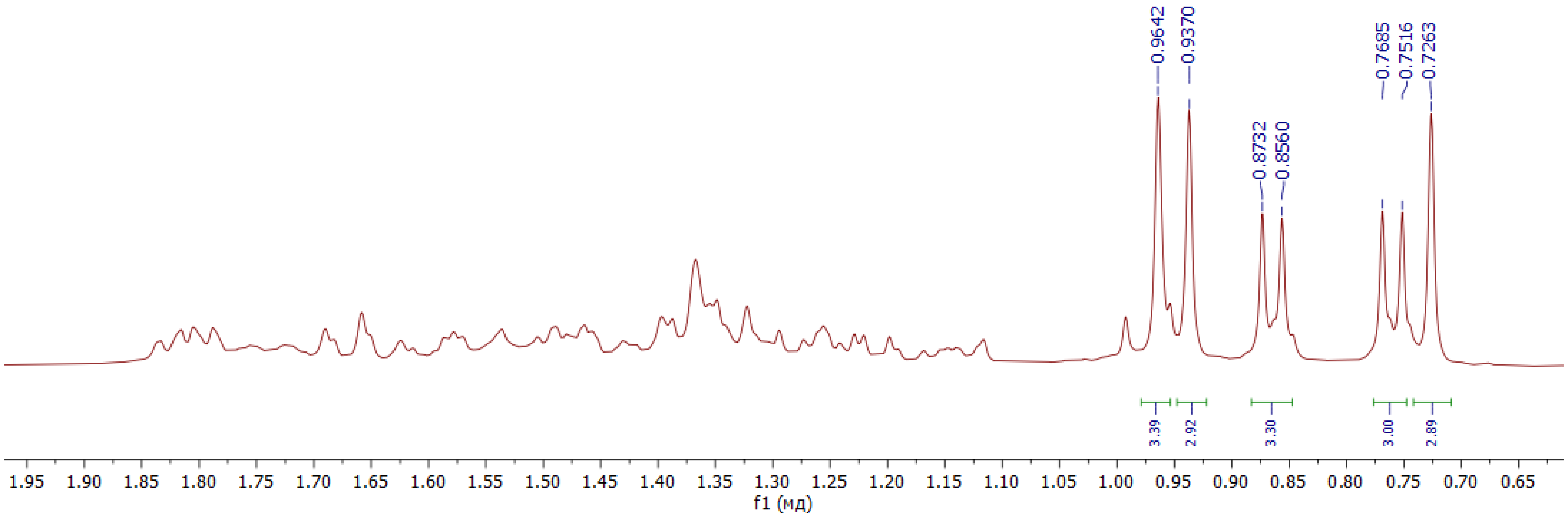

0.9642
0.9370
0.8732
0.8560
0.7685
0.7516
0.7263
3.39
2.92
3.30
3.00
2.89
1.95 1.90 1.85 1.80 1.75 1.70 1.65 1.60 1.55 1.50 1.45 1.40 1.35 1.30 1.25 1.20 1.15 1.10 1.05 1.00 0.95 0.90 0.85 0.80 0.75 0.70 0.65
f1 (мд)

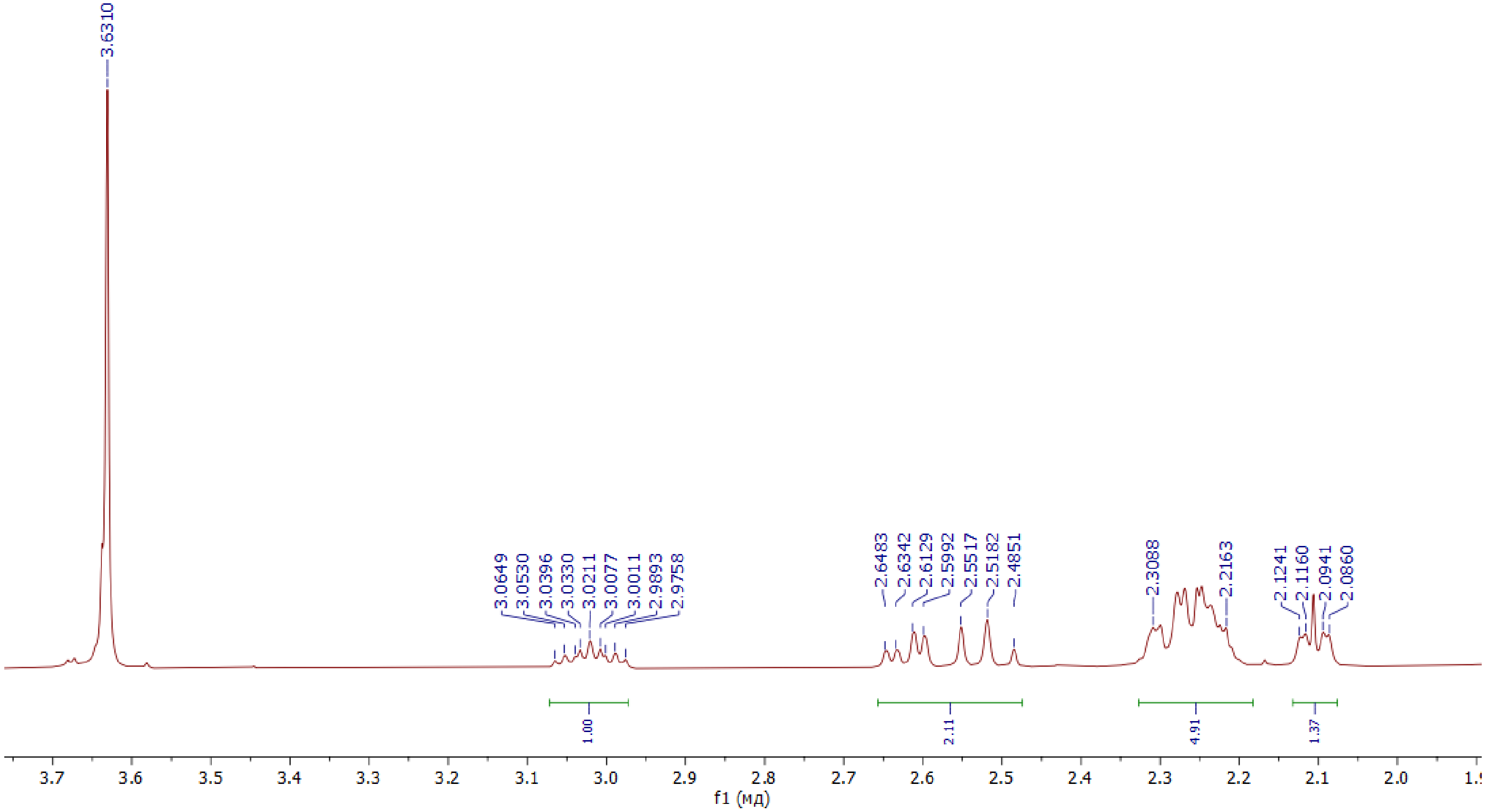

3.6310
3.0649
3.0530
3.0396
3.0330
3.0211
3.0077
3.0011
2.9893
2.9758
2.6483
2.6342
2.6129
2.5992
2.5517
2.5182
2.4851
2.3088
2.2163
2.1241
2.1160
2.0941
2.0860
1.00
2.11
4.91
1.37
3.7 3.6 3.5 3.4 3.3 3.2 3.1 3.0 2.9 2.8 2.7 2.6 2.5 2.4 2.3 2.2 2.1 2.0
f1 (мд)

^{13}C NMR spectra of 2-Cyano-4-oxo-3,23-dinorlupan-28-oic acid methyl ester (17).

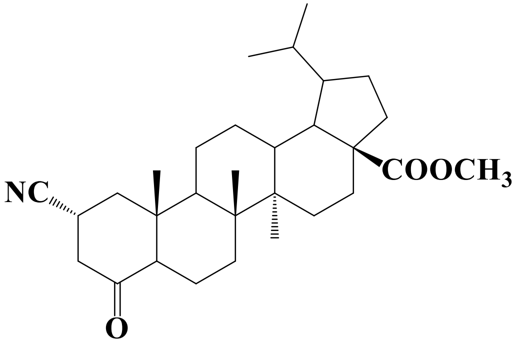

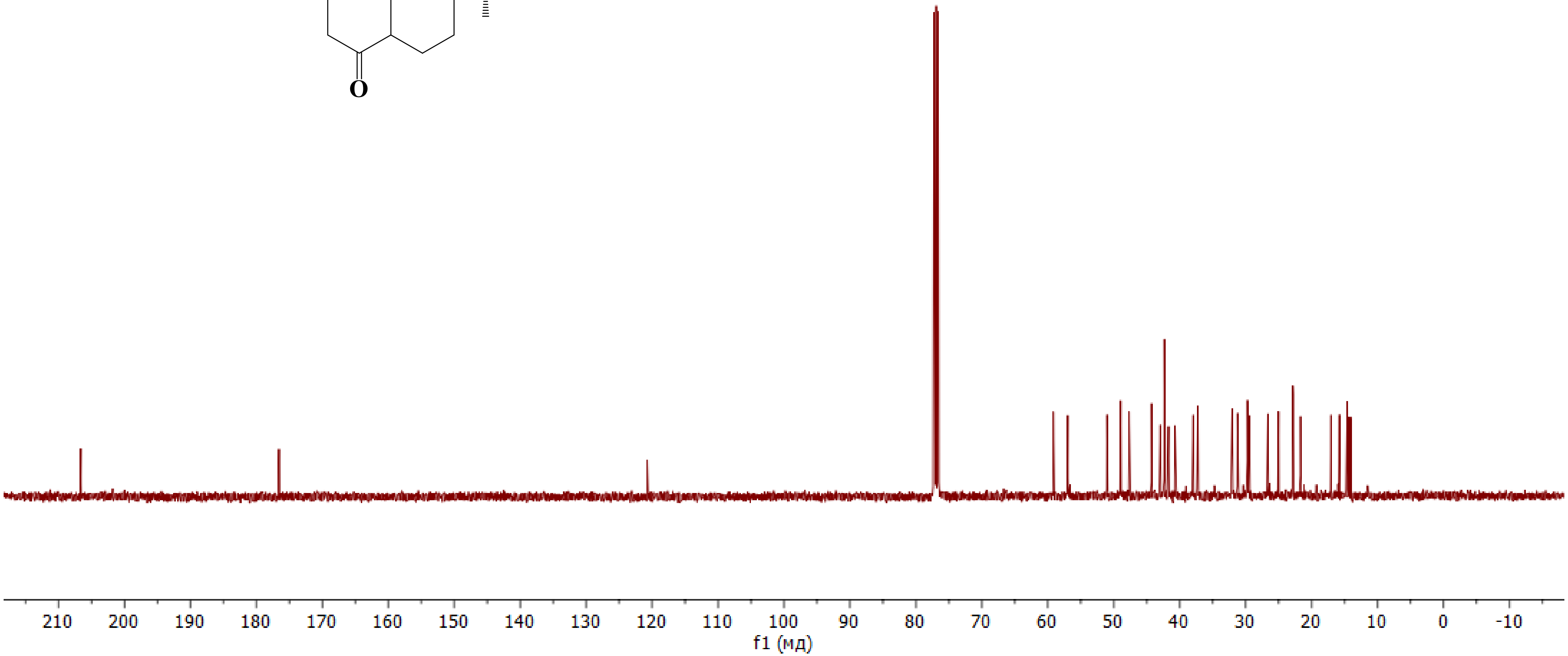

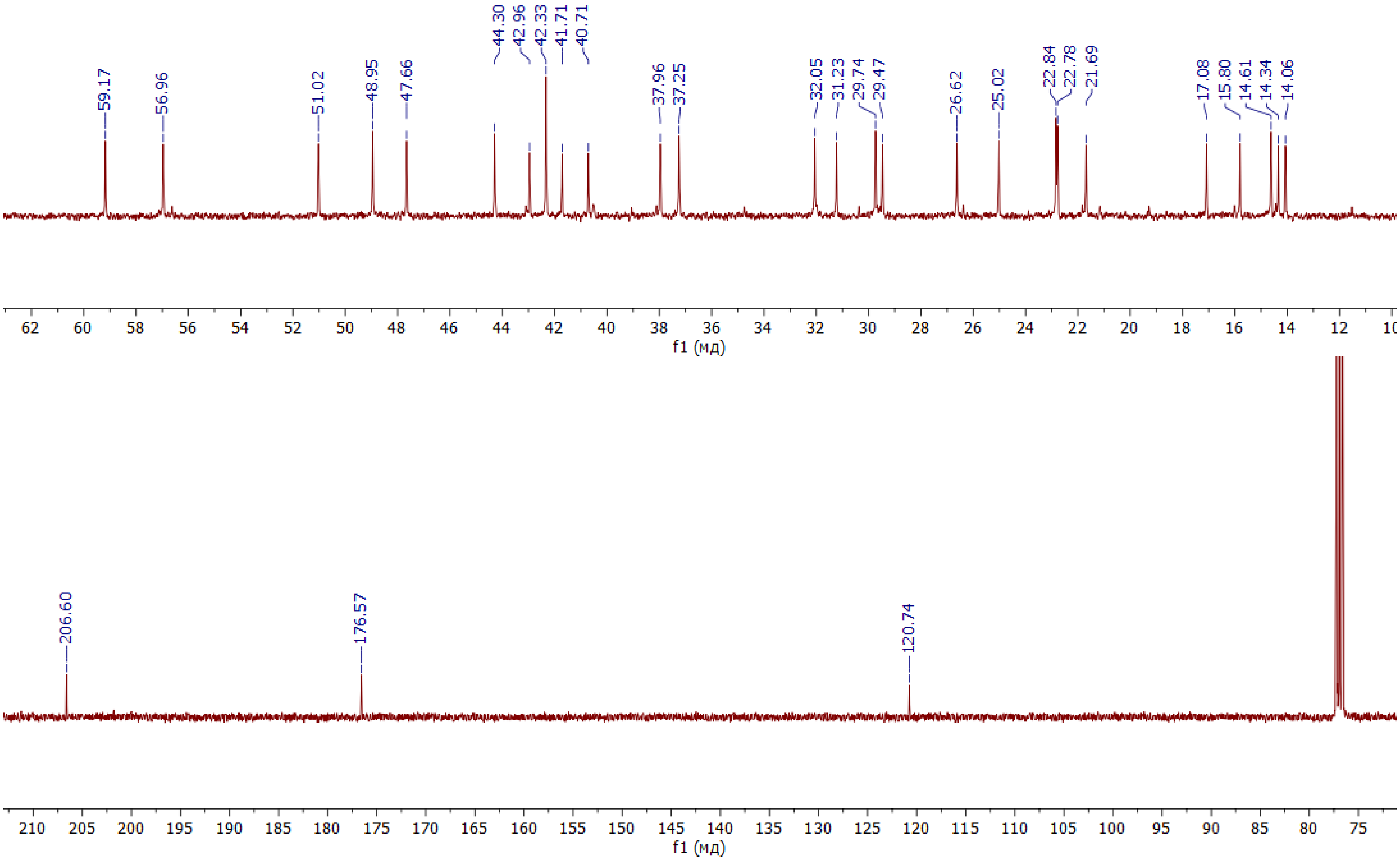

59.17
56.96
51.02
48.95
47.66
44.30
42.96
42.33
41.71
40.71
37.96
37.25
32.05
31.23
29.74
29.47
26.62
25.02
22.84
22.78
21.69
17.08
15.80
14.61
14.34
14.06
62 60 58 56 54 52 50 48 46 44 42 40 38 36 34 32 30 28 26 24 22 20 18 16 14 12
f1 (мд)
206.60
176.57
120.74
210 205 200 195 190 185 180 175 170 165 160 155 150 145 140 135 130 125 120 115 110 105 100 95 90 85 80 75
f1 (мд)

^{1}H NMR spectra of 2-Cyano-4,28-dihydroxy-3,23-dinorlupan (18).

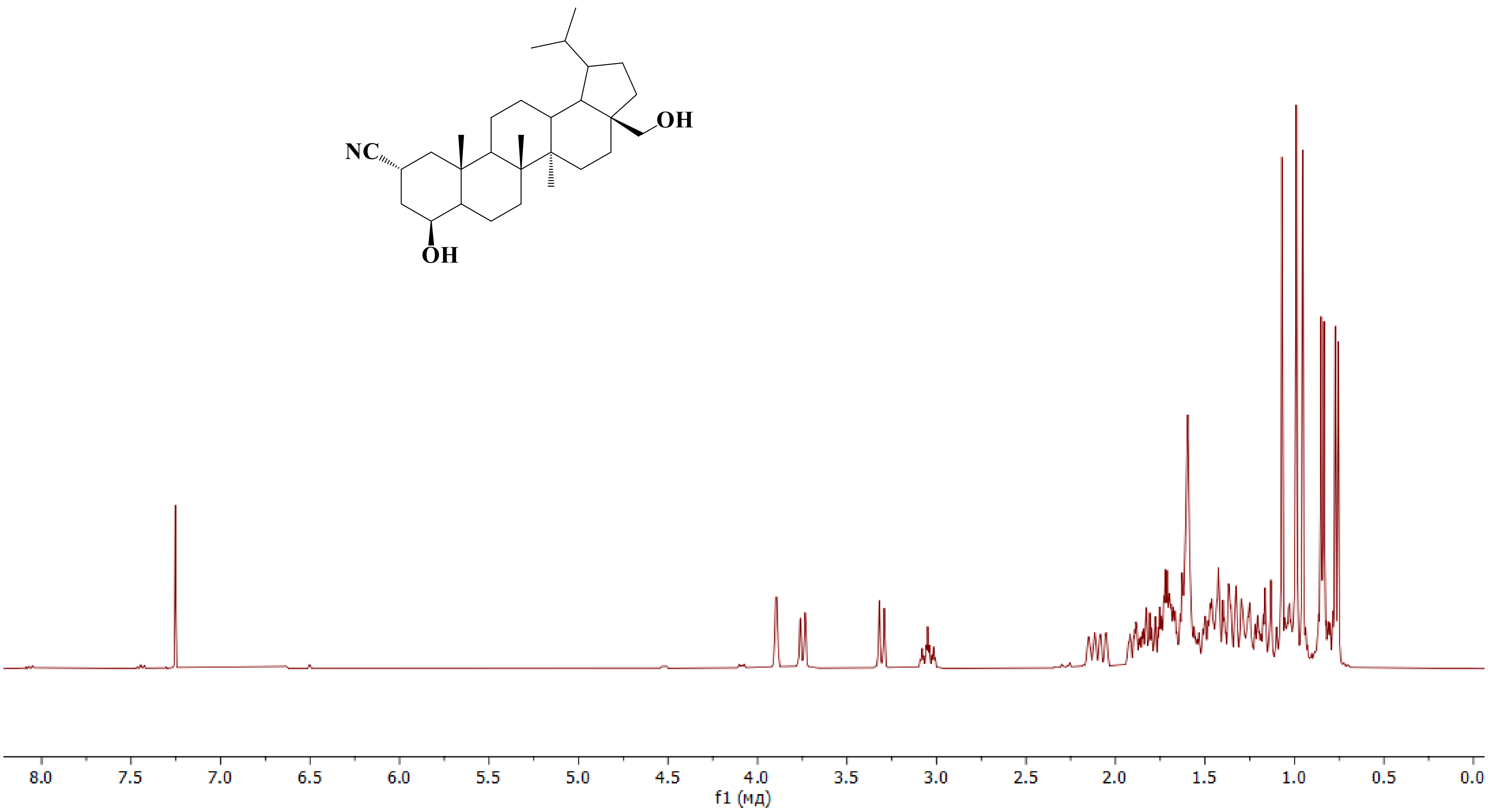

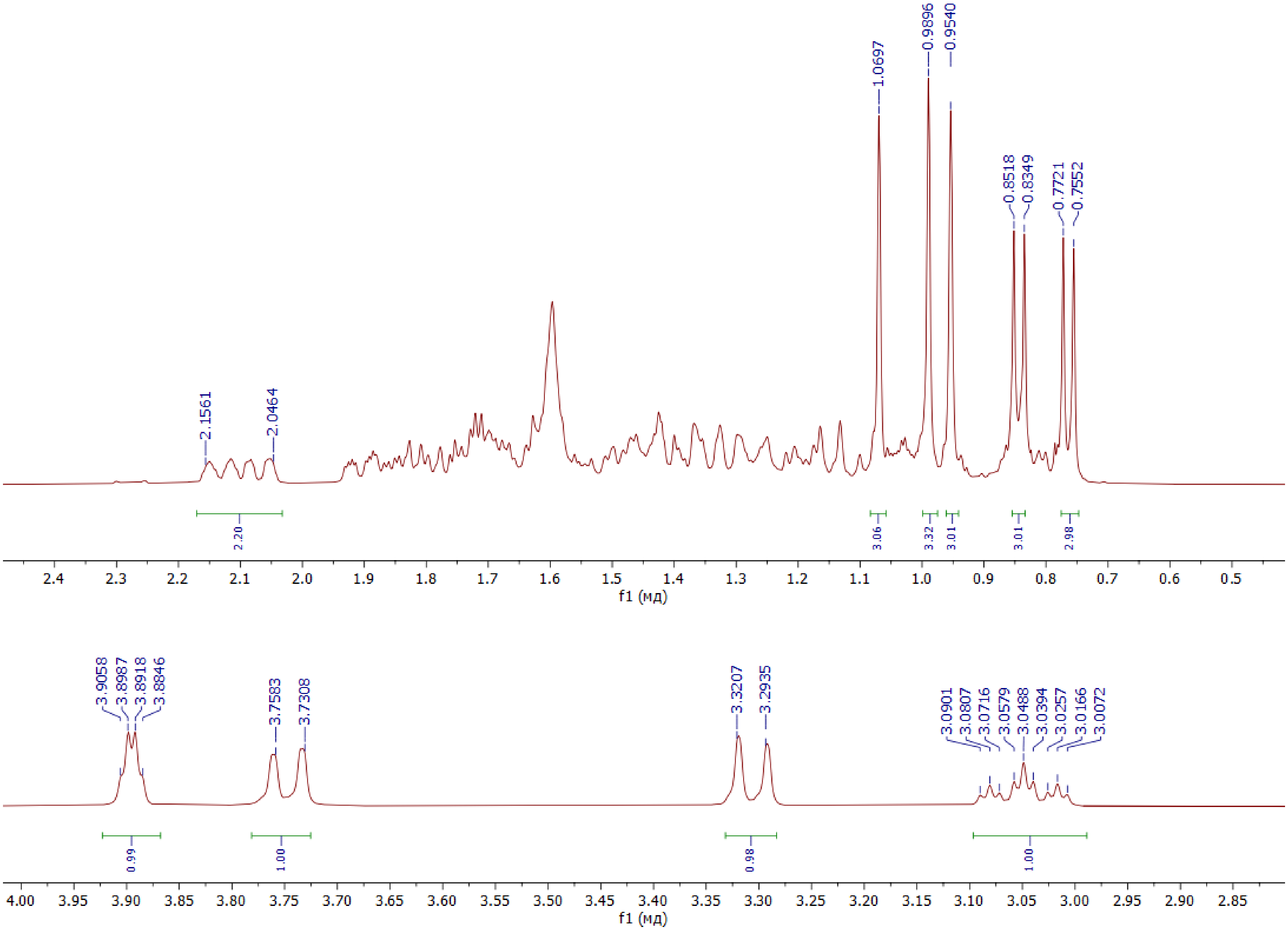
2.1561
2.0464
1.0697
0.9896
0.9540
0.8518
0.8349
0.7721
0.7552
2.20
3.06
3.32
3.01
3.01
2.98
2.4 2.3 2.2 2.1 2.0 1.9 1.8 1.7 1.6 1.5 1.4 1.3 1.2 1.1 1.0 0.9 0.8 0.7 0.6 0.5
f1 (мд)
3.9058
3.8987
3.8918
3.8846
3.7583
3.7308
3.3207
3.2935
3.0901
3.0807
3.0716
3.0579
3.0488
3.0394
3.0257
3.0166
3.0072
0.99
1.00
0.98
1.00
4.00 3.95 3.90 3.85 3.80 3.75 3.70 3.65 3.60 3.55 3.50 3.45 3.40 3.35 3.30 3.25 3.20 3.15 3.10 3.05 3.00 2.95 2.90 2.85
f1 (мд)

^{13}C NMR spectra of 2-Cyano-4,28-dihydroxy-3,23-dinorlupan (18).

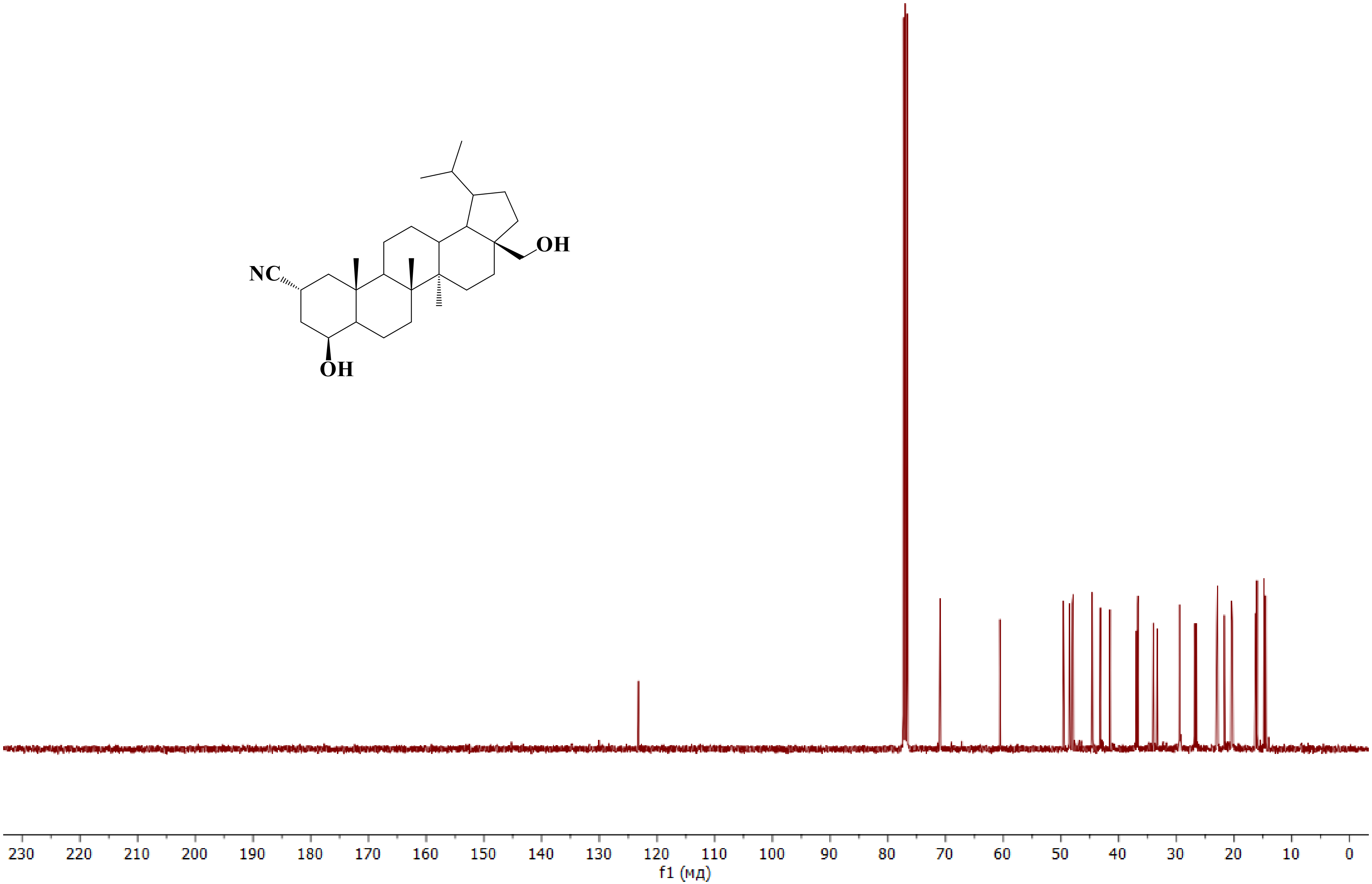

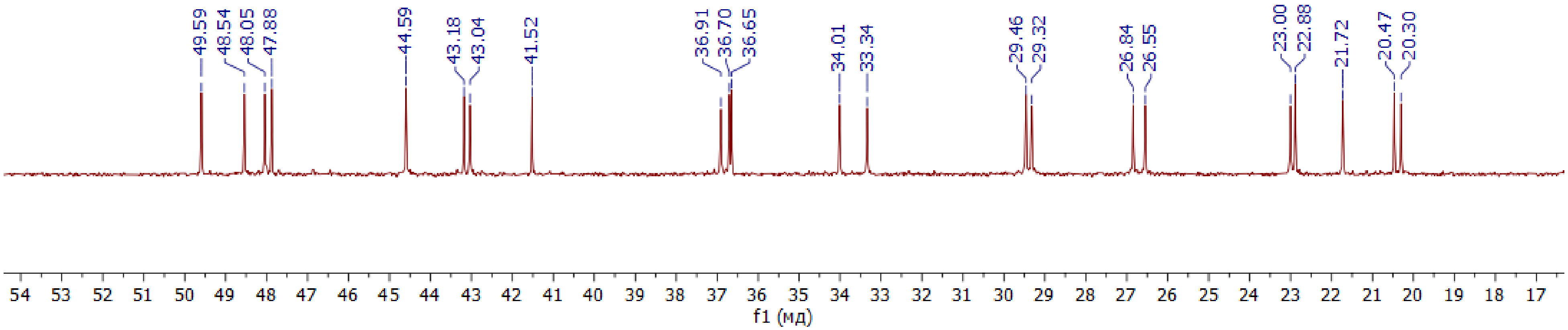
49.59
48.54
48.05
47.88
44.59
43.18
43.04
41.52
36.91
36.70
36.65
34.01
33.34
29.46
29.32
26.84
26.55
23.00
22.88
21.72
20.47
20.30
54 53 52 51 50 49 48 47 46 45 44 43 42 41 40 39 38 37 36 35 34 33 32 31 30 29 28 27 26 25 24 23 22 21 20 19 18 17
f1 (мд)

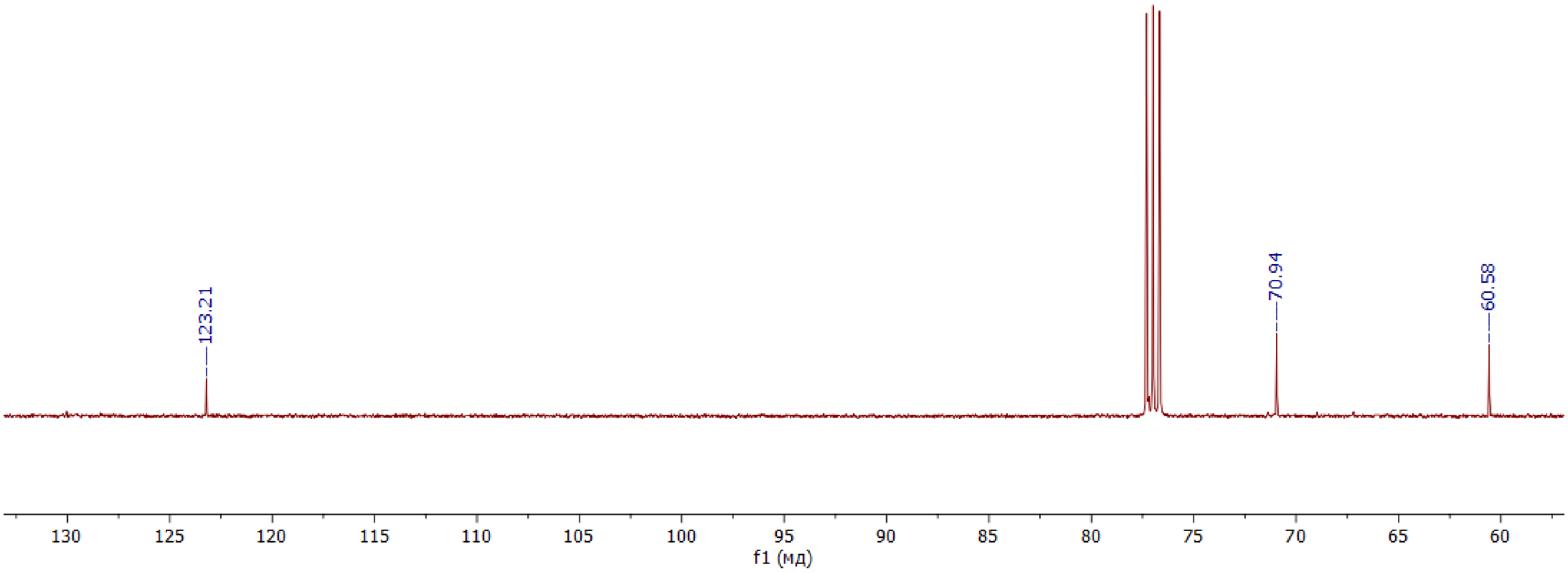
123.21
70.94
60.58
130 125 120 115 110 105 100 95 90 85 80 75 70 65 60
f1 (мд)

¹H NMR spectra of 2-Cyano-4-hydroxy-3,23-dinorlupan-28-oic acid methyl ester (19).

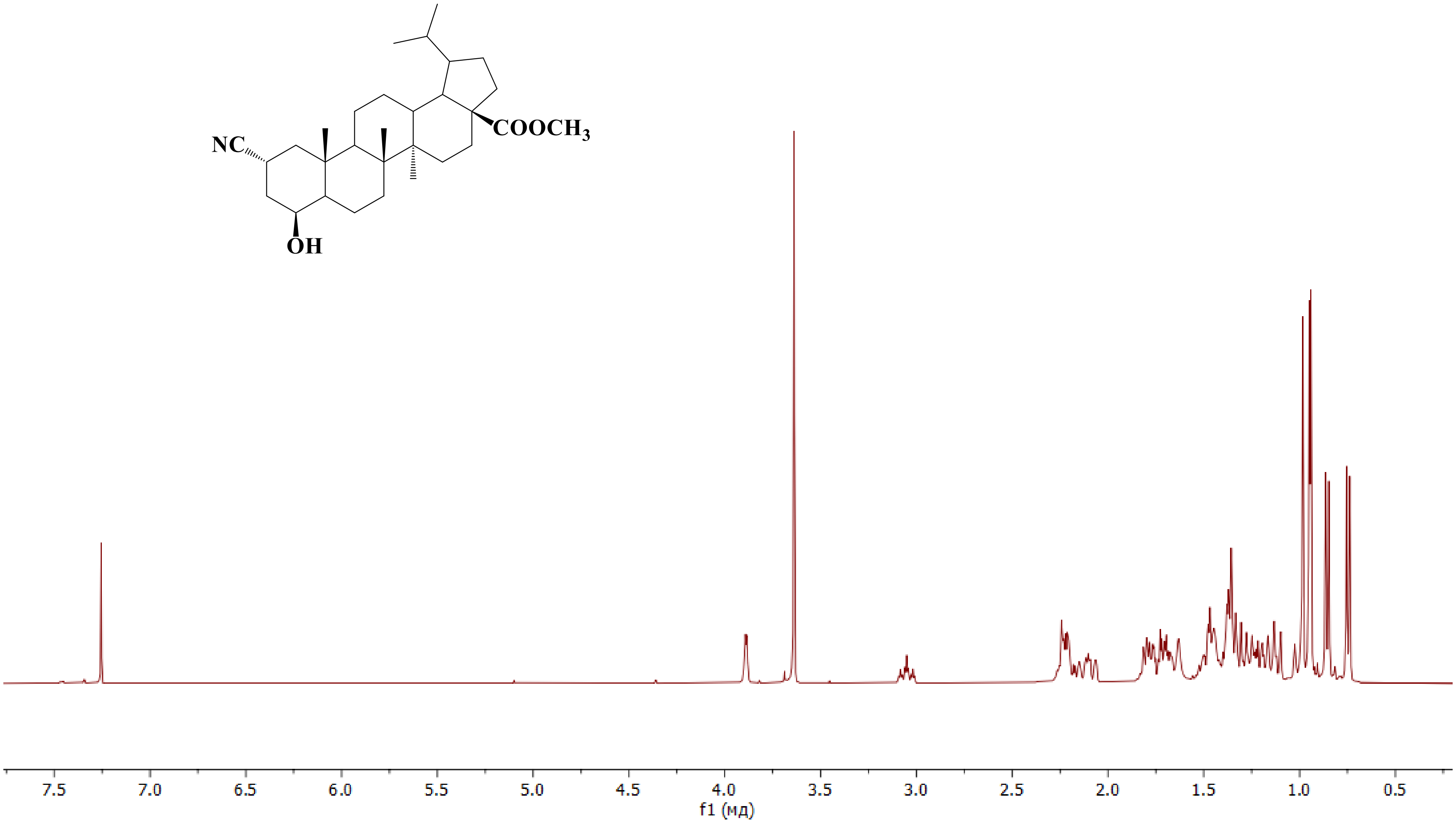

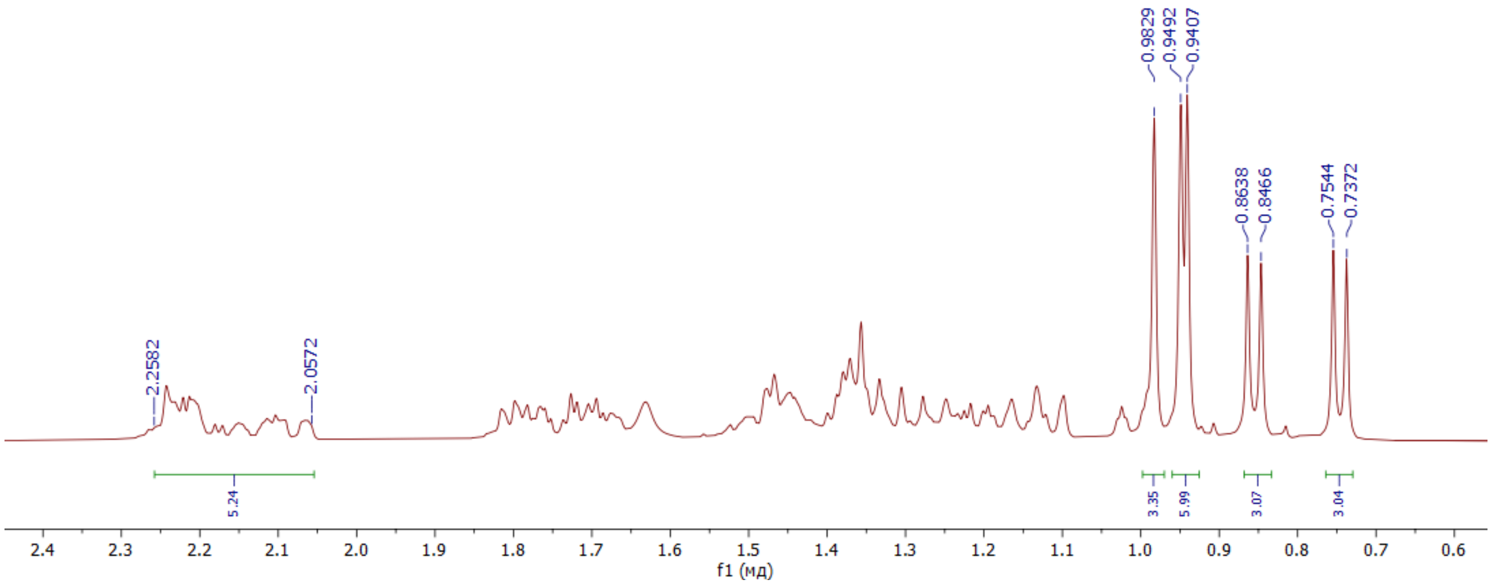

2.2582
2.0572
0.9829
0.9492
0.9407
0.8638
0.8466
0.7544
0.7372
5.24
3.35
5.99
3.07
3.04
2.4 2.3 2.2 2.1 2.0 1.9 1.8 1.7 1.6 1.5 1.4 1.3 1.2 1.1 1.0 0.9 0.8 0.7 0.6
f1 (мд)

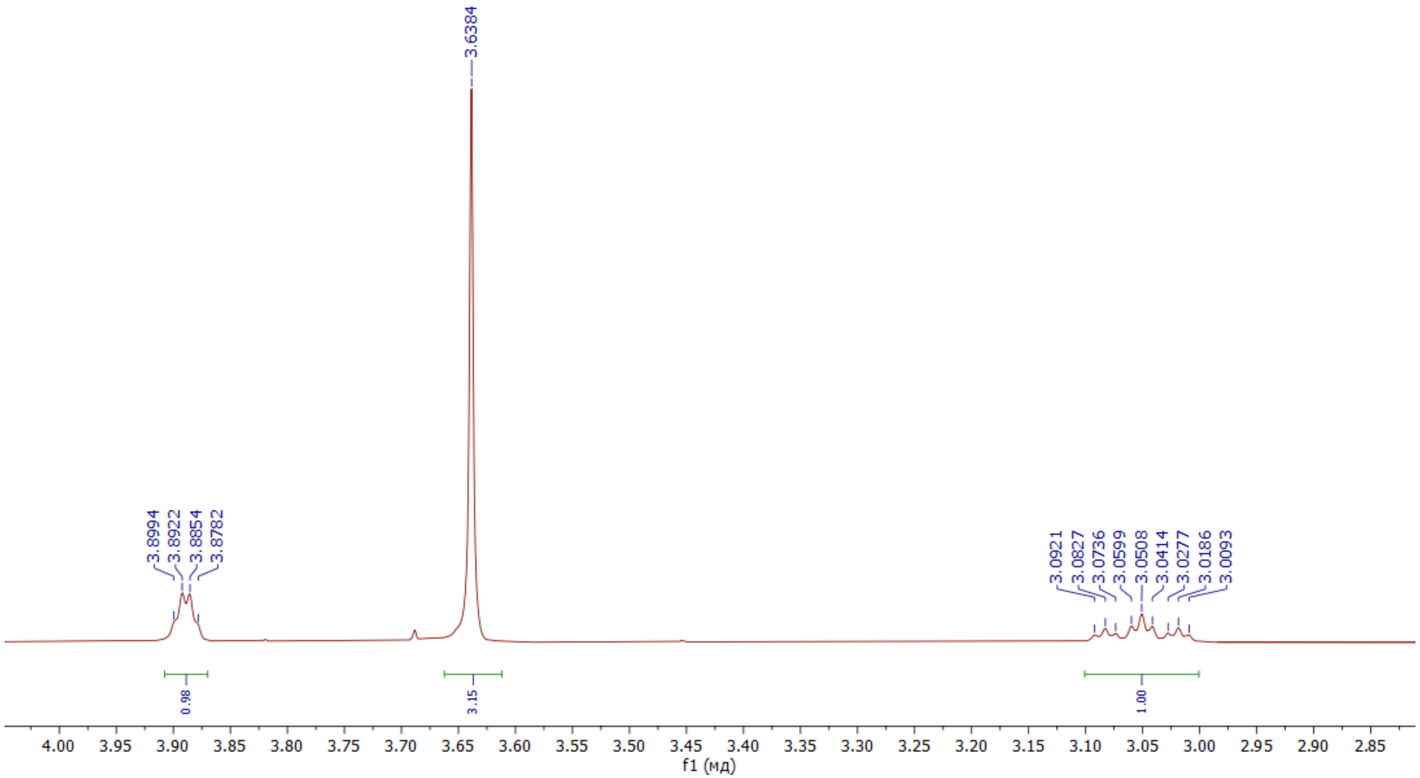

3.6384
3.8994
3.8922
3.8854
3.8782
3.0921
3.0827
3.0736
3.0599
3.0508
3.0414
3.0277
3.0186
3.0093
0.98
3.15
1.00
4.00 3.95 3.90 3.85 3.80 3.75 3.70 3.65 3.60 3.55 3.50 3.45 3.40 3.35 3.30 3.25 3.20 3.15 3.10 3.05 3.00 2.95 2.90 2.85
f1 (мд)

^{13}C NMR spectra of 2-Cyano-4-hydroxy-3,23-dinorlupan-28-oic acid methyl ester (19).

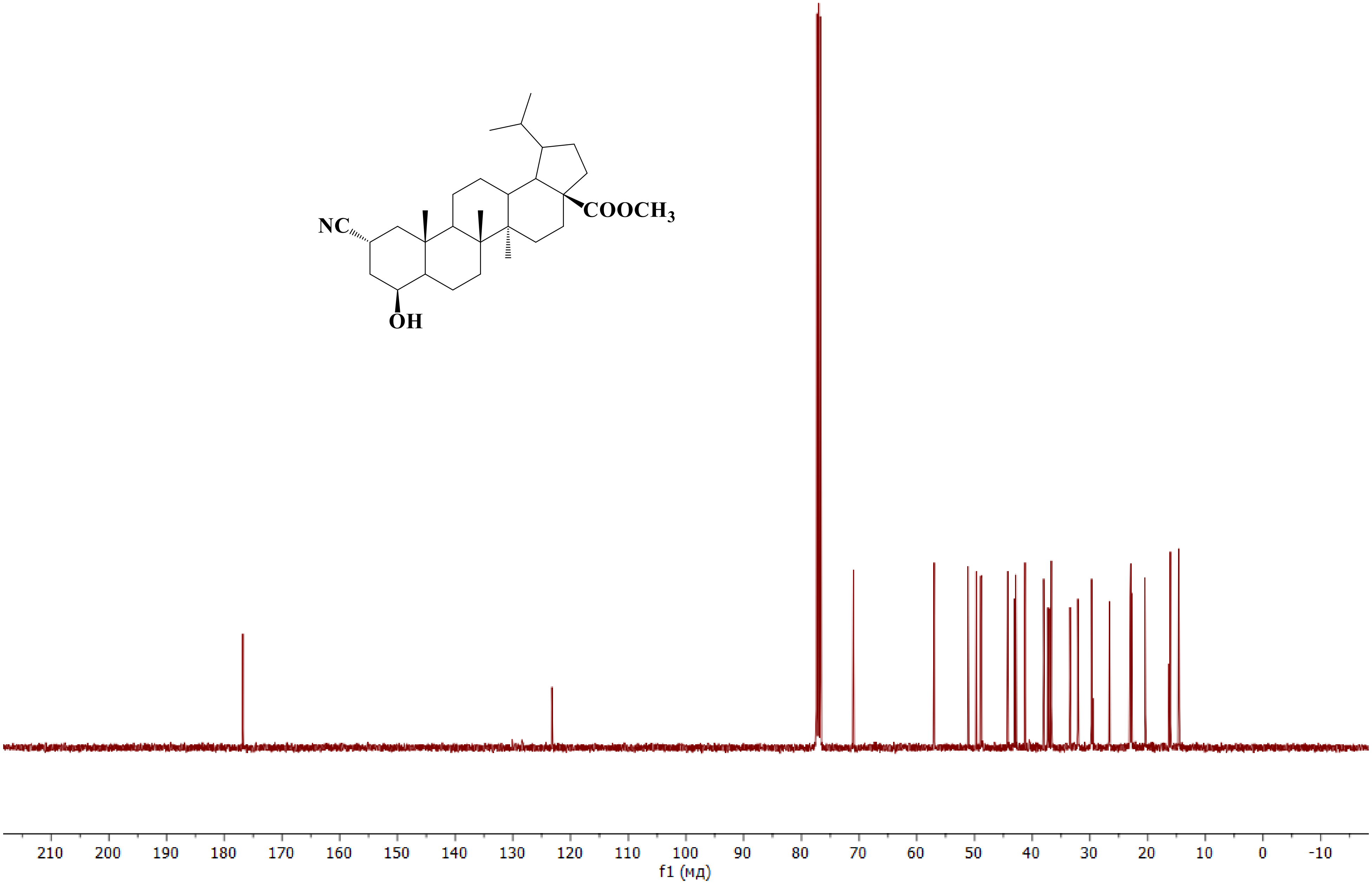

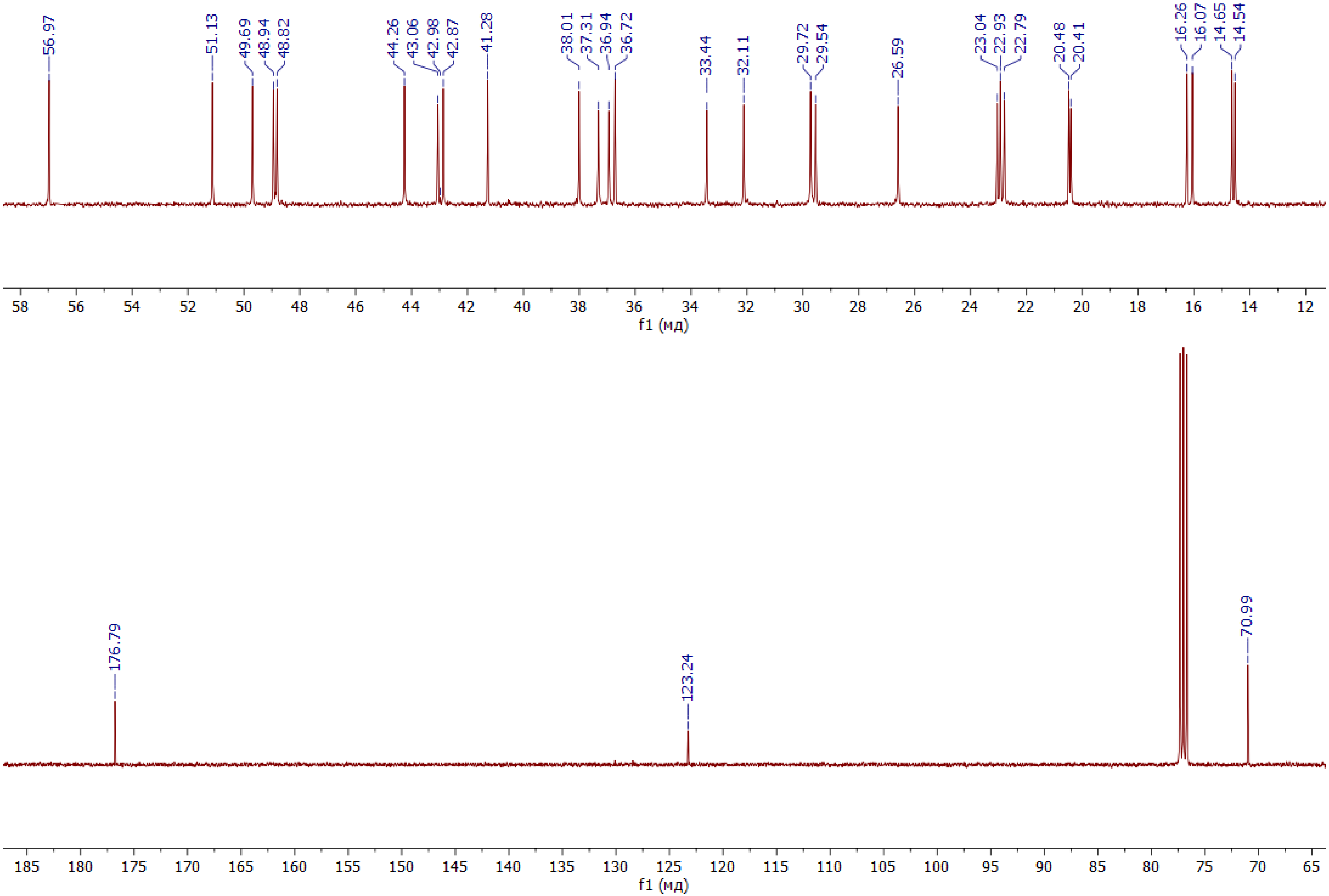
56.97
51.13
49.69
48.94
48.82
44.26
43.06
42.98
42.87
41.28
38.01
37.31
36.94
36.72
33.44
32.11
29.72
29.54
26.59
23.04
22.93
22.79
20.48
20.41
16.26
16.07
14.65
14.54
58 56 54 52 50 48 46 44 42 40 38 36 34 32 30 28 26 24 22 20 18 16 14 12
f1 (мд)
176.79
123.24
70.99
185 180 175 170 165 160 155 150 145 140 135 130 125 120 115 110 105 100 95 90 85 80 75 70 65
f1 (мд)

COSY NMR spectra of 2-Cyano-4-hydroxy-3,23-dinorlupan-28-oic acid methyl ester (19).

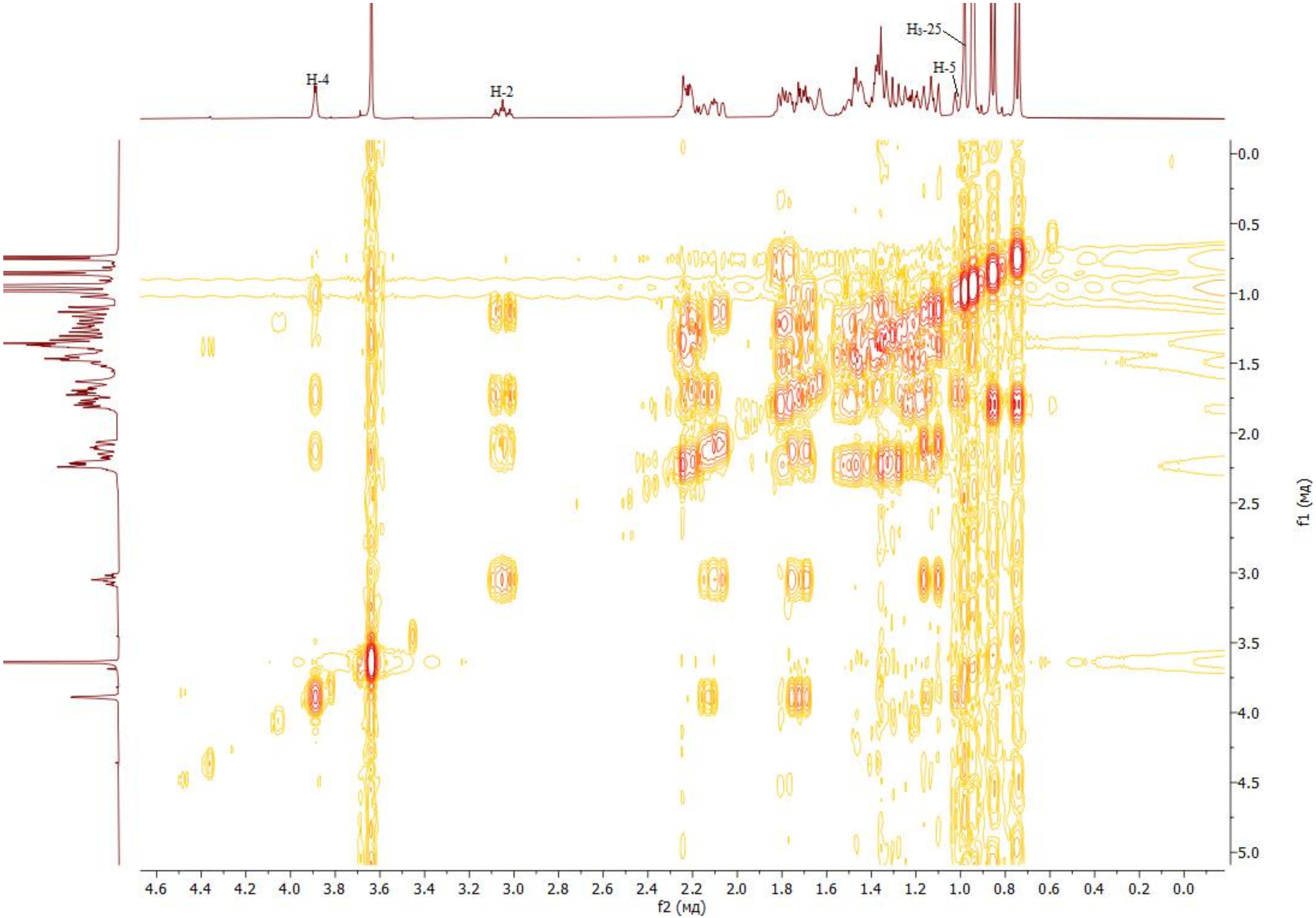

HSQC NMR spectra of 2-Cyano-4-hydroxy-3,23-dinorlupan-28-oic acid methyl ester (19).

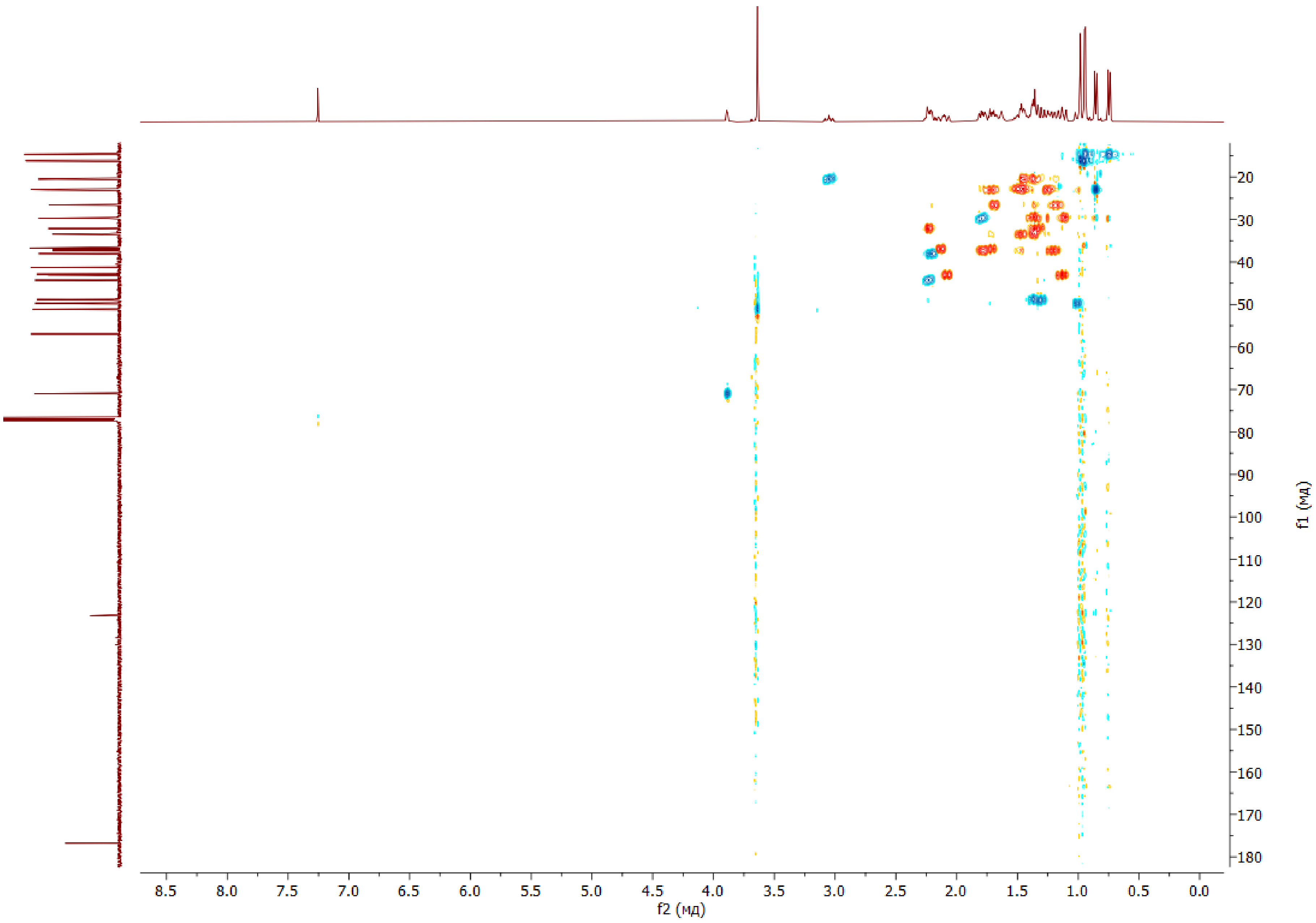

HSQC NMR spectra of 2-Cyano-4-hydroxy-3,23-dinorlupan-28-oic acid methyl ester (19).

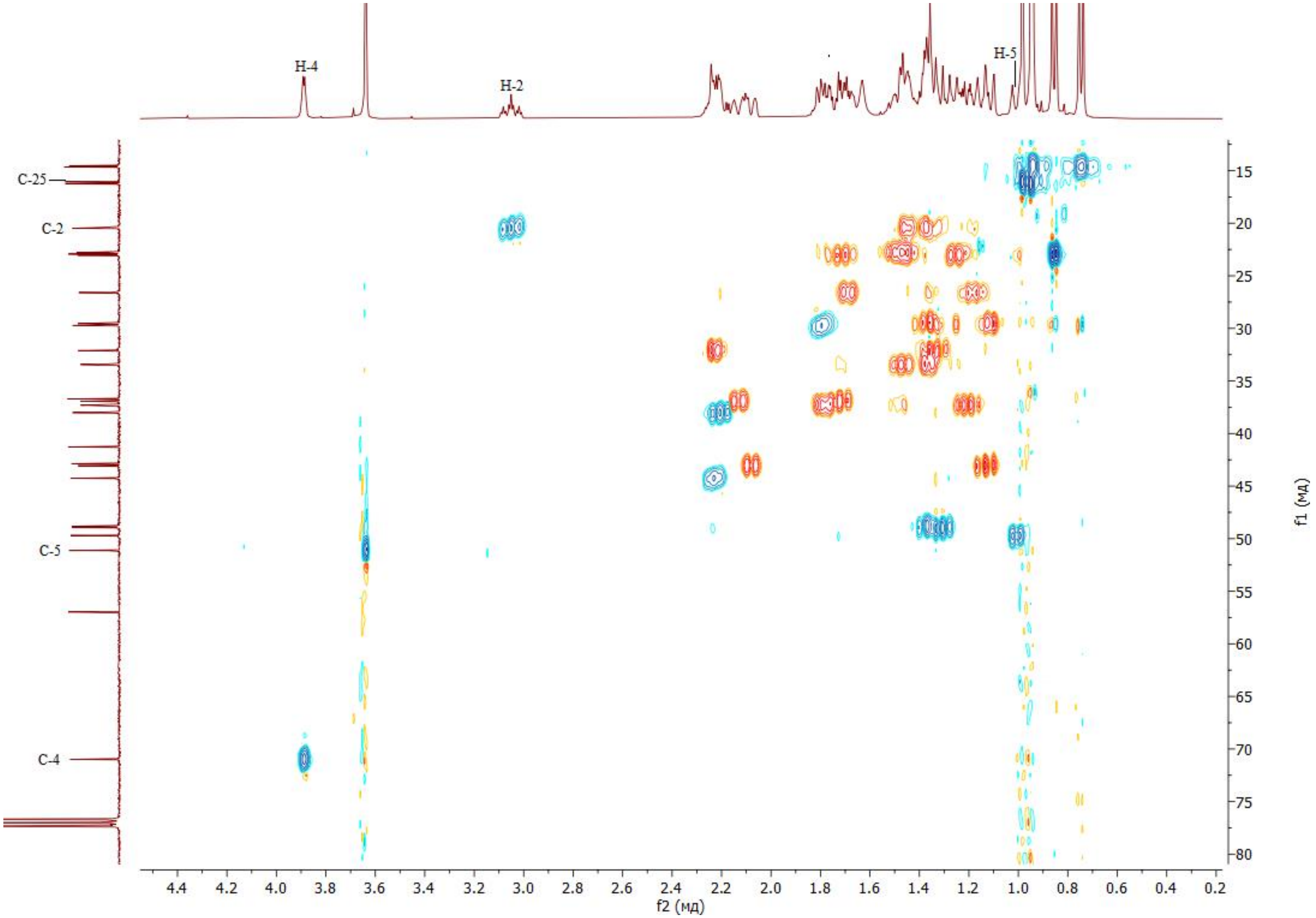

HMBC NMR spectra of 2-Cyano-4-hydroxy-3,23-dinorlupan-28-oic acid methyl ester (19).

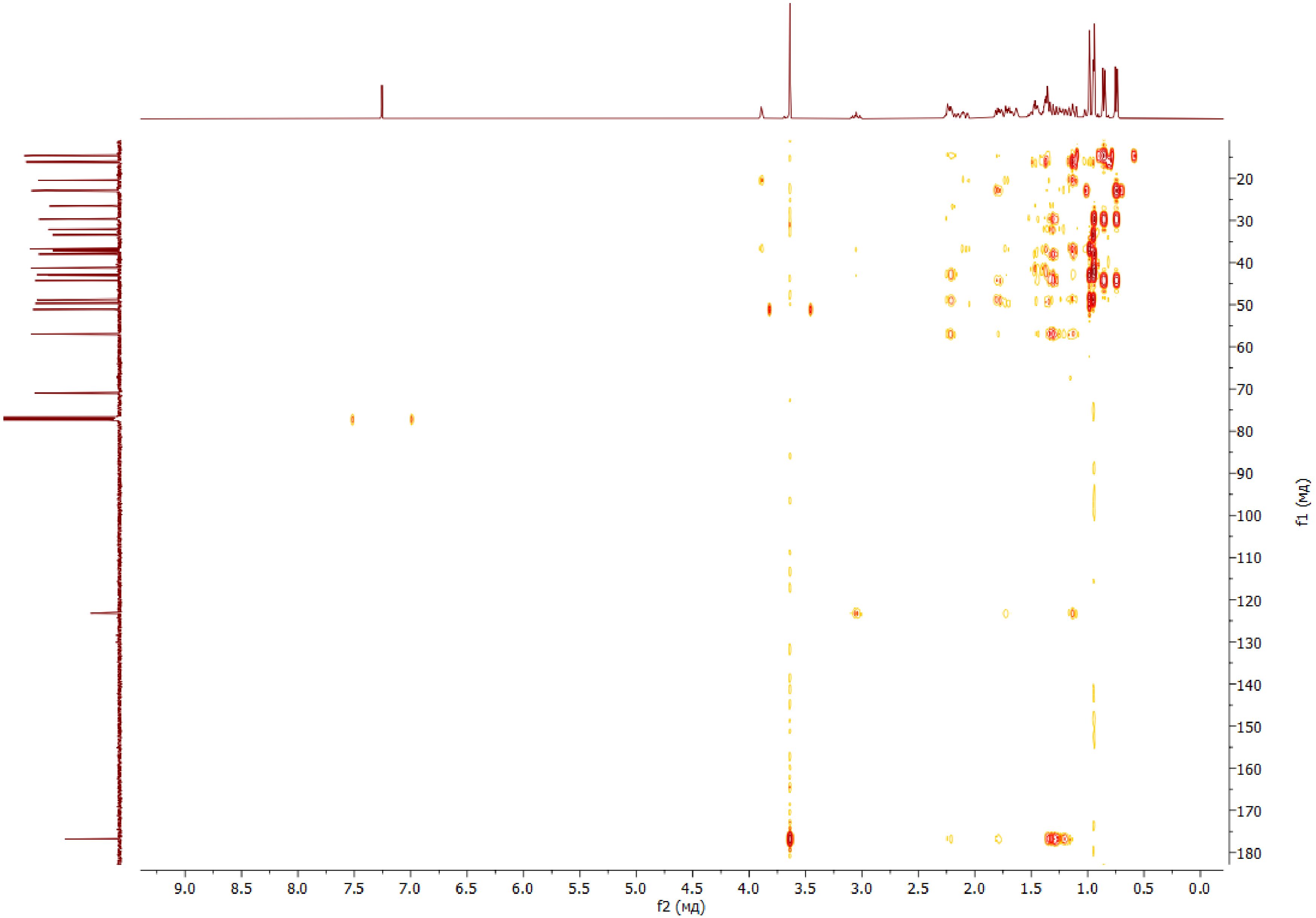

NOESY NMR spectra of 2-Cyano-4-hydroxy-3,23-dinorlupan-28-oic acid methyl ester (19).

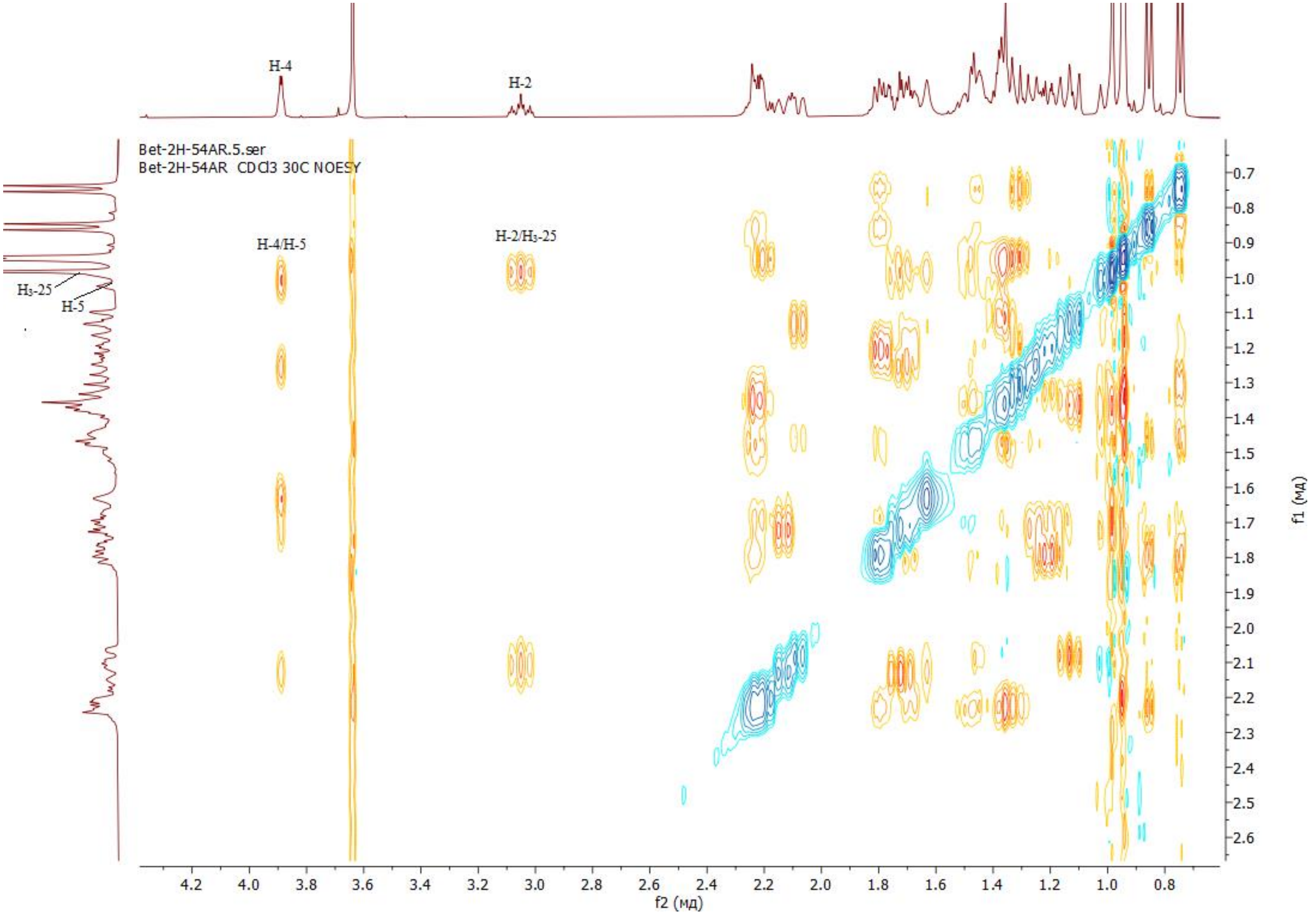

¹H NMR spectra of 28-Benzoyloxy-2-cyano-4-oxo-3,4-seco-3,23-dinorlupan (20)

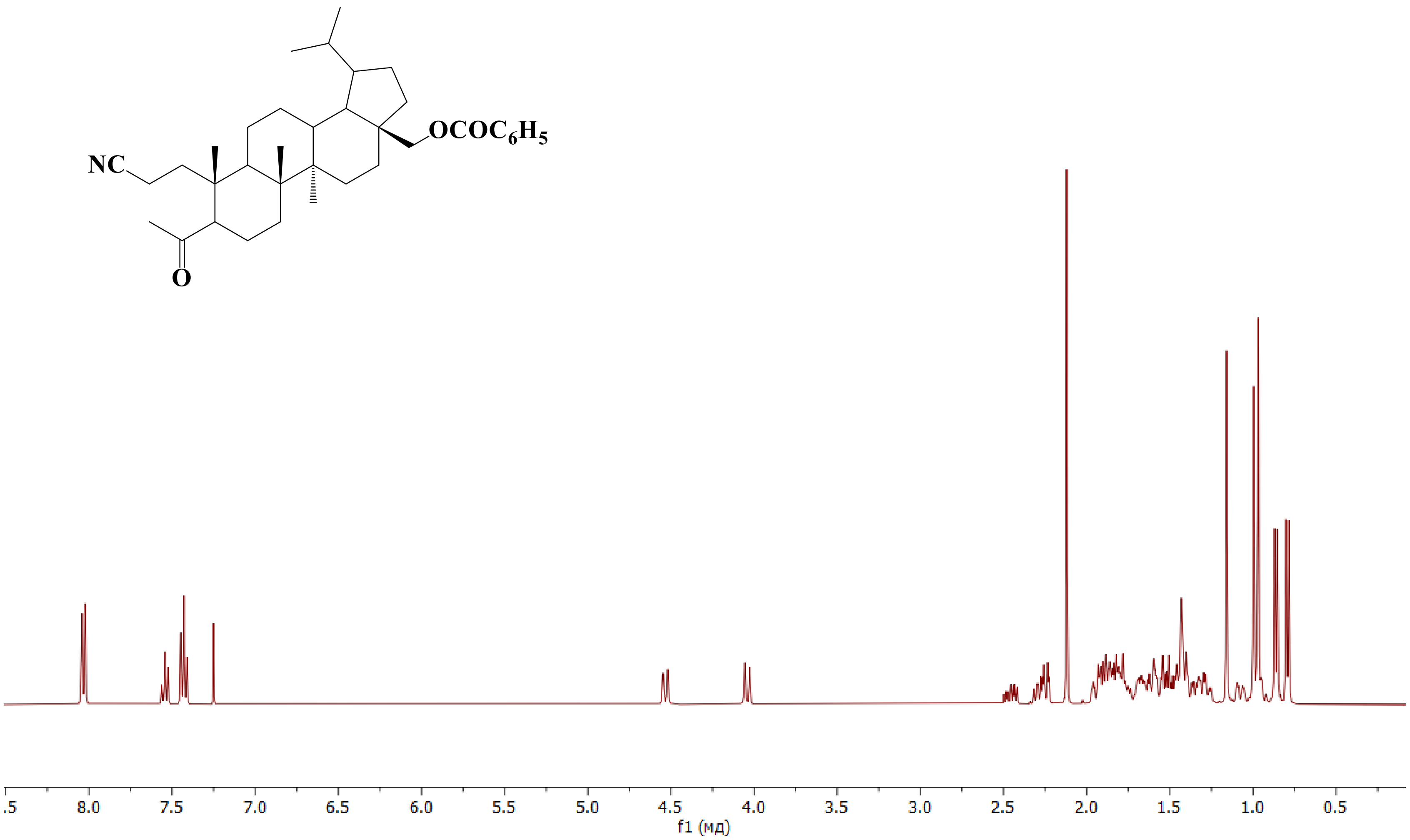

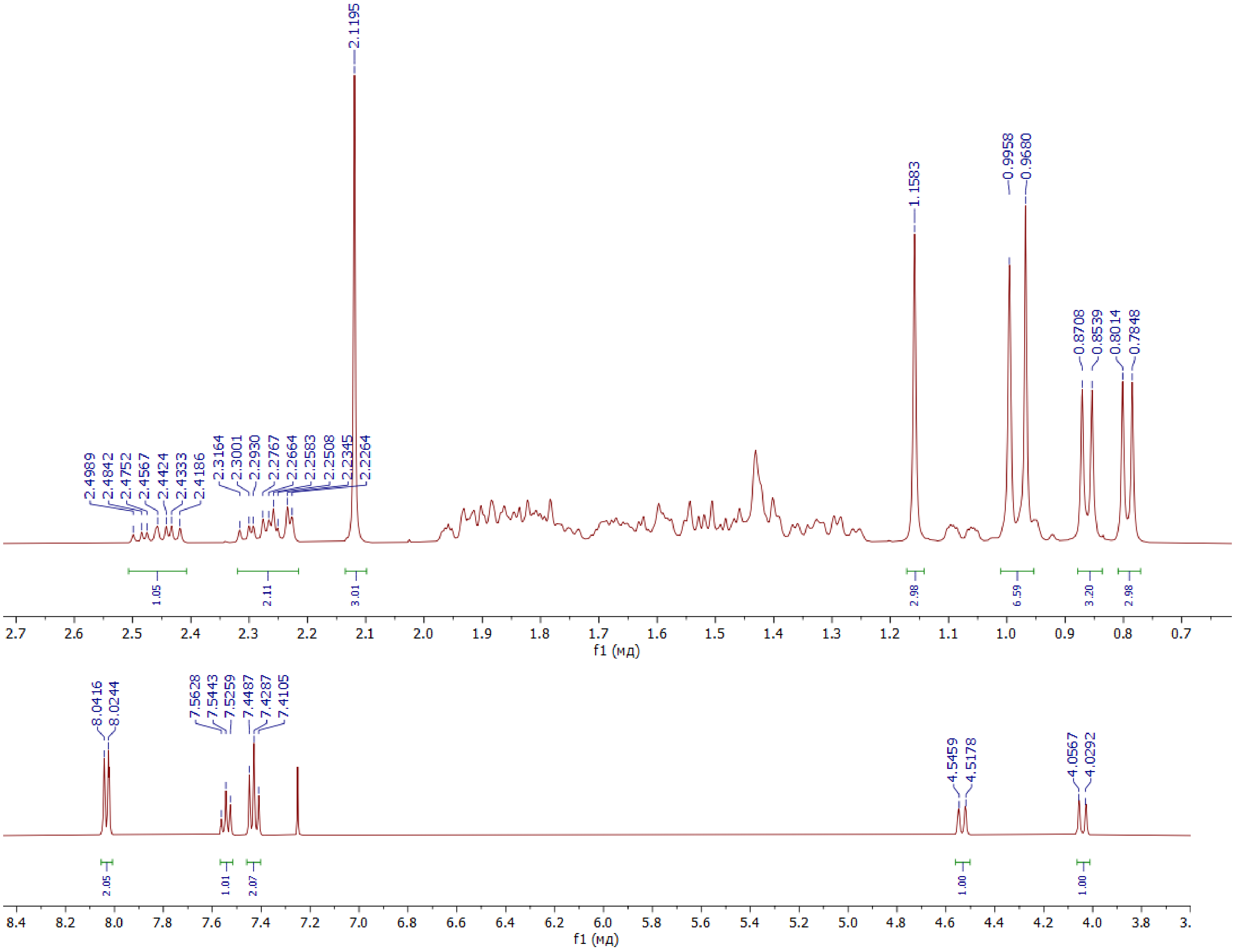

2.4989
2.4842
2.4752
2.4567
2.4424
2.4333
2.4186
2.3164
2.3001
2.2930
2.2767
2.2664
2.2583
2.2508
2.2345
2.2264
2.1195
1.1583
0.9958
0.9680
0.8708
0.8539
0.8014
0.7848
1.05
2.11
3.01
2.98
6.59
3.20
2.98
f1 (мд)
8.0416
8.0244
7.5628
7.5443
7.5259
7.4487
7.4287
7.4105
4.5459
4.5178
4.0567
4.0292
2.05
1.01
2.07
1.00
1.00
f1 (мд)

^{13}C NMR spectra of 28-Benzoyloxy-2-cyano-4-oxo-3,4-seco-3,23-dinorlupan (20)

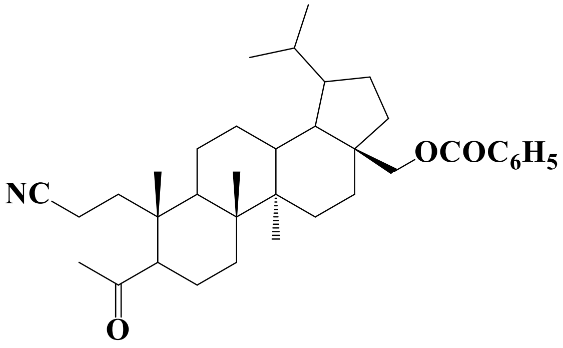

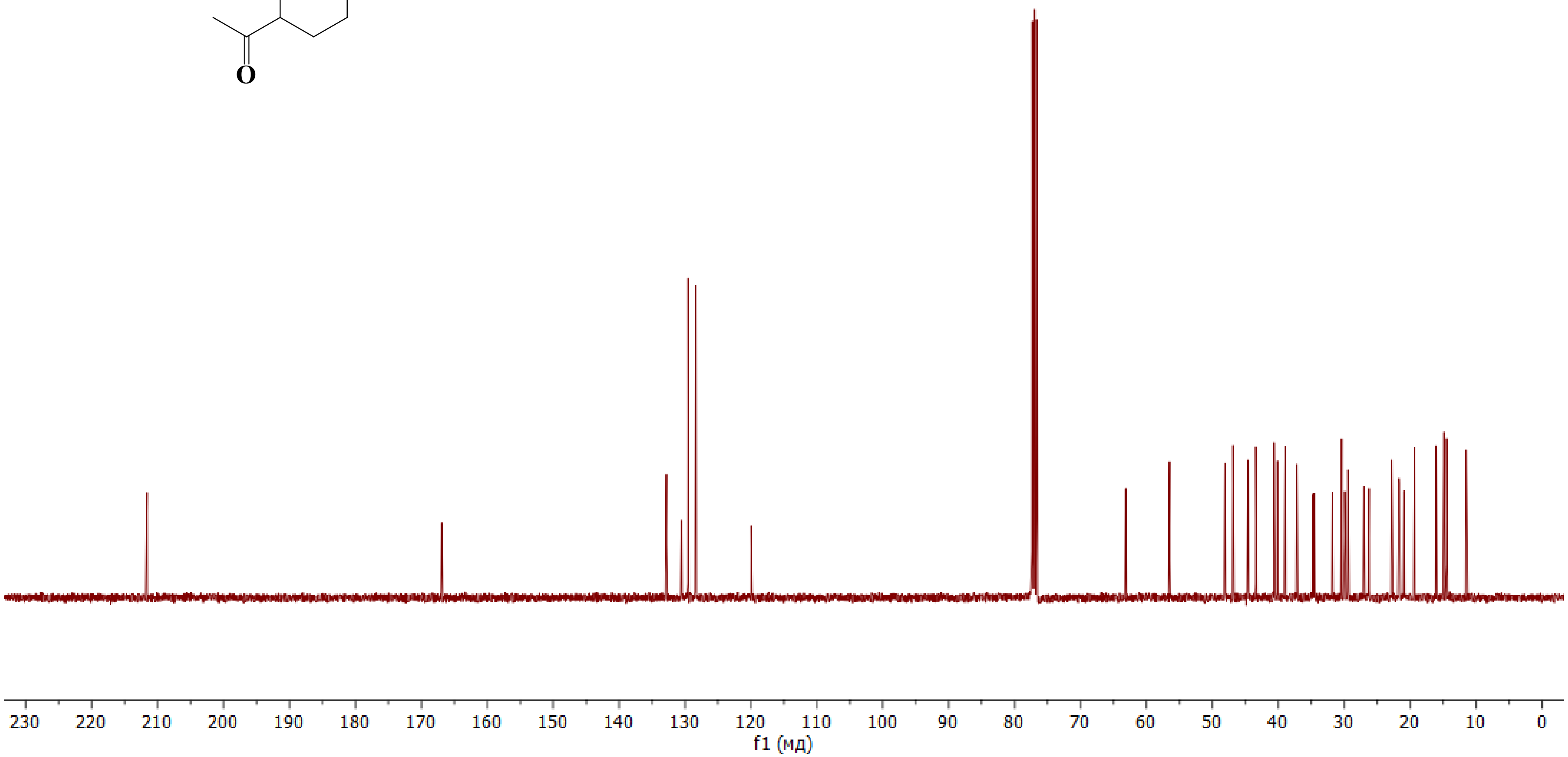

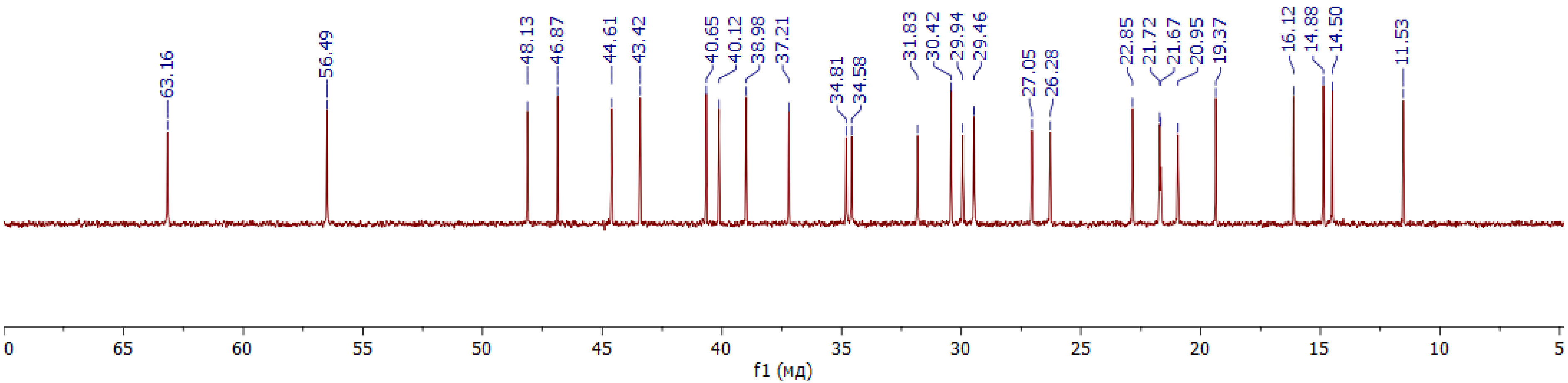
63.16
56.49
48.13
46.87
44.61
43.42
40.65
40.12
38.98
37.21
34.81
34.58
31.83
30.42
29.94
29.46
27.05
26.28
22.85
21.72
21.67
20.95
19.37
16.12
14.88
14.50
11.53
0 65 60 55 50 45 40 35 30 25 20 15 10 5
f1 (мд)

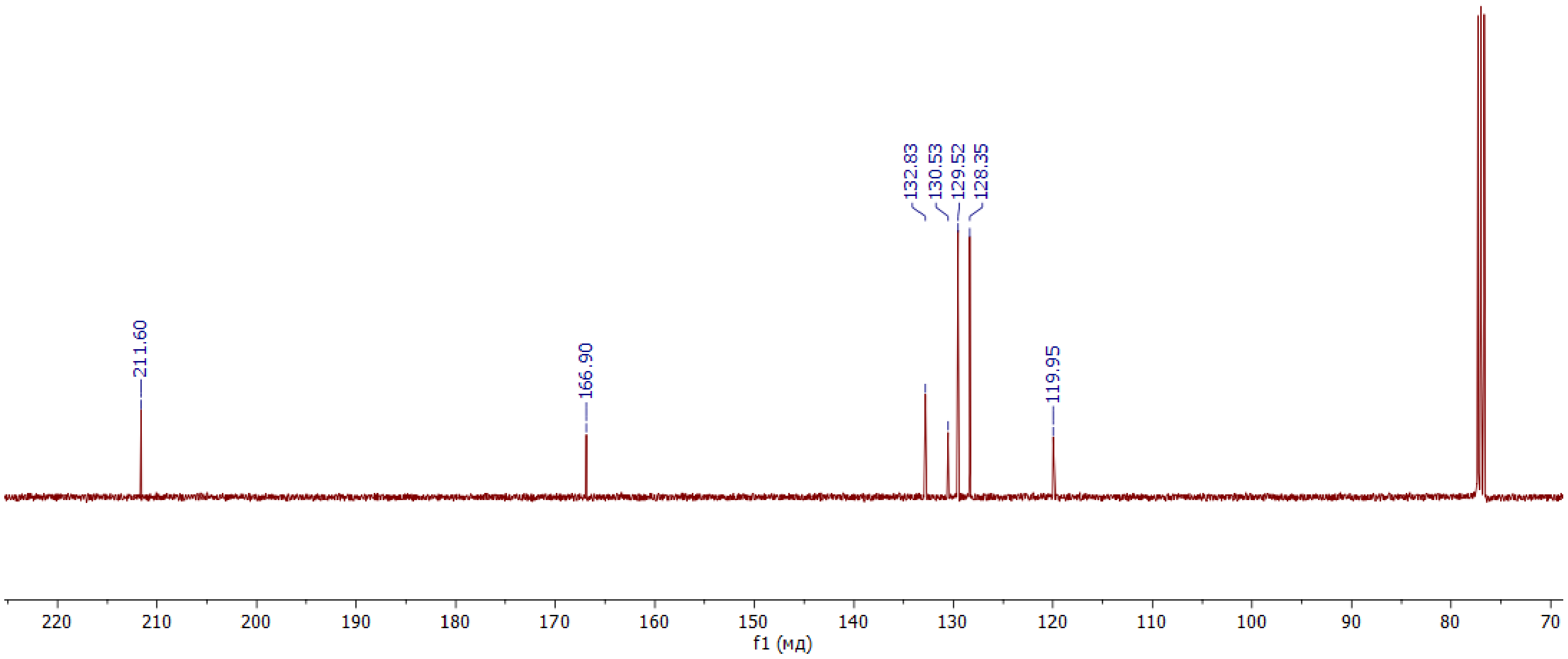
211.60
166.90
132.83
130.53
129.52
128.35
119.95
220 210 200 190 180 170 160 150 140 130 120 110 100 90 80 70
f1 (мд)

^{1}H NMR spectra of 2-Cyano-4-oxo-3,4-seco-3,23-dinorlupan-28-oic acid methyl ester (21)

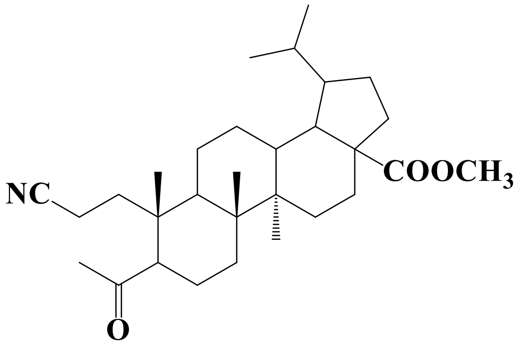

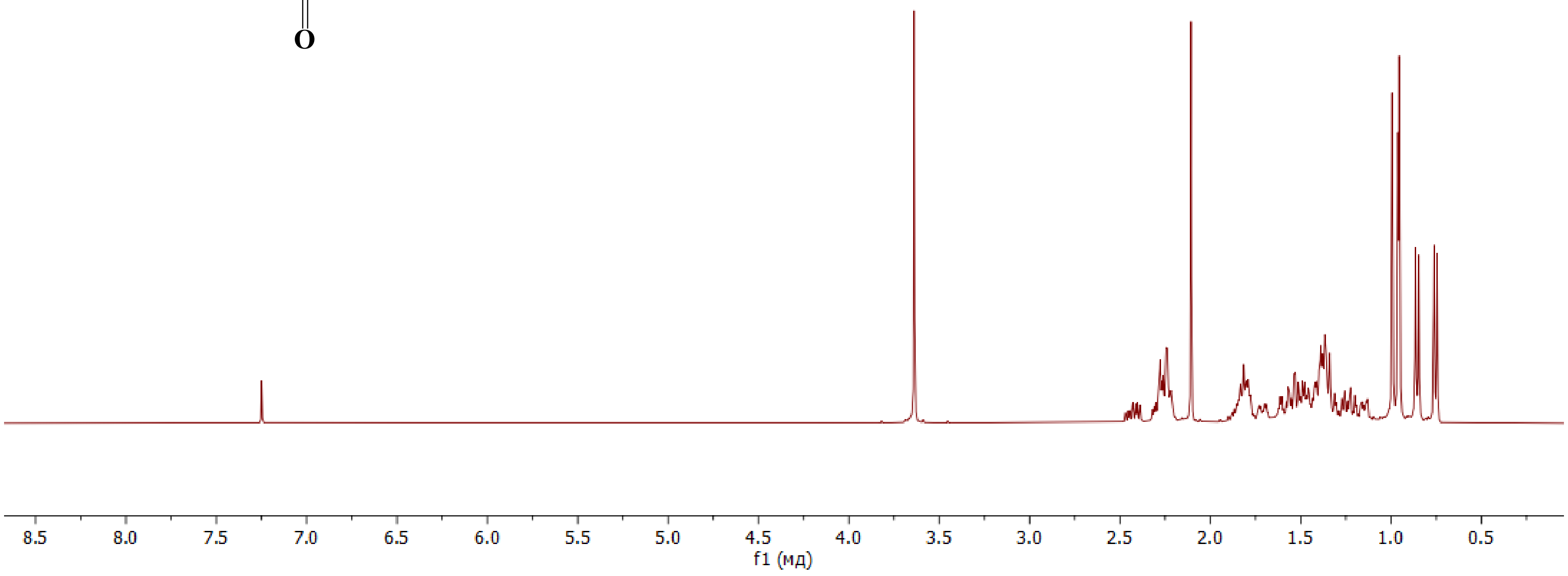

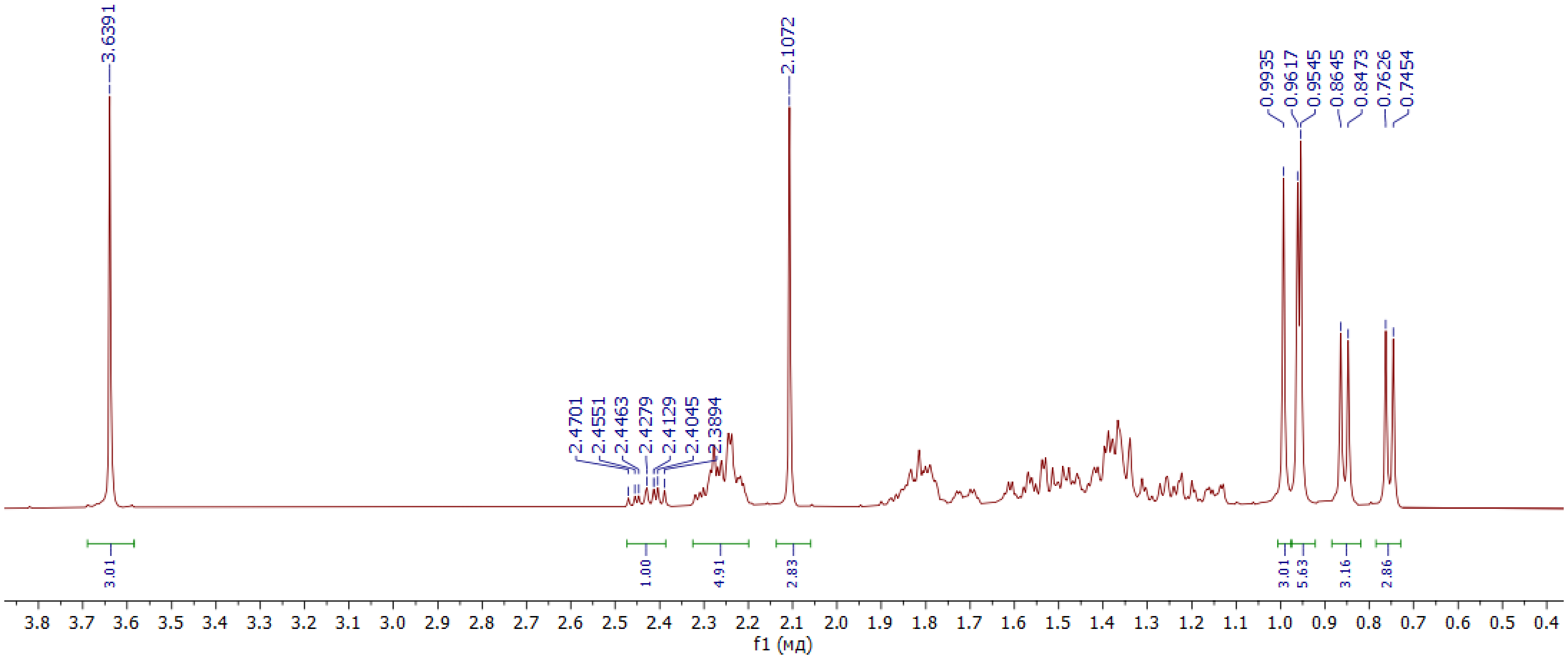

3.6391
2.1072
2.4701
2.4551
2.4463
2.4279
2.4129
2.4045
2.3894
0.9935
0.9617
0.9545
0.8645
0.8473
0.7626
0.7454
3.01
1.00
4.91
2.83
3.01
5.63
3.16
2.86
3.8 3.7 3.6 3.5 3.4 3.3 3.2 3.1 3.0 2.9 2.8 2.7 2.6 2.5 2.4 2.3 2.2 2.1 2.0 1.9 1.8 1.7 1.6 1.5 1.4 1.3 1.2 1.1 1.0 0.9 0.8 0.7 0.6 0.5 0.4
f1 (мд)

^{13}C NMR spectra of 2-Cyano-4-oxo-3,4-seco-3,23-dinorlupan-28-oic acid methyl ester (21)

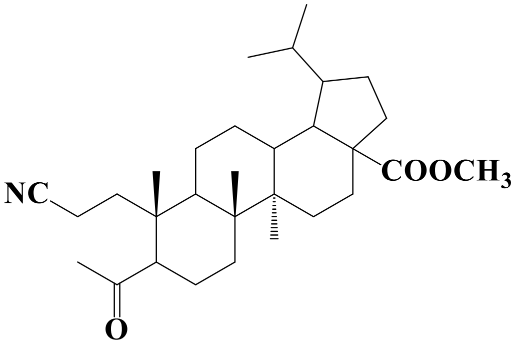

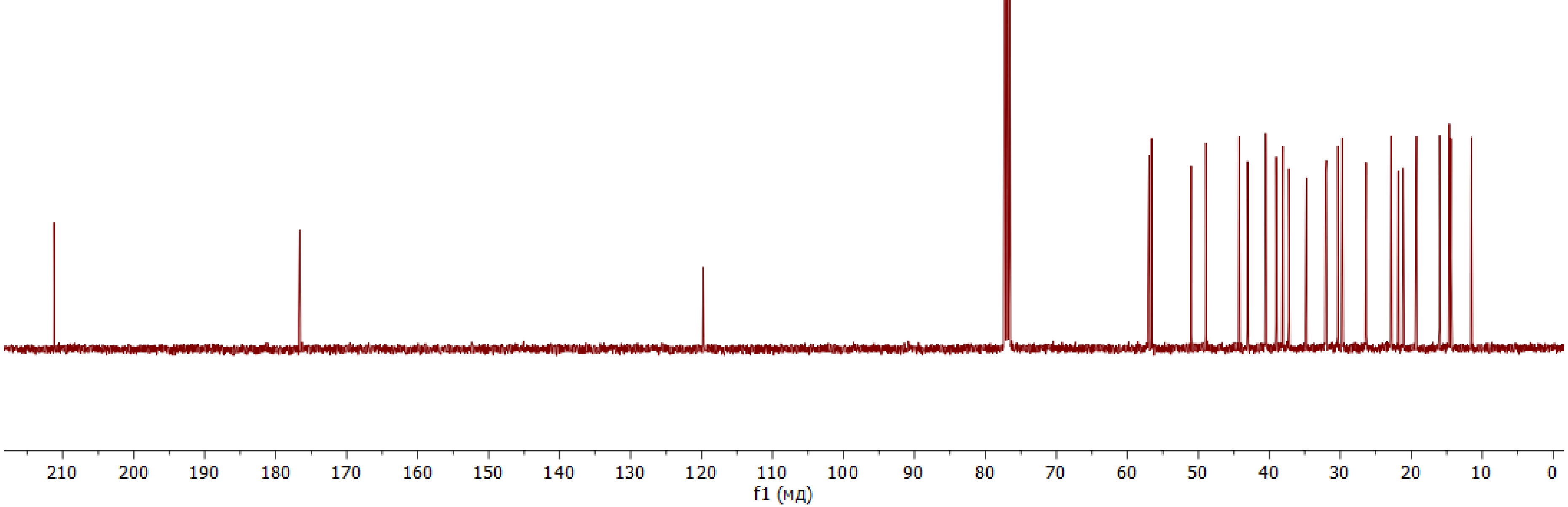

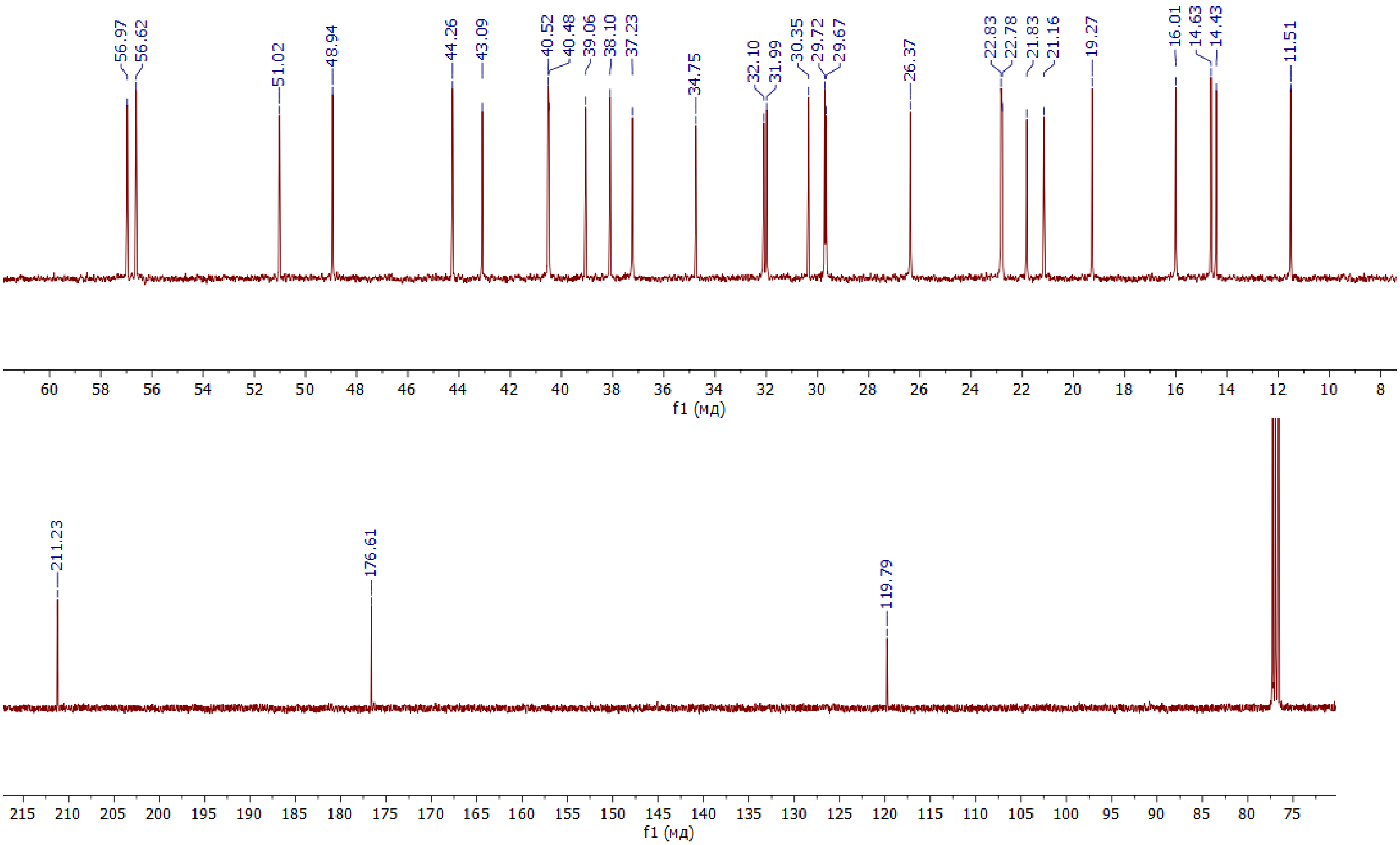
56.97
56.62
51.02
48.94
44.26
43.09
40.52
40.48
39.06
38.10
37.23
34.75
32.10
31.99
30.35
29.72
29.67
26.37
22.83
22.78
21.83
21.16
19.27
16.01
14.63
14.43
11.51
f1 (мд)
211.23
176.61
119.79
f1 (мд)

^{1}H NMR spectra of 2-Cyano-28-hydroxy-3,4-seco-3,23-dinor-2,4-cyclolup-2(4)-ene (22).

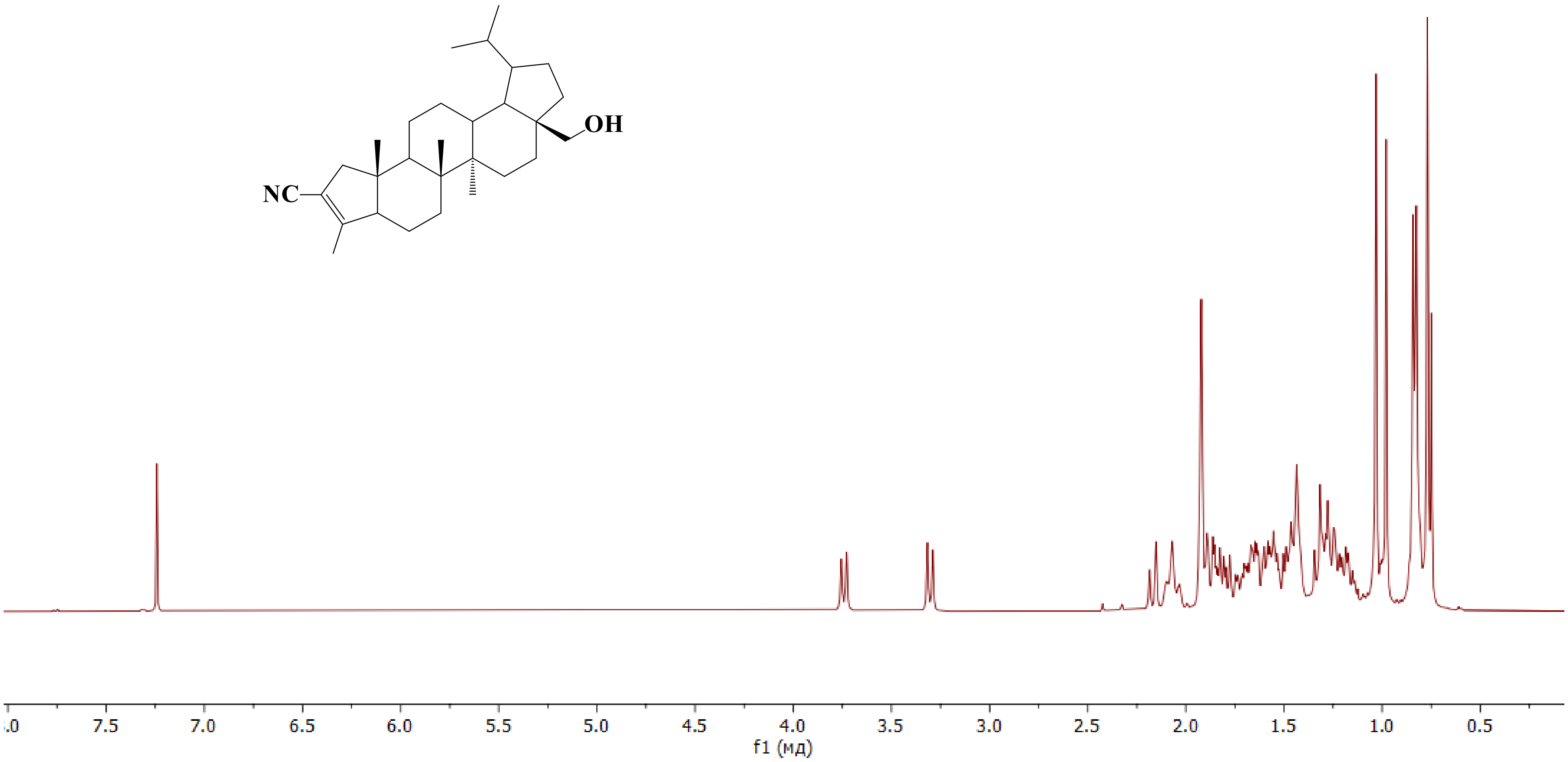

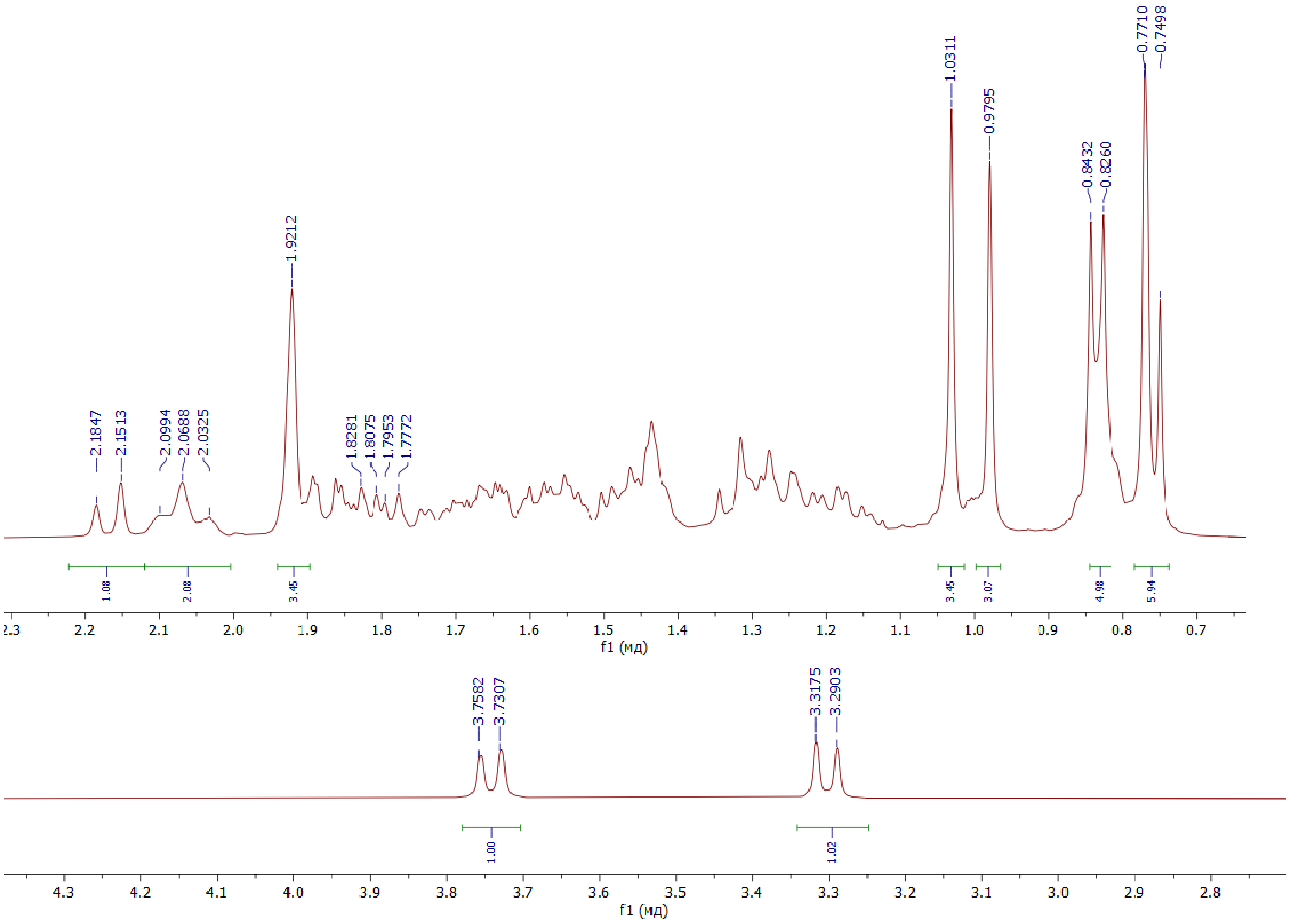
2.1847
2.1513
2.0994
2.0688
2.0325
1.9212
1.8281
1.8075
1.7953
1.7772
1.0311
0.9795
0.8432
0.8260
0.7710
0.7498
1.08
2.08
3.45
3.45
3.07
4.98
5.94
f1 (мд)
3.7582
3.7307
3.3175
3.2903
1.00
1.02
f1 (мд)

^{13}C NMR spectra of 2-Cyano-28-hydroxy-3,4-seco-3,23-dinor-2,4-cyclolup-2(4)-ene (22).

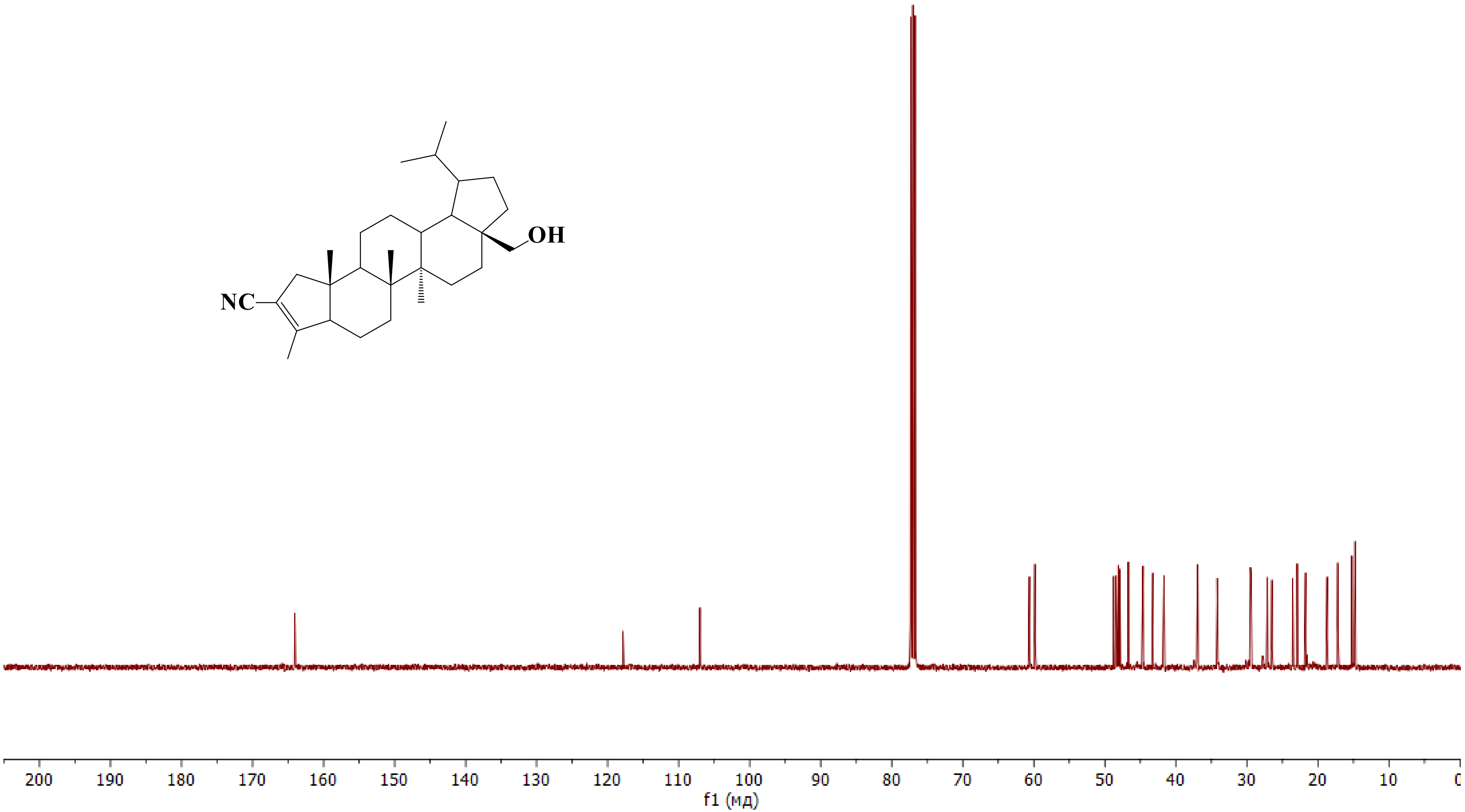

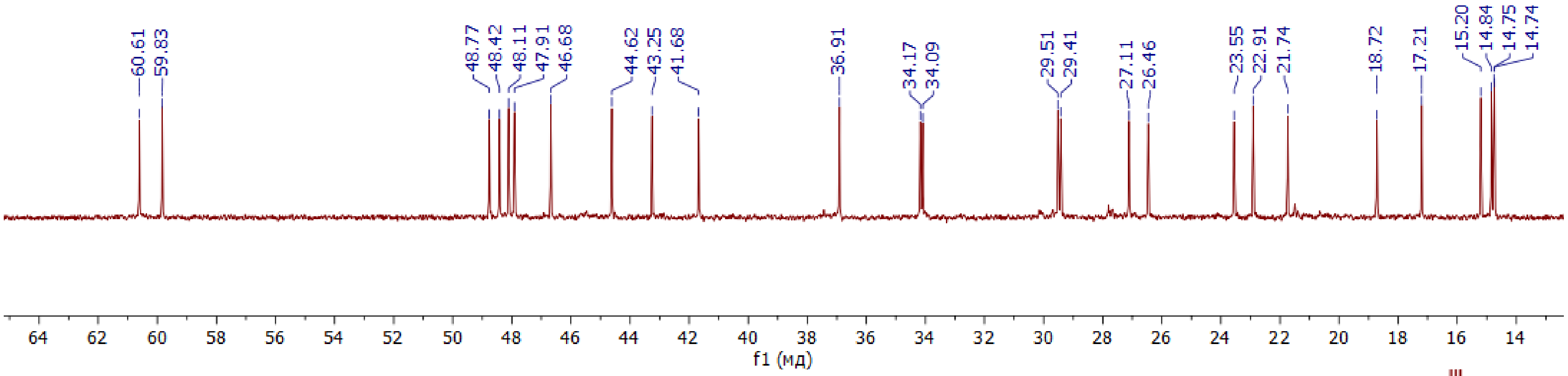
60.61
59.83
48.77
48.42
48.11
47.91
46.68
44.62
43.25
41.68
36.91
34.17
34.09
29.51
29.41
27.11
26.46
23.55
22.91
21.74
18.72
17.21
15.20
14.84
14.75
14.74
64 62 60 58 56 54 52 50 48 46 44 42 40 38 36 34 32 30 28 26 24 22 20 18 16 14
f1 (мд)

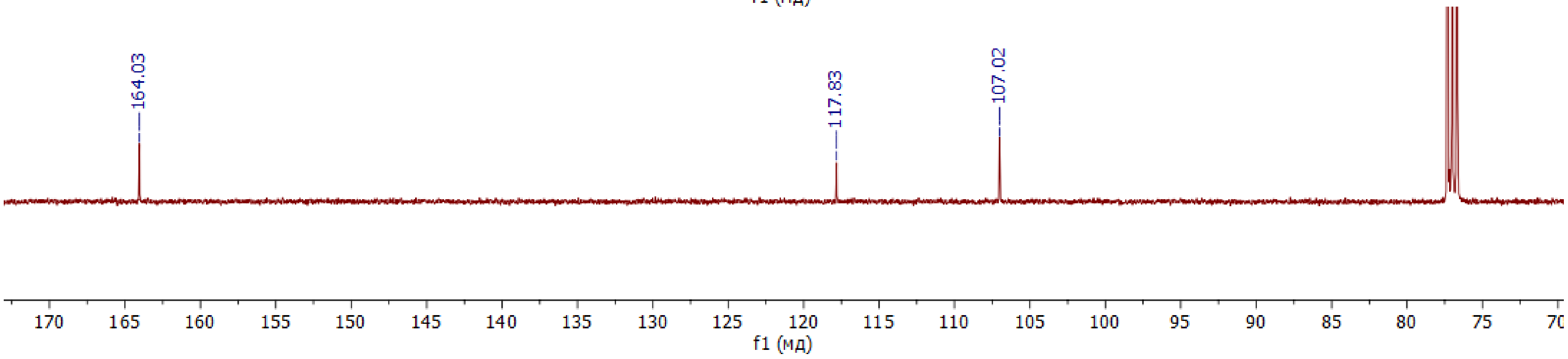
164.03
117.83
107.02
170 165 160 155 150 145 140 135 130 125 120 115 110 105 100 95 90 85 80 75 70
f1 (мд)

^{1}H NMR spectra of 2-Cyano-3,4-seco-3,23-dinor-2,4-cyclolup-2(4)-en-28-oic acid methyl ester (23).

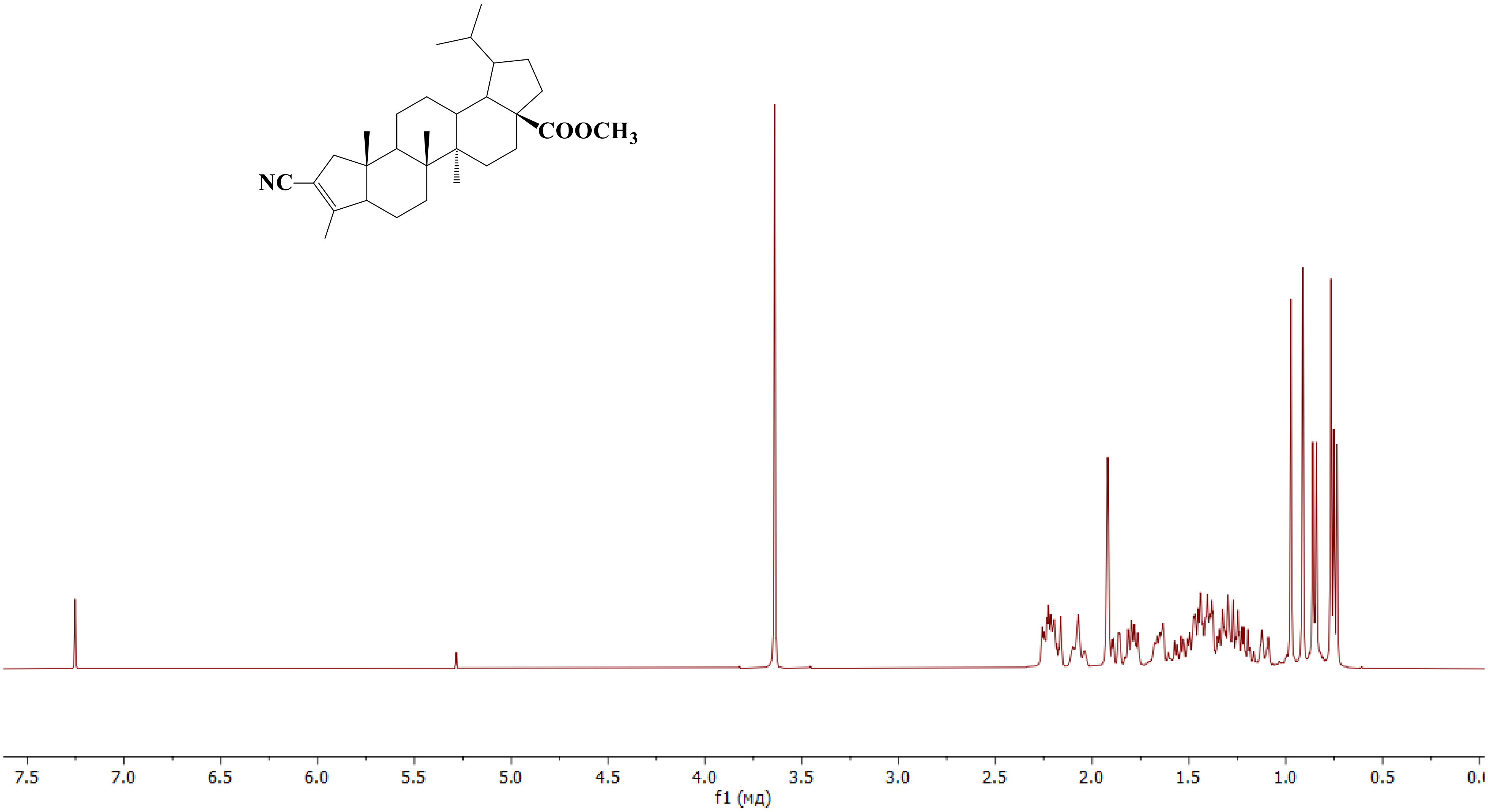

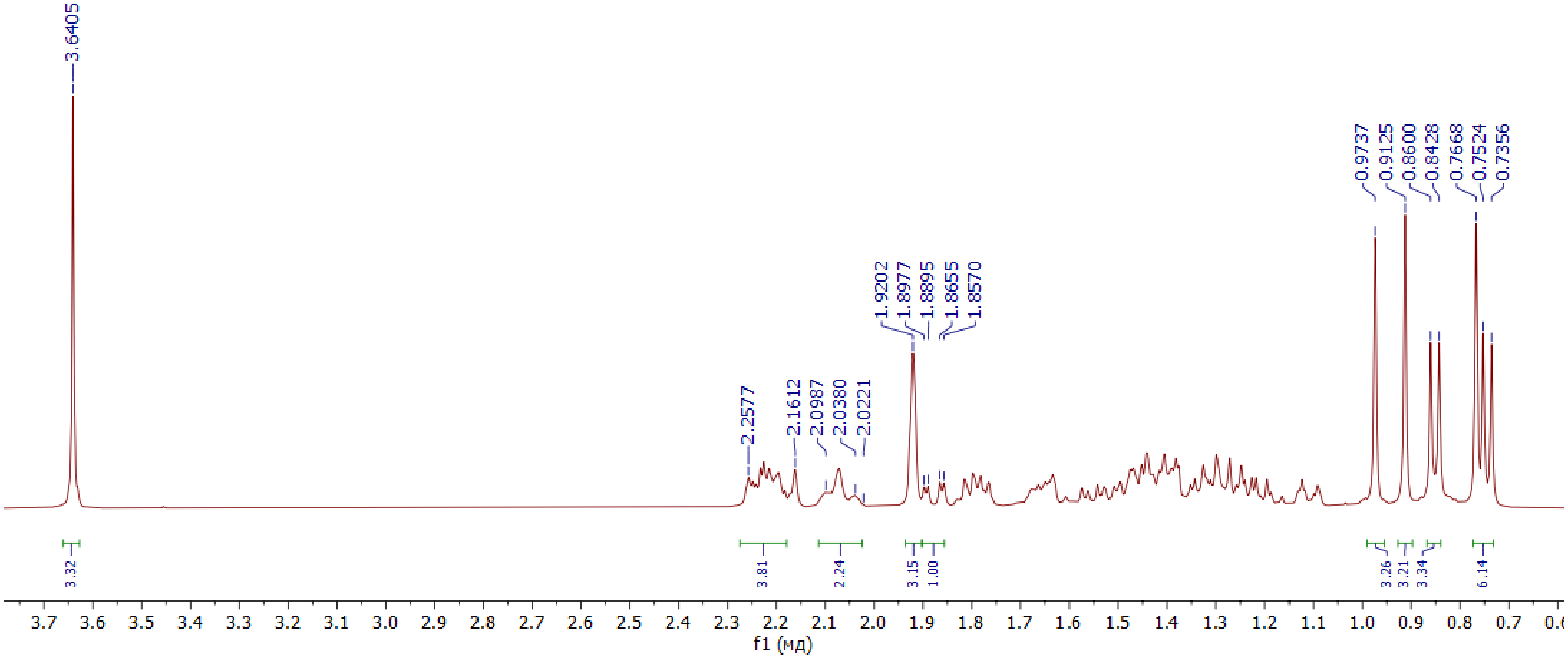
3.6405
2.2577
2.1612
2.0987
2.0380
2.0221
1.9202
1.8977
1.8895
1.8655
1.8570
0.9737
0.9125
0.8600
0.8428
0.7668
0.7524
0.7356
3.32
3.81
2.24
3.15
1.00
3.26
3.21
3.34
6.14
f1 (мд)

^{13}C NMR spectra of 2-Cyano-3,4-seco-3,23-dinor-2,4-cyclolup-2(4)-en-28-oic acid methyl ester (23).

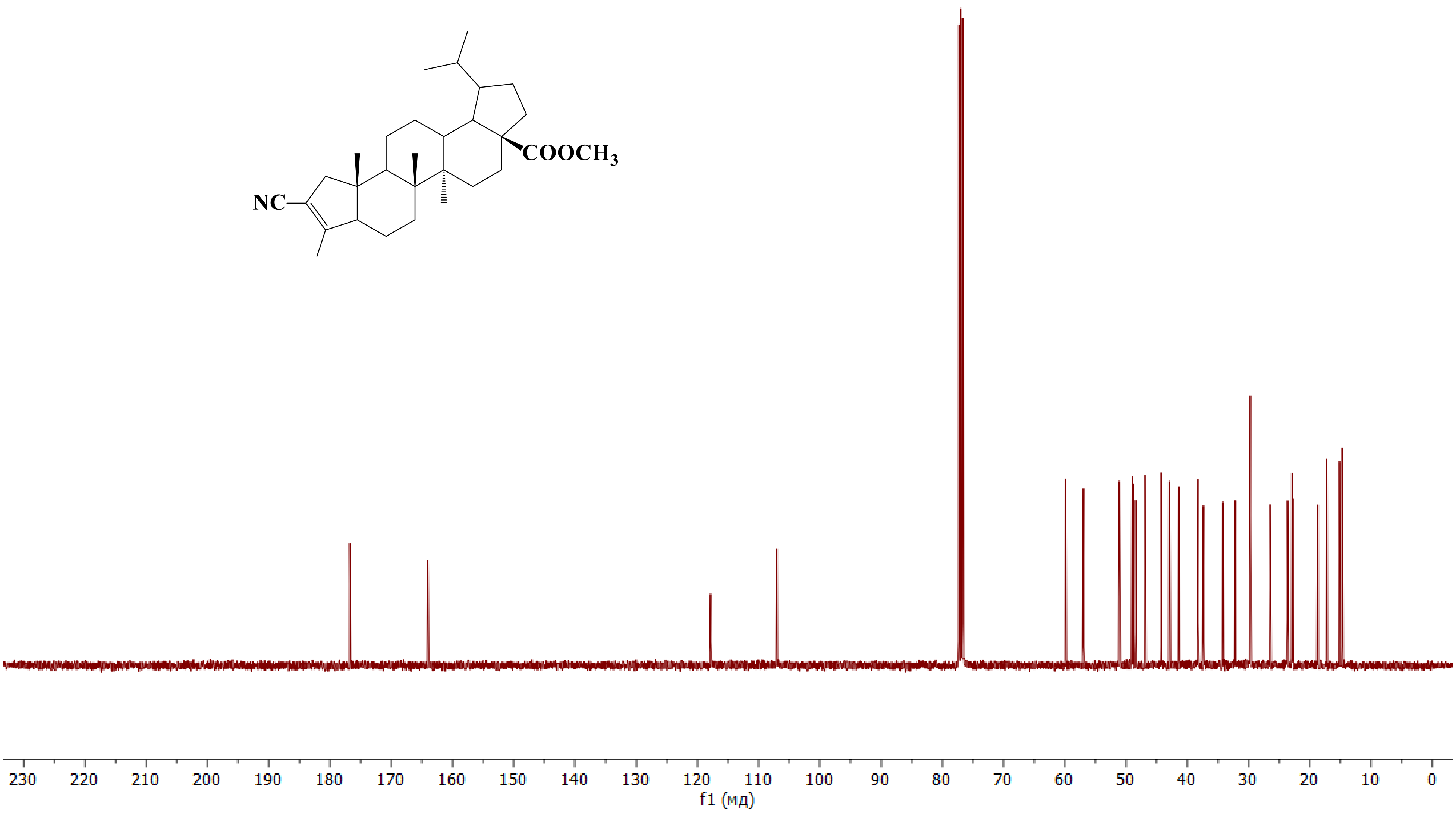

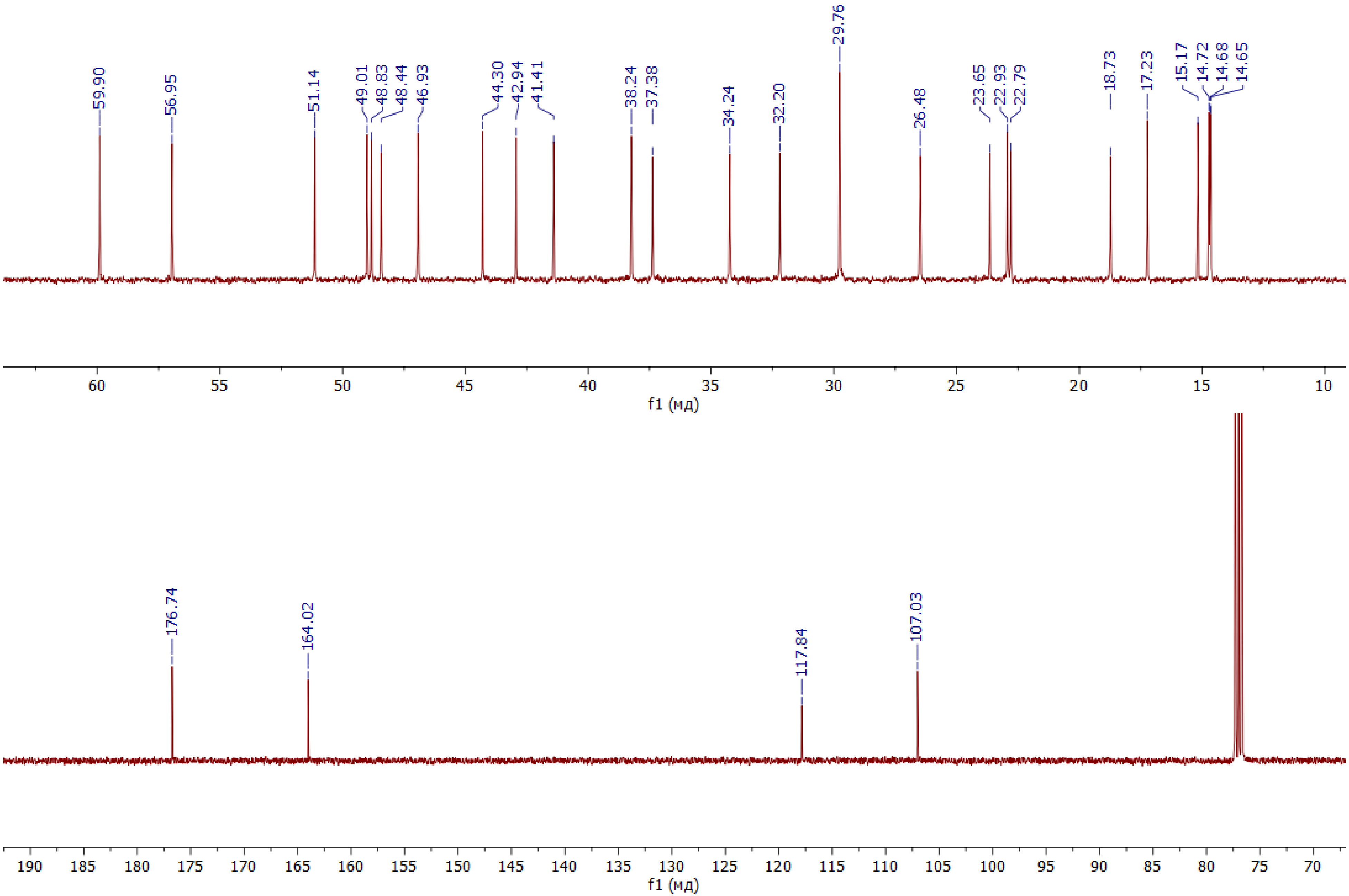
59.90
56.95
51.14
49.01
48.83
48.44
46.93
44.30
42.94
41.41
38.24
37.38
34.24
32.20
29.76
26.48
23.65
22.93
22.79
18.73
17.23
15.17
14.72
14.68
14.65
60 55 50 45 40 35 30 25 20 15 10
f1 (мд)
176.74
164.02
117.84
107.03
190 185 180 175 170 165 160 155 150 145 140 135 130 125 120 115 110 105 100 95 90 85 80 75 70
f1 (мд)

^{1}H NMR spectra of 28-Benzoyloxy-24-bromo-2-cyano-4-oxo-3,4-seco-3,23-dinorlupan (28).

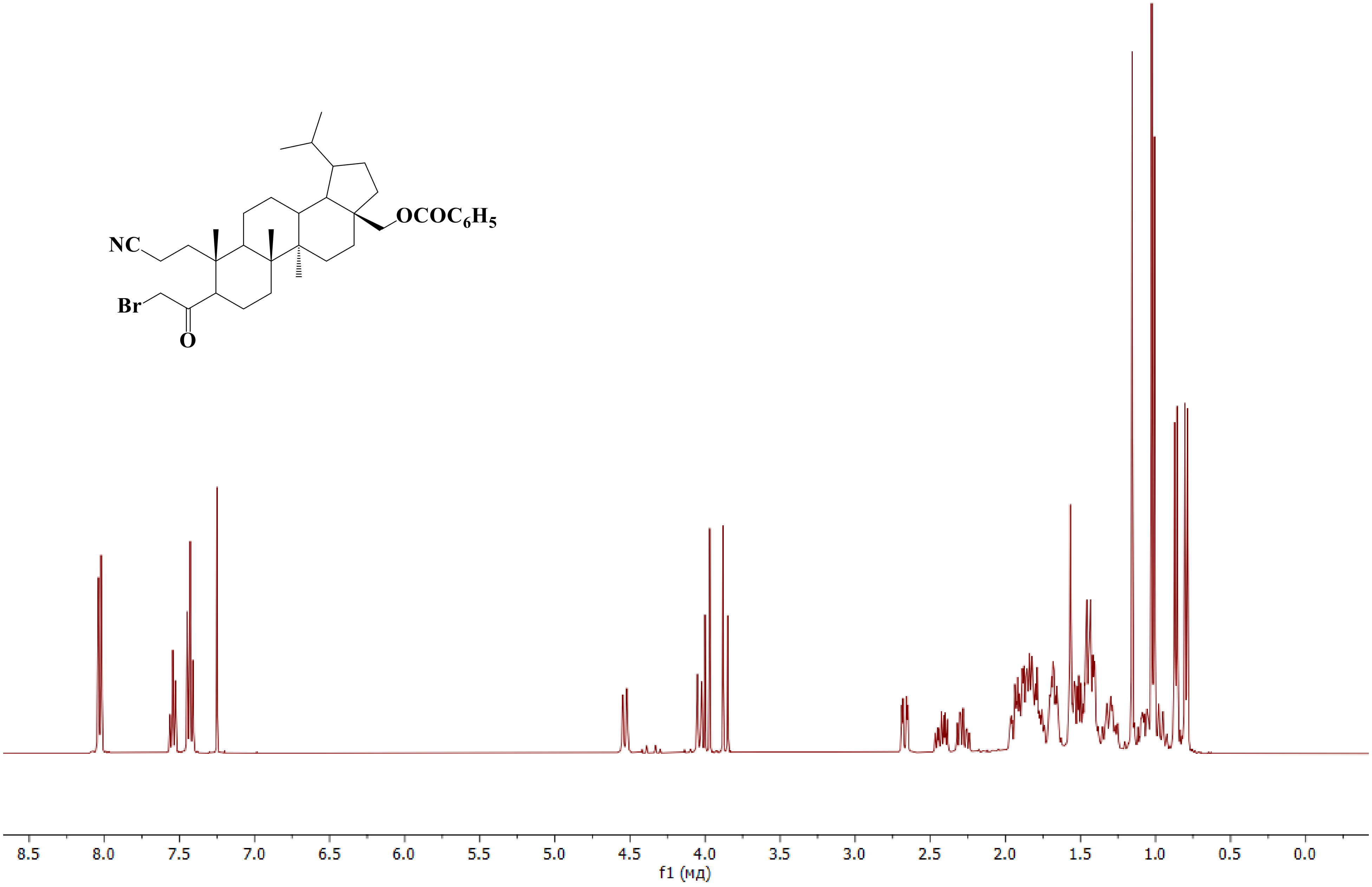

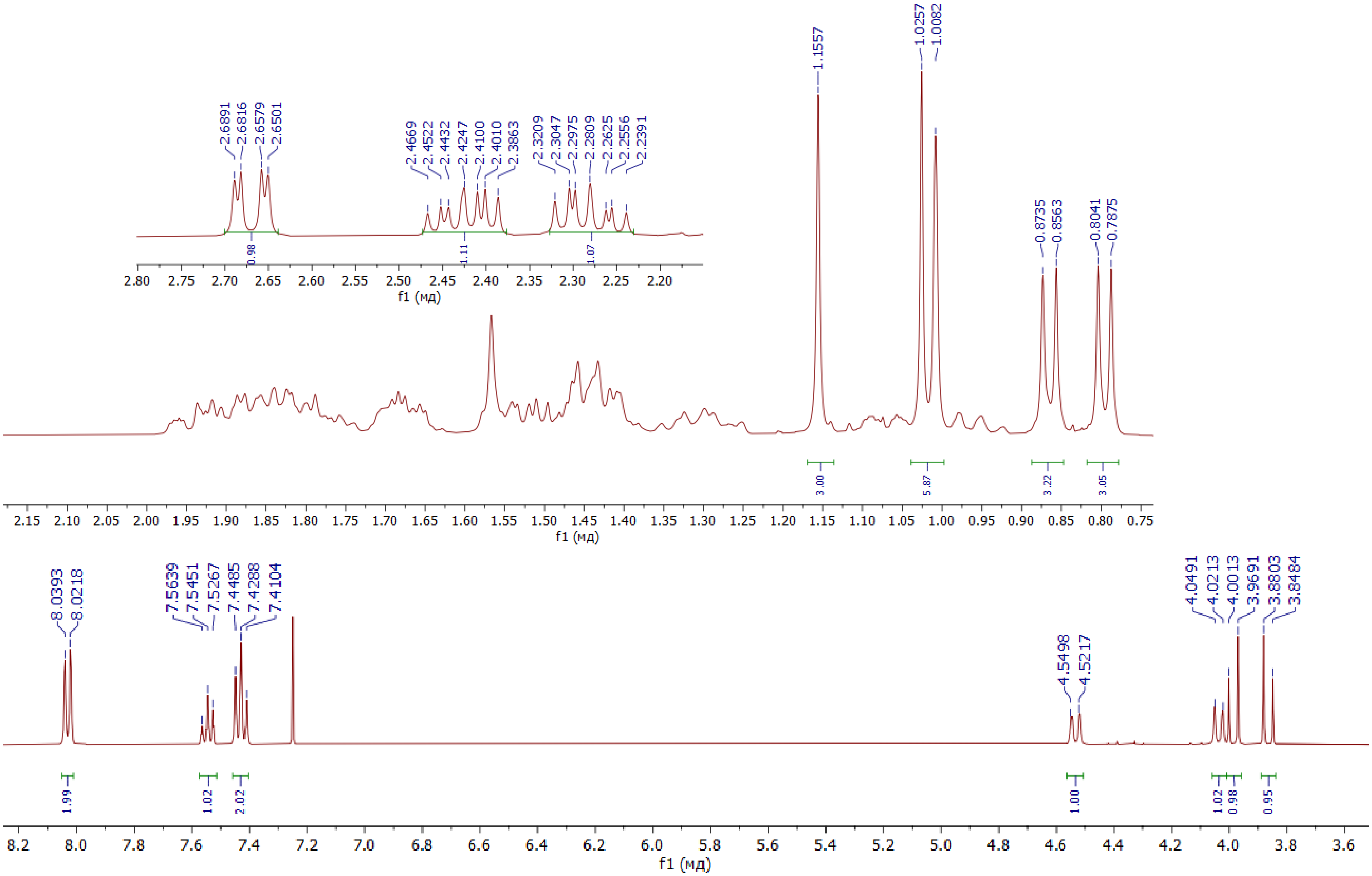
2.6891
2.6816
2.6579
2.6501
2.4669
2.4522
2.4432
2.4247
2.4100
2.4010
2.3863
2.3209
2.3047
2.2975
2.2809
2.2625
2.2556
2.2391
0.98
1.11
1.07
f1 (мд)
1.1557
1.0257
1.0082
0.8735
0.8563
0.8041
0.7875
3.00
5.87
3.22
3.05
f1 (мд)
8.0393
8.0218
7.5639
7.5451
7.5267
7.4485
7.4288
7.4104
4.5498
4.5217
4.0491
4.0213
4.0013
3.9691
3.8803
3.8484
1.99
1.02
2.02
1.00
1.02
0.98
0.95
f1 (мд)

^{13}C NMR spectra of 28-Benzoyloxy-24-bromo-2-cyano-4-oxo-3,4-seco-3,23-dinorlupan (28).

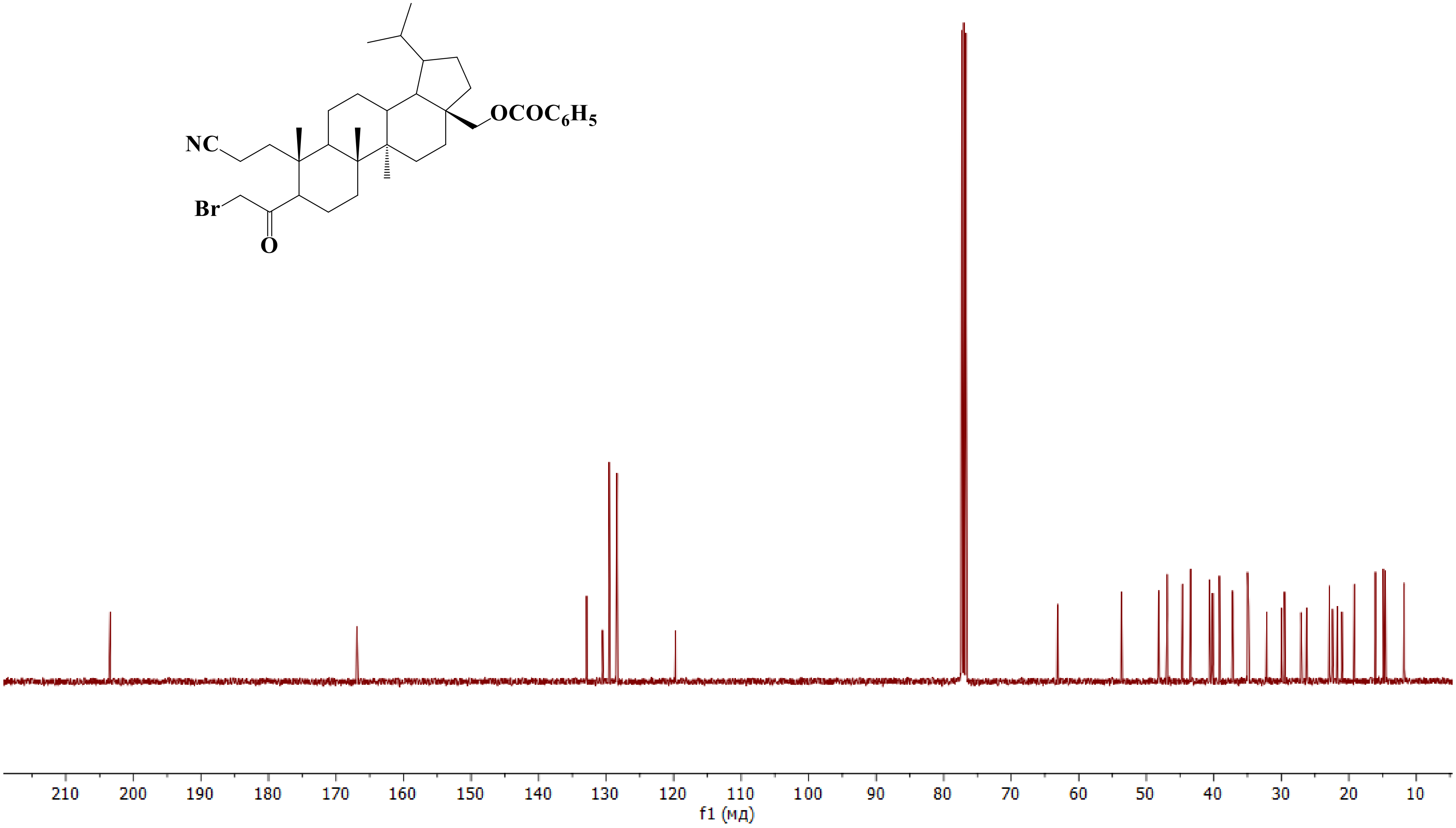

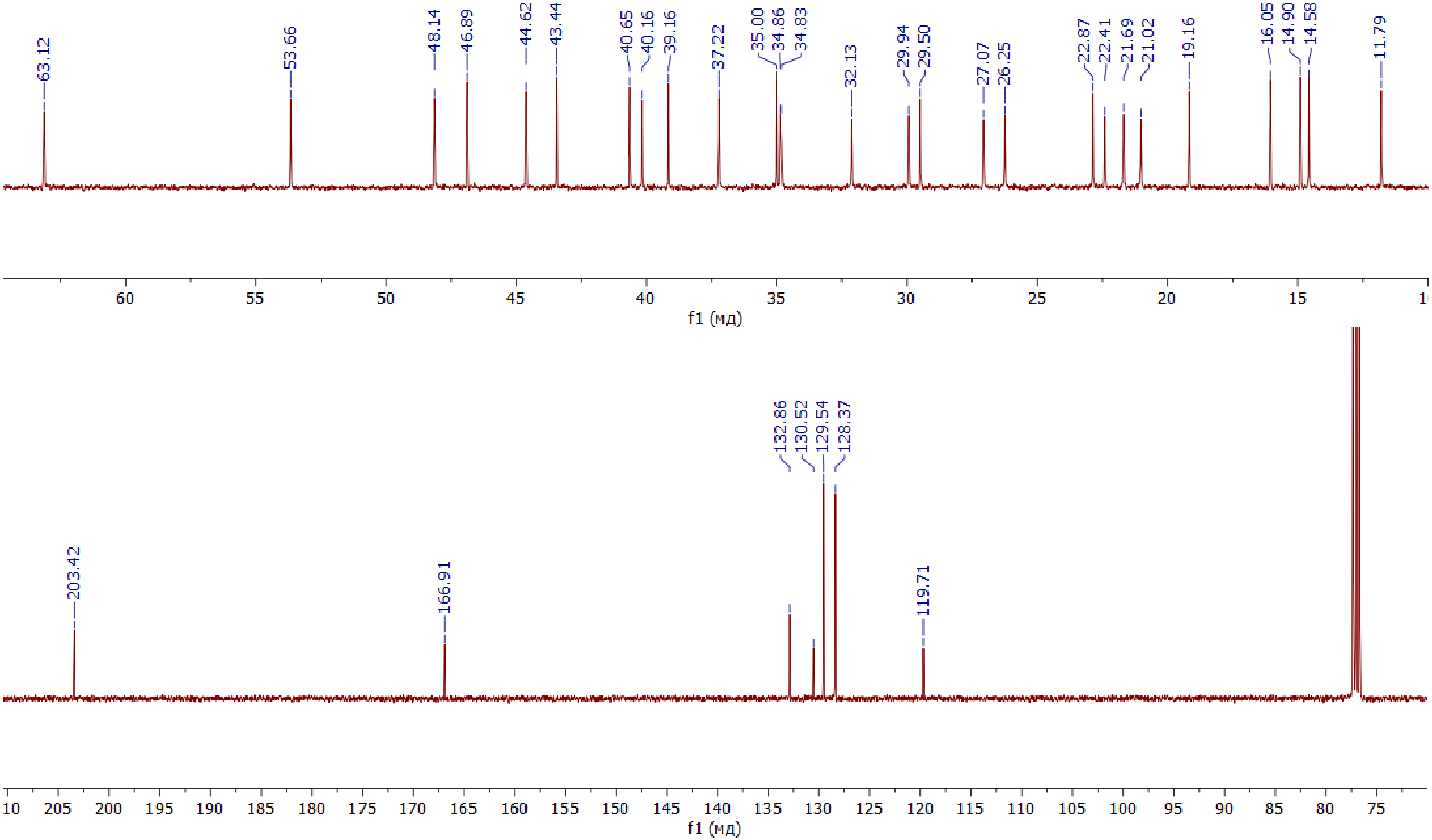
63.12
53.66
48.14
46.89
44.62
43.44
40.65
40.16
39.16
37.22
35.00
34.86
34.83
32.13
29.94
29.50
27.07
26.25
22.87
22.41
21.69
21.02
19.16
16.05
14.90
14.58
11.79
f1 (мд)
203.42
166.91
132.86
130.52
129.54
128.37
119.71
f1 (мд)

^{1}H NMR spectra of 28-Benzoyloxy-2-cyano-24-dibromo-4-oxo-3,4-seco-3,23-dinorlupan (29).

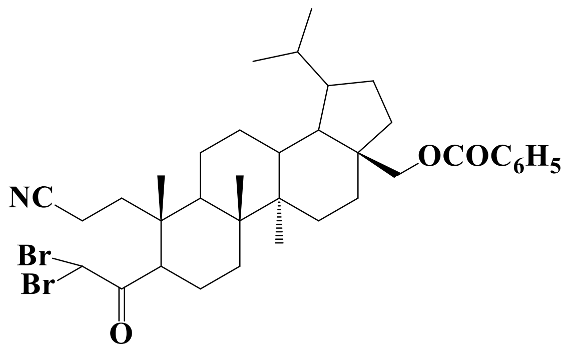

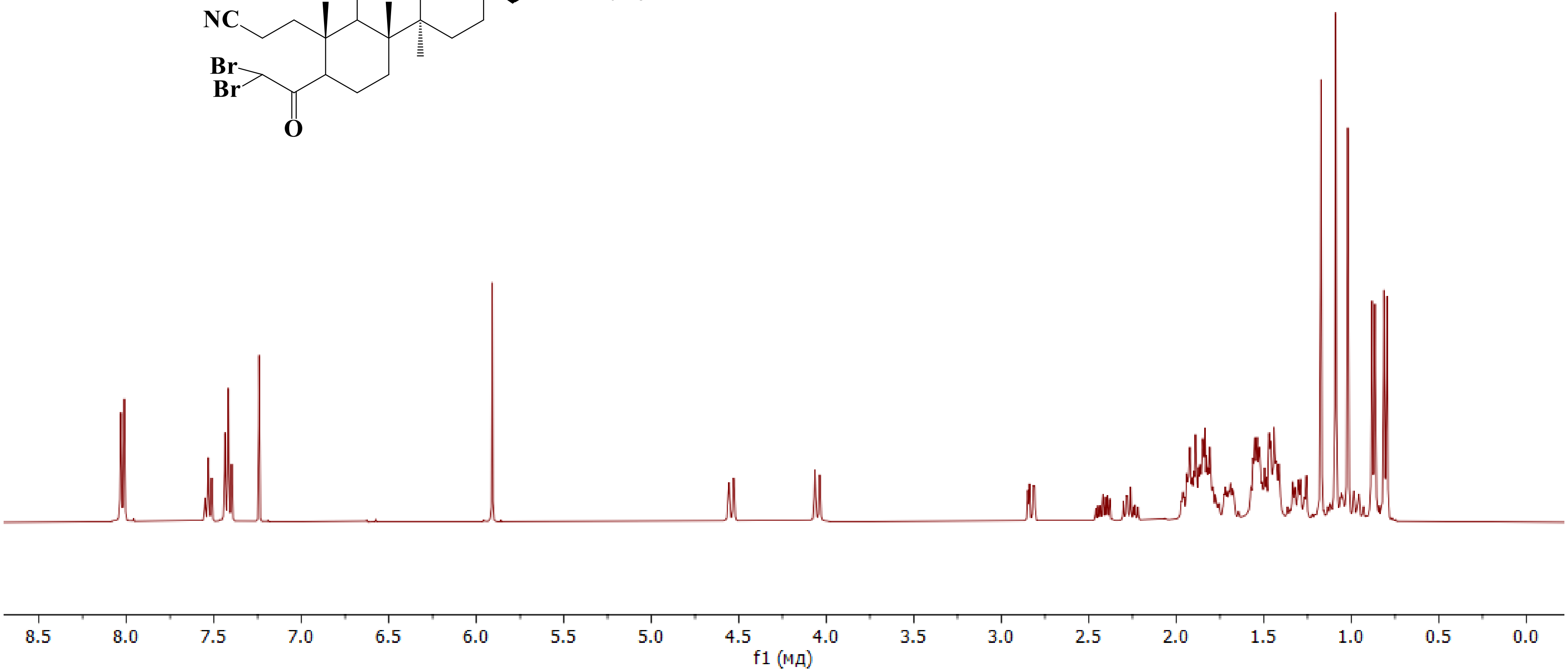

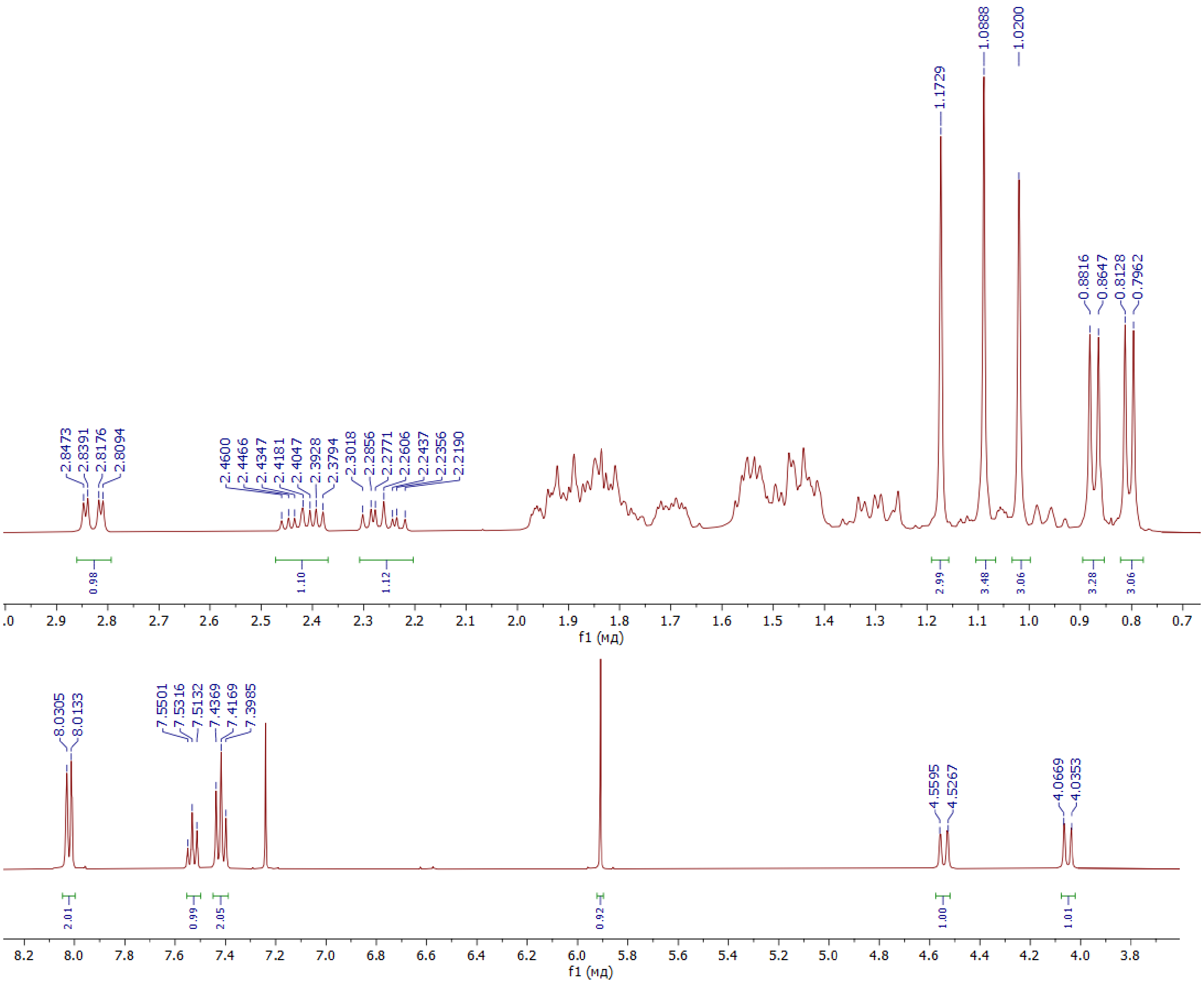

2.8473
2.8391
2.8176
2.8094
2.4600
2.4466
2.4347
2.4181
2.4047
2.3928
2.3794
2.3018
2.2856
2.2771
2.2606
2.2437
2.2356
2.2190
1.1729
1.0888
1.0200
0.8816
0.8647
0.8128
0.7962
0.98
1.10
1.12
2.99
3.48
3.06
3.28
3.06
.0 2.9 2.8 2.7 2.6 2.5 2.4 2.3 2.2 2.1 2.0 1.9 1.8 1.7 1.6 1.5 1.4 1.3 1.2 1.1 1.0 0.9 0.8 0.7
f1 (мд)
8.0305
8.0133
7.5501
7.5316
7.5132
7.4369
7.4169
7.3985
4.5595
4.5267
4.0669
4.0353
2.01
0.99
2.05
0.92
1.00
1.01
8.2 8.0 7.8 7.6 7.4 7.2 7.0 6.8 6.6 6.4 6.2 6.0 5.8 5.6 5.4 5.2 5.0 4.8 4.6 4.4 4.2 4.0 3.8
f1 (мд)

13C NMR spectra of 28-Benzoyloxy-2-cyano-24-dibromo-4-oxo-3,4-seco-3,23-dinorlupan (29).

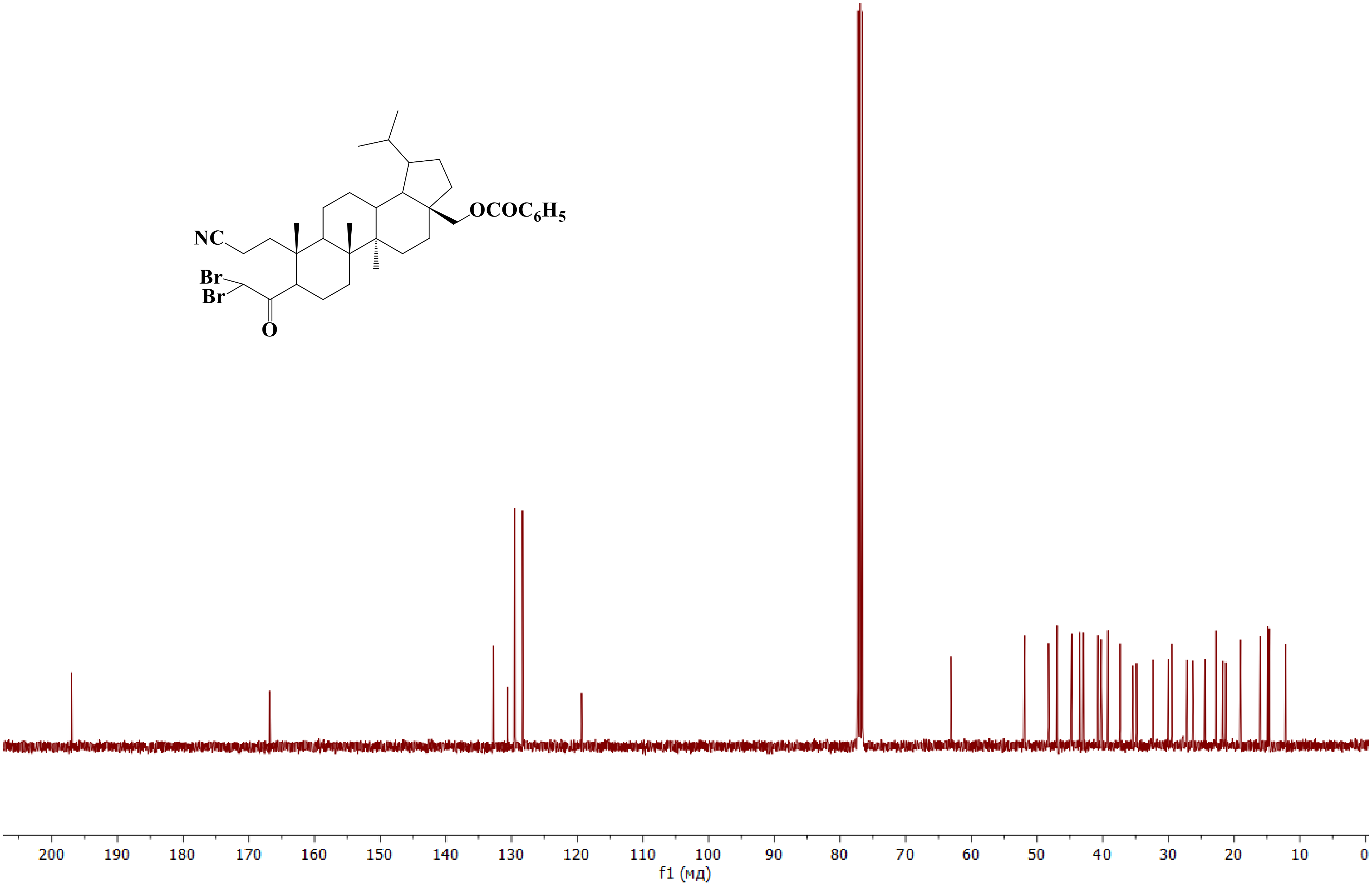

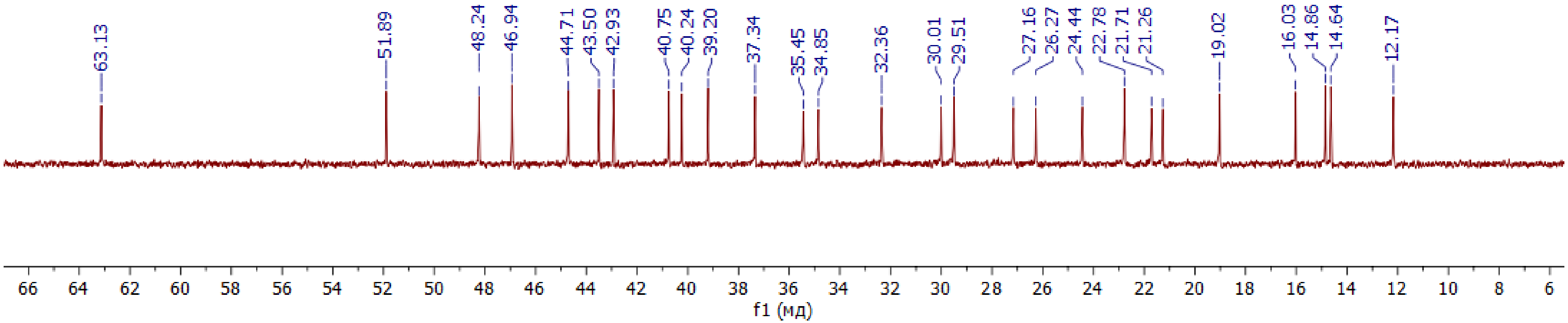
63.13
51.89
48.24
46.94
44.71
43.50
42.93
40.75
40.24
39.20
37.34
35.45
34.85
32.36
30.01
29.51
27.16
26.27
24.44
22.78
21.71
21.26
19.02
16.03
14.86
14.64
12.17
66 64 62 60 58 56 54 52 50 48 46 44 42 40 38 36 34 32 30 28 26 24 22 20 18 16 14 12 10 8 6
f1 (мд)

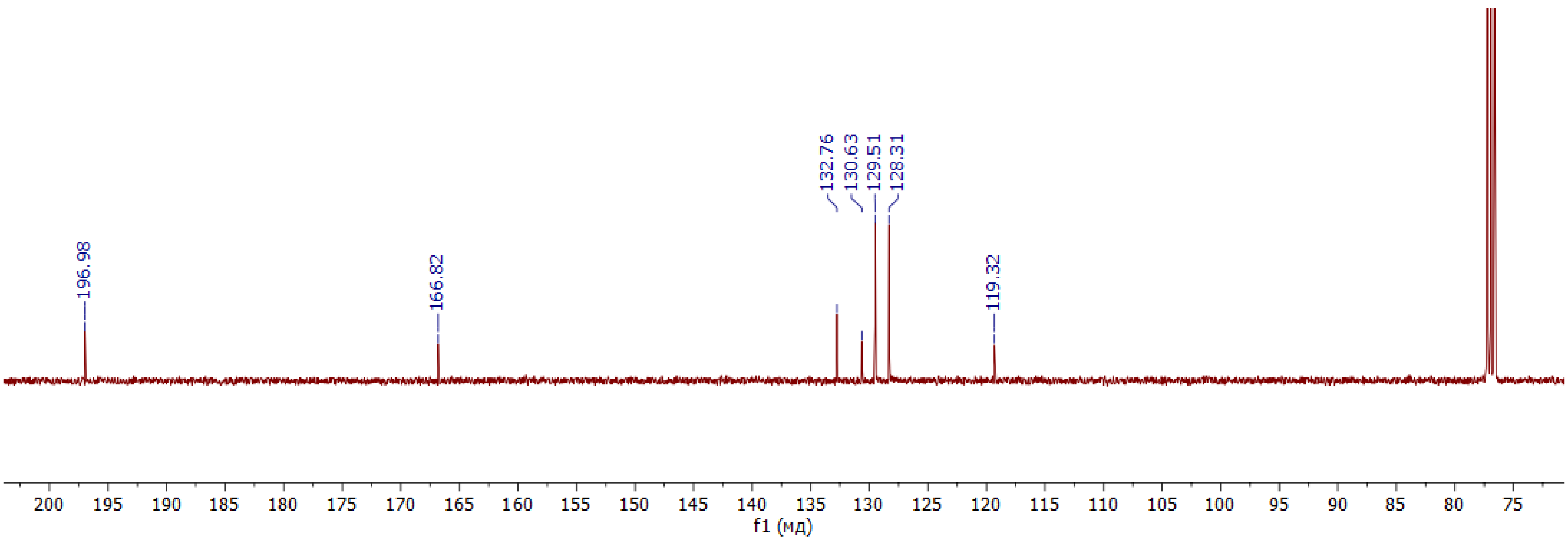
196.98
166.82
132.76
130.63
129.51
128.31
119.32
200 195 190 185 180 175 170 165 160 155 150 145 140 135 130 125 120 115 110 105 100 95 90 85 80 75
f1 (мд)

^{1}H NMR spectra of 24-Bromo-2-cyano-4-oxo-3,4-seco-3,23-dinorlupan-28-oic acid methyl ester (30)

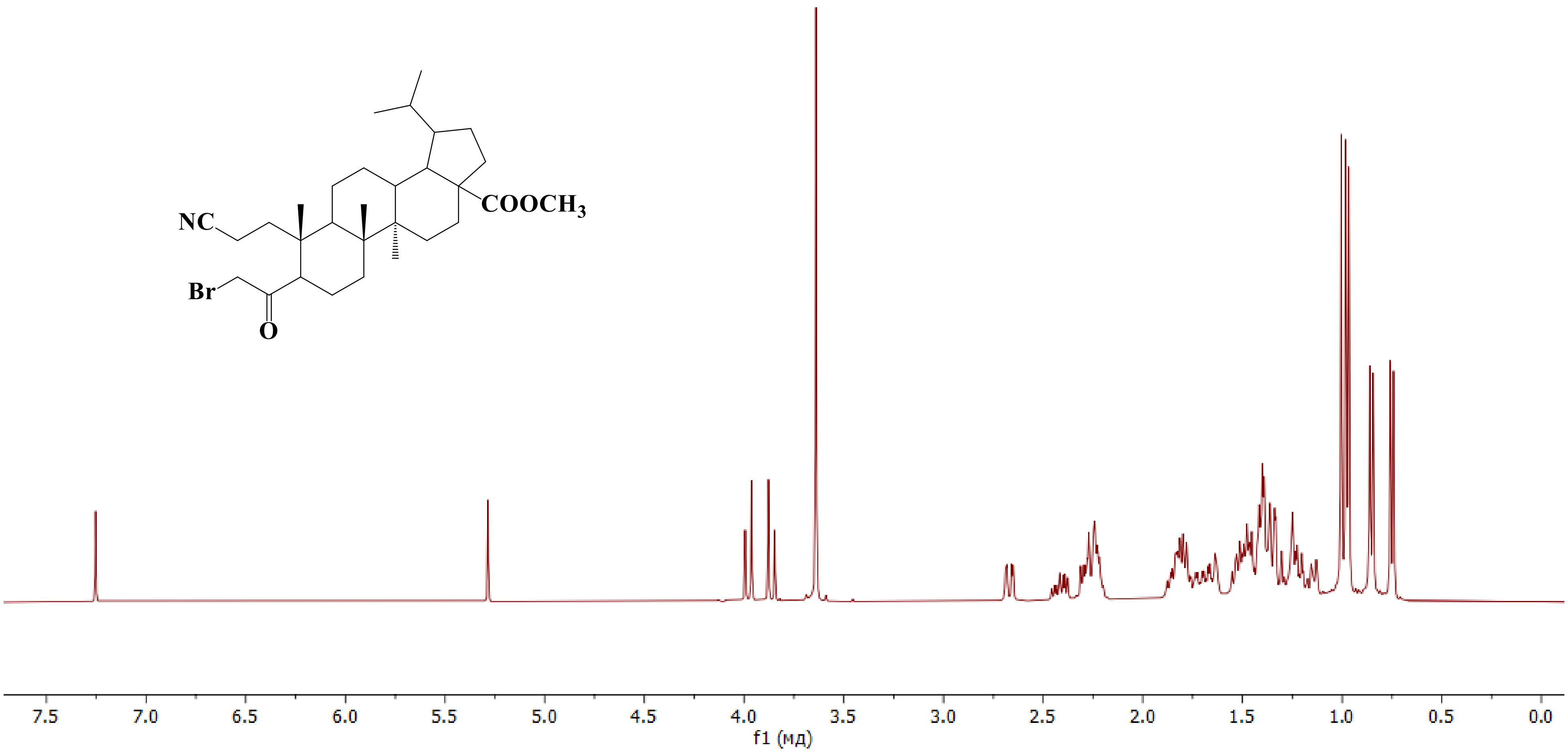

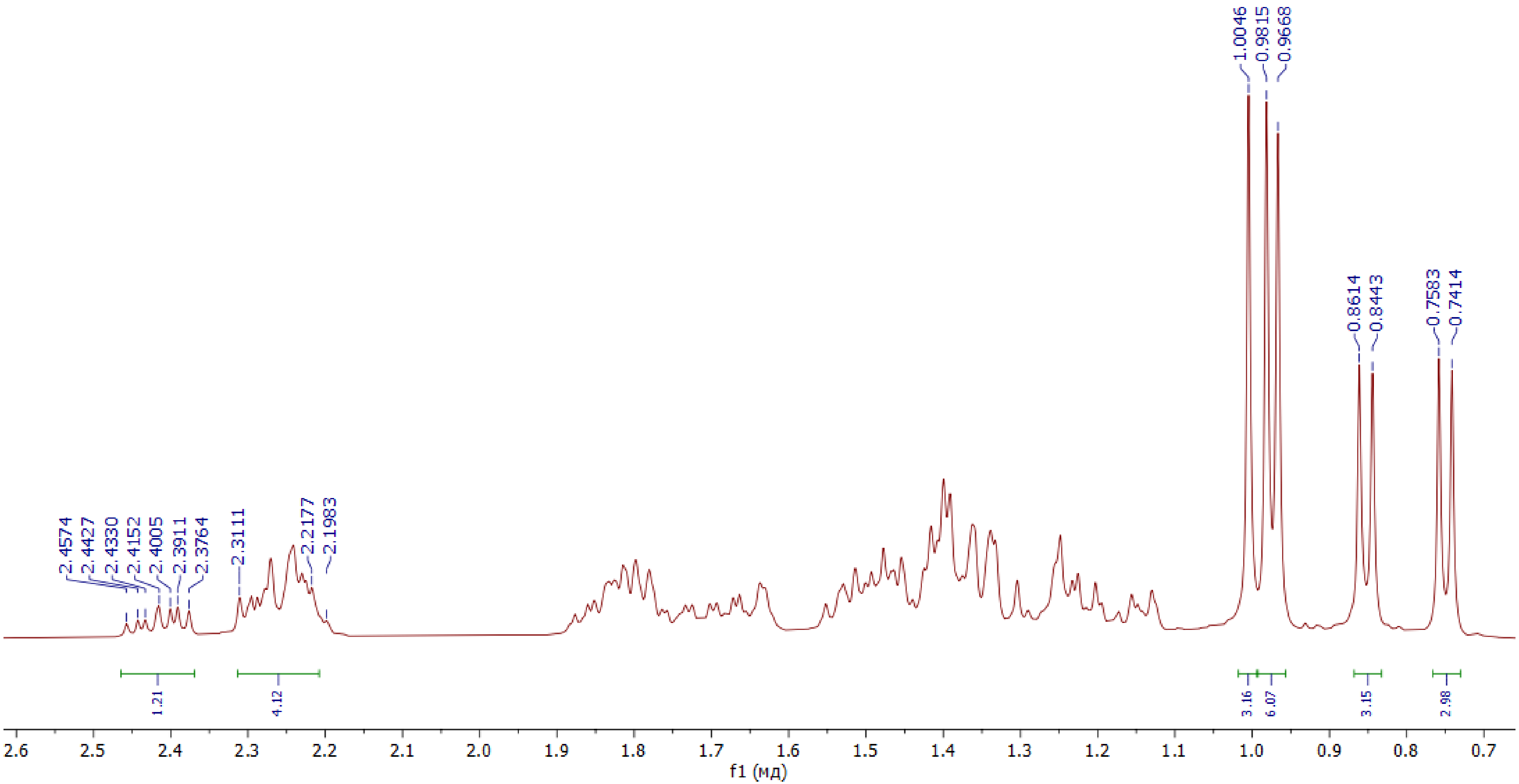

2.4574
2.4427
2.4330
2.4152
2.4005
2.3911
2.3764
2.3111
2.2177
2.1983
1.0046
0.9815
0.9668
0.8614
0.8443
0.7583
0.7414
1.21
4.12
3.16
6.07
3.15
2.98
2.6
2.5
2.4
2.3
2.2
2.1
2.0
1.9
1.8
1.7
1.6
1.5
1.4
1.3
1.2
1.1
1.0
0.9
0.8
0.7
f1 (мд)

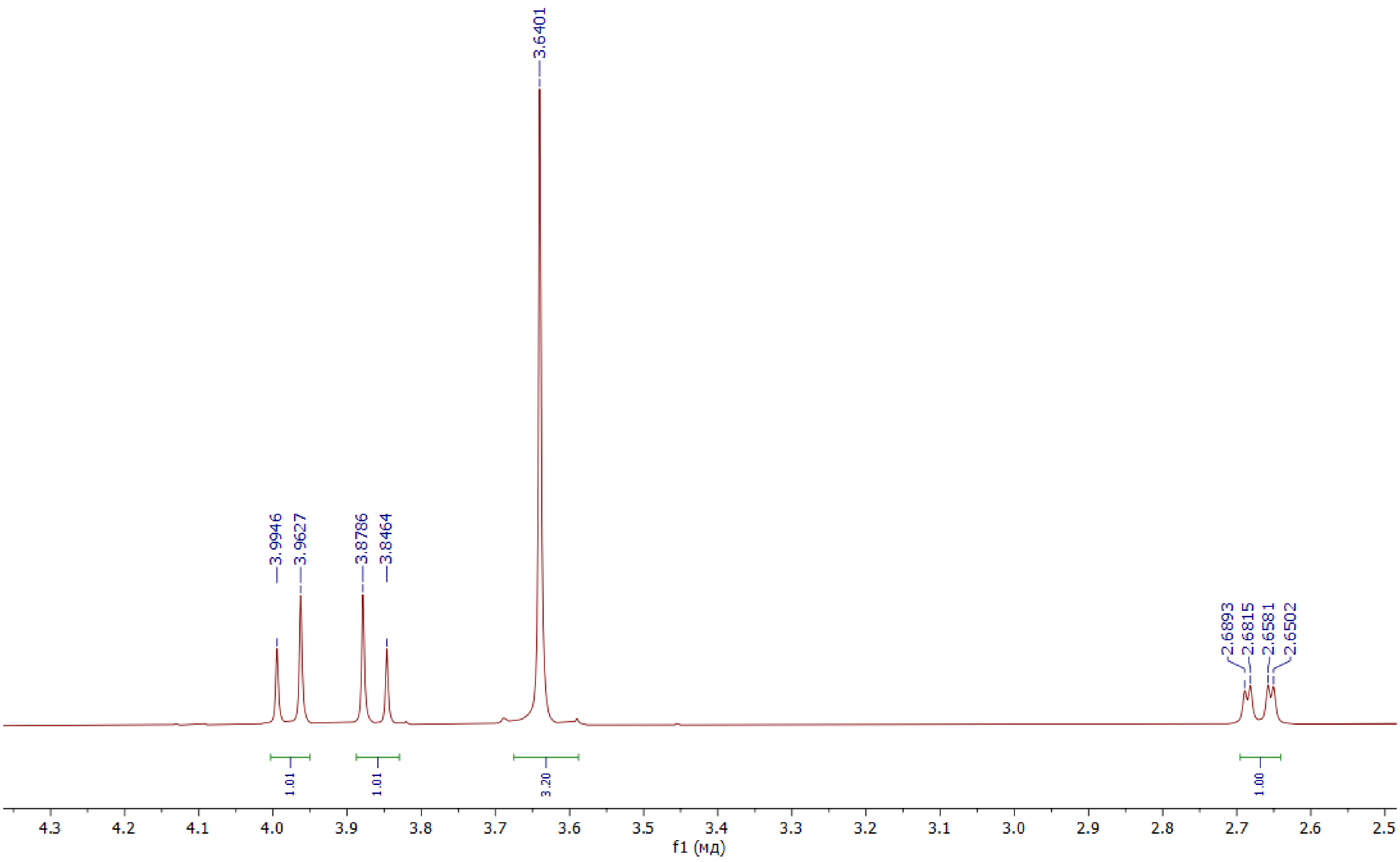

3.9946
3.9627
3.8786
3.8464
3.6401
2.6893
2.6815
2.6581
2.6502
1.01
1.01
3.20
1.00
4.3 4.2 4.1 4.0 3.9 3.8 3.7 3.6 3.5 3.4 3.3 3.2 3.1 3.0 2.9 2.8 2.7 2.6 2.5
f1 (мд)

^{13}C NMR spectra of 24-Bromo-2-cyano-4-oxo-3,4-seco-3,23-dinorlupan-28-oic acid methyl ester (30)

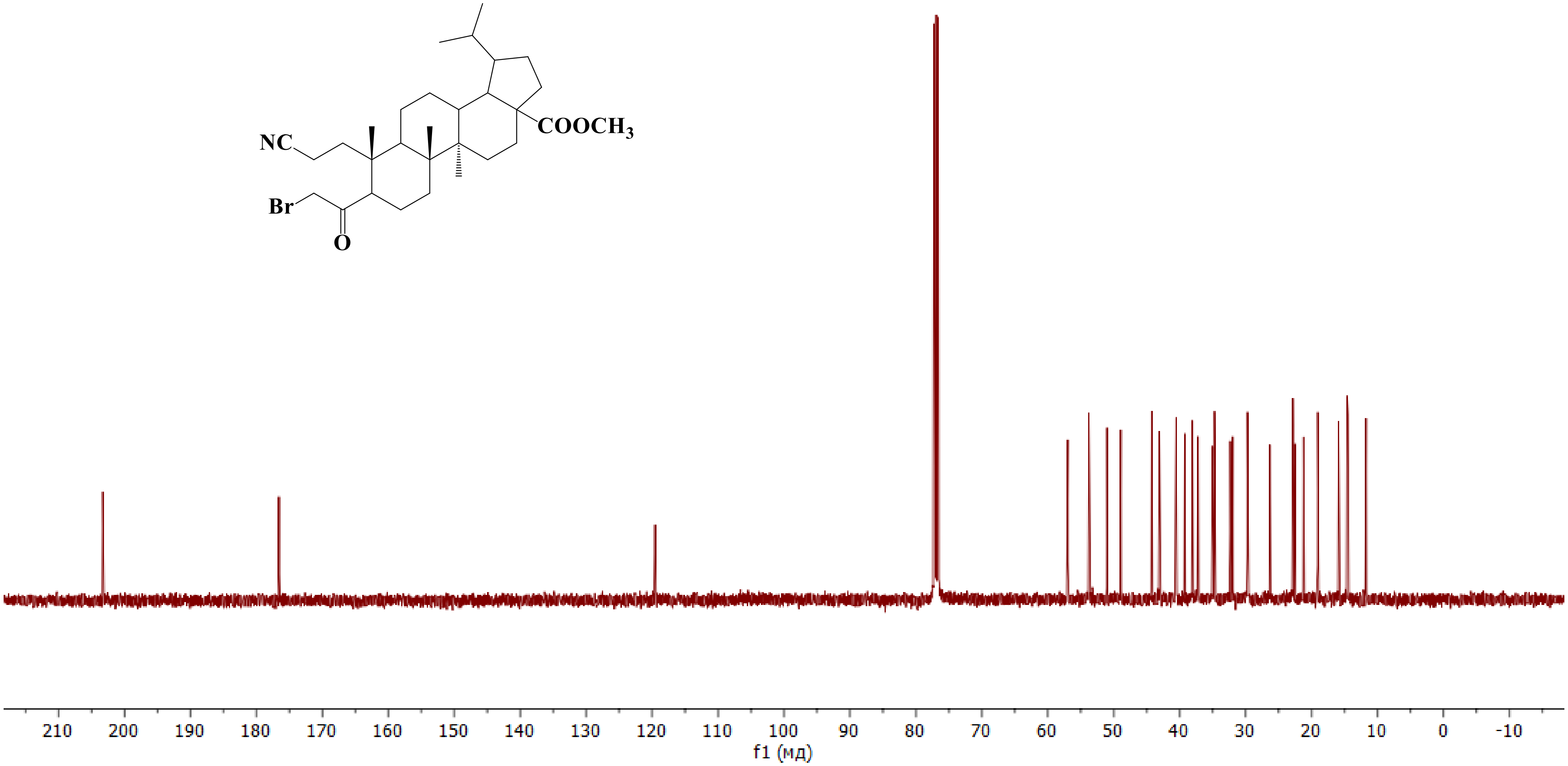

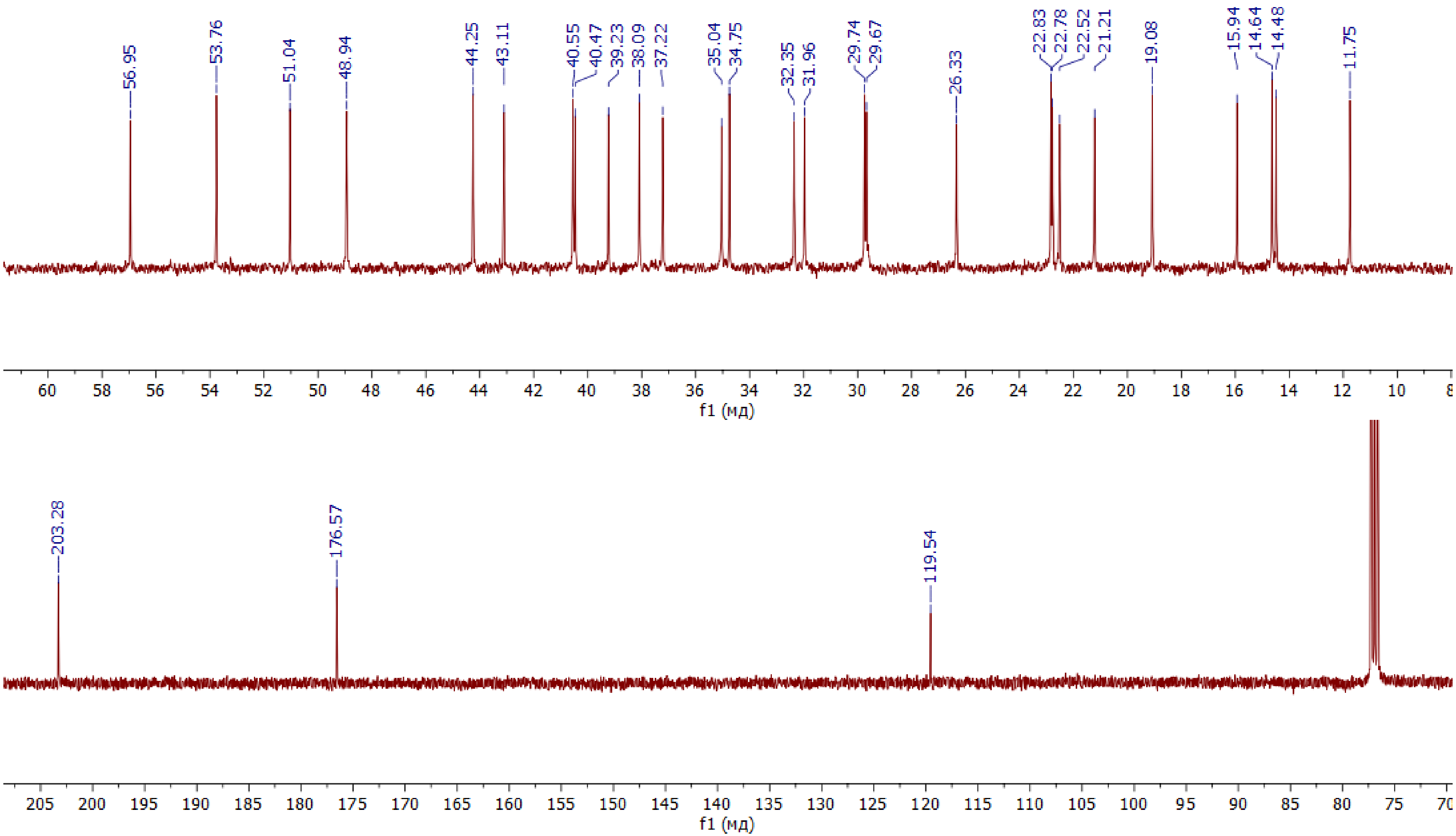
56.95
53.76
51.04
48.94
44.25
43.11
40.55
40.47
39.23
38.09
37.22
35.04
34.75
32.35
31.96
29.74
29.67
26.33
22.83
22.78
22.52
21.21
19.08
15.94
14.64
14.48
11.75
f1 (мд)
203.28
176.57
119.54
f1 (мд)

^1H NMR spectra of 2-Cyano-24-dibromo-4-oxo-3,4-seco-3,23-dinorlupan-28-oic acid methyl ester (31).

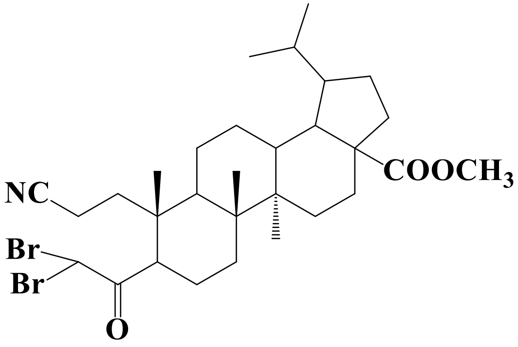

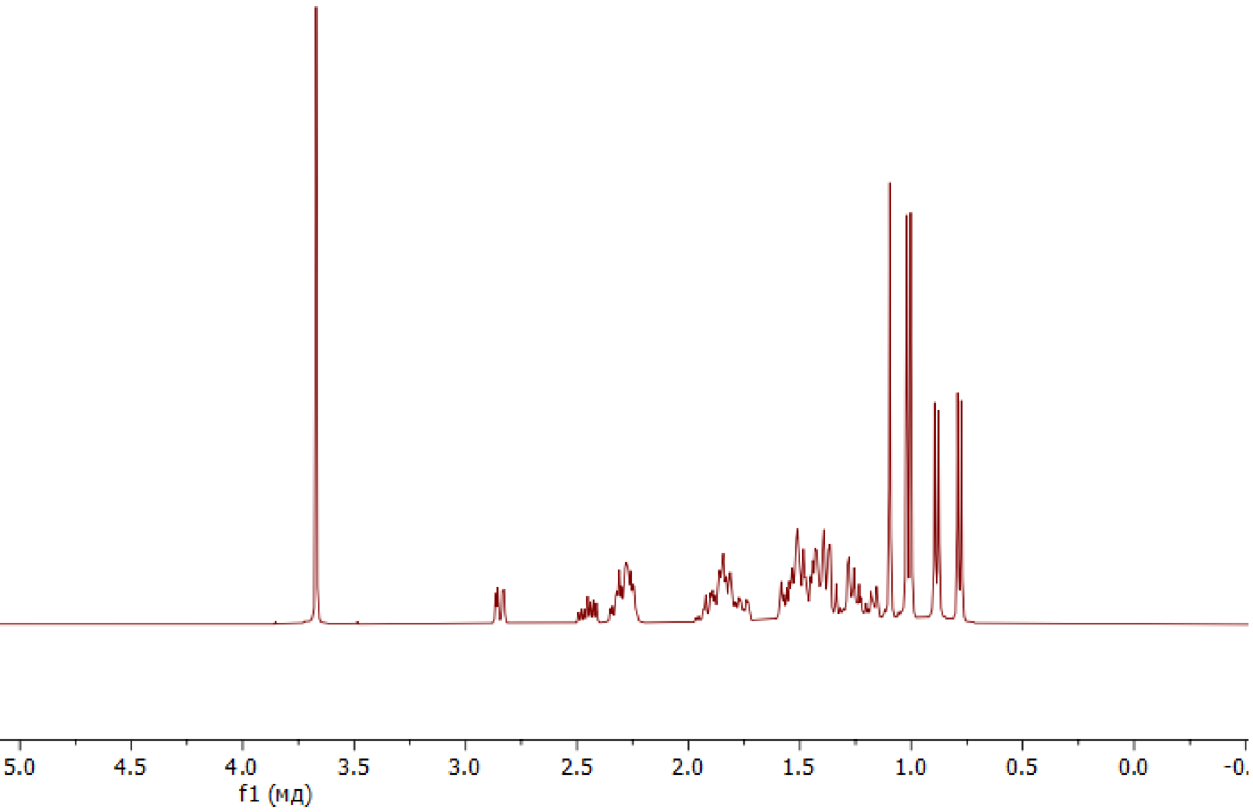

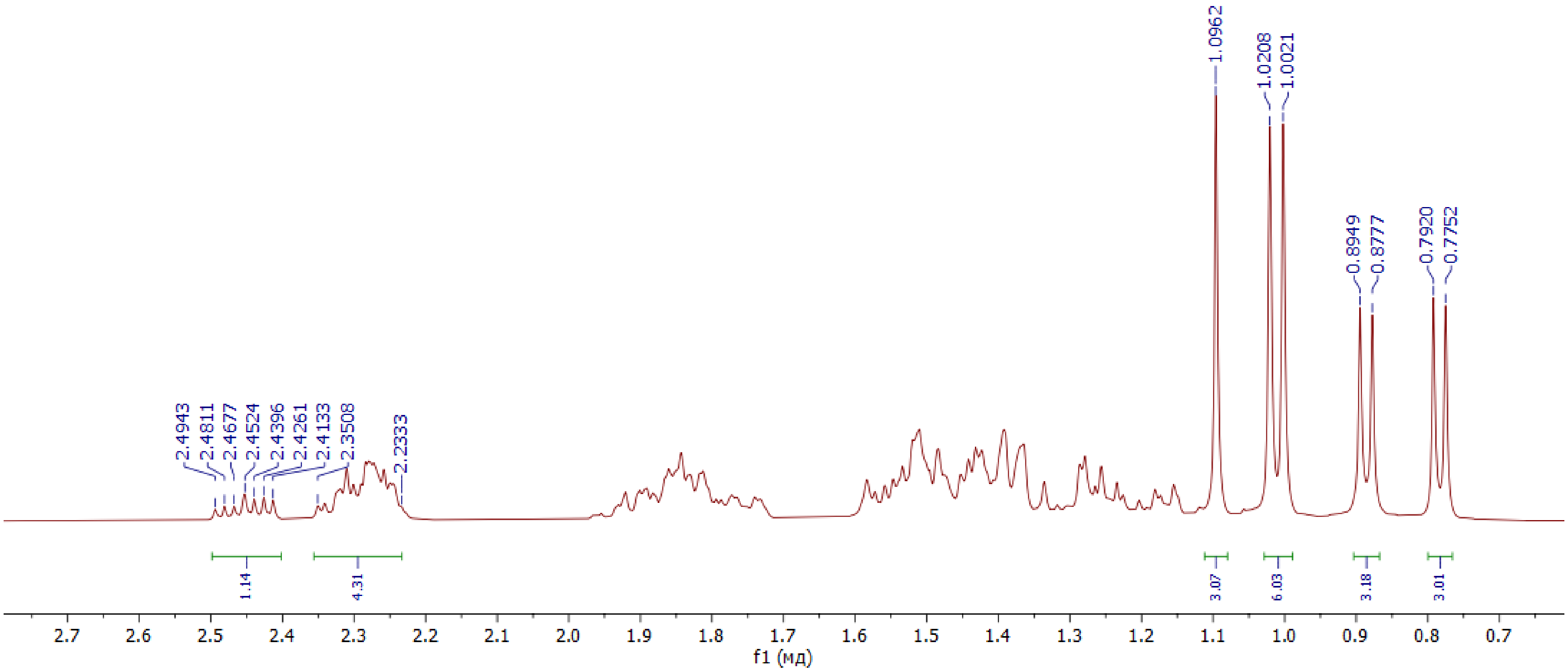
2.4943
2.4811
2.4677
2.4524
2.4396
2.4261
2.4133
2.3508
2.2333
1.0962
1.0208
1.0021
0.8949
0.8777
0.7920
0.7752
1.14
4.31
3.07
6.03
3.18
3.01
2.7
2.6
2.5
2.4
2.3
2.2
2.1
2.0
1.9
1.8
1.7
1.6
1.5
1.4
1.3
1.2
1.1
1.0
0.9
0.8
0.7
f1 (мд)

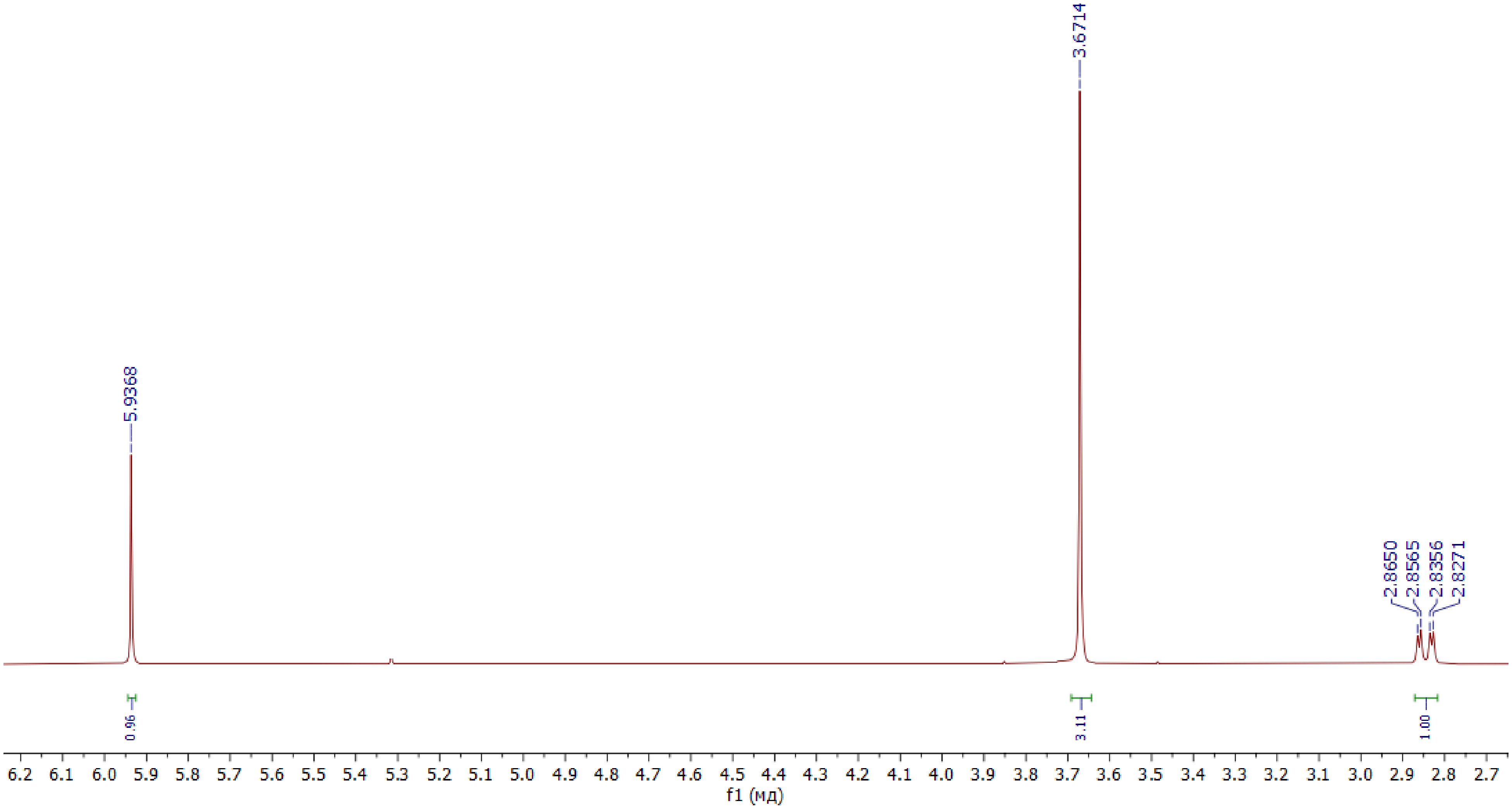
5.9368
3.6714
2.8650
2.8565
2.8356
2.8271
0.96
3.11
1.00
6.2 6.1 6.0 5.9 5.8 5.7 5.6 5.5 5.4 5.3 5.2 5.1 5.0 4.9 4.8 4.7 4.6 4.5 4.4 4.3 4.2 4.1 4.0 3.9 3.8 3.7 3.6 3.5 3.4 3.3 3.2 3.1 3.0 2.9 2.8 2.7
f1 (мд)

^{13}C NMR spectra of 2-Cyano-24-dibromo-4-oxo-3,4-seco-3,23-dinorlupan-28-oic acid methyl ester (31).

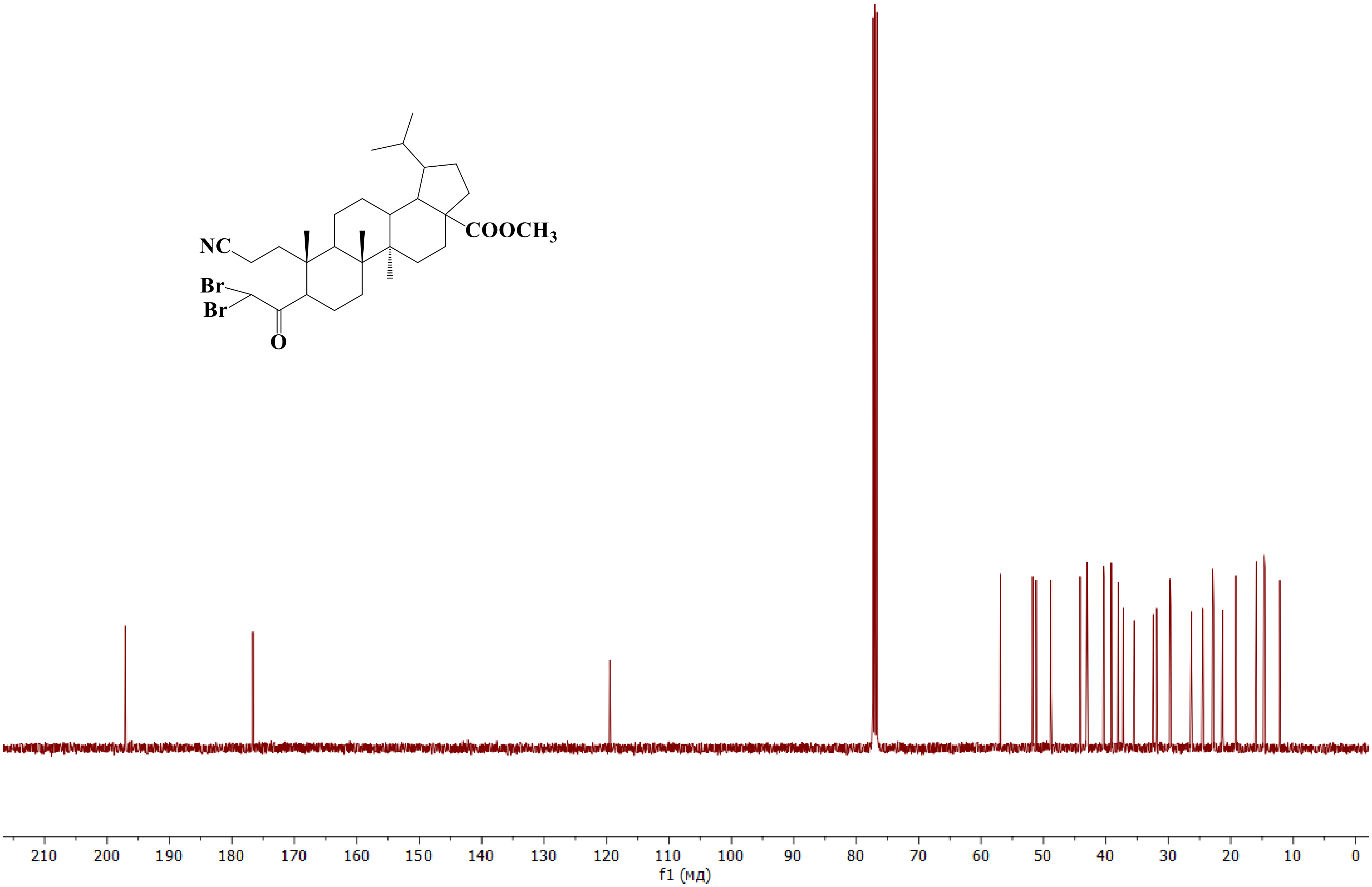

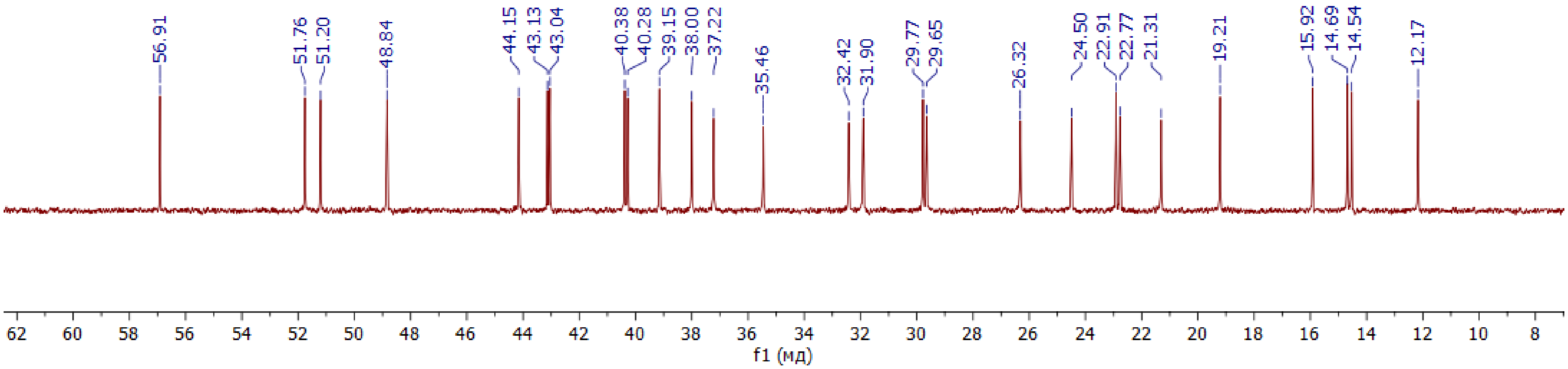
56.91
51.76
51.20
48.84
44.15
43.13
43.04
40.38
40.28
39.15
38.00
37.22
35.46
32.42
31.90
29.77
29.65
26.32
24.50
22.91
22.77
21.31
19.21
15.92
14.69
14.54
12.17
62 60 58 56 54 52 50 48 46 44 42 40 38 36 34 32 30 28 26 24 22 20 18 16 14 12 10 8
f1 (мд)

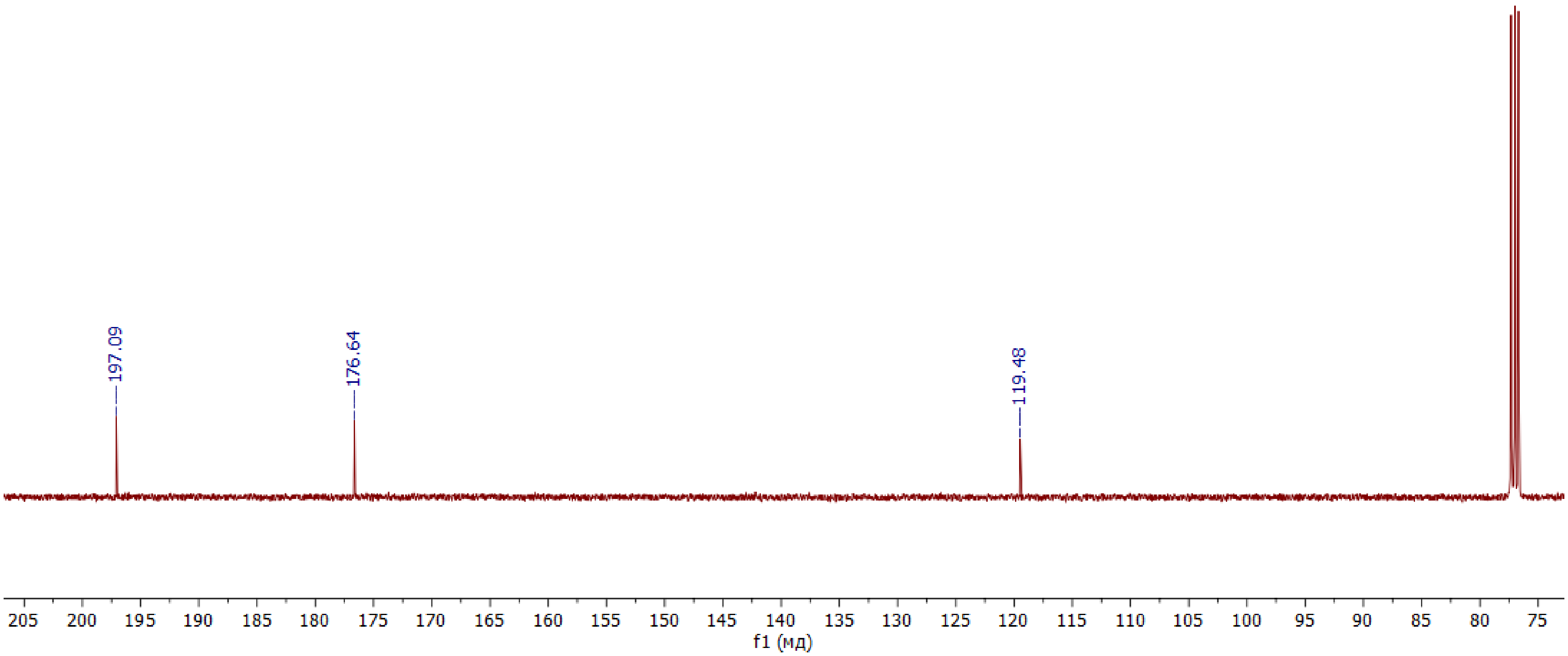
197.09
176.64
119.48
205 200 195 190 185 180 175 170 165 160 155 150 145 140 135 130 125 120 115 110 105 100 95 90 85 80 75
f1 (мд)

^{1}H NMR spectra of 2-Cyano-4-oxo-24-tribromo-3,23-dinorlupan-28-oic acid methyl ester (32).

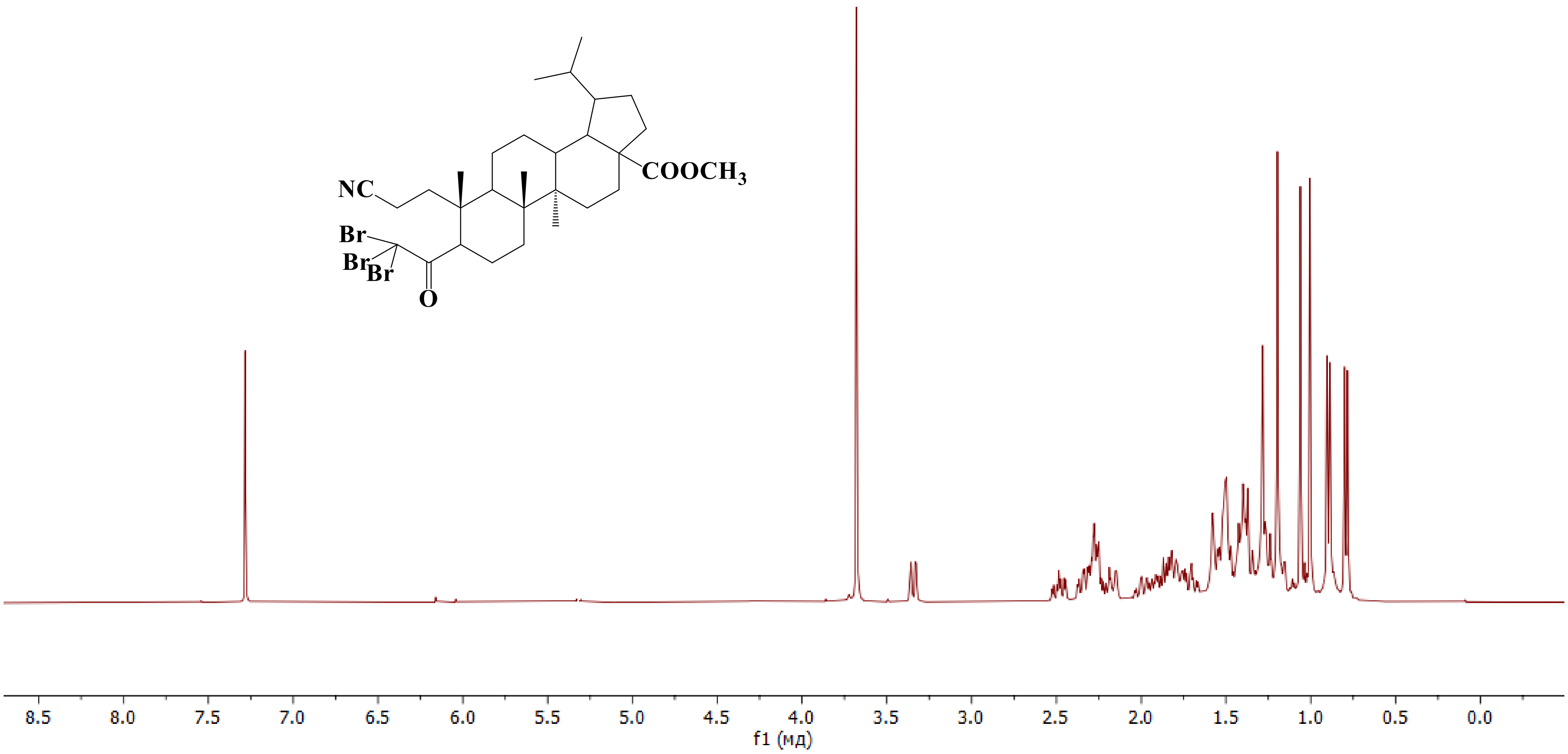

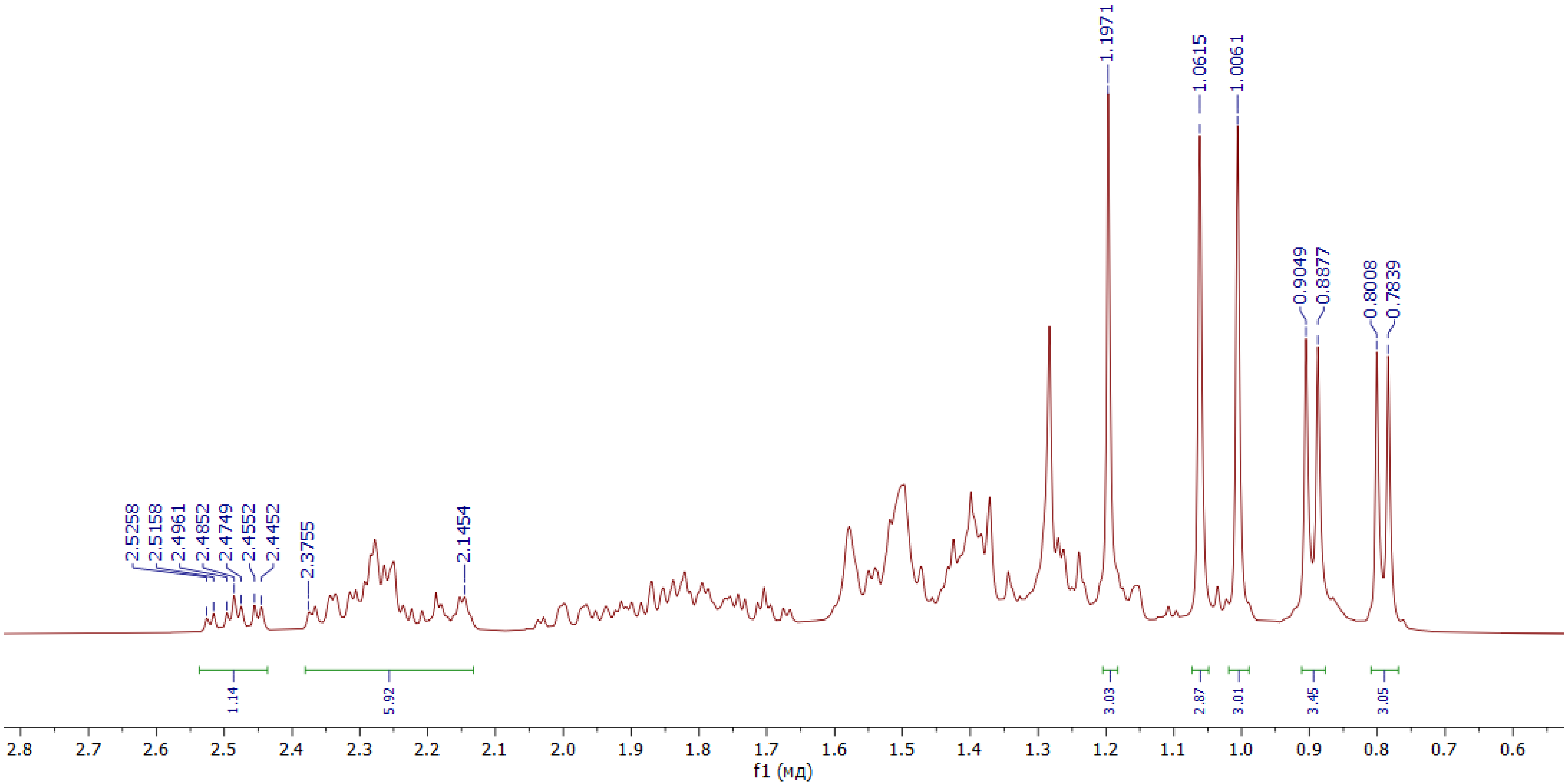
2.5258
2.5158
2.4961
2.4852
2.4749
2.4552
2.4452
2.3755
2.1454
1.1971
1.0615
1.0061
0.9049
0.8877
0.8008
0.7839
1.14
5.92
3.03
2.87
3.01
3.45
3.05
2.8 2.7 2.6 2.5 2.4 2.3 2.2 2.1 2.0 1.9 1.8 1.7 1.6 1.5 1.4 1.3 1.2 1.1 1.0 0.9 0.8 0.7 0.6
f1 (мд)

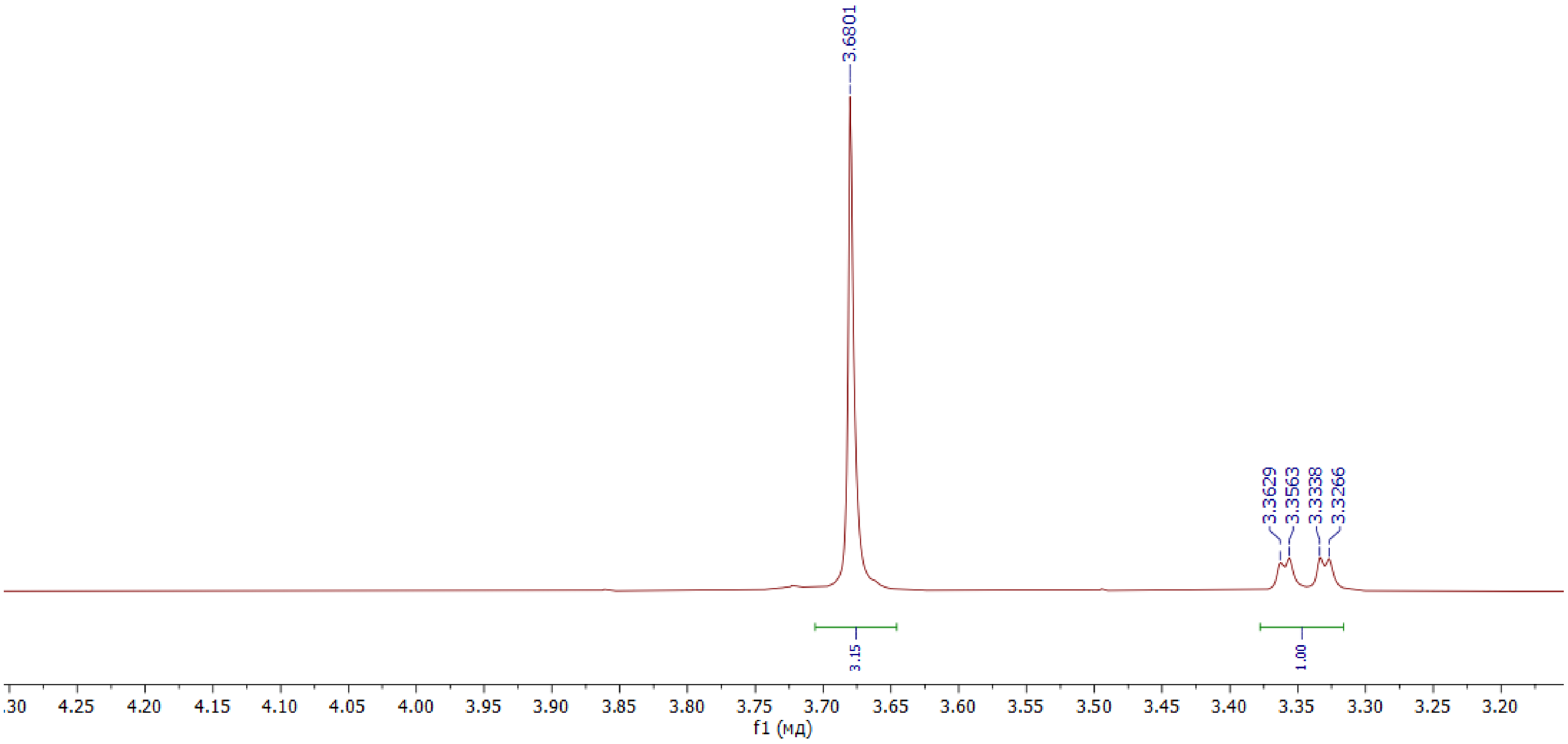

3.6801
3.3629
3.3563
3.3338
3.3266
3.15
1.00
.30
4.25
4.20
4.15
4.10
4.05
4.00
3.95
3.90
3.85
3.80
3.75
3.70
3.65
3.60
3.55
3.50
3.45
3.40
3.35
3.30
3.25
3.20
f1 (мд)

^{13}C NMR spectra of 2-Cyano-4-oxo-24-tribromo-3,23-dinorlupan-28-oic acid methyl ester (32).

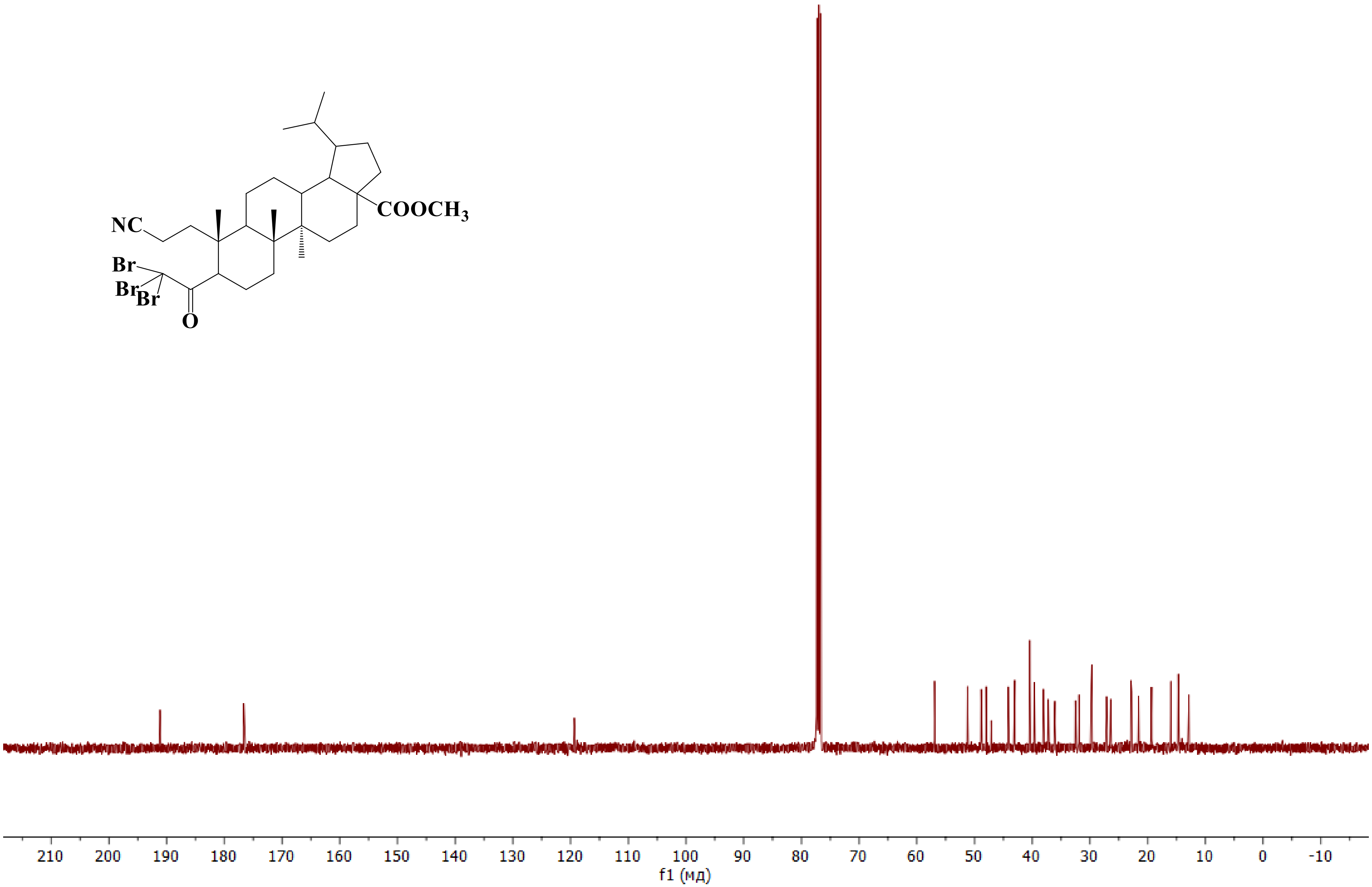

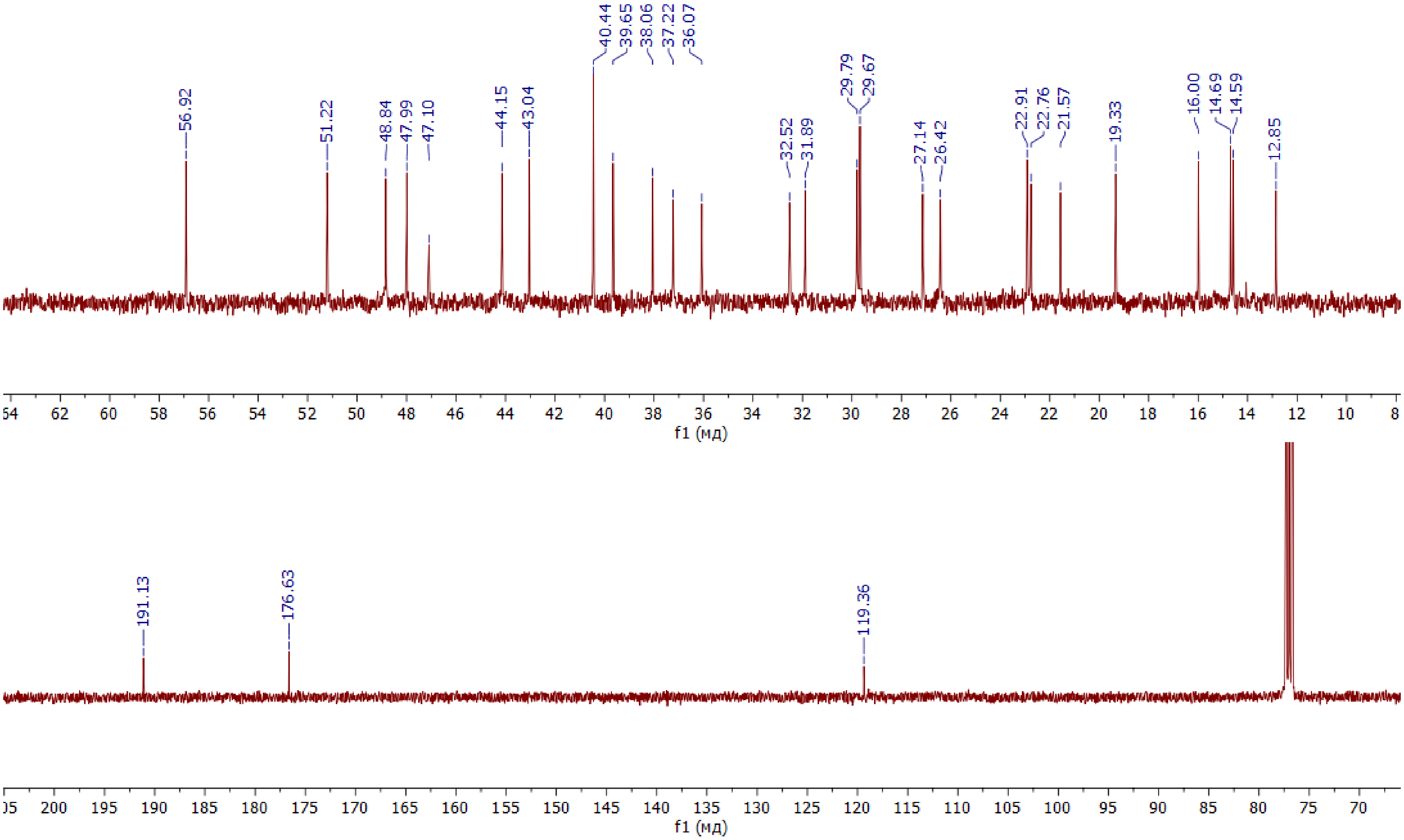

56.92
51.22
48.84
47.99
47.10
44.15
43.04
40.44
39.65
38.06
37.22
36.07
32.52
31.89
29.79
29.67
27.14
26.42
22.91
22.76
21.57
19.33
16.00
14.69
14.59
12.85
f1 (мд)
191.13
176.63
119.36
f1 (мд)

Mass spectra (MS).

28-Benzoyloxy-2-cyano-3,4-seco-3-norlup-4(23)-en-24-al (4).

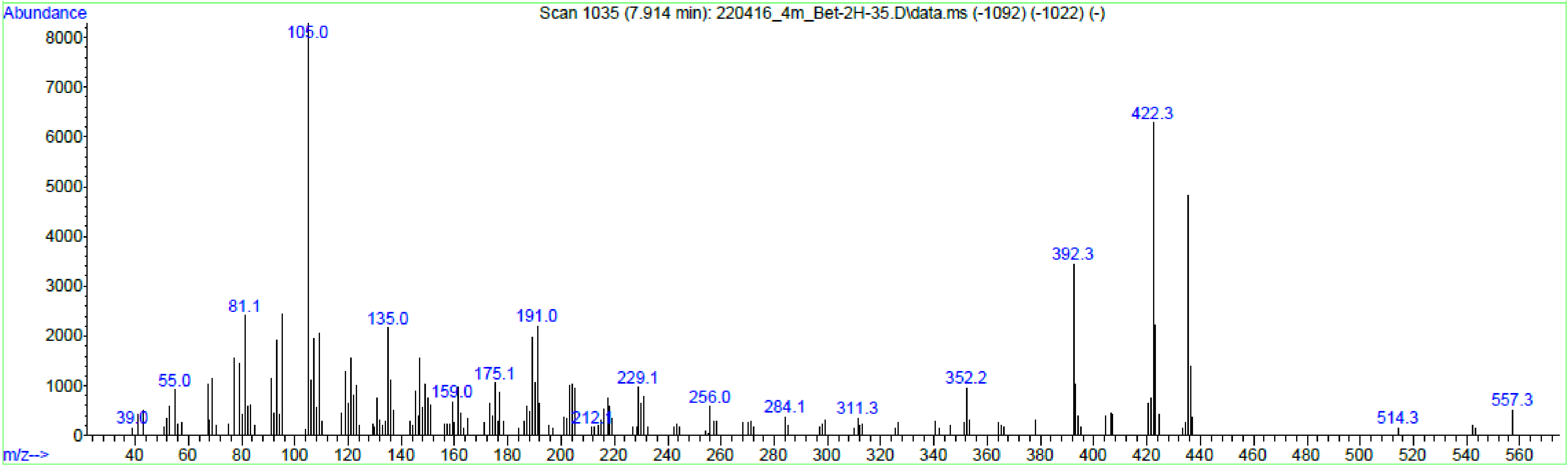

2-Cyano-3,4-seco-3-norlup-4(23)-en-24-al-28-oic acid methyl ester (5).

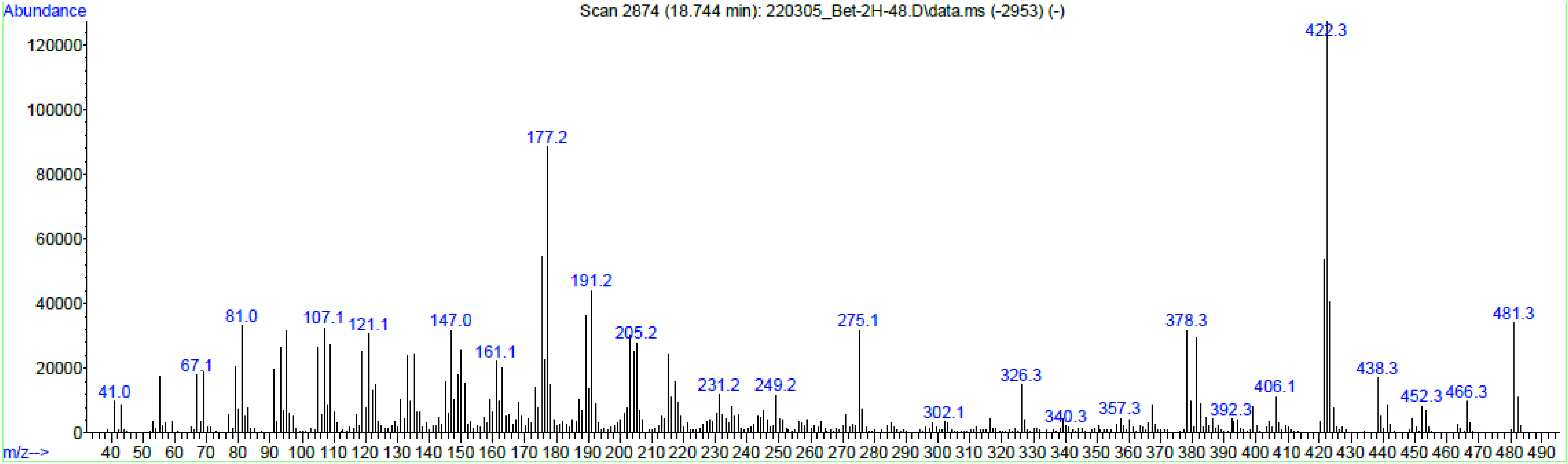

28-Benzoxy-24-bromo-2-cyano-3,4-seco-3-norlup-4(23)-ene (6).

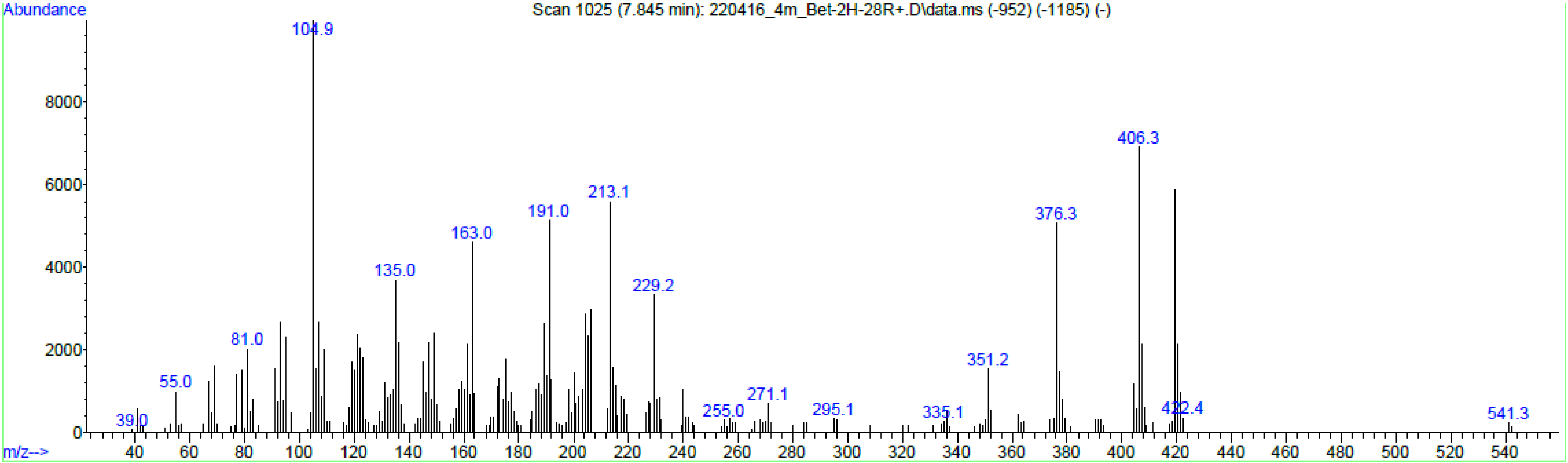

24-Bromo-2-cyano-3-norlup-4(23)-en-28-oic acid methyl ester (7).

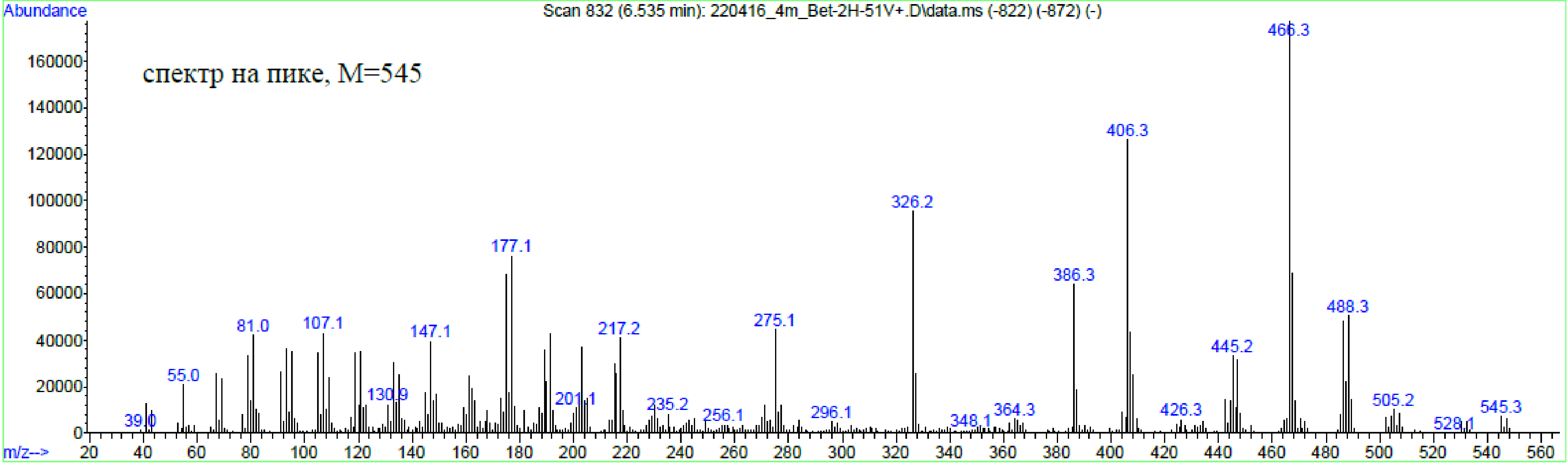

2-Cyano-28-hydroxy-3-norlup-2(24),4(23)-diene (8).

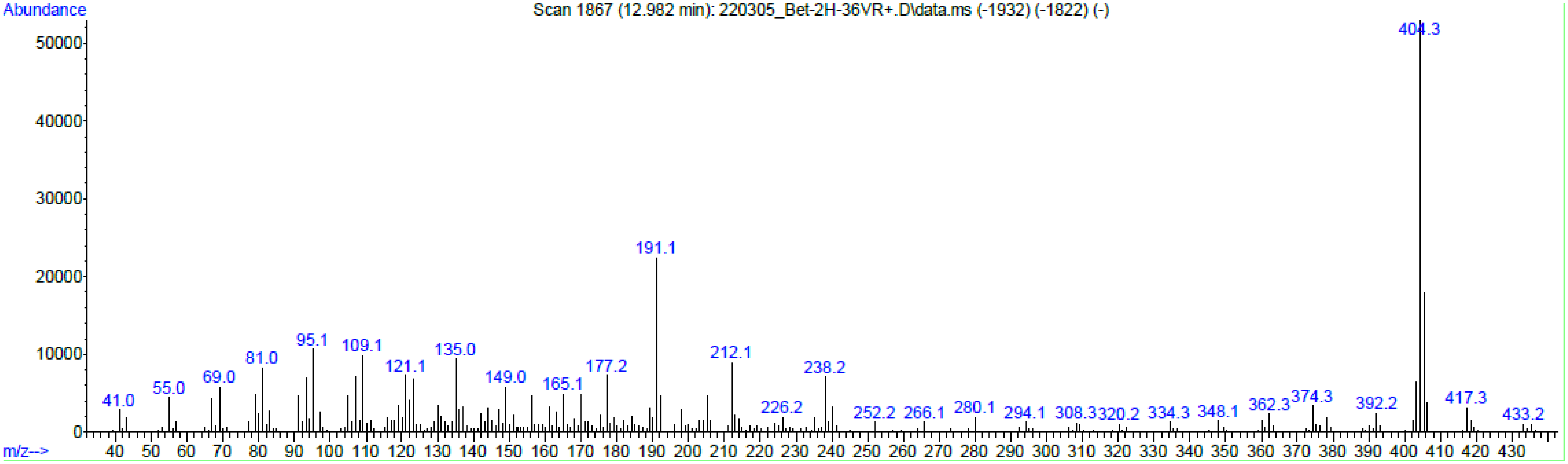

2-Cyano-24β,28-dihydroxy-3-norlup-4(23)-ene (9).

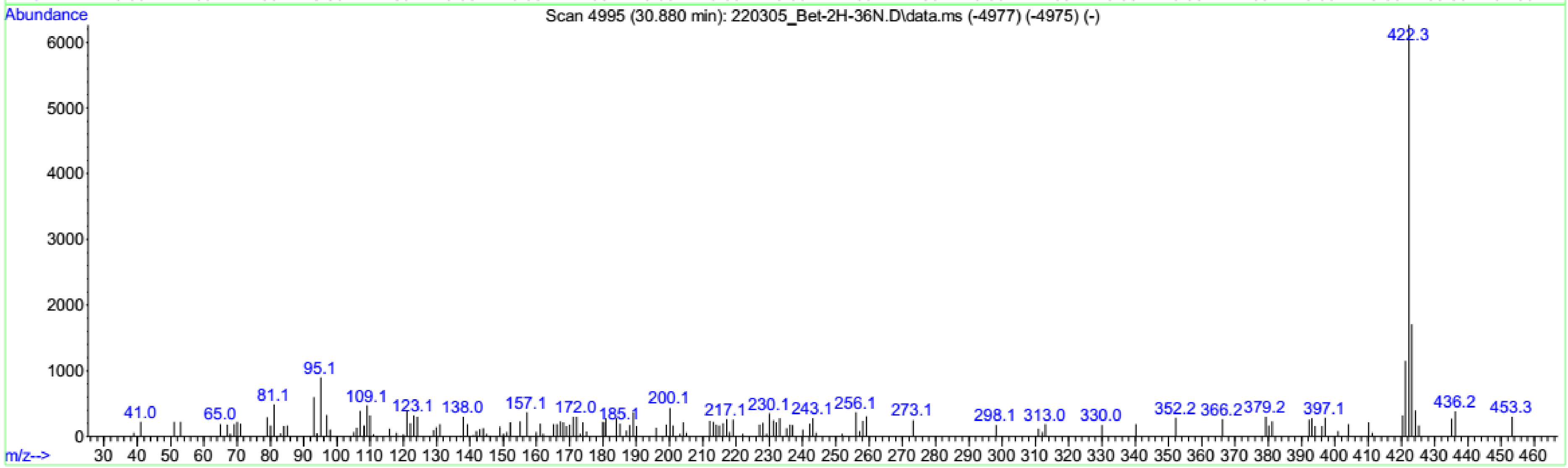

2-Cyano-3-norlup-2(24),4(23)-diene-28-oic acid methyl ester (10)

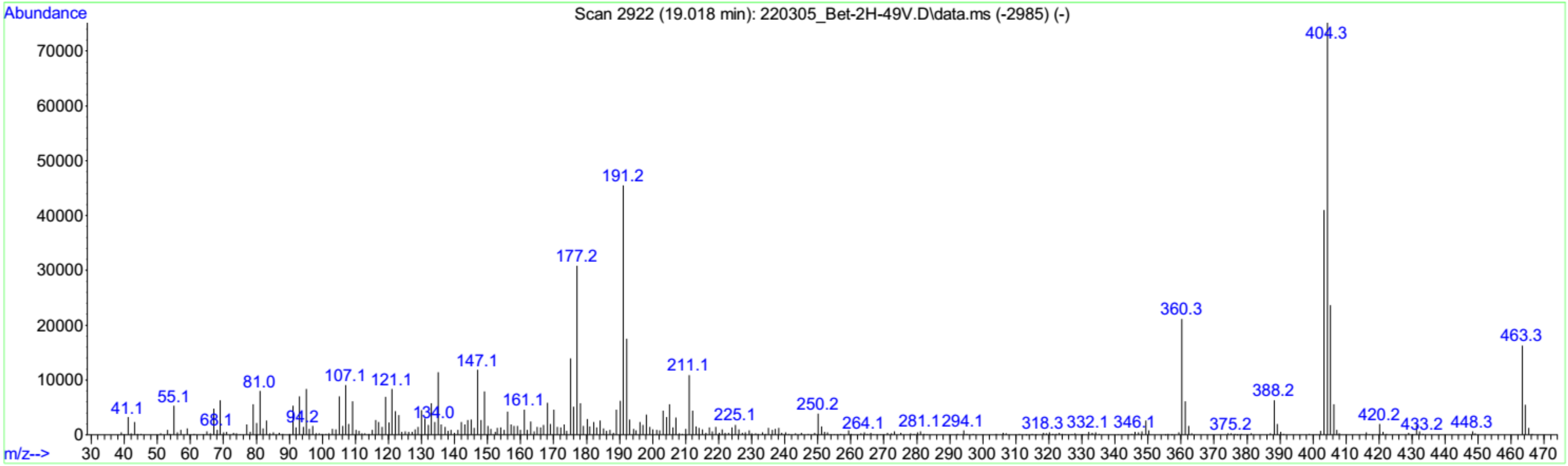

2-Cyano-24β-hydroxy-3-norlup-4(23)-en-28-oic acid methyl ester (11).

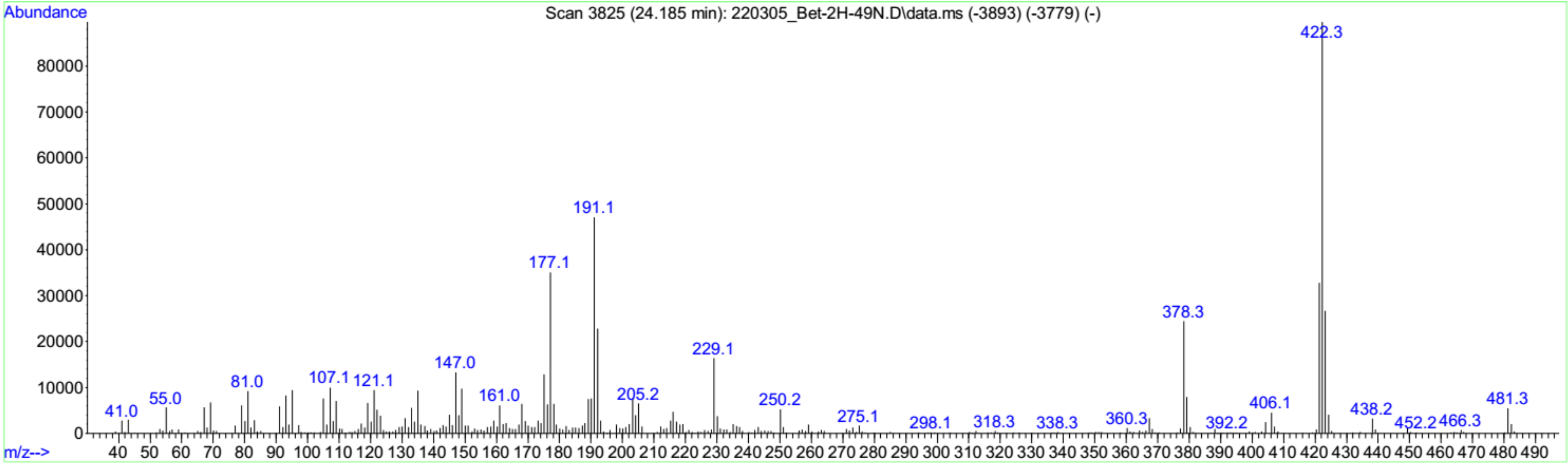

2-Cyano-28-hydroxy-3,23-dinorlup-2(4)-en (12).

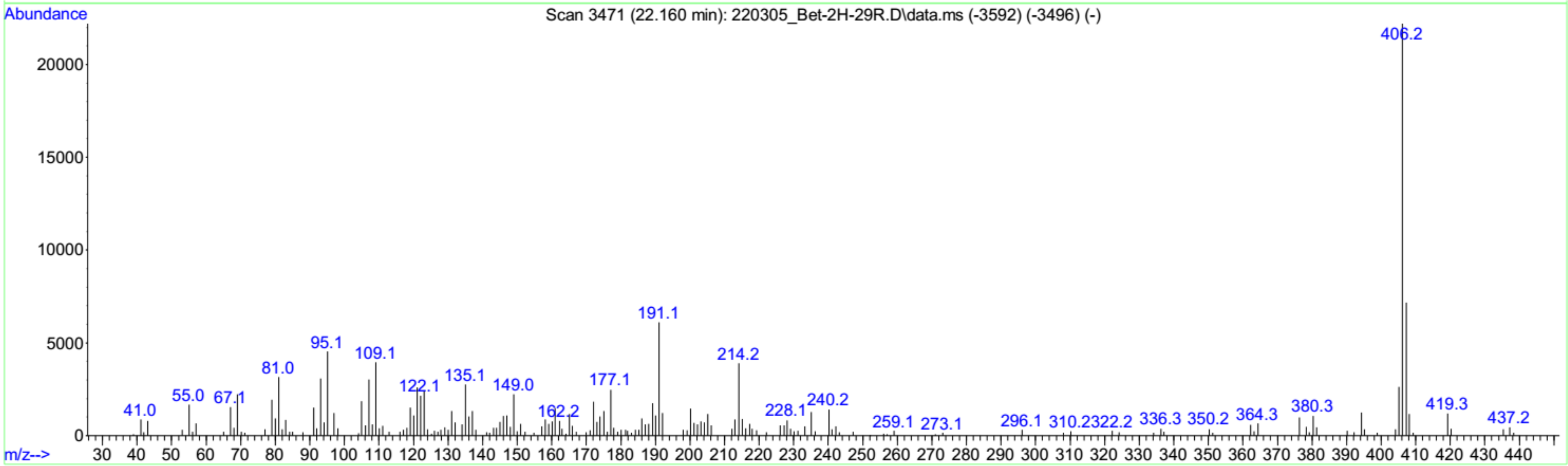

2-Cyano-3,23-dinorlup-2(4)-en-28-oic acid methyl ester (13).

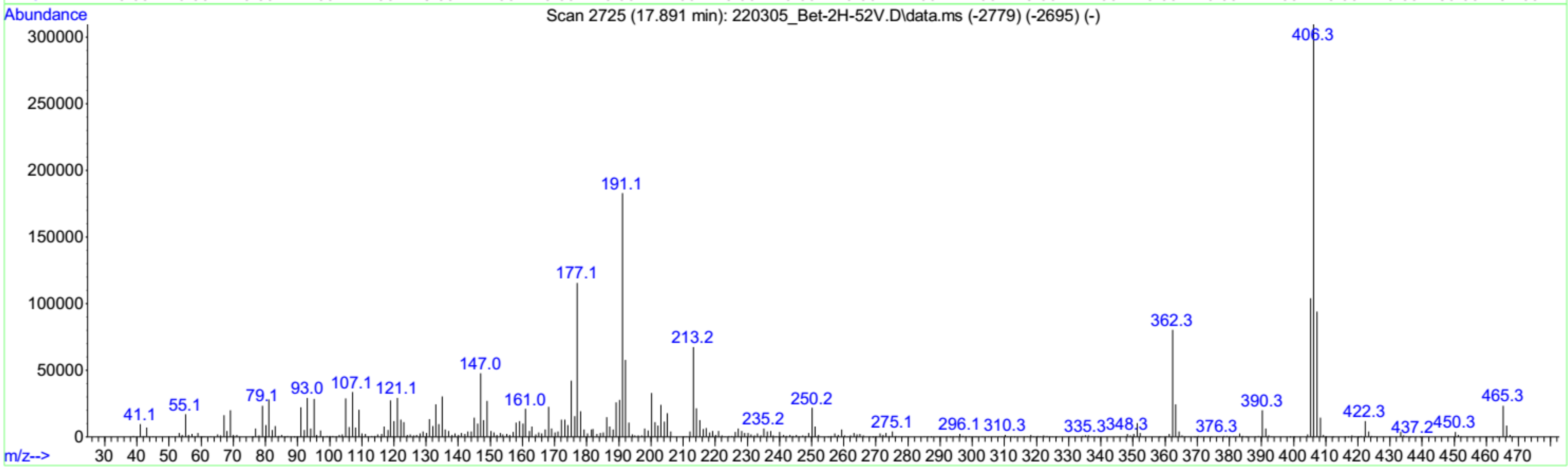

2-Cyano-28-hydroxy-4-oxo-3,23-dinorlup-2(24)-ene (14).

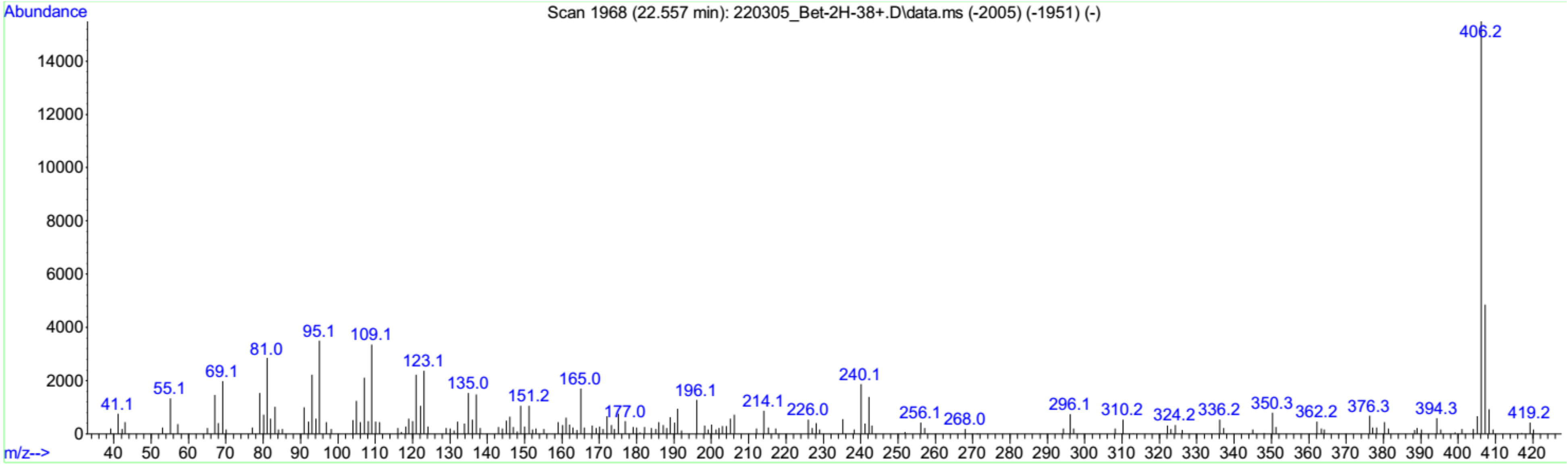

2-Cyano-4-oxo-3,23-dinorlup-2(24)-en-28-oic acid methyl ester (15).

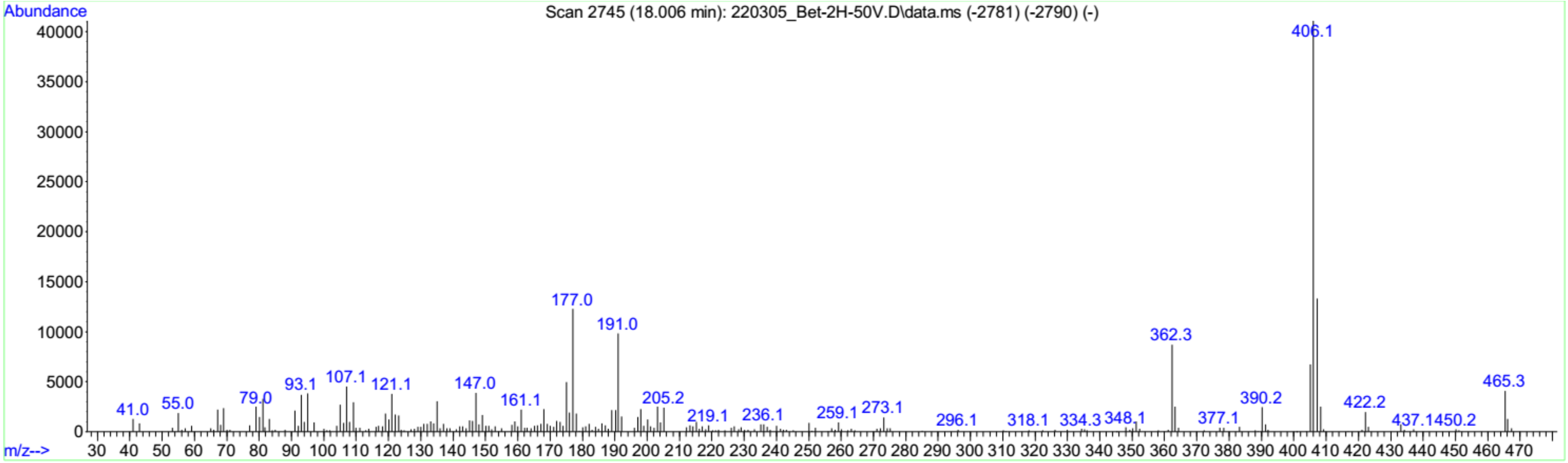

2-Cyano-28-hydroxy-4-oxo-3,23-dinorlupan (16).

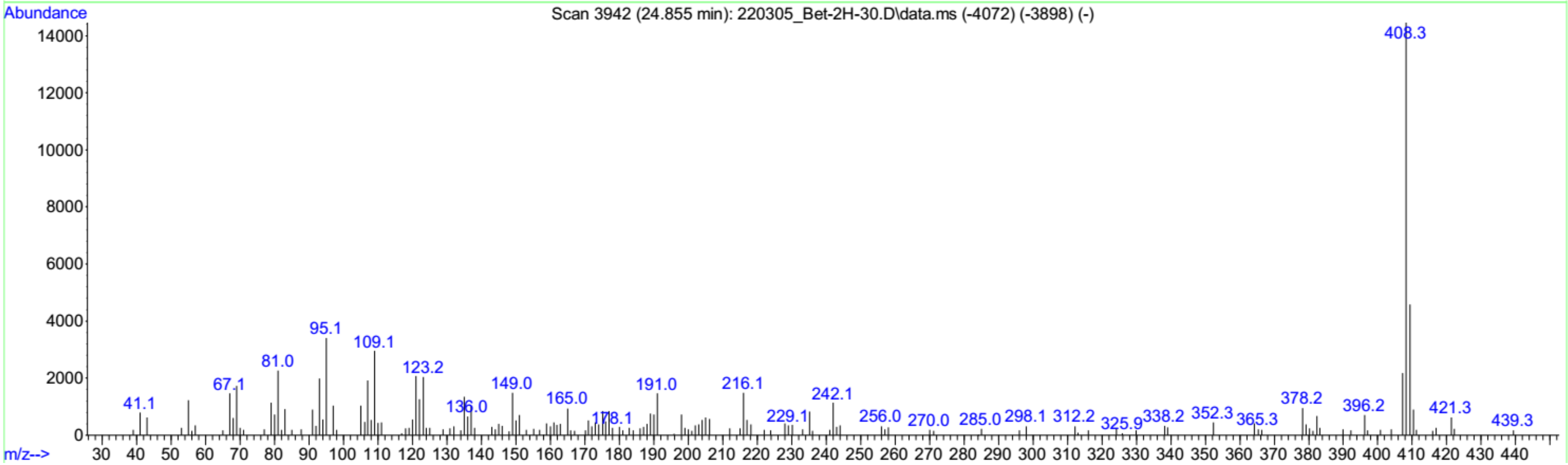

2-Cyano-4-oxo-3,23-dinorlupan-28-oic acid methyl ester (17).

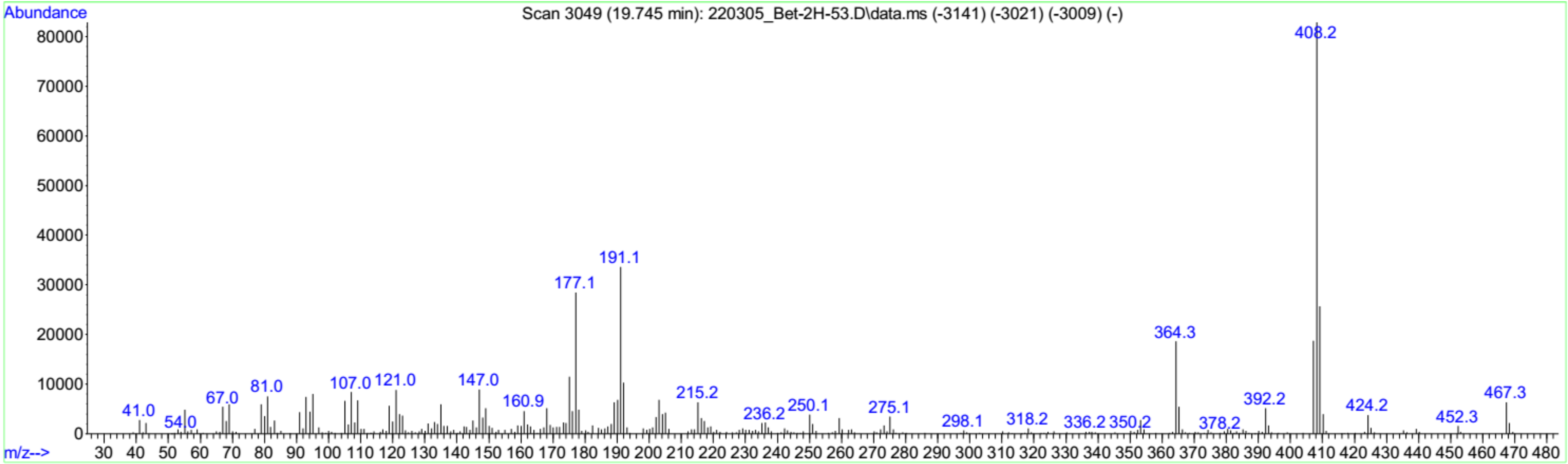

2-Cyano-4β,28-dihydroxy-3,23-dinorlupan (18).

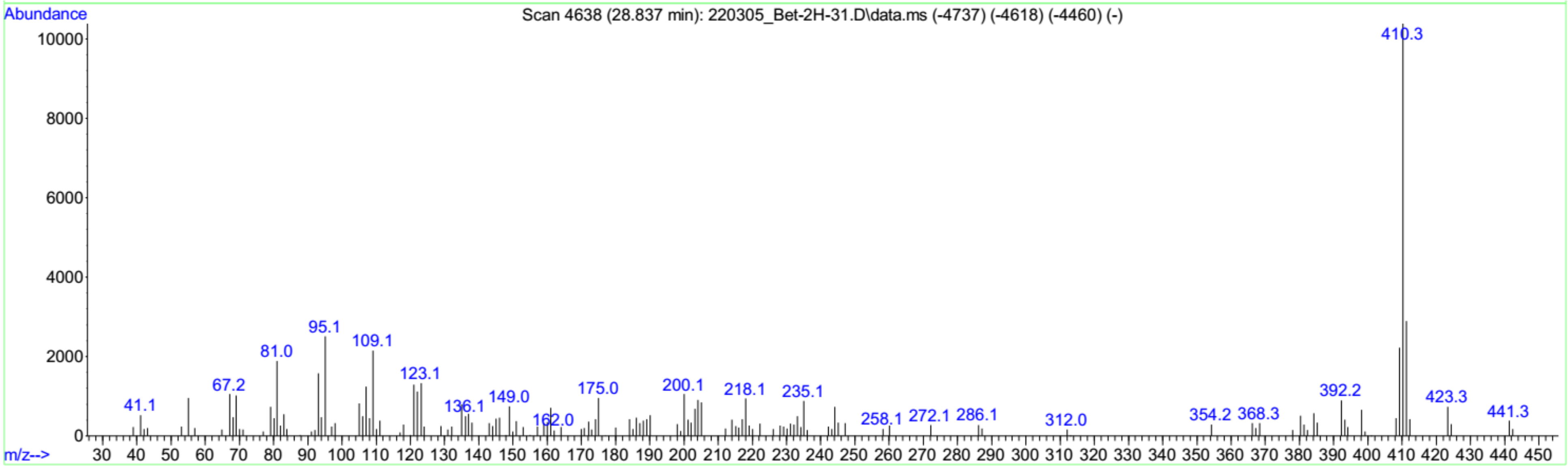

2-Cyano-4-hydroxy-3,23-dinorlupan-28-oic acid methyl ester (19).

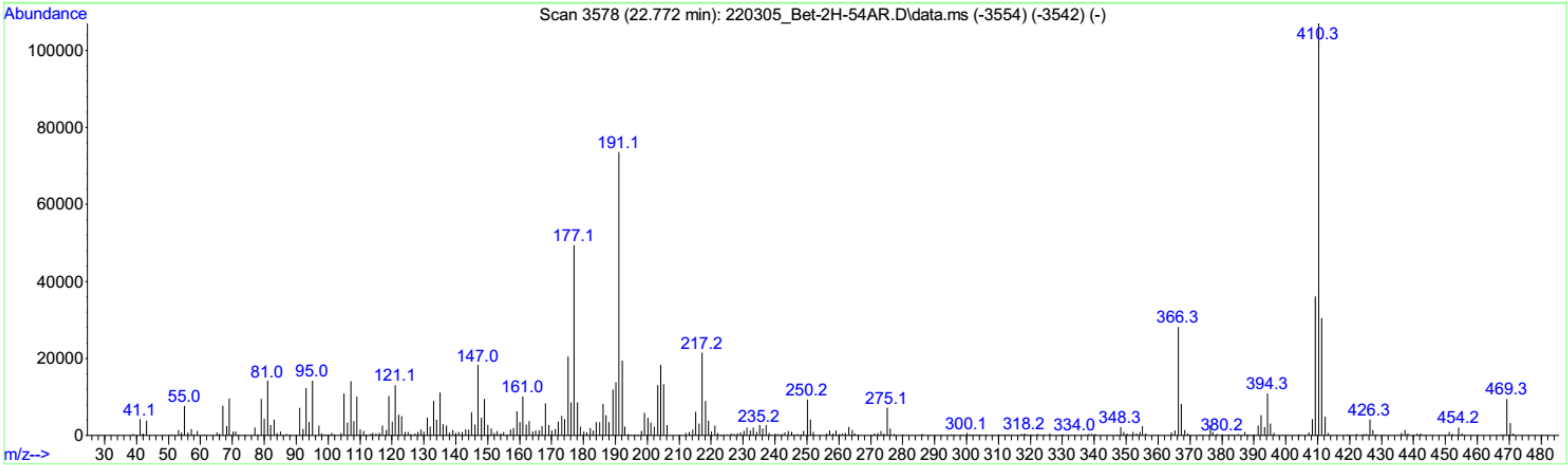

28-Benzoyloxy-2-cyano-4-oxo-3,4-seco-3,23-dinorlupan (20).

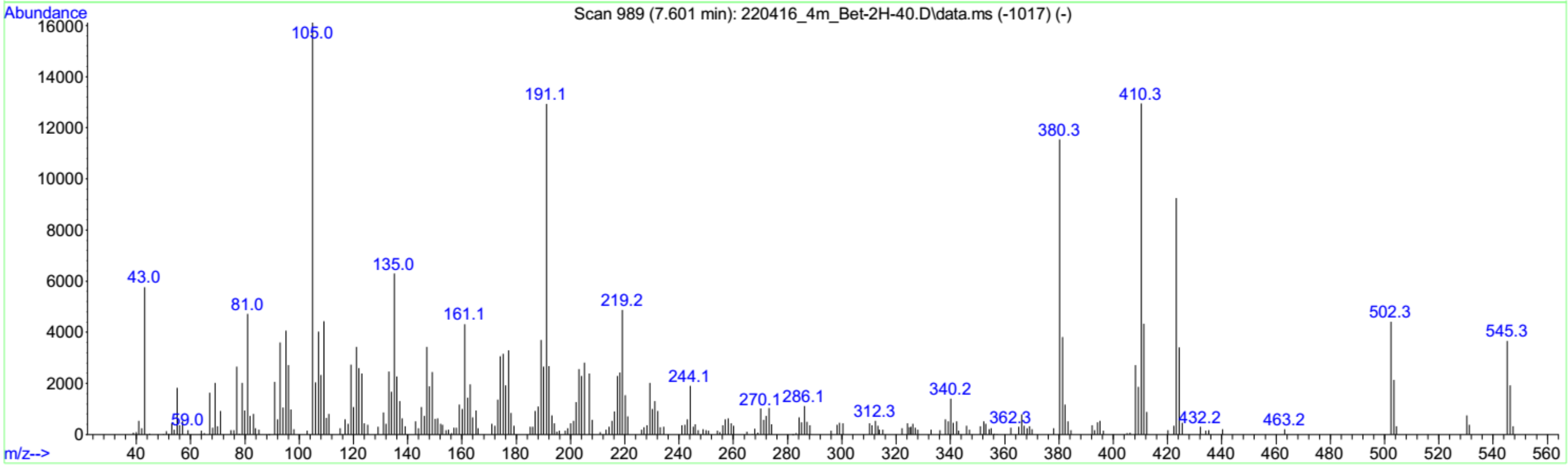

2-Cyano-4-oxo-3,4-seco-3,23-dinorlupan-28-oic acid methyl ester (21).

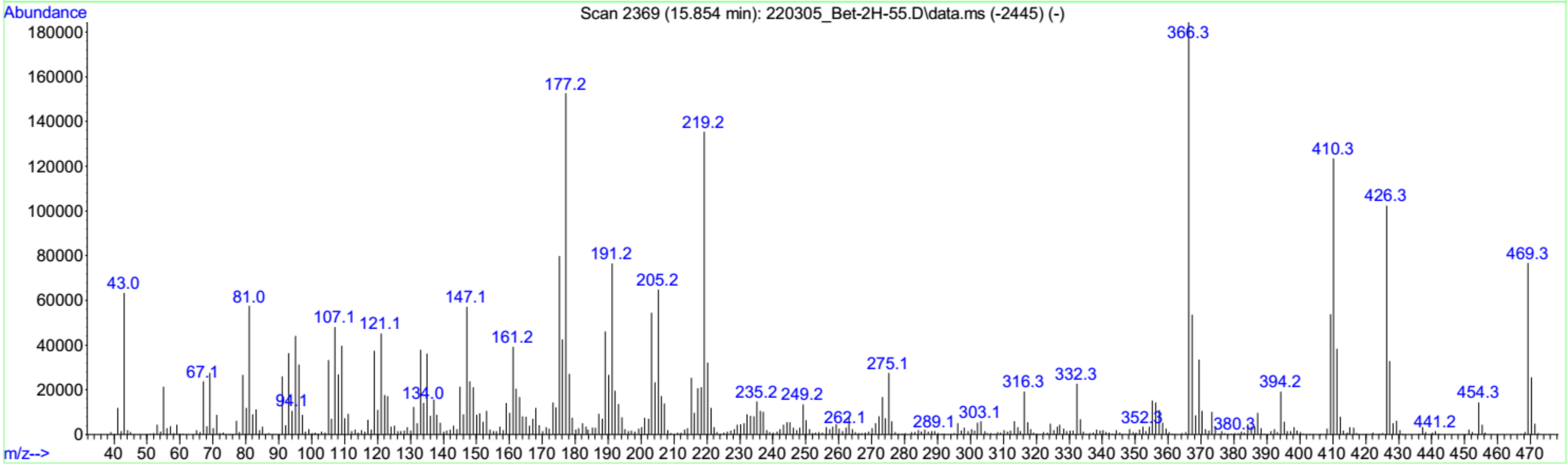

2-Cyano-28-hydroxy-3-norlup-4(23)-ene (22).

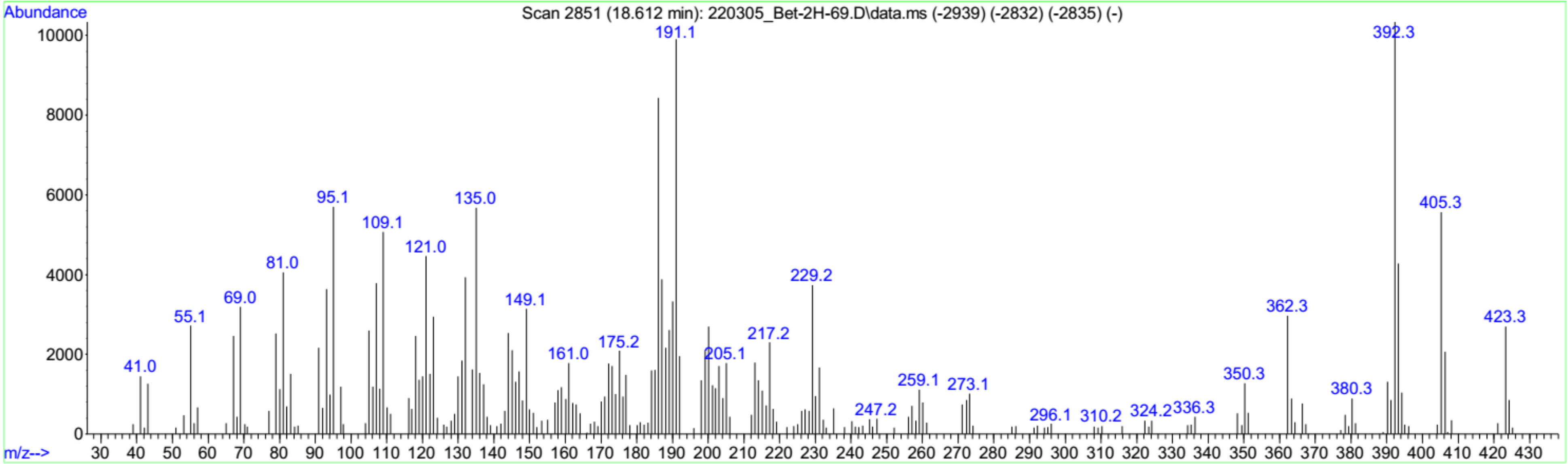

2-Cyano-3-norlup-4(23)-ene-28-oic acid methyl ester (23).

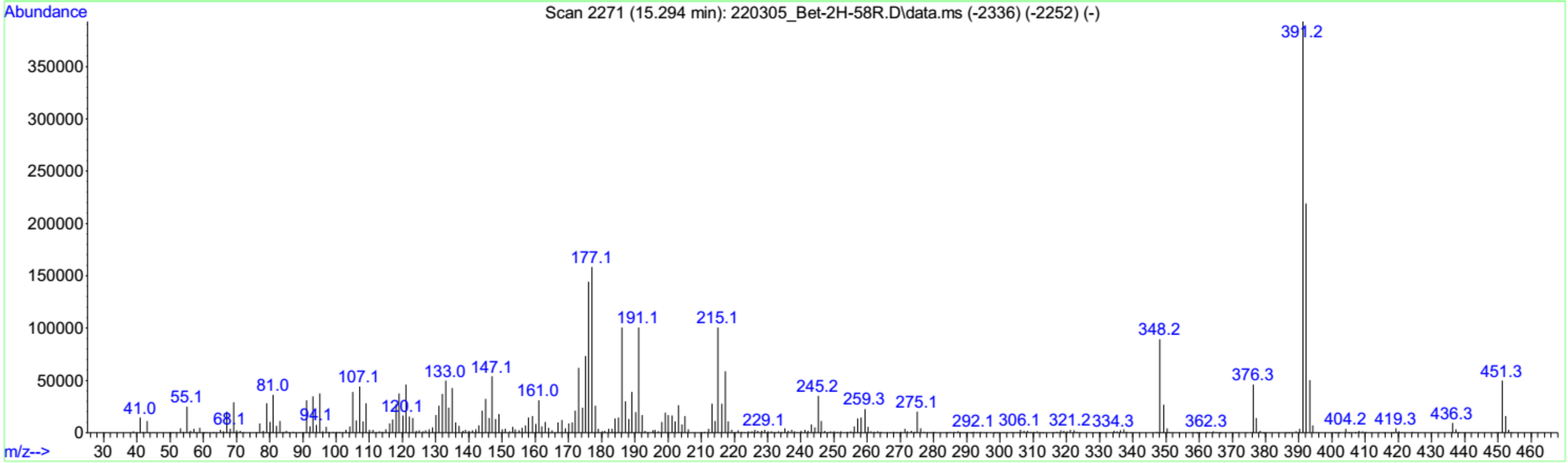

28-Benzoyloxy-24-bromo-2-cyano-4-oxo-3,4-seco-3,23-dinorlupan (28).

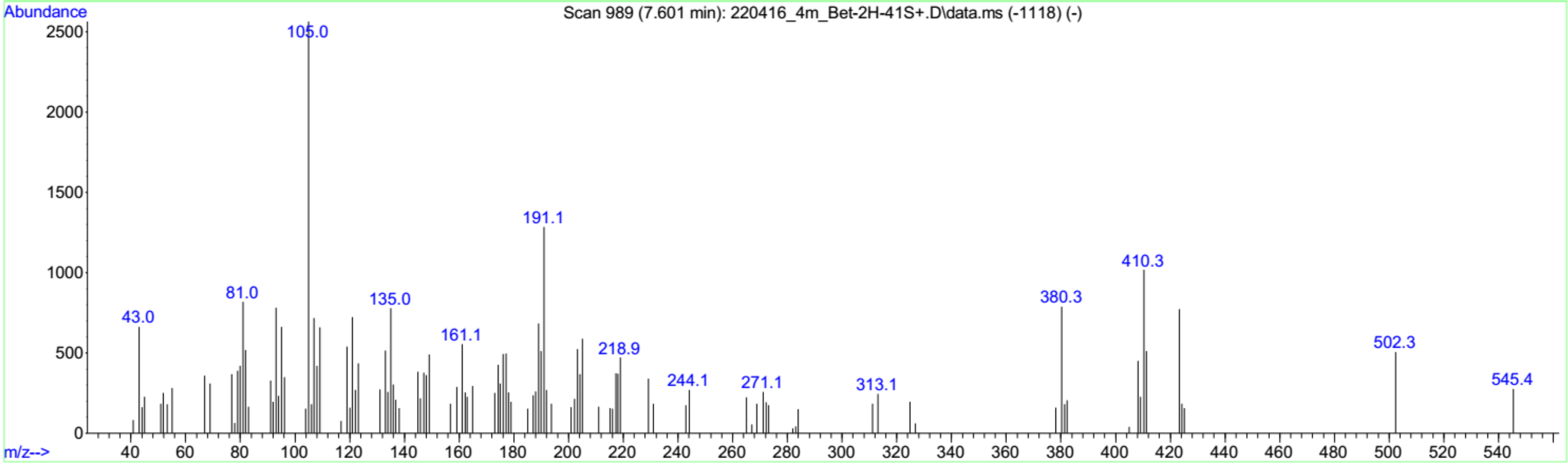

24-Bromo-2-cyano-4-oxo-3,4-seco-3,23-dinorlupan-28-oic acid methyl ester (30)

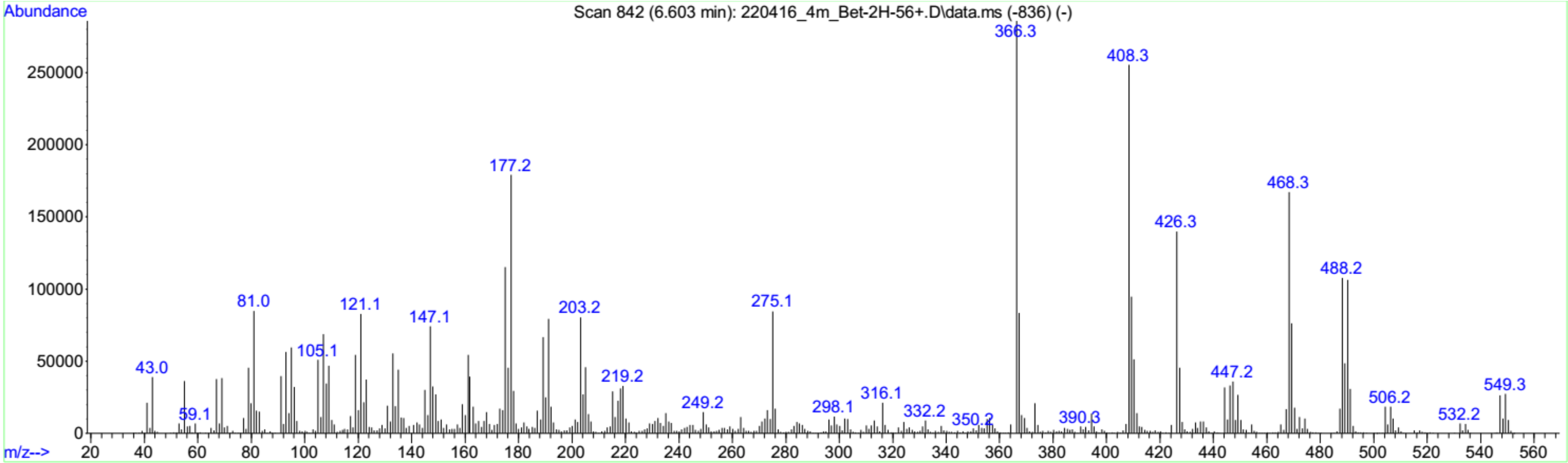

2-Cyano-24,24-dibromo-4-oxo-3,4-seco-3,23-dinorlupan-28-oic acid methyl ester (31).

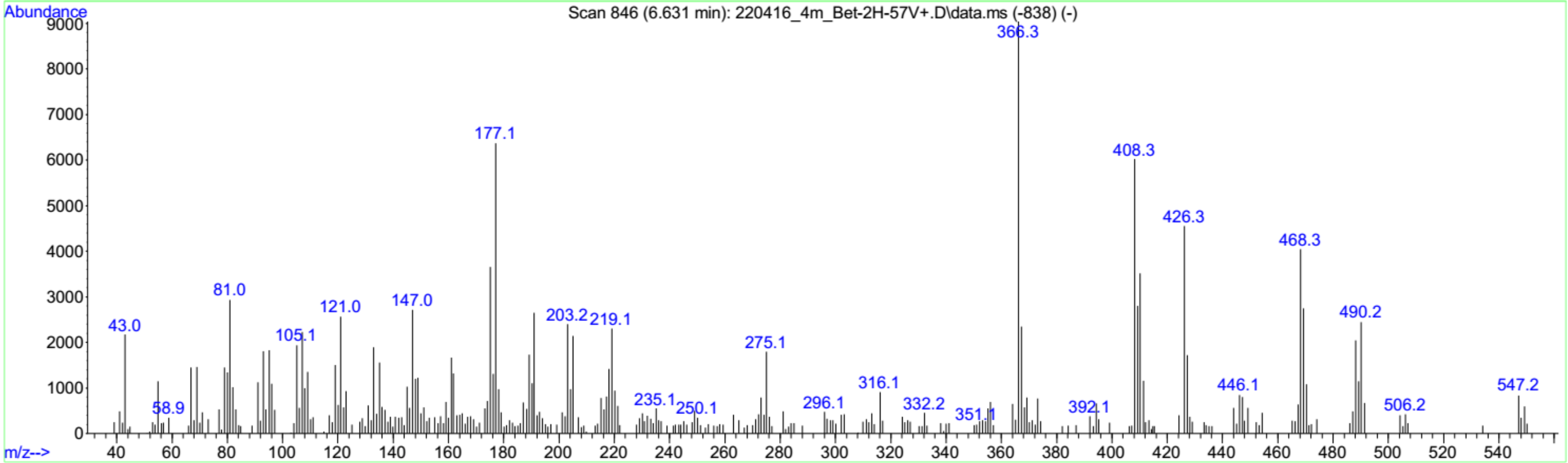

Table S1. The cytotoxic effect of 3,4-seco-lupane derivatives **4–23** and **28–31** against human cancer and human embryonic kidney cell lines

Compounds	Cell Lines IC$_{50}$ (mean ± SD), μM									
	**HBL-100/Dox**	**HBL-100**	**MCF-7**	**HCT116**	**RD TE32**	**MS**	**A549**	**PC-3**	**HEpG2**	**HEK293**
**4**	>100	>100	>200	>200	>100	>100	>100	>100	>100	>100
**5**	>100	>200	>200	>200	>100	>100	>100	>100	>100	>200
**6**	>200	>100	>200	>200	>200	>100	>100	>100	>100	>200
**7**	>200	>200	>100	>200	>200	>100	>100	>100	>100	>100
**8**	>200	>100	>100	>200	>200	>100	>100	>100	>100	>100
**9**	>100	>200	>200	>100	>100	>100	>100	>100	>100	>100
**10**	>200	>100	>100	>200	>100	>100	>100	>100	>100	>200
**11**	>200	73.31±1.51	>100	>200	>200	>100	>100	>100	>100	>200
**12**	63.03±1.92	36.61±1.44	64.21±3.01	51.04±3.67	98.56±2.33	>200	44.40±2.44	55.66±4.89	>100	76.53±2.49
**13**	>200	>100	>100	>100	>100	>100	>100	>100	>100	>200
**14**	54.01±1.82	46.94±2.22	56.73±3.72	49.90±0.10	49.10±1.37	69.10±1.03	63.21±1.42	28.27±2.29	>100	77.77±2.90
**15**	>100	>100	>100	>100	>100	>100	>100	>100	>100	>100
**16**	>200	>100	>100	>200	>100	>100	>100	>100	>100	>100
**17**	>200	>200	>100	>100	>100	>100	>100	>100	>100	>100
**18**	>200	>100	>100	>200	>200	>100	>100	>100	>100	>100
**19**	21.14±2.23	16.03±0.94	47.90±0.26	28.23±2.06	25.17±0.11	22.20±2.00	25.93±1.75	17.22±0.69	31.80±2.68	34.62±1.09
**20**	>200	>200	>100	>100	>100	>100	>100	>100	>100	>100
**21**	>200	>100	97.47±4.21	>100	>100	>100	>100	>100	>100	>100
**22**	>100	>100	89.31±3.01	74.25±2.56	84.68±3.68	>100	>200	36.46±1.31	>100	>100
**23**	>100	>100	70.24±3.09	>100	>100	>100	>200	>200	>200	>100
**28**	>200	>100	86.14±10.14	>100	>100	>100	>100	>100	>100	>100
**29**	>200	>100	48.96±5.33	>200	73.23±5.24	>100	>100	>100	>100	>100
**30**	4.53±0.21	3.62±0.33	12.04±0.06	13.50±0.34	7.05±0.91	45.97±2.20	27.73±0.31	34.07±1.28	49.60±0.50	24.17±0.45
**31 (MK)**	1.80±0.14	0.99±0.001	7.06±0.72	3.86±0.08	0.66±0.04	16.47±1.53	7.01±0.21	10.35±1.27	16.55±0.41	11.47±0.56
**Dox**	29.61±0.94	0.35±0.03	0.38±0.09	1.30±0.34	1.27 ±0.03	1.29±0.16	2.04±0.21	12.60±0.20	1.78±0.30	0.44±0.04

Table S2. Selectivity index (SI) values of compounds **12**, **14**, **19**, **30** and **31**

Cell lines	SI range of active compounds					
	**12**	**14**	**19**	**30**	**31 (MK)**	**Dox**
MCF-7	1.19	1.37	0.72	2.02	1.62	1.00
HCT116	1.50	1.56	1.23	1.79	2.95	0.31
RD TE32	0.78	1.58	1.37	3.41	16.43	0.31
MS	<0.38	1.13	1.56	0.53	0.70	0.31
A549	1.72	1.23	1.34	0.87	1.64	0.20
PC-3	1.37	2.75	2.01	0.71	1.11	0.03
HEpG2	<0.77	<0.78	1.09	0.49	0.69	0.22
HBL-100	2.09	1.66	2.16	6.72	11.50	1.00
HBL-100/Dox	1.21	1.44	1.64	5.37	6.39	0.01

Table S3. Cell cycle distribution of HBL-100 cells treated with **MK** and Dox (at doses corresponding to IC_{50} values) for 12-24 h.

Time, h	**Samples**	**Cell cycle of HBL-100, %**			
		**subG0/G1**	**G0/G1**	**S-phase**	**G2/M**
**12**	Untreated cells	1.13±0.13	63.27±0.13	22.34±0.31	14.38±0.15
	**MK**	1.44±0.44	59.78±0.94	24.35±0.43	15.88±0.50
	Dox	1.43±0.02	25.46±0.03	48.19±0.09	26.35±0.05
**13**	Untreated cells	0.81±0.06	60.44±0.94	24.85±0.96	14.71±0.02
	**MK**	1.74±0.05	56.56±0.16	26.74±0.54	16.70±0.71
	Dox	1.46±0.01	22.99±0.19	46.86±0.22	28.03±0.03
**14**	Untreated cells	1.19±0.09	61.50±1.47	24.13±1.67	15.41±0.20
	**MK**	1.74±0.18	56.69±0.01	26.35±0.16	16.96±0.16
	Dox	2.37±0.03	22.99±0.24	48.25±0.51	28.76±0.75
**15**	Untreated cells	1.01±0.12	63.87±1.01	23.19±0.19	12.94±1.20
	**MK**	2.38±0.42	57.83±1.04	25.88±0.49	16.29±0.55
	Dox	1.72±0.15	17.32±0.29	57.27±0.14	25.41±0.16
**16**	Untreated cells	1.04±0.02	59.62±0.75	24.75±0.41	15.63±0.34
	**MK**	4.89±1.93	56.91±0.07	24.26±0.08	18.83±0.14
	Dox	2.28±0.19	24.52±1.28	61.28±2.01	14.20±3.22
**17**	Untreated cells	0.89±0.12	56.67±0.46	26.41±0.76	16.92±1.23
	**MK**	3.50±0.42	55.78±0.79	25.83±0.24	18.39±1.04
	Dox	1.88±0.26	12.11±1.06	56.17±1.64	31.72±0.57
**18**	Untreated cells	0.93±0.14	58.31±1.45	27.97±0.65	14.10±0.79
	**MK**	4.39±0.82	56.81±0.03	27.81±0.04	15.38±0.01
	Dox	3.04±0.91	8.10±0.01	55.42±0.92	36.48±0.93
**24**	Untreated cells	1.30±0.42	55.37±1.15	27.97±0.64	16.66±0.50
	**MK**	8.22±0.59	66.48±1.33	29.09±0.89	4.43±0.50
	Dox	2.04±0.45	4.88±0.78	76.69±0.75	18.43±0.75

Table S4. Cell cycle distribution of HBL-100/Dox cells treated with **MK** and Dox (at doses corresponding to IC_{50} values) for 12-24 h.

Time, h	**Samples**	**Cell cycle of HBL-100/Dox, %**			
		**subG0/G1**	**G0/G1**	**S-phase**	**G2/M**
	Untreated cells	0.20±0.14	53.09±3.62	30.10±3.09	16.81±0.53
**12**	**MK**	4.84±1.48	50.93±0.43	30.69±0.04	18.38±0.34
	Dox	2.21±0.49	39.62±0.67	50.74±0.99	9.63±1.66
	Untreated cells	1.24±0.41	51.13±0.58	34.79±1.68	14.08±2.27
**13**	**MK**	4.89±0.48	47.82±0.67	34.76±0.20	17.42±0.46
	Dox	2.40±0.55	34.77±0.94	53.50±2.04	11.74±1.09
	Untreated cells	1.25±0.19	43.57±2.58	37.17±1.69	19.26±0.89
**14**	**MK**	4.87±0.60	51.68±1.34	33.78±1.08	14.54±0.25
	Dox	2.24±1.12	32.36±0.50	57.31±0.68	10.30±0.17
	Untreated cells	1.40±0.35	45.45±2.04	36.48±1.20	18.07±0.84
**15**	**MK**	5.06±0.09	52.12±1.18	33.36±2.57	14.52±1.40
	Dox	1.37±0.47	35.29±3.38	56.60±1.88	8.10±1.49
	Untreated cells	1.81±0.11	50.52±0.66	31.97±1.23	17.51±0.56
**16**	**MK**	6.55±0.11	51.84±0.88	25.94±2.58	22.22±1.70
	Dox	2.30±0.47	32.41±0.43	56.07±0.72	11.52±1.13
	Untreated cells	1.87±0.22	40.34±1.76	38.61±0.60	21.16±0.03
**17**	**MK**	9.08±0.39	52.16±1.94	28.02±1.34	19.82±0.60
	Dox	2.12±0.54	26.86±0.57	57.98±1.34	15.16±0.77
	Untreated cells	1.72±0.74	39.26±0.57	39.61±0.60	21.13±0.03
**18**	**MK**	10.42±0.98	57.26±1.18	17.19±2.31	25.55±1.12
	Dox	3.70±1.08	28.59±0.59	56.22±1.37	15.19±1.98
	Untreated cells	2.23±0.34	51.24±1.17	35.58±1.10	13.19±0.07
**24**	**MK**	13.47±0.18	64.14±0.55	8.99±0.42	26.87±0.13
	Dox	2.44±0.68	19.60±2.59	73.17±2.11	4.76±1.02

Table S5. Annexin V-FITC and PI-stained HBL-100 and HBL-100/Dox cells

**Compound**	**Viable cells, %**	**Early apoptotic cells, %**	**Late apoptotic cells, %**	**Dead cells, %**
		**HBL-100 cells**		
Untreated cells	97.30±0.35	2.12±0.20	0.15±0.04	0.42±0.12
Dox	83.89±0.82	12.35±0.42	1.93±0.29	1.80±0.31
**MK** (IC_{50})	89.58±0.94	4.43±0.49	3.59±0.82	2.69±0.13
**MK** ($2xIC_{50}$)	76.73±1.19	7.29±0.29	10.37±1.01	5.59±0.46
		**HBL-100/Dox cells**		
Untreated cells	97.37±0.25	1.41±0.25	0.27±0.14	1.05±0.34
Dox	95.74±1.10	2.56±0.37	0.82±0.55	0.84±0.24
**MK** (IC_{50})	87.82±0.45	5.04±0.52	4.74±0.04	2.52±0.08
**MK** ($2xIC_{50}$)	78.69±1.29	5.50±0.44	10.94±0.82	4.59±0.45

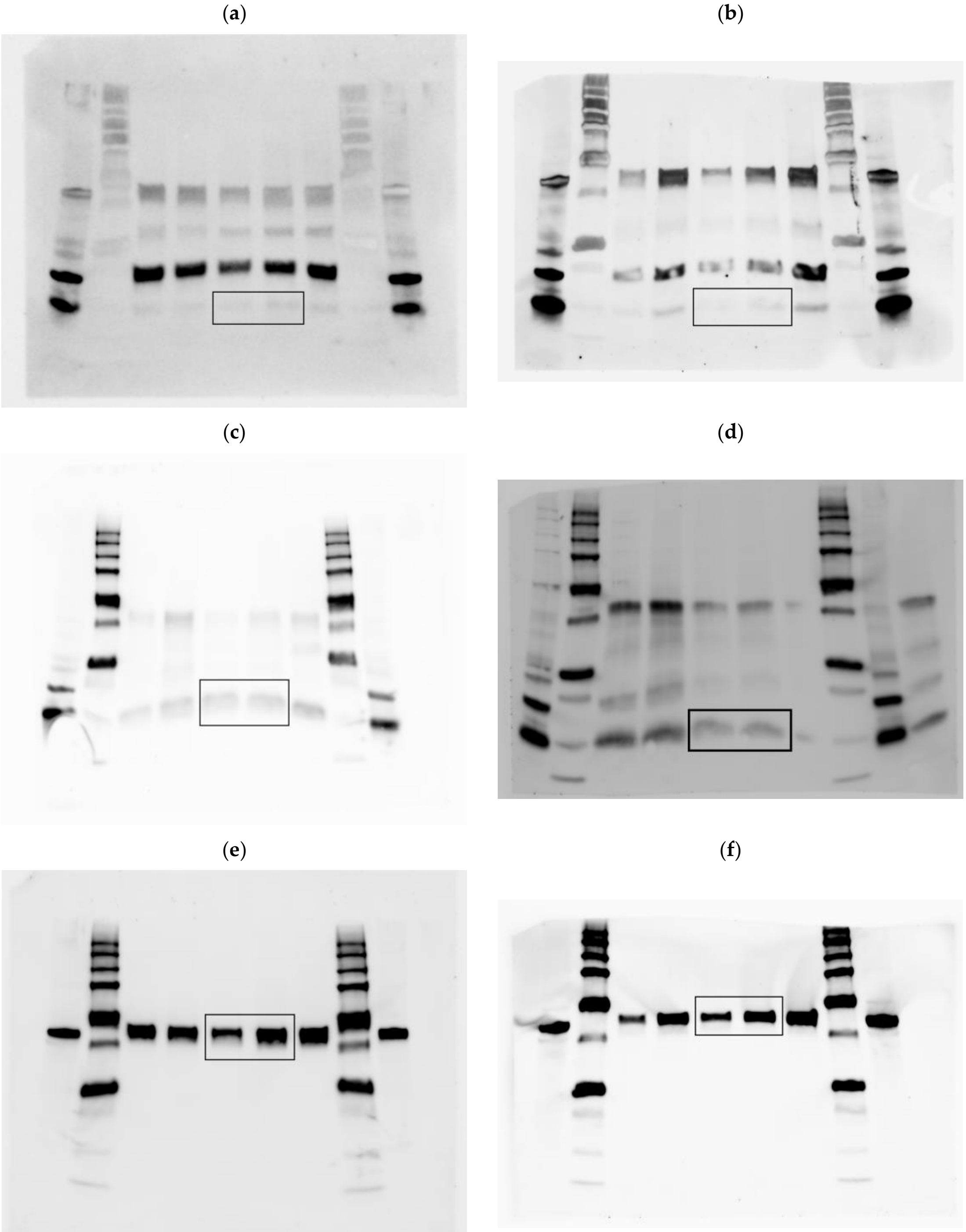

Figure S3. Expression of cytochrome *c* and ß-Actin in HBL-100 and HBL-100/Dox cells after **MK**-treatment for 16 h. Black squares indicate lines used in this study:

(**a**) WB of cytosolic fraction of the HBL-100 cell lysates after **MK**-treatment for 16 h with 1.0 µM: line 1, 3, 4, 7, 9 – inapplicable in this research, lines 2, 8 - MW, line 5 – control, line 6 - **MK**-treatment with 1.0 µM. Staining with primary AT of cytochrome *c* (18 kDa)

(**b**) WB of cytosolic fraction of the HBL-100/Dox cell lysates after **MK**-treatment for 16 h with 1.8 µM: line 1, 3, 4, 7, 9 – inapplicable in this research, lines 2, 8 - MW, line 5 – control, line 6 - **MK**-treatment with 1.0 µM. Staining with primary AT of cytochrome *c* (18 kDa)

(**c**) WB of mitochondrial fraction of the HBL-100 cell lysates after **MK**-treatment for 16 h with 1.0 µM: line 1, 3, 4, 7, 9 – inapplicable in this research, lines 2, 8 - MW, line 5 – control, line 6 - **MK**-treatment with 1.0 µM. Staining with primary AT of cytochrome *c* (18 kDa)

(**d**) WB of mitochondrial fraction of the HBL-100/Dox cell lysates after **MK**-treatment for 16 h with 1.8 μM: line 1, 3, 4, 7, 9 – inapplicable in this research, lines 2, 8 - MW, line 5 – control, line 6 - **MK**-treatment with 1.8 μM. Staining with primary AT of cytochrome *c* (18 kDa)

(**e**) WB of cytosolic fraction of the HBL-100 cell lysates after **MK**-treatment for 16 h with 1.0 μM: line 1, 3, 4, 7, 9 – inapplicable in this research, lines 2, 8 - MW, line 5 – control, line 6 - **MK**-treatment with 1.0 μM. Staining with primary AT of ß-Actin (45 kDa)

(**f**) WB of cytosolic fraction of the HBL-100/Dox cell lysates after **MK**-treatment for 16 h with 1.8 μM: line 1, 3, 4, 7, 9 – inapplicable in this research, lines 2, 8 - MW, line 5 – control, line 6 - **MK**-treatment with 1.0 μM. Staining with primary AT of β-Actin (45 kDa)

(**a**) (**b**)

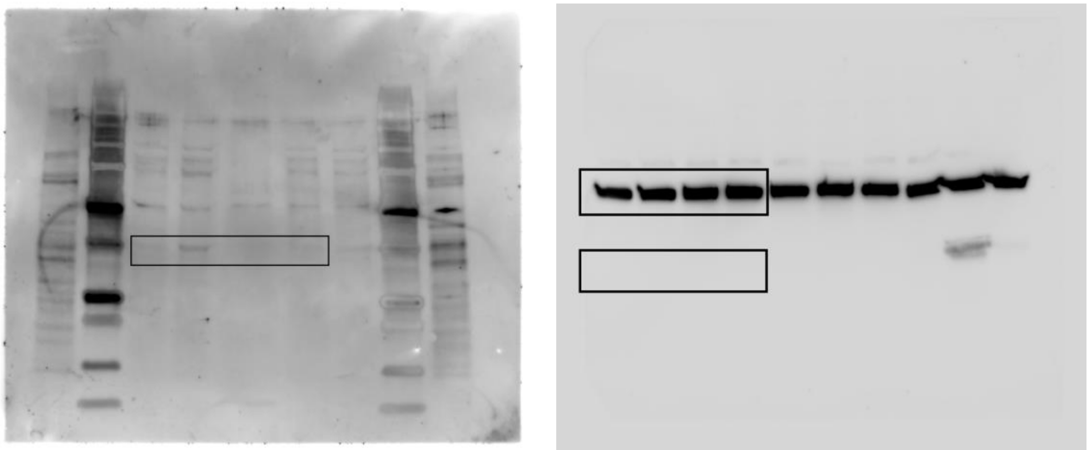

Figure S4. Expression of caspase-9, -8 and ß-Actin in HBL-100 and HBL-100/Dox cells after **MK**-treatment for 16 h. Black squares indicate lines used in this study:

(**a**) WB of cytosolic fraction of the HBL-100 and HBL-100/Dox cell lysates after **MK**-treatment for 16 h with 1.0 and 1.8 μM, respectively: line 1, 7, 9 – inapplicable in this research, lines 2, 8 - MW, line 3 – untreated HBL-100/Dox cells, line 4 - **MK**-treated HBL-100/Dox cells, line 5 - untreated HBL-100 cells, line 6 - **MK**-treated HBL-100 cells. Staining with primary AT of caspase 9 (37 kDa)

(**a**) WB of cytosolic fraction of the HBL-100 and HBL-100/Dox cell lysates after **MK**-treatment for 16 h with 1.0 and 1.8 μM, respectively: lines from 5 to 10 – inapplicable in this research, line 1 – untreated HBL-100 cells, line 2 - **MK**-treated HBL-100 cells, line 3 - untreated HBL-100/Dox cells, line 4 - **MK**-treated HBL-100/Dox cells. Staining with primary AT of caspase 8 (18 kDa) and β-Actin (45 kDa)

(**a**)

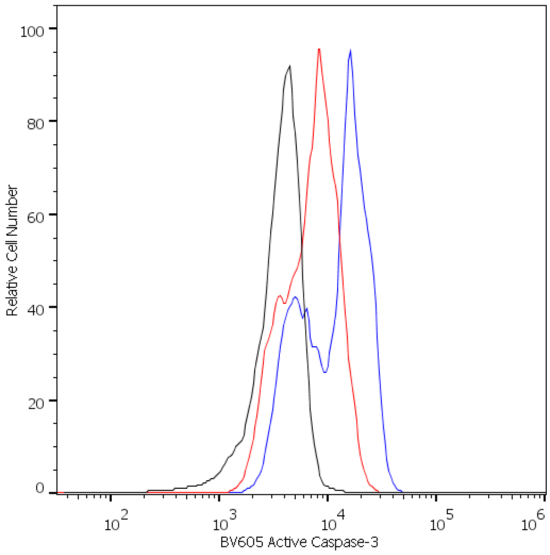

Compound	gMIF
Untreated cells	3213±217
**MK** (IC_{50})	6755±301
**MK** ($2\times IC_{50}$)	11048±278

(**b**)

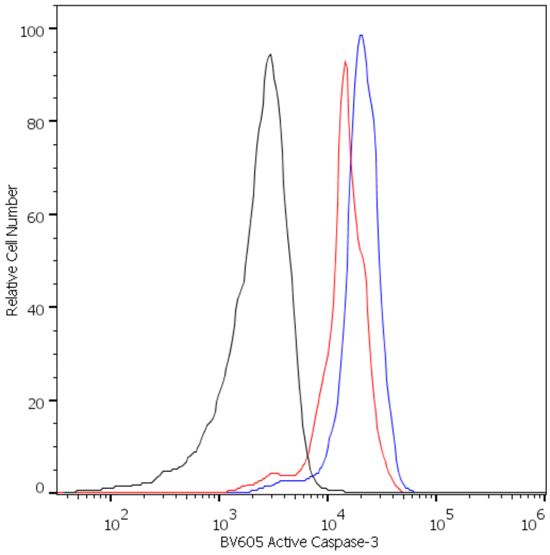

Compound	gMIF
Untreated cells	2349±199
**MK** (IC_{50})	13428±202
**MK** ($2\times IC_{50}$)	19252±347

Figure S5. Flow cytometric analysis of Active Caspase-3 expressed by apoptotic HBL-100 (**a**) and HBL-100/Dox (**b**) cells. Cells were untreated (black line histogram) or **MK**-treated (IC_{50} – red line histogram, $2\times IC_{50}$ – blue line histogram) for 24 h to induce apoptosis.
